# Effectiveness and Safety of Nusinersen and Risdiplam in Spinal Muscular Atrophy: A Systematic Review

**DOI:** 10.1002/acn3.70274

**Published:** 2025-12-05

**Authors:** Amin Mehrabian, Peter Auguste, Amy Grove, Anna Brown, Janette Parr, Mubarak Patel, Furqan Butt, Jeremiah Donoghue, Mehdi Yousefi, Jo Parsons

**Affiliations:** ^1^ Centre for Evidence and Implementation Science University of Birmingham Birmingham UK

**Keywords:** effectiveness, nusinersen, risdiplam, safety, SMA, spinal muscular atrophy

## Abstract

**Objective:**

Spinal Muscular Atrophy (SMA) is a rare genetic disorder marked by progressive muscle weakness and mobility loss. It has a profound physical, emotional and social impact on patients and caregivers, requiring comprehensive medical and supportive care. SMA is classified into Types 1–4, with some individuals identified presymptomatically. This systematic review examined the safety and effectiveness of nusinersen and risdiplam for treating SMA.

**Methods:**

We searched research databases, relevant websites and existing systematic reviews. Screening, data extraction and quality assessment were conducted independently by two authors, with discrepancies resolved by a third. Internal quality appraisal ensured methodological rigour. A total of 131 studies reported in 148 sources were included. The review is registered with PROSPERO (CRD42024512226).

**Results:**

Both treatments showed improvements in motor function and milestones, with high survival rates across most SMA types. Motor function improvements were consistent, but other outcomes—such as bulbar and respiratory function, and ventilation needs—were variable. Adverse events were common across all treatments and SMA types, with some serious cases reported, including deaths in Types 1 and 2.

**Interpretation:**

This comprehensive review highlights the clinical effectiveness and safety of nusinersen and risdiplam across all SMA types. However, variability in outcomes and limited comparative data introduce uncertainty. The findings underscore the need for more high‐quality randomised controlled trials to strengthen the evidence base for SMA treatment.

**Abbreviations**
6MWT: 6‐Minute Walk Test
AE: adverse event
ALSFRS‐R: Amyotrophic Lateral Sclerosis Functional Rating Scale—Revised
CGI‐I: Clinical Global Impression–Improvement
CHOP INTEND: Children's Hospital of Philadelphia Infant Test of Neuromuscular Disorders
CMAP: Compound Muscle Action Potential
ECG: electrocardiogram
EK2: Egen Klassifikation 2
GFFS: Global Fatigue and Function Scale
HFMSE: Hammersmith Functional Motor Scale—Expanded
HINE‐2: Hammersmith Infant Neurological Examination, section 2
HRQoL: Health‐related quality of life
MMT: Manual Muscle Testing
MRC‐SS: Medical Research Council Sum Score
MTA: Multiple Technology Appraisal
MUNE: Motor Unit Number Estimation
NHS: National Health Service
NICE: National Institute for Health and Care Excellence
PRISMA: Preferred Reporting Items for Systematic Reviews and Meta‐Analyses
PROSPERO: Prospective Register of Systematic Reviews
RCT: randomised‐controlled trial
RHS: Revised Hammersmith Scale
RULM: revised upper limb module
SLR: systematic literature review
SMA: spinal muscular atrophy
SMN: survival motor neuron
TUG: Timed Up and Go Test
UK: United Kingdom
WHO: World Health Organization

## 1 Introduction

Spinal muscular atrophy (SMA) is a rare genetic disorder characterised by muscle weakness and progressive loss of mobility (prenatal to 18 years). It has a significant physical, emotional and social impact on patients and their caregivers, necessitating comprehensive medical and supportive care [1, 2, 3]. SMA is classified into Types 1–4, with some patients recognised as presymptomatic [4]. In the human body, the Survival Motor Neuron (SMN) protein is essential for the survival and function of motor neurons, which are nerve cells that control muscle movement. It is produced by the SMN1 and SMN2 genes. A deficiency in SMN protein, due to mutations in the SMN1 gene, leads to SMA, a condition characterised by muscle weakness and atrophy [5, 6, 7]. However, SMA types present differently, from severe muscle weakness/respiratory issues in infants to milder muscle weakness in adults [8, 9].

Whilst there is no cure for SMA, pharmacological interventions for SMA treatment have advanced. The outcome of treatment for presymptomatic SMA differs with the number of SMN2 copies [10]. Primary treatments for SMA are nusinersen (Spinraza) and risdiplam (Evrysdi). Nusinersen received approval for the treatment of SMA in the United Kingdom [11], United States [12, 13], Europe [14], Canada [15] and Australia [16] between 2016 and 2019. Subsequently, risdiplam was approved in these same regions between 2020 and 2021 [14, 17, 18, 19, 20].

Nusinersen is an antisense oligonucleotide that increases the production of functional SMN protein by targeting the SMN2 gene. It is administered via spinal injections and is recommended for all patients with confirmed presymptomatic SMA or types 1, 2, 3 and 4 SMA [21, 22].

Risdiplam developed in 2021, is available to SMA type 1, 2 and 3 patients or presymptomatic individuals with 1–4 SMN2 copies. It is administered orally and is designed for patients with SMA types 1, 2 and 3 or with 1–4 SMN2 copies [17, 23, 24].

In addition to pharmacological treatments, supportive measures such as strength and flexibility exercises and breathing and feeding assistance devices are recommended for people with SMA [25, 26, 27].

Previous systematic reviews examined the clinical efficacy of treatments for SMA [28, 29, 30, 31, 32, 33, 34, 35, 36, 37, 38, 39], (one focused on adverse events exclusively) [38]. Most reviews examined different treatment options (including different combinations of treatments including onasemnogene abeparvovec [28, 29, 31], olexsomine [36, 37] and combination therapies [31, 36]), and were limited to SMA Types 1, 2 and 3. Reviews were often limited to paediatric populations and included numerous outcomes and study designs. A recent systematic review examined the clinical effectiveness of nusinersen and risdiplam in a narrower population than the current review, examining outcomes in Asian patients with Type 2–4 SMA only [39], and did not include any studies of risdiplam, limiting the relevance of this review when considering the effectiveness of both treatments.

Both nusinersen and risdiplam are currently under evaluation by the National Institute for Health and Care Excellence (NICE) for a multiple technology appraisal (MTA) for SMA patients within National Health Service (NHS) practice [40]. No comprehensive systematic review has been conducted to encompass both drugs with a substantial list of outcomes that aligns with the NICE‐wide topic scope to provide a robust source of evidence. We therefore identified an unmet need to examine the clinical effectiveness and safety of nusinersen and risdiplam.

Our systematic review addresses this unmet need by evaluating the clinical effectiveness and safety of nusinersen and risdiplam across all SMA types (0–4), including presymptomatic patients, up to January 2025. Unlike previous reviews, we applied no restrictions on publication year, included both real‐world evidence and clinical trials, and adopted a global perspective. Where applicable, we used Cochrane's risk of bias tools to ensure methodological rigour and considered all reported outcomes (such as bulbar, respiratory, motor functions, mortality, etc.). This comprehensive approach enhances the reliability and relevance of our findings for informing clinical decision‐making and clinical practice.

Therefore, this systematic review evaluates the safety and effectiveness of nusinersen, and risdiplam for the treatment of all types of SMA (types 0, 1, 2, 3, 4) and presymptomatic patients up to February 2025.

## 2 Methods

This systematic review was performed following the principles outlined in the Cochrane Handbook for Systematic Reviews of Interventions [41], and the protocol registered on the International Prospective Register of Systematic Reviews, (PROSPERO) (CRD42024512226), and can be found in the Tables S1 and S2 [8]. The review is reported according to the Preferred Reporting Items for Systematic Reviews and Meta‐Analyses (PRISMA) guidelines [42]. The PRISMA checklist can be found in Data S3.

### 2.1 Searches and Screening

Searches were built by an information specialist (AB) using terms for SMA, nusinersen and risdiplam and used both free text keywords and, where available, thesaurus (MeSH/EMTREE) terms. Searches were limited to studies published in the English language. Searches were developed in Embase (via Ovid) and checked for accuracy by a second information specialist (RC) for accuracy and completeness before being translated for other sources (details provided in the Data S4).

Searches were conducted on 29th January 2024 and updated on 13th January 2025 in Embase (Ovid); MEDLINE All (Ovid); Cochrane CENTRAL (Wiley); International HTA database (INAHTA); Science Citation Index and Conference Proceedings (Web of Science), ClinicalTrials.gov, WHO International Clinical Trials Registry Platform. Websites of selected international HTA and medicines approval agencies (NICE, Scottish Medicines Consortium, Canadian Agency for Drugs and Technologies in Health, Institute for Clinical and Economic Review, U.S. Food and Drug Administration, Medicines and Healthcare Products Regulatory Agency and European Medicines Agency) were searched on 25th January 2024 and updated on 16th January 2025. Reference lists of included studies, and included studies in relevant systematic reviews were screened for any additional studies eligible for inclusion. Records were exported to EndNote 21, where duplicates were systematically identified and removed.

### 2.2 Inclusion and Exclusion Criteria

Titles and abstracts were screened according to the criteria listed in Table 1, by two reviewers independently, and any conflicts were resolved by a third reviewer. Full texts of all potentially eligible studies were retrieved and screened independently by two reviewers, and any conflicts were resolved. When conflicts were not resolved, discrepancies were resolved by a third reviewer. Reasons for exclusions at the full text stage were recorded.

**TABLE 1:** Inclusion and exclusion criteria.

Factor	Inclusion criteria	Exclusion criteria
Design	RCTs, and non‐randomised trials, observational studies, SLRs and meta‐analyses^a^	Editorials Commentaries Case reports Case series (where *n* < 20) Conference abstracts Single‐arm studies or studies with no comparator with a small sample‐size (i.e., *n* < 20) or those where no results were reported at 12 months or beyond from initiation of treatment
Interventions	Nusinersen monotherapy Risdiplam monotherapy	Concomitant or previous participation in any investigational drug Any history of cell therapy
Population	People with types 0, 1, 2, 3 or 4 5q SMA, or pre‐symptomatic 5q SMA confirmed with genetic testing with 1 to 4 SMN2 copies	Received spinal fusion surgery following a diagnosis of scoliosis (prohibits safe administration of nusinersen) Hospitalisation or respiratory conditions history or planned at the time of screening or tracheostomy History of surgery for scoliosis or hip fixation Presence of clinically relevant ECG abnormalities before study drug administration Unstable gastrointestinal, renal, hepatic, endocrine or cardiovascular system diseases
Comparators	Established clinical management. Best supportive care The interventions will be compared to each other. In addition, for children aged 12 months and under with a bi‐allelic mutation in the SMN1 gene and present with a clinical diagnosis of type 1 5q SMA or pre‐symptomatically with up to 3 copies of the SMN2 gene Onasemnogene abeparvovec	No exclusion criteria
Outcomes	The outcome measures to be considered include: Motor function (including, where applicable, both age‐appropriate gross motor milestones and fine motor skills) Bulbar function (including, for example, swallowing and ability to communicate) Frequency and duration of hospitalisation Respiratory function Complications of spinal muscular atrophy (including, for example, scoliosis and muscle contractures) Need for non‐invasive or invasive ventilation Stamina and fatigue Mortality Adverse effects of treatment HRQoL (for patients and carers)	No exclusion criteria

Abbreviations: ECG, electrocardiogram; HRQoL, health‐related quality of life; RCT, randomised‐controlled trial; SLR, systematic literature review.

^a^ SLRs and meta‐analyses were included past the abstract screening stage to enable bibliography searching but excluded at the full‐text stage.

### 2.3 Data Extraction

All included studies were extracted by one reviewer using a pre‐defined extraction form (see Data S5). Accuracy checking was completed by a second reviewer. Any conflicts were resolved by a third reviewer. Extracted data included:

- Study characteristics
- Patient baseline characteristics
- Treatment characteristics
- Outcome characteristics for each included outcome reported

### 2.4 Risk of Bias (RoB) Assessment

Studies were assessed for RoB using either the Cochrane RoB assessment tool for RCTs [43]; ROBINS‐I [44] for non‐randomised studies and the Joanna Briggs Institute (JBI) Checklist tool for Case Series/single arm studies [45].

### 2.5 Narrative Synthesis

Data was summarised and tabulated to provide evidence on the clinical effectiveness and safety of nusinersen and risdiplam. A narrative synthesis provides a comprehensive overview and critique of each included study, with emphasis on the consistency of outcomes measured and methodological quality of the studies.

## 3 Results

**FIGURE 1:**
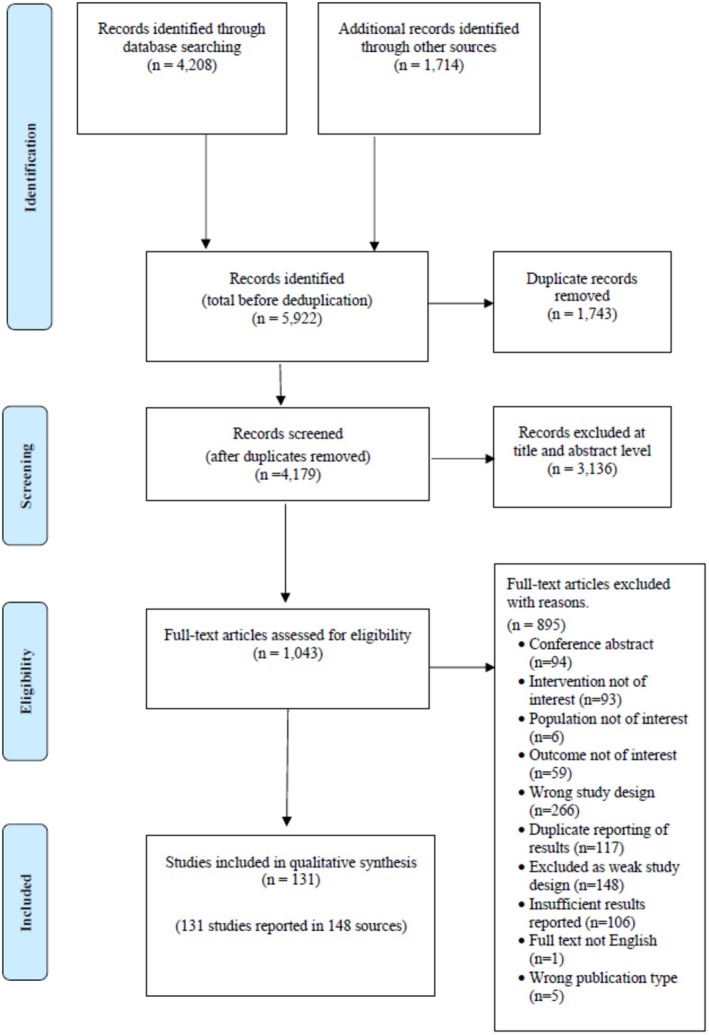
Preferred Reporting for Systematic Review and Meta‐Analysis (PRISMA) flow of search results.

Systematic searches yielded a total of 5922 records. After removing duplicates, 4179 records were screened for inclusion, of which 3136 records were excluded based on title and abstract. The remaining 1043 records were screened at full text, of which 895 studies were excluded, with the reasons for exclusion shown in Figure 1. In total 131 studies, reported in 148 sources were included in this review.

### 3.1 Characteristics of Included Studies

Across the 131 included studies, the populations of interest were people with presymptomatic SMA, and types 1, 2, 3 and 4 SMA. Studies included a wide range of subgroups, including SMA type and SMN2 copy numbers (Full details of included studies and all sources can be found in Table S6). Most studies examined multiple SMA types. Three studies examined presymptomatic SMA alone [46, 47, 48]. Sixteen studies examined type 1 SMA alone [21, 49, 50, 51, 52, 53, 54, 55, 56, 57, 58, 59, 60, 61, 62, 63, 64, 65]. Three studies examined type 2 [66, 67, 68], four examined type 3 [69, 70, 71, 72], eight studies examined type 1 and 2 [73, 74, 75, 76, 77, 78, 79, 80] and 31 examined type 2 and 3 [22, 81, 82, 83, 84, 85, 86, 87, 88, 89, 90, 91, 92, 93, 94, 95, 96, 97, 98, 99, 100, 101, 102, 103, 104, 105, 106, 107, 108, 109, 110]. No studies examined type 4 alone. Mixed type studies were common [111, 112, 113, 114, 115, 116, 117, 118, 119, 120, 121, 122, 123, 124, 125, 126, 127, 128, 129, 130, 131, 132, 133, 134, 135, 136, 137, 138, 139, 140, 141, 142, 143, 144, 145, 146, 147, 148, 149, 150, 151, 152, 153, 154, 155, 156, 157, 158, 159, 160, 161, 162, 163, 164, 165, 166, 167, 168, 169, 170, 171, 172, 173, 174, 175, 176], however only one of these examined all SMA types combined [156].

Nusinersen was the intervention in most included studies (*n* = 112) (See Data S7 for citation list of studies where nusinersen was the intervention). Risdiplam was the primary intervention in 14 included studies [47, 52, 54, 68, 75, 78, 79, 83, 99, 100, 104, 105, 122, 175]. Both nusinersen and risdiplam were included as interventions in three studies [51, 143, 144] and nusinersen and onasemnogene abeparvovec in two studies [53, 124].

Comparators were identified in 33 studies (most studies were single‐arm or observational studies). These included standard of care (*n* = 2) [108, 111], controls (*n* = 15) [66, 71, 77, 89, 91, 96, 101, 113, 124, 126, 130, 159, 166, 168, 171] placebo/sham procedures (*n* = 7) [21, 22, 76, 100, 104, 105, 127] and different dose regimes (*n* = 3) [54, 84, 129], interventions (*n* = 5) [51, 57, 64, 144, 151] and cohorts (*n* = 1) [155].

Countries and settings of included studies can be found in Tables 2 and 3.

**TABLE 2:** Country of included studies.

Country	Number of studies	Study IDs
Germany	17	[71, 78, 80, 82, 83, 87, 88, 95, 102, 103, 118, 130, 135, 150, 153, 169, 173]
United States	12	[63, 79, 84, 85, 113, 115, 126, 128, 132, 140, 144, 159, 171]
Italy	10	[49, 53, 59, 60, 61, 69, 70, 92, 96, 101]
Poland	5	[58, 141, 142, 145, 149]
United Kingdom	3	[50, 75, 172]
Croatia	3	[99, 117, 162]
France	3	[73, 74, 77]
Japan	3	[152, 164, 165]
Australia	3	[120, 133, 139]
Romania	5	[57, 106, 116, 119, 151]
Sweden	2	[89, 143]
Korea	2	[90, 123]
Netherlands	3	[64, 110, 161]
China	9	[91, 107, 121, 126, 138, 146, 157, 170, 174]
Turkey	4	[65, 98, 158, 160]
Israel	2	[55, 86]
Brazil	2	[56, 93]
Belgium	2	[124, 125]
Spain	2	[131, 168]
Hong Kong	1	[136]
Hungary	1	[163]
India	1	[108]
Kuwait	1	[112]
Saudi Arabia	1	[111]
Switzerland	1	[167]
Multiple countries (less than 6)	18	[54, 62, 66, 67, 72, 97, 104, 105, 109, 114, 129, 134, 137, 148, 152, 154, 155, 156]
Multiple countries (6 or more)	12	[21, 22, 46, 47, 52, 68, 100, 122, 127, 147, 154, 176]
Country not reported	6	[48, 51, 81, 94, 166, 175]

**TABLE 3:** Setting of included studies.

Setting	Number of studies	Study IDs
Specific treatment centres for SMA	27	[55, 56, 66, 67, 73, 74, 78, 87, 94, 95, 97, 101, 109, 110, 116, 126, 130, 141, 144, 149, 150, 154, 155, 161, 167, 168, 173]
University hospitals	24	[57, 62, 63, 64, 71, 82, 84, 90, 102, 103, 107, 115, 119, 124, 125, 129, 131, 135, 140, 146, 157, 158, 159, 160, 162, 170]
Hospitals or secondary care	26	[22, 46, 50, 52, 56, 68, 83, 85, 88, 89, 91, 99, 100, 104, 105, 106, 112, 121, 122, 127, 136, 139, 152, 172, 177, 178]
Undefined clinical or health settings	12	[59, 77, 79, 98, 138, 142, 145, 148, 163, 164, 169, 174]
Remote settings	6	[75, 111, 117, 143, 165, 175]
Tertiary care setting	5	[108, 120, 123, 132, 133]
Medical schools or teaching centres	4	[60, 118, 151, 153]
Registries	4	[53, 70, 96, 156]
Early access programme (EAP)	2	[58, 61]
Outpatient settings	2	[54, 171]
Secondary/tertiary centre	2	[69, 92, 147]
A medical centre	1	[86]
Databases	3	[137, 176]
Previous clinical trials	1	[51]
Unspecified or un‐reported setting	14	[21, 47, 48, 49, 65, 72, 76, 80, 81, 113, 114, 128, 134, 166]

### 3.2 Risk of Bias Assessment

Overall ratings of RoB were assessed for each included study and full outcomes of assessments for each domain are presented in the Tables S8–S10.

Most RCTs (*n* = 5) were rated as having ‘some concerns’ whilst one was rated as low risk of bias. Most non‐randomised trials were rated as ‘serious’ risk of bias (*n* = 20), with *n* = 7 rated as ‘critical’ and *n* = 2 rated as ‘moderate’ risk of bias. Most single‐arm studies (*n* = 88) were rated as ‘high quality’, with the remainder (*n* = 8) rated as ‘low quality.’

### 3.3 Assessment of Effectiveness and Safety

#### 3.3.1 Narrative Synthesis

Included studies reported clinical effectiveness and safety of nusinersen and risdiplam using several outcomes. Given the volume of evidence in the 131 included studies, we have reported outcomes grouped by the five groups of SMA Types as reported in included studies. Full outcome data is presented in the Table S10 (motor function outcomes), Table S11 (other outcomes) and Table S12 (adverse events).

#### 3.3.2 Presymptomatic SMA

There were limited studies included that examined pre‐symptomatic SMA, and few reported many outcomes of interest. From the available evidence both nusinersen and risdiplam appear effective for improving or stabilising motor function and motor milestones, stabilising bulbar function, and there was 100% survival and no need for permanent ventilation amongst presymptomatic patients. AEs were reported for all interventions, with some being attributed to treatment, and others being considered as not being treatment related.

Specifically, three studies examined presymptomatic patients [46, 47, 48]. Outcomes: motor function, bulbar function and frequency or duration of hospitalisation. A summary of outcomes can be found in Table 4.

**TABLE 4:** Summary of presymptomatic outcomes.

Outcomes	Nusinersen	Risdiplam
Motor function		
CHOP INTEND	Mean scores: 62.1 (2 copies), 63.4 (3 copies); 7/18 max scores	57.1% max score; 100% ≥ 40 points
HINE‐2	Age‐appropriate development; 13/18 max scores	28.6% max score; all developed complex abilities
WHO Milestones	All could sit; some stood/walked with support	Not specified
CMAP	Stable/increased scores	Increased scores in 5/7 patients
Bulbar function	Maintained swallowing; 76% no choking concerns	Maintained swallowing and feeding
Hospitalisation	Not specified	No hospitalisations
Weight	93% retained body weight	Not specified
Ventilation	No permanent; some temporary	No permanent
Survival	100% survival	100% survival
Adverse events	100% AEs; 48% serious AEs	38.9% skin/tissue AEs; 50% GI AEs; no serious AEs

Abbreviations: AE, adverse event; CHOP INTEND, Children's Hospital of Philadelphia Infant Test of Neuromuscular Disorders; CMAP, compound muscle action potential; GI, gastrointestinal; HINE‐2, Hammersmith Infant Neurological Examination; WHO, World Health Organization.

##### 3.3.2.1 Motor Function

Three studies examined motor function in presymptomatic patients [46, 47, 48] Motor function was measured using the Children's Hospital of Philadelphia Infant Test of Neuromuscular Disorders (CHOP INTEND), Hammersmith Infant Neurological Examination (HINE‐2), World Health Organisation (WHO) motor milestones, Compound Muscle Action Potential (CMAP). Overall, both nusinersen and risdiplam treatments led to improvements (or stabilisation) in motor function [46, 47, 48]. Nusinersen showed remarkable progress in motor milestones, with many patients achieving age‐appropriate motor development and additional WHO motor milestones over time [46, 48]. Patients treated with risdiplam also demonstrated substantial motor development, acquiring complex motor skills and showing increased CMAP scores [46, 47].

##### 3.3.2.2 Bulbar Function

Two studies examining presymptomatic SMA reported bulbar function. One examined the effectiveness of nusinersen [46] and one risdiplam [47]. The majority of participants with presymptomatic SMA maintained their ability to swallow [46, 47] and feed orally [47]. One study examined choking, with 76% (19/25) parents or caregivers not being concerned about their child choking at final assessment [46].

##### 3.3.2.3 Frequency or Duration of Hospitalisation

Only one presymptomatic SMA study reported frequency or duration of hospitalisation [47], reporting no hospitalisations during the receipt of risdiplam during the study.

##### 3.3.2.4 Complications of SMA

No studies of presymptomatic patients receiving nusinersen reported participants requiring permanent ventilation [46, 47], and 100% survival was reported in presymptomatic patients following nusinersen [46, 48].

##### 3.3.2.5 Safety: Adverse Events

Adverse events (AEs) were reported in two presymptomatic studies. One examining nusinersen [46] and one examining risdiplam [47]. For nusinersen, 100% of patients reported AEs, and 48% reported Serious Adverse Events (SAEs) [46]. In risdiplam, 38.9% reported AEs related to skin/tissue disorder and 50% related to gastrointestinal disorders. No SAEs or deaths were reported for presymptomatic patients receiving risdiplam [47]. No AEs were definitely attributed to treatment in [46].

#### 3.3.3 Type 1 SMA

Evidence of effectiveness in Type 1 SMA was available in 16 studies; however outcome reporting varied. Overall improvements were seen in motor function and bulbar function among Type 1 patients following both nusinersen and risdiplam. Few studies measured hospitalisation among this population, and there was mixed effectiveness reported for respiratory function. Ventilation was generally improved, and there was a high rate of survival across treatment. There were generally fewer AEs reported in patients receiving treatment compared to control conditions.

16 studies examined type 1 SMA, reporting motor function, bulbar function, frequency or duration of hospitalisation and respiratory function [21, 49, 50, 51, 52, 53, 54, 55, 56, 57, 58, 59, 60, 61, 62, 63, 64, 65]. A summary of outcomes can be found in Table 5.

**TABLE 5:** Summary of Type 1 outcomes.

Outcome	Nusinersen	Risdiplam
Motor function		
HFMSE	Significant increase in scores	Not specified
CHOP INTEND	Increased/stabilised scores	Increased scores
6MWT	One patient walked 165 m at 48 months	Not specified
HINE‐2	Majority stable; some reached sitting ability; 41% responders	Increased scores; higher doses = more responders
WHO Milestones	Not specified	More milestones reached than patients treated with nusinersen
CMAP	36% responders	Not specified
EK2	5/41 patients improved	Not specified
MMT	Not specified	Greater improvement versus placebo
BSID‐III	Not specified	60% could sit for 5+ s; 40% for 30+ s
Bulbar function	Mixed results: some improvement, stability or decline	Improved feeding/swallowing
Hospitalisation	Not specified	0.94 hospitalisations/year; median 17 nights
Respiratory function	Mixed results: improvements, need for support, reduced ventilation time	Not specified
Scoliosis	Worsening	Not specified
Growth	Not specified	Median change in height‐for‐age: −11
Weight	Not specified	Median change in weight‐for‐age: 3
Ventilation	Less need for permanent ventilation	19%–25% did not need support
Mortality	84% survival; 7/68 died	93% survival; 4/21 died
Adverse events	96% reported AEs; common: headache, fever, vomiting	78% reported AEs; common: respiratory issues
Serious AEs	76% reported SAEs; 1–7 deaths	68% reported SAEs; 24 SAEs. Less SAEs in one study in patients treated with risdiplam than nusinersen
HRQoL	Not specified	Improvements in emotional health, temperament, mood, growth and development; no change in pain/discomfort, parent impact, family cohesion, overall health; decline in physical abilities and general health perceptions

Abbreviations: 6MWT, 6‐Minute Walk Test; AE, adverse event; BSID‐III, Bayley Scales of Infant and Toddler Development, Third Edition; CHOP INTEND, Children's Hospital of Philadelphia Infant Test of Neuromuscular Disorders; CMAP, Compound Muscle Action Potential; EK2, Egen Klassifikation 2; GI, gastrointestinal; HFMSE, Hammersmith Functional Motor Scale—Expanded; HINE‐2, Hammersmith Infant Neurological Examination, section 2; HRQoL, health‐related quality of life; MMT, manual muscle testing; SAE, serious adverse event; WHO, World Health Organization.

##### 3.3.3.1 Motor Function

The Hammersmith Functional Motor Scale Expanded (HFMSE) showed significant score increases following nusinersen treatment, with one study reporting a mean increase from 14.7 to 18.8 at 48 months [61], and another showing a 1.60‐point increase compared to a decline in the control group at 24 months [56]. CHOP INTEND scores improved in most patients across eight studies [21, 56, 57, 58, 59, 60, 61, 65], with some showing score stabilisation [57, 60, 61]. Risdiplam studies indicated higher dose patients had greater score increases [54]. Improvements were also noted in studies combining nusinersen with risdiplam [51] or onasemnogene abeparvovec [53].

In the Six Minute Walk Test (6MWT), one participant improved to walk 165 m at 48 months [61]. Hammersmith Infant Neurological Examination (HINE‐2) studies showed stability in most patients [59], with some achieving the ability to sit [60] and 44.5% improving from baseline at 48 months [61]. Risdiplam studies showed overall score increases and higher doses leading to more responders and better head control [54]. Studies didn't directly compare the two treatments, but more studies of risdiplam showed increased WHO Motor milestones achieved than studies of nusinersen [51]. Studies reporting CMAP showed 36% of the treatment group as responders compared to 5% of the control group [21]. Egen Klassification (EK2) and Manual Muscle Testing (MMT) studies reported improvements with nusinersen [56] and risdiplam [52], respectively. Bayley Scales of Infant and Toddler Development III (BSID‐III) showed 60% of patients could sit without support for 5 s, and 40% for 30 s [52].

##### 3.3.3.2 Bulbar Function

Of the 16 studies examining type 1 SMA, nine measured bulbar function. In relation to feeding, nine reported this outcome, with four showing some improvement in feeding, one following nusinersen [61], one following nusinersen and onasemnogene abeparvovec [53] and two following risdiplam [52, 54]. Two studies examining nusinersen treatment showed no change [49] or stability [56] and three (all examining nusinersen) showed a decline in the ability to feed orally following treatment [55, 60, 64]. Three of the seven studies examined swallowing, with one showing improved swallowing following risdiplam treatment [54], one showing that the trajectory of decline was less sharp following nusinersen [49] and one showing a decline in swallowing following nusinersen [64] treatment.

##### 3.3.3.3 Frequency or Duration of Hospitalisation

One study reported the frequency of hospitalisation in type 1 patients following treatment with risdiplam. The rate of hospitalisations per person per year was 0.94, and the median duration of hospitalisation was 17 nights [52].

##### 3.3.3.4 Respiratory Function

Three studies examined the effectiveness of nusinersen on respiratory function in Type 1 patients. One study reported improvements in most patients [50], while one reported all patients needing to use respiratory support [55]. A further study reported a reduction in time on ventilatory support in the majority of patients [56].

##### 3.3.3.5 Complications of SMA

One study showed worsening of scoliosis following nusinersen [55], whilst the other showed no association between Cobb angle and number of nusinersen administrations [65]. One study examining growth and weight in patients with type 1 showed worsening following risdiplam. The median change from baseline to 24 months for the weight‐for‐age percentile and length−/height‐for‐age percentile was −4.2 and −17.3, respectively [52].

##### 3.3.3.6 Need for Ventilation

Five studies examined type 1 patients' need for ventilation. In two studies most participants did not need permanent ventilation following treatment: one when patients were treated with nusinersen [21], where fewer patients receiving nusinersen than control patients needed permanent ventilation, and one when patients were treated with risdiplam [52]. Improvements were seen in patients receiving nusinersen [57] where hours on ventilation were reduced. Most patients receiving non‐invasive ventilation remained stable in patients receiving nusinersen [61]. Almost a quarter of patients receiving risdiplam (19%–25%) did not require ventilation support [54].

##### 3.3.3.7 Mortality

Five studies examined mortality or survival in type 1 patients. All studies showed most patients to be alive at follow‐up. Amongst patients receiving nusinersen, 7/68 patients died [60] and 84% of patients survived (vs. 61% in the sham treatment arm) [21]. Amongst patients receiving risdiplam, 93% were alive at follow‐up [52] and 4/21 died [54]. More reduction in death and permanent ventilation was observed in patients receiving risdiplam than in those receiving nusinersen in one study [51].

##### 3.3.3.8 Safety: Adverse Events

Eight studies reported adverse events (AEs) in Type 1 patients. In studies examining nusinersen, there were 96% patients reporting an AE compared to 98% in control [21], 2.4% patients [56], no patients compared to two in control [57], 12 patients [58] and 20.8% patients reported a SAE [61]. In patients receiving risdiplam, 78% patients reported AEs [52] and 202 AEs were reported [54].

Eight studies examined serious AEs (SAEs). Of patients receiving nusinersen, 76% of patients reported SAEs compared to 95% in the sham treatment arm [21], one patient [57] and seven patients [60] died. Of patients receiving risdiplam, 68% of patients reported SAEs [52] and 24 SAEs were reported [54]. Fewer SAEs were reported in patients receiving risdiplam than nusinersen in one study [51].

The most common AEs reported among nusinersen patients were headache [56, 61], fever, vomiting and loss of appetite [57], post lumbar puncture syndrome and respiratory problems [58]. The most common AEs among risdiplam patients were respiratory‐related AEs [52, 54].

##### 3.3.3.9 Health Related Quality of Life (HRQoL)

Only one study examining the effectiveness of risdiplam on type 1 SMA examined HRQoL [52]. The Infant and Toddler Quality of Life Questionnaire (ITQOL‐SF47) was used, and showed improvement in emotional health, temperament and mood, growth and development domains; no change in bodily pain/discomfort, parent impact on time limitation, family cohesion, overall health domains; and a decline in physical abilities and general health perceptions.

#### 3.3.4 Type 1 and 2 SMA

Eight studies [73, 74, 75, 76, 77, 78, 79, 80], reported the effectiveness of type 1 and 2 SMA and two [67, 68], reported Type 2 alone. Many outcomes were supported by very few, or no studies making it difficult to provide conclusive evidence of the effectiveness of nusinersen and risdiplam for Type 1 and 2 SMA. Noting this limitation, studies of Type 1 and 2 SMA showed improved respiratory function but small or non‐significant improvements in bulbar function or need for ventilation. Overall motor function appeared to improve following treatment, but studies were inconsistent. The majority of studies reported a high number of patients with AEs, including some SAEs including death. A summary of outcomes can be found in Table 6.

**TABLE 6:** Summary of Type 1 and 2 outcomes.

Outcome	Nusinersen	Risdiplam
Motor function		
HFMSE	Significant increase in mean scores; more clinically meaningful increases in treated versus untreated patients	Not specified
RULM	Significant increase in scores between baseline and 12 months; more clinically meaningful increases in treated versus untreated patients	Not specified
CHOP INTEND	Improvement in mean total scores from 35.1 to 50.3	Not specified
HINE‐2	Significant improvements in head control, sitting, voluntary grasp, ability to kick supine, rolling, crawling and standing; 78% responders in infantile‐onset versus 0% sham; more sham patients able to stand in later‐onset	Not specified
WHO Milestones	93% gained new milestones; 2 patients lost a motor ability; 1 remained stable	Not specified
CGI‐I	More patients improved compared to those initially in the sham treatment arm	Not specified
MFM	Non‐significant increase in one subscale from 55%–61%; median total score increased by 6 points	Not specified
Bulbar function	Non‐significant increase in number of patients needing feeding support	Not specified
Respiratory function	Better respiratory muscle strength and significantly better FVC in Type 2 patients versus control	Not specified
Need for ventilation	No overall change; non‐significant increase in Type 1 patients; increase in patients with 2 SMN2 copies, decrease in patients with 3 SMN2 copies; longer time on ventilation in sham versus nusinersen	Not specified
Growth	Mean weight, body length, head circumference and chest circumference increased over time	Not specified
Adverse events	95 AEs in 25 patients; most common AE: vomiting (29% nusinersen vs. 14% sham); 6 patients died; common AEs: lumbar puncture procedure, headache	More AEs in Type 2 patients (85% vs. 15% in Type 1); 100 AEs in Type 2 versus 30 AEs in Type 1; 4 SAEs and 10 SAEs reported; common AEs: respiratory, gastrointestinal, skin issues, headache, diarrhoea and pyrexia
HRQoL	No significant difference in family/caregiver HRQoL between treated and untreated patients; fathers of treated children reported more negative impact on productivity and daily activities	Not specified

Abbreviations: AE, adverse event; CGI‐I, Clinical Global Impression–Improvement; CHOP INTEND, Children's Hospital of Philadelphia Infant Test of Neuromuscular Disorders; FVC, forced vital capacity; HFMSE, Hammersmith Functional Motor Scale – Expanded; HINE‐2, Hammersmith Infant Neurological Examination, section 2; HRQoL, health‐related quality of life; MFM, Motor Function Measure; RULM, Revised Upper Limb Module; SAE, serious adverse event; WHO, World Health Organization.

##### 3.3.4.1 Motor Function

Studies on motor function in Type 1 and Type 2 patients following nusinersen treatment show significant improvements. HFMSE and Revised Upper Limb Module (RULM) both reported significant score increases in Type 2 patients [67]. CHOP INTEND scores improved in Type 1 and Type 2 SMA patients, with mean total scores increasing from 35.1 to 50.3 [73]. The Hammersmith Infant Neurological Examination (HINE‐2) showed significant improvements in head control, sitting, voluntary grasp and other motor functions in Type 1 and Type 2 patients [73, 77].

WHO Motor Milestones indicated that 93% of Type 1 and 2 patients gained new milestones following nusinersen treatment [74]. The Clinical Global Impressions of Improvement (CGI‐I) showed more improvements in patients receiving nusinersen compared to those in the sham treatment arm [76]. Motor Function Measurement (MFM) studies reported increases in scores, with one study showing a non‐significant increase in one subscale and another showing a median total score increase of 6 points [73, 77].

##### 3.3.4.2 Bulbar Function

One study examining bulbar function in patients with type 1 and 2 SMA following treatment with nusinersen reported a non‐significant increase in the number of patients needing feeding support [73].

##### 3.3.4.3 Respiratory Function

One study examining respiratory function in patients with Type 1 and 2 following treatment with nusinersen, reported better respiratory muscle strength and significantly better forced vital capacity (FVC) in patients with type 2 SMA compared to control [77].

##### 3.3.4.4 Need for Ventilation

Three studies examined the effectiveness of nusinersen on type 1 and 2 patients' need for ventilation. No change was seen in the number of patients needing ventilation support overall, but there was a non‐significant increase in the number of patients needing ventilation support among patients with type 1 SMA [73]. A further study showed an increase in the number of patients needing ventilation support in patients with 2 SMN2 copies, and a decrease in patients with 3 SMN2 copies [74]. When examining time on ventilation in patients receiving nusinersen, longer time was spent receiving ventilation support in patients receiving sham treatment arm (infantile‐onset: 11.1%, later‐onset: 43.8%) or sham then nusinersen (infantile‐onset: 8.5%, later‐onset: 48.6%) compared to those receiving nusinersen (infantile‐onset: 0%, later‐onset: 17.6%) [76].

##### 3.3.4.5 Complications of SMA

###### 3.3.4.5.1 Growth

One study examined growth in patients with type 1 and 2 following nusinersen treatment. Mean weight, body length, head circumference and chest circumference increased over time for participants in all groups [76].

##### 3.3.4.6 Safety: Adverse Events

Five studies report adverse events in patients with type 1 and 2 SMA. Two examined the effectiveness of nusinersen and reported 95 AEs in 25 patients [73]. A further study reported the most common AE as vomiting, reported by 29% of patients receiving nusinersen and 14% of patients receiving sham following nusinersen [76]. Three studies reported AEs following risdiplam, with more AEs reported among type 2 patients (85% vs. 15% in type 1 [75] and 100 AEs reported in type 2 vs. 30 AEs reported in type 1) [78]. 173 AEs were reported in one study [79]. SAEs are reported in nusinersen with six patients dying [73], four SAEs [75], 10 SAEs [78] and 31 SAEs [79] reported following risdiplam. One further study reported AEs in type 2 patients, with 92% reporting at least one AE, and 14% reporting SAEs [68].

The most common AEs following nusinersen were AEs related to the lumbar puncture procedure and headache [73], and following risdiplam treatment were respiratory, gastrointestinal, skin issues and headache [75, 78], as well as diarrhoea and pyrexia [79].

##### 3.3.4.7 Health Related Quality of Life (HRQoL)

Only one study reported HRQoL in patients with type 1 and 2 SMA following nusinersen. Family/caregiver HRQoL was measured, and there was no significant difference between treated and untreated patients. Fathers of treated children reported a greater negative impact on productivity at work and daily activities than fathers of untreated patients [80].

#### 3.3.5 Type 2 and 3 SMA

Twenty‐eight studies examined the effectiveness of nusinersen or risdiplam in patients with Type 2 and 3 SMA [22, 81, 82, 83, 84, 85, 86, 87, 88, 89, 90, 91, 92, 93, 94, 96, 97, 98, 99, 100, 101, 102, 103, 104, 105, 106, 107, 108, 109, 110]. A summary of outcomes can be found in Table 7.

**TABLE 7:** Summary of Type 2 and 3 outcomes.

Outcome	Nusinersen	Risdiplam
Motor function		
HFMSE	Improvements in 14 studies; no change or stabilisation in 4 studies; significant improvements in Type 3 patients	Increase in scores; 22.6% improved on at least one motor scale; mean increase of 0.95
RULM	Significant increase in some studies; no significant increase in others; improvements in treated versus control	Significant improvements in some studies; no significant improvements in others
CHOP INTEND	Mean increase of 2.37 points	Not specified
6MWT	Clinically meaningful improvements in most patients; some regained ability to walk	No significant increase in Type 3 patients
WHO Milestones	20% gained at least one milestone versus 6% in control; no milestones lost during treatment	Not specified
RHS	Significant increase at 6 months, not beyond	Increase in 2/6 patients; meaningful improvement in 3 patients
ALSFRS‐R	Increase of 1 point in 3 studies	Not specified
MRC‐SS	Increases in Type 3 patients; significant increases in ambulatory patients	Not specified
CMAP	Increase in some studies; no significant changes in others	Not specified
EK2	Improvement by at least 2 points in 5 patients	Not specified
MMT	Significant increase at 6 and 14 months, then no further increase	Not specified
MUNE	Increase in Type 2 patients; decrease in Type 3 patients	No significant differences overall
MFM	Not specified	Improvements in 32% of patients; stabilisation in 58%
GFFS	Increase in all cohorts; increased hand strength in 3 patients	Not specified
Bulbar function	Mixed results: some improved, some worsened; increased need for tube feeding	Similar numbers improved and worsened
Hospitalisation	Rate of 0.1 per patient versus 0.49 in control	Not specified
Respiratory function	No significant improvement in some studies; FVC remained stable	No significant improvement; improvement in 10/15 patients
Scoliosis	Worsening in 12 non‐ambulant patients	Not specified
Weight	Not specified	9/31 patients gained more than 5% body weight
Hip and knee motion	Worsened motion in 1 Type 2 and 1 Type 3 patient	Not specified
Need for ventilation	9% started occasional ventilation; none needed permanent ventilation; one discontinued ventilation	Not specified
Stamina and fatigue	Not specified	Reduction in fatigue after treatment
Adverse events	Majority reported at least one AE; common AEs: post lumbar procedure syndrome, headache, back pain, respiratory and gastrointestinal difficulties; 17%–18% reported SAEs	92.5% reported an AE; common AEs: headache, nausea, back pain; no worsening in QoL
HRQoL	No significant change in PedsQL GCS; improvement in financial burden; decline in burden on time, improvement in patient and caregiver QoL on some scales	No worsening in QoL

Abbreviations: 6MWT, 6‐Minute Walk Test; AE, adverse event; ALSFRS‐R, Amyotrophic Lateral Sclerosis Functional Rating Scale—Revised; CHOP INTEND, Children's Hospital of Philadelphia Infant Test of Neuromuscular Disorders; CMAP, Compound Muscle Action Potential; EK2, Egen Klassifikation 2; FVC, forced vital capacity; GFFS, Global Fatigue and Function Scale; HFMSE, Hammersmith Functional Motor Scale—Expanded; HRQoL, health‐related quality of life; MMT, Manual Muscle Testing; MRC‐SS, Medical Research Council Sum Score; MUNE, Motor Unit Number Estimation; MFM, Motor Function Measure; PedsQL GCS, Paediatric Quality of Life Inventory Generic Core Scales; QoL, quality of life; RHS, Revised Hammersmith Scale; RULM, Revised Upper Limb Module; SAE, serious adverse event; WHO, World Health Organization.

Most outcomes were reported by very low numbers of studies. Type 2 and 3 SMA patients showed improvements or stabilisation of motor function, minimal improvement in respiratory function, worsening of complications of SMA, an increased need for ventilation, and where reported, the majority, or all patients reported at least one AE.

##### 3.3.5.1 Motor Function

Studies on motor function in Type 2 and 3 patients following nusinersen treatment show varied results. HFMSE scores improved in most studies, though some showed no change or stabilisation [82, 94, 102, 103] RULM scores generally increased, with some studies showing more improvement in Type 3 patients [85, 92, 96, 97, 102, 107, 108]. CHOP INTEND scores showed a mean increase [93]. The 6MWT indicated significant improvements in walking distance for many patients [81, 85, 92]. WHO Motor Milestones showed gains in new milestones, with no losses during treatment [22, 97, 109]. RHS scores increased in most studies [86, 108]. Amyotrophic Lateral Sclerosis Functional Rating Scale—Revised (ALSFRS‐R) and Medical Research Council Sum Score (MRC‐SS) scores also showed improvements [81, 86, 98, 102]. CMAP results were mixed, with some studies showing increases and others no significant changes [71, 85, 91, 108].

Other motor function outcomes were measured in individual studies and details can be found in Table S11.

##### 3.3.5.2 Bulbar Function

Five studies reported bulbar function in type 2 and 3 SMA patients. Four studies examined swallowing. In studies examining nusinersen, six patients improved while six worsened and no new swallowing impairments developed [82] and minimal nusinersen patients needed treatment compared to all untreated patients [101]. Two studies examined the effectiveness of risdiplam on swallowing; similar numbers of patients improved and worsened following risdiplam [83, 99]. One study examined feeding following nusinersen treatment with an increased need for tube feeding after treatment [97].

##### 3.3.5.3 Frequency or Duration of Hospitalisation

Two studies examined the rate of hospitalisation in patients receiving nusinersen. One patient needed hospitalisation [81] and the rate of hospitalisation was 0.1 per patient in patients treated with nusinersen compared to 0.49 in the control group [93].

##### 3.3.5.4 Respiratory Function

Six studies examined respiratory function in type 2 and 3 patients. No significant improvement was seen in two studies of nusinersen [86, 92] and one of risdiplam [100, 104, 105]. FVC remained stable [88], and increased [109] following nusinersen treatment and 10/15 patients reported improvement following risdiplam [99].

##### 3.3.5.5 Complications of SMA

Studies on complications of SMA in Type 2 and 3 patients following treatment with nusinersen and risdiplam show varied outcomes. Scoliosis worsened in 12 non‐ambulant patients treated with nusinersen [93]. Weight gain of more than 5% was observed in 9 out of 31 patients treated with risdiplam [99]. Hip and knee motion worsened in one Type 2 and one Type 3 patient receiving nusinersen [89]. The need for ventilation was examined, with 9% of nusinersen‐treated patients starting occasional ventilation, none requiring permanent ventilation and one discontinuing ventilation [97]. Fatigue reduction was noted in patients treated with risdiplam [99].

##### 3.3.5.6 Safety: Adverse Events

Ten studies reported AEs in patients with type 2 and 3 SMA and two studies reported AEs in patients with type 3 only. Among patients with type 2 and 3, eight reported the effectiveness of nusinersen. The majority, or all patients reported at least one AE in three papers (89% [84], 100% [85], 93% vs. 100% in the control group) [22], but low rates of AE were reported in other studies examining the effectiveness of nusinersen (reported in 3/37 patients [86], in 41.4% of patients [92], in 4.2% of patients [93], nine AEs reported [94] and AEs reported in 64/256 [97]). SAEs were reported by two studies, with 17% of patients receiving nusinersen reporting a SAE vs. 29% in the control group [22] and 18% reporting a SAE [85].

In studies examining AEs following nusinersen in type 3 only, there was a 1.3% discontinuation rate because of AEs [72], and no SAEs were reported [70].

One study reported the effectiveness of risdiplam in types 2 and 3. 92.5% of patients reported an AE compared to 91.7% in the placebo group [100, 104, 105].

The most common AEs reported for type 2 and 3 patients were post lumbar procedure syndrome [85], headache in six studies [22, 85, 86, 92, 93, 94], back pain [22, 84, 93] and respiratory and gastrointestinal difficulties [97]. In another study of type 2 and 3 patients [108] most frequently reported AEs were deranged coagulation profile (60%) and raised urine spot protein creatine ratio (33.3%) In one study examining type 3 only, headache, nausea and back pain were most reported [70].

##### 3.3.5.7 Health Related Quality of Life (HRQoL)

HRQoL was reported in five studies of patients with type 2 or 3 SMA. Patients receiving nusinersen showed no significant change in the Paediatric Quality of Life Inventory—Generic Core Scales (PedsQL GCS) [84, 90], or Assessment of Caregiver Experience with Neuromuscular Disease (ACEND) [108] but showed improvement in financial burden and decline on burden on time on ACEND [90]. Improvements were seen for patients and caregivers on Paediatric Quality of Life Inventory—Family Impact Module (PedsQL FIM), Neuromuscular Module (PedsQL NMM) and SMA Independence Scale—Upper Limb Module (SMAIS‐ULM).[107]Patients receiving risdiplam showed no worsening in QoL following treatment [99].

#### 3.3.6 Mixed Type SMA

Fifty studies reported the effectiveness of nusinersen on a mixture of SMA types [81, 95, 111, 112, 113, 116, 117, 118, 119, 120, 121, 123, 125, 126, 127, 128, 130, 131, 132, 133, 134, 135, 136, 139, 140, 141, 142, 145, 146, 148, 149, 150, 151, 152, 153, 154, 156, 157, 158, 160, 161, 162, 163, 164, 165, 167, 168, 170, 171, 173, 174] one reported the effectiveness of risdiplam [122], one reported the effectiveness of nusinersen and onasemnogene abeparvovec [124] and one reported the effectiveness of nusinersen and risdiplam [143]. In studies where patients had a mixture of SMA types, there was generally an improvement in motor function, an increased need for ventilation, a worsening of scoliosis, increased hospitalisation and a worsening of feeding ability. As there was a large range of SMA types reported by studies in this category, results are less clear on the impact of nusinersen and risdiplam by SMA type. A summary of outcomes can be found in Table 8.

**TABLE 8:** Summary of mixed SMA types outcomes.

Outcome	Nusinersen	Risdiplam
Motor function		
HFMSE	Majority of studies showed increases: 6 showed stabilisation, 1 showed mixed results	Not specified
RULM	Mixed results in some, increases in others	Not specified
CHOP INTEND	Varied results in some, increases in others	Not specified
6MWT	Majority showed improvement, some showed no significant increase or worsening	Not specified
HINE‐2	Scores unchanged in some, increased in others	Not specified
WHO Milestones	Significant increases, stable or higher milestones	Not specified
RHS	1 study: no significant increase in 12/17 Type 3 patients	Not specified
CGI‐I	Majority showed improvement	Not specified
ALSFRS‐R	Majority showed improvement	Not specified
MRC‐SS	1 study: significant increase by 2.4–2.5 points	Not specified
CMAP	Increases observed	Not specified
EK2	1 study: significant increase	Not specified
TUG	1 study: faster scores over time	Not specified
MUNE	1 study: increased scores	Not specified
GFFS	1 study: significant increase in scores	Not specified
Bulbar function	General worsening, more needing tube feeding	Not specified
Hospitalisation	Slight increase in rate and duration	Not specified
Respiratory function	Improvements in some, no change or decline in others	Not specified
Scoliosis	Worsening observed	Not specified
Weight	1 study: higher BMI in patients weaning off nusinersen	Not specified
Need for ventilation	Increase in need for support, some stopped ventilation	Not specified
Stamina and fatigue	Mixed results, increased and improved fatigue	Not specified
Mortality	Median survival 823 days, 58% alive at end	Not specified
Adverse events	19 studies reported AEs: varied rates, common AEs: headache, back pain, lumbar puncture site pain, post lumbar puncture syndrome	Not specified
HRQoL	Mixed results, some showed no significant difference, some showed improvements, one showed decline	1 study: reported HRQoL following both treatments

Abbreviations: 6MWT, 6‐Minute Walk Test; AE, adverse event; ALSFRS‐R, Amyotrophic Lateral Sclerosis Functional Rating Scale – Revised; CGI‐I, Clinical Global Impression–Improvement; CHOP INTEND, Children's Hospital of Philadelphia Infant Test of Neuromuscular Disorders; CMAP, Compound Muscle Action Potential; EK2, Egen Klassifikation 2; GFFS, Global Fatigue and Function Scale; HFMSE, Hammersmith Functional Motor Scale—Expanded; HINE‐2, Hammersmith Infant Neurological Examination, section 2; HRQoL, health‐related quality of life; MMT, Manual Muscle Testing; MRC‐SS, Medical Research Council Sum Score; MUNE, Motor Unit Number Estimation; RHS, Revised Hammersmith Scale; RULM, Revised Upper Limb Module; TUG, Timed Up and Go test; WHO, World Health Organization.

##### 3.3.6.1 Motor Function

Studies on motor function in mixed SMA types following nusinersen treatment show varied results. HFMSE scores generally increased, though some studies showed stabilisation or both improvements and declines [116, 125, 133, 139, 152, 156, 167]. RULM scores mostly improved, with some studies showing stabilisation or mixed results [156, 167, 170]. CHOP INTEND scores increased, especially with higher doses of nusinersen, though some studies had varied results [139, 152, 162]. The 6MWT showed improvements in most studies, with some reporting no significant increase or mixed results [117, 125, 149]. HINE‐2 scores generally increased, with some stabilisation in later years [123, 133, 136, 152, 157, 161, 164, 165, 174]. WHO Motor Milestones showed significant increases and stability in all patients [112, 127, 131, 133]. RHS, CGI‐I, ALSFRS‐R and MRC‐SS scores also improved in most studies [117, 125, 164, 165, 168]. CMAP scores increased in all studies, with higher scores in patients with more SMN2 copies [113, 116, 139, 157, 170]. EK2, TUG, MUNE and GFFS scores also showed improvements in individual studies [125, 128, 139, 168].

##### 3.3.6.2 Bulbar Function

Bulbar function was reported in six studies of nusinersen, indicating a general worsening in relation to feeding, with more patients needing tube feeding or more support following nusinersen treatment [117, 126, 161, 167], increases in bulbar functioning in 48.8% of patients [157] and mixed results with type 1c patients retaining the ability to feed while type 1b lost the ability to feed [131]. Bulbar function was reported in one study of mixed type following nusinersen and onasemnogene abeparvovec[124]showing no significant differences after 1 year of treatment.

##### 3.3.6.3 Frequency or Duration of Hospitalisation

Two studies reported hospitalisation following nusinersen treatment. There was a slight increase in the rate of hospitalisation [81, 133] and an increase in duration of hospitalisation from 2.88 days pre‐treatment to 11.23 days post‐treatment [81].

##### 3.3.6.4 Respiratory Function

Eleven studies reported respiratory function following nusinersen treatment. Five studies reported improvements in FVC and general respiratory function [125, 128, 131, 133, 168], five reported no change following nusinersen treatment [118, 120, 156, 157, 170] and one reported a decline in one patient following nusinersen treatment [117]. One study reported respiratory function in patients with mixed SMA type, showing no change following risdiplam treatment [122].

##### 3.3.6.5 Complications of SMA

Studies on complications of SMA following nusinersen treatment show varied outcomes. Scoliosis worsened in all examined cases [131, 136, 154, 157].

One study reported improvements in height‐for‐age measures [158]. Weight outcomes were mixed, with one study noting higher BMI in patients weaning off nusinersen [153] and another reporting a decrease in BMI [158]. The need for ventilation generally increased, though some patients were able to stop ventilation support [126, 133, 142, 154, 161, 165, 167]. Fatigue outcomes were also mixed, with one study reporting increased fatigue [118] and another noting improvements [156].

##### 3.3.6.6 Mortality

Two studies reported survival/time to death following nusinersen treatment. Median survival was 823 days, with 10 patients dying during the study period [142], and median time to death was 73.0 weeks in patients receiving nusinersen compared to 22.6 weeks in patients receiving sham or sham then nusinersen. 58% of patients alive at baseline, were alive at the end of the trial [127].

##### 3.3.6.7 Safety: Adverse Events

Twenty‐five studies of mixed SMA types reported AEs during or following nusinersen treatment. Of these, the number of AEs reported varied, but some reported high rates of AEs 91% patients [134], 77% patients [168], 62.9% patients [174], whilst others reported more moderate or low rates [81, 112, 117, 125, 128, 131, 132, 135, 140, 145, 149, 156, 157, 161, 164, 165, 167, 170]. Eight studies report the presence of SAEs, although these are generally low incidents [117, 131, 132, 141, 154, 164, 165, 168].

The most common AEs reported in studies examining the effectiveness of nusinersen in studies with mixed SMA types were headache [81, 125, 128, 134, 135, 140, 145, 149, 157, 161, 164, 165, 170], back pain [125, 134, 135, 145, 149, 157, 160, 161, 168], lumbar puncture site pain [81, 128, 145, 170] and post lumbar puncture syndrome [134, 156, 168]. One study examining mixed SMA type following risdiplam reported AEs [122], with most common ones being pyrexia (24%), respiratory tract infection (21%) and headache (18%).

##### 3.3.6.8 Health Related Quality of Life HRQoL

Eleven studies reported HRQoL following nusinersen. Of these, four showed no significant difference as a result of nusinersen treatment on HRQoL [95, 111, 125, 153, 171], three showed improvements compared to before nusinersen or to the control group [119, 126, 148, 150, 173] and one study showed a change of −4.8 points on the SF36 scale [118].

One study reported HRQoL following nusinersen and risdiplam [143].

#### 3.3.7 Unreported SMA Type

The review findings included 14 studies which did not report SMA Type [114, 115, 129, 137, 138, 144, 147, 155, 159, 166, 169, 172, 175, 176]. There was a worsening of feeding ability and an increased need for ventilation. Motor function generally increased, but 6MWT and TUG were performed better in control patients. AEs and SAEs were reported. These results are limited in their contribution within this review, as it is not clear, or not reported, which SMA types the outcomes are referring to.

## 4 Discussion

### 4.1 Summary of Methodology and Key Findings

This systematic review aimed to examine the safety and effectiveness of nusinersen, and risdiplam for the treatment of all types of SMA (types 0, 1, 2, 3, 4) and presymptomatic patients. Comprehensive searches resulted in a synthesis of 131 included studies.

The review found that while motor function improvements were largely consistent across treatments and SMA types, other health outcomes such as bulbar function, respiratory function and the need for ventilation showed mixed results. Adverse events were common across all treatments and SMA types, with some serious cases reported, for example deaths reported following risdipam and nusinersen treatment in type 1 and following nusinersen treatment in type 1 and 2 patients, although it remains unclear whether these deaths were due to the drug or the progression of the disease.

There was limited evidence on the effectiveness of treatment in presymptomatic patients. Both nusinersen and risdiplam showed improvements in motor function and stabilised bulbar function. Some presymptomatic studies showed 100% survival during the study period following both nusinersen and risdiplam, and improvement in ventilation following nusinersen. AEs were reported for all interventions, with some being attributed to treatment, and others being considered as not treatment‐related.

Nusinersen and risdiplam treatments significantly improved or stabilised motor function (in most measures reported) in type 1 patients, and studies combining nusinersen with other treatments also reported positive outcomes. The HINE‐2 test indicated stability in most patients, with some achieving new milestones. Higher doses of risdiplam yielded better results in motor function outcomes, and the WHO Motor Milestones test showed more milestones gained with risdiplam than nusinersen. Bulbar function and survival generally improved in type 1 patients, whilst results on respiratory function and the need for ventilation were mixed following nusinersen and risdiplam. Interestingly, there were generally fewer AEs reported in patients receiving treatment compared to control conditions. This observation suggests that treatment‐emergent events may have been related to the progression of the disease rather than the treatment itself, further highlighting the benefits of the treatment.

For patients with type 1 and type 2 SMA, nusinersen treatment significantly improved motor function. Studies show increased scores and new motor milestones, with notable improvements in head control, sitting and voluntary grasp. More patients treated with nusinersen showed overall improvement in reported outcomes compared to those in sham treatment arms.

Improvements were seen in respiratory function and growth, but there were limited numbers of studies examining these outcomes in Type 1 and 2 SMA. There was no change in the need for ventilation or bulbar function among these patients. The majority of studies reported high numbers of patients with AEs, including some SAEs including death following nusinersen treatment, which may be related to either the progression of the disease or the treatment itself.

Studies on motor function in Type 2 and 3 patients following nusinersen treatment show varied results. Most studies reported improvements in HFMSE and RULM scores, though some showed no change or stabilisation. CHOP INTEND and 6MWT scores generally improved, indicating better motor function and walking distance. WHO Motor Milestones and other motor function measures showed gains during treatment. CMAP results were mixed, with some studies showing increases and others no significant changes.

Nusinersen and risdiplam treatments for type 2 and 3 SMA patients show varied outcomes in managing complications. Scoliosis and hip/knee motion worsened in some patients, while others experienced weight gain and reduced fatigue. Ventilation needs were slightly increased, with occasional use in a few cases and no permanent ventilation required. Where reported, the majority or all patients reported at least one AE.

Studies examining mixed SMA types following nusinersen treatment show varied results in motor function, but overall improvements were generally seen. Worsening was seen in results of SMA complications, with varied results in height, weight and fatigue. Scoliosis worsened in all cases, while ventilation generally improved. The varied results in motor function and SMA complications following nusinersen treatment may be influenced by the mixed severity of the patient group. Patients with later or more severe phenotypes at the time of treatment are less likely to see significant benefits, as the progression of the disease may have already caused substantial impairment. This heterogeneity in patient severity could potentially dilute the overall observed treatment effects, making it challenging to draw definitive conclusions about the efficacy of nusinersen across different SMA types and stages.

In relation to study quality and/or risk of bias, overall, most included RCTs had ‘some concerns’, most non‐randomised studies had ‘serious’ risk of bias and most single arm studies were rated as ‘high quality’. Bias in non‐randomised studies is likely due to the lack of blinding in non‐randomised studies (although it is acknowledged that this might partly be due in some cases to blinding not being reported rather than not completed).

### 4.2 Comparison to Previous Literature

Findings of this review are in line with previous literature. Previous studies show improvement in motor function and ventilation in patients with type 1 SMA following nusinersen [28], improvements in motor function in patients with type 1 SMA following risdiplam [32], improvements in motor function in patients with type 2 and 3 SMA [30] and improvements in motor function in SMA patients following either nusinersen or risdiplam [33].

Recent systematic review evidence of Asian patients with type 2–4 SMA showed findings consistent with that of the current review, with significant improvements seen in motor function measures and motor milestone responses [39], however this previous review only included studies of nusinersen, leaving a gap in knowledge of the effectiveness of risdiplam.

### 4.3 Implications for Policy and Practice

This systematic review highlights the need for high‐quality RCTs that examine the effectiveness and safety of nusinersen and risdiplam, in all SMA types, including UK‐based studies to ensure generalisability to UK treatment pathways. It is acknowledged however.

That conducting clinical trials in this population is extremely challenging and expensive, and randomisation is difficult with an intrathecal product (although it was achieved in the ENDEAR trial with a sham procedure) [21]. Additionally placebo trials for a severe disease such as SMA have raised ethical questions. Current evidence is limited to single or limited numbers of SMA types, often examining different outcomes. A more standardised approach to treating and measuring SMA types and outcomes will ensure a comprehensive evaluation of the effectiveness of treatments. There is also limited RCT evidence for all SMA types, with much evidence relying on single‐arm studies and the methodological limitations surrounding lack of randomisation and blinding that are often associated with this type of study [21, 179]. Nevertheless, single‐arm studies may still hold significant value, particularly for universally degenerative and lethal conditions like SMA, where natural history controls can reveal substantial deviations if the treatment is effective. Additionally, it is crucial to note that the pivotal nusinersen trial (ENDEAR), initially an RCT with a sham treatment arm, was terminated early due to ethical concerns when it became clear that treated children were diverging from the typical SMA progression.

There is currently insufficient evidence to make any conclusions about the relative benefits or effectiveness of nusinersen versus risdiplam, and this was not the intention of this review. However, future research would benefit from examining the acceptability of administration techniques, as nusinersen and risdiplam vary greatly in how they are administered (spinal injection vs. oral administration) and this will therefore be an important consideration.

This research addresses limitations in previous systematic reviews on the topic, providing a comprehensive and thorough review of evidence on all types of SMA, and measuring all available outcomes. This review demonstrates that there is relatively limited evidence available on presymptomatic, type 0 and type 4 SMA, which may impact decision making and treatments for these patient populations. Although the studies NURTURE and RAINBOWFISH provide strong evidence for transforming outcomes; hence, the majority of countries now incorporate newborn screening, data collection through registries will be crucial for continuing information on the presymptomatically treated group. The lack of type 0 patients in studies of SMA treatment is likely due to the circumstances surrounding babies born with type 0 rarely surviving beyond birth [180] or the first month of life [181]. Type 4 SMA is the mildest type of SMA, with less than 5% of all SMA cases being attributed to type 4 [182]. It is possible that the perceived need for research in this population is reduced, given the severity of symptoms and impact of types 1–3.

The findings revealed limited evidence on the clinical effectiveness of risdiplam, potentially attributable to its more recent introduction into clinical treatment pathways for SMA (2020 available in US [19] and Canada [183]) compared to 2016 for nusinersen (FDA approval granted in US [13]).

## 5 Strengths and Limitations

This is the first review to examine all SMA types and treatment options. The strength of the review comes from the methodological rigour applied by dual screening, data extraction and accuracy checking of both the search strategy and quality assessments.

This review addresses limitations with previous systematic reviews. This systematic review includes both nusinersen and risdiplam, the most recently approved treatments for Spinal Muscular Atrophy (SMA), while some other systematic reviews have only focused on one treatment. Recently published, Zhao et al. [39] conducted a systematic review of real‐world studies from mainly East Asian countries (except one from Israel), focusing on SMA types 2–4 and publications from 2017 to 2024. The review assessed motor function outcomes (RULM, HFMSE, 6MWT) and safety, using the Newcastle–Ottawa Scale for bias assessment. It excluded clinical trials and did not evaluate risdiplam efficacy, as only two Asian safety studies on risdiplam were included.

In contrast, our study applied no publication year limits and included both real‐world evidence and clinical trials to ensure a comprehensive evaluation of the relatively new therapies, nusinersen and risdiplam. We adopted a global scope, used Cochrane's risk of bias tools, and included all SMA types (presymptomatic, types 0–4) and all relevant outcomes. This broader, more rigourous approach minimises bias and enhances the reliability of our findings for clinical practice.

The review considers an extensive and comprehensive list of outcomes, stratified by SMA types, to provide a reliable source for clinical practice. This stratification is based on NHS practice and includes an extensive list of outcomes: motor function tools, bulbar function, hospitalisation, respiratory function, SMA complications, the need for non‐invasive or invasive ventilation, stamina and fatigue, mortality, adverse effects of treatment and health‐related quality of life (HRQoL) for both patients and carers.

This review aims to report all evidence, on all eligible study designs since the introduction of nusinersen and risdiplam to clinical practice up to January 2025. Notably, no such comprehensive evidence synthesis has been conducted to date.

Based on these considerations, an unmet need was identified to review and compile an updated, comprehensive report that can be effectively utilised in clinical practice. This review bridges the gap in current evidence and provides a robust resource for healthcare professionals.

Overall, there was significant heterogeneity within results, outcome measures and study designs making meta‐analysis of data non‐viable. The methodological quality of the majority of included studies was poor. While most included studies were single‐arm designs, which inherently limit comparative evidence for different interventions, they still provide valuable insights, particularly in the context of universally degenerative conditions like SMA. The decision to exclude conference abstracts was made for pragmatic reasons due to the vast number of results eligible for inclusion in the review. However, excluding conference abstracts increases the risk of excluding recent research that may not have been published, particularly on newer treatments such as risdiplam. Notwithstanding, the search was updated in January 2025 to ensure all recent publications were included. Searches were limited to studies published in the English language.

A further limitation of the review and the findings was the inconsistent reporting of outcomes in the included literature. Some outcomes were not reported as often as others (e.g., mortality, SMA complications, Stamina and Fatigue), making it difficult to make any overarching conclusions about the effectiveness of treatment in relation to these outcomes.

Many AEs were identified in included studies, following both nusinersen and risdiplam. However, it is important to consider other potential factors, such as disease progression or patient characteristics, that may contribute to severe events.

Very few studies were based in the UK (*n* = 3), with no studies recruiting solely UK patients. This creates some uncertainty around the generalisability of the review findings, to UK patients given the differences in treatment regimes and healthcare services provided to patients and their families and carers. Some country‐specific variations in care and treatment of SMA patients have been identified from global studies of SMA registry evidence. For example, variations between countries have been observed in relation to ventilation, feeding tube use and scoliosis surgery rates [184]. These differences in guidelines for care in SMA patients have been attributed to variations in healthcare systems (nationalised vs. private healthcare), availability of specialist care and social and cultural attitudes to life‐limiting conditions [184].

## 6 Conclusions

This study examined the clinical effectiveness and safety of nusinersen and risdiplam in treating SMA. Comprehensive searches of databases and relevant resources, and thorough screening of titles, abstracts and full texts led to the inclusion of 131 studies reported in 148 sources.

This review is the largest and most comprehensive review including the effectiveness of nusinersen and risdiplam on all SMA Types. There is inconsistency in the outcomes measured and tools used, both within and between SMA Types. Some SMA Types include only a small number of studies, and many included studies are single‐arm studies; despite having natural history comparators, this leads to uncertainty in the results.

## Author Contributions

A.G., J.Pa. and P.A. conceived the study concept and design. J.Pa., A.M., J.Par., M.P., F.B. and J.D. were involved in data acquisition, analysis and interpretation. J.Pa. and A.M. drafted the manuscript. J.Pa., A.M., P.A., A.G., A.B., J.Par., M.P., F.B., J.D. and M.Y. were involved in the critical revision of the manuscript for important intellectual content. A.G. acquired funding. A.B. provided study resources. J.Pa. was the project supervisor. All authors read and approved the final manuscript.

## Funding

This award was funded by the National Institute for Health and Care Research (NIHR) Evidence Synthesis programme (NIHR award ref.: NIHR131964), led by AG. See the NIHR Funding and Awards website for further award information. Evidence Synthesis|NIHR. The funders did not play any role in the study design, data collection and analysis, decision to publish or preparation of the manuscript.

## Ethics Statement

The authors have nothing to report.

## Consent

The authors have nothing to report.

## Conflicts of Interest

The authors declare no conflicts of interest.

## Supporting information


**Data S1:** acn370274‐sup‐0001‐supinfo.docx.

## Data Availability

Data for this systematic review manuscript is derived from published studies.
