# Supplementary material for: Effectiveness and Safety of Nusinersen and Risdiplam in Spinal Muscular Atrophy: A Systematic Review
Source: Ann Clin Transl Neurol. 2025 Dec 5;13(2):218–41. doi: 10.1002/acn3.70274 (PMC12883690; doi:10.1002/acn3.70274)
Supplement: Supplementary file 1 — Data S1: acn370274‐sup‐0001‐supinfo.docx. [file ACN3-13-218-s001.docx]

The clinical effectiveness and safety of nusinersen and risdiplam in Spinal Muscular Atrophy (SMA): A systematic review

Amin Mehrabian^1^, Peter Auguste^1^, Amy Grove^1^, Anna Brown^1^, Janette Parr^1^, Mubarak Patel^1^, Furqan Butt^1^, Jeremiah Donoghue^1^, Mehdi Yousefi^1^, Jo Parsons^1^*

^1^Centre for Evidence and Implementation Science, University of Birmingham, Edgbaston Park Road, Birmingham, UK

*Corresponding author [j.e.parsons@bham.ac.uk](mailto:j.e.parsons@bham.ac.uk) (ORCID ID: 0000-0002-6542-8492)

# Supplementary Table S1: PROSPERO Registered Protocol

| **Section and Topic** | **Item #** | **Checklist item** | **Location where item is reported** |
| --- | --- | --- | --- |
| **TITLE** | | |  |
| Title | 1 | Identify the report as a systematic review. | P1 |
| **ABSTRACT** | | |  |
| Abstract | 2 | See the PRISMA 2020 for Abstracts checklist. | P2 |
| **INTRODUCTION** | | |  |
| Rationale | 3 | Describe the rationale for the review in the context of existing knowledge. | P2-4 |
| Objectives | 4 | Provide an explicit statement of the objective(s) or question(s) the review addresses. | P4 |
| **METHODS** | | |  |
| Eligibility criteria | 5 | Specify the inclusion and exclusion criteria for the review and how studies were grouped for the syntheses. | P5-7 & Table 1 |
| Information sources | 6 | Specify all databases, registers, websites, organisations, reference lists and other sources searched or consulted to identify studies. Specify the date when each source was last searched or consulted. | P5 |
| Search strategy | 7 | Present the full search strategies for all databases, registers and websites, including any filters and limits used. | P5 & Appendix 1 |
| Selection process | 8 | Specify the methods used to decide whether a study met the inclusion criteria of the review, including how many reviewers screened each record and each report retrieved, whether they worked independently, and if applicable, details of automation tools used in the process. | P5-6 |
| Data collection process | 9 | Specify the methods used to collect data from reports, including how many reviewers collected data from each report, whether they worked independently, any processes for obtaining or confirming data from study investigators, and if applicable, details of automation tools used in the process. | P7 |
| Data items | 10a | List and define all outcomes for which data were sought. Specify whether all results that were compatible with each outcome domain in each study were sought (e.g. for all measures, time points, analyses), and if not, the methods used to decide which results to collect. | P7 |
|  | 10b | List and define all other variables for which data were sought (e.g. participant and intervention characteristics, funding sources). Describe any assumptions made about any missing or unclear information. | P7 |
| Study risk of bias assessment | 11 | Specify the methods used to assess risk of bias in the included studies, including details of the tool(s) used, how many reviewers assessed each study and whether they worked independently, and if applicable, details of automation tools used in the process. | P8 |
| Effect measures | 12 | Specify for each outcome the effect measure(s) (e.g. risk ratio, mean difference) used in the synthesis or presentation of results. | P8 |
| Synthesis methods | 13a | Describe the processes used to decide which studies were eligible for each synthesis (e.g. tabulating the study intervention characteristics and comparing against the planned groups for each synthesis (item #5)). | P8 |
|  | 13b | Describe any methods required to prepare the data for presentation or synthesis, such as handling of missing summary statistics, or data conversions. | n/a |
|  | 13c | Describe any methods used to tabulate or visually display results of individual studies and syntheses. | P8 |
|  | 13d | Describe any methods used to synthesize results and provide a rationale for the choice(s). If meta-analysis was performed, describe the model(s), method(s) to identify the presence and extent of statistical heterogeneity, and software package(s) used. | P8 |
|  | 13e | Describe any methods used to explore possible causes of heterogeneity among study results (e.g. subgroup analysis, meta-regression). | P8 |
|  | 13f | Describe any sensitivity analyses conducted to assess robustness of the synthesized results. | n/a |
| Reporting bias assessment | 14 | Describe any methods used to assess risk of bias due to missing results in a synthesis (arising from reporting biases). | P12 |
| Certainty assessment | 15 | Describe any methods used to assess certainty (or confidence) in the body of evidence for an outcome. | n/a |
| **RESULTS** | | |  |
| Study selection | 16a | Describe the results of the search and selection process, from the number of records identified in the search to the number of studies included in the review, ideally using a flow diagram. | P8 |
|  | 16b | Cite studies that might appear to meet the inclusion criteria, but which were excluded, and explain why they were excluded. | n/a |
| Study characteristics | 17 | Cite each included study and present its characteristics. | P10-12 & Appendix 3 |
| Risk of bias in studies | 18 | Present assessments of risk of bias for each included study. | P12 & Supplemental Table 1,2 & 3 |
| Results of individual studies | 19 | For all outcomes, present, for each study: (a) summary statistics for each group (where appropriate) and (b) an effect estimate and its precision (e.g. confidence/credible interval), ideally using structured tables or plots. | P13- 34 & Appendix 4,5 & 6 |
| Results of syntheses | 20a | For each synthesis, briefly summarise the characteristics and risk of bias among contributing studies. | P13- 34 & Appendix 4,5 & 6 |
|  | 20b | Present results of all statistical syntheses conducted. If meta-analysis was done, present for each the summary estimate and its precision (e.g. confidence/credible interval) and measures of statistical heterogeneity. If comparing groups, describe the direction of the effect. | n/a |
|  | 20c | Present results of all investigations of possible causes of heterogeneity among study results. | n/a |
|  | 20d | Present results of all sensitivity analyses conducted to assess the robustness of the synthesized results. | n/a |
| Reporting biases | 21 | Present assessments of risk of bias due to missing results (arising from reporting biases) for each synthesis assessed. | n/a |
| Certainty of evidence | 22 | Present assessments of certainty (or confidence) in the body of evidence for each outcome assessed. | n/a |
| **DISCUSSION** | | |  |
| Discussion | 23a | Provide a general interpretation of the results in the context of other evidence. | P34- 37 |
|  | 23b | Discuss any limitations of the evidence included in the review. | P39- 40 |
|  | 23c | Discuss any limitations of the review processes used. | P39- 40 |
|  | 23d | Discuss implications of the results for practice, policy, and future research. | P37- 38 |
| **OTHER INFORMATION** | | |  |
| Registration and protocol | 24a | Provide registration information for the review, including register name and registration number, or state that the review was not registered. | P4 |
|  | 24b | Indicate where the review protocol can be accessed, or state that a protocol was not prepared. | P4 |
|  | 24c | Describe and explain any amendments to information provided at registration or in the protocol. | n/a |
| Support | 25 | Describe sources of financial or non-financial support for the review, and the role of the funders or sponsors in the review. | P64 |
| Competing interests | 26 | Declare any competing interests of review authors. | P65 |
| Availability of data, code and other materials | 27 | Report which of the following are publicly available and where they can be found: template data collection forms; data extracted from included studies; data used for all analyses; analytic code; any other materials used in the review. | On submission platform |

*From:*  Page MJ, McKenzie JE, Bossuyt PM, Boutron I, Hoffmann TC, Mulrow CD, et al. The PRISMA 2020 statement: an updated guideline for reporting systematic reviews. BMJ 2021;372:n71. doi: 10.1136/bmj.n71. This work is licensed under CC BY 4.0. To view a copy of this license, visit <https://creativecommons.org/licenses/by/4.0/>

# Supplementary S2: PROSPERO registered Protocol


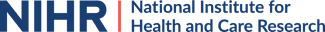


**PROSPERO**

International prospective register of systematic reviews

**Treatment of spinal muscular atrophy: systematic literature review of clinical and cost-effectiveness evidence**

*Peter Auguste, Jo Parsons, Mehdi Yousefi, Mubarak Patel, Anna Brown, Amin Mehrabian, Janette Parr, Amy Grove*

**Citation**

Peter Auguste, Jo Parsons, Mehdi Yousefi, Mubarak Patel, Anna Brown, Amin Mehrabian, Janette Parr, Amy Grove. Treatment of spinal muscular atrophy: systematic literature review of clinical and cost-effectiveness evidence. PROSPERO 2024 Available from <https://www.crd.york.ac.uk/PROSPERO/view/CRD42024512226>

REVIEW TITLE AND BASIC DETAILS

**Review title**

Treatment of spinal muscular atrophy: systematic literature review of clinical and cost-effectiveness evidence

**Review objectives**

To undertake a systematic review and meta-analysis of evidence to understand the clinical and cost-effectiveness of treatments for spinal muscular atrophy.

**Keywords**

Economic evaluation, Nusinersen, Onasemnogene aberparvovec, Risdiplam, Spinal muscular atrophy, Systematic review

SEARCHING AND SCREENING

**Searches**

Searches will be conducted in a range of sources, including: Embase (Ovid); MEDLINE All (Ovid); Cochrane CENTRAL (the Cochrane Central Register of Controlled Trials (CENTRAL, Wiley); International HTA database (INAHTA); Science Citation Index and Conference Proceedings (Web of Science), ClinicalTrials.gov, WHO International Clinical Trials Registry Platform and websites of selected international HTA and medicines approval agencies (NICE, Scottish Medicines Consortium, Canadian Agency for Drugs and Technologies in Health, Institute for Clinical and Economic Review.
Clinical searches were undertaken on 29 January 2024 and cost-effectiveness searches on 30 January 2024
Restrictions: only full publications in the English Language will be considered

**Study design**

Clinical effectiveness: Randomised controlled trials (RCTs), and non-randomised trials, observational studies, case reports
Cost-effectiveness: Full economic evaluations (e.g., cost-effectiveness, cost utility, cost benefit, cost consequence, or cost minimisation analyses)

ELIGIBILITY CRITERIA

**Condition or domain being studied**

Spinal muscular atrophy (SMA) is an uncommon condition that causes muscle weakness. Over time SMA leads to problems with the function of the spinal cord and loss of movement in the body. SMA is usually caused by faults in the persons genes and affects the motor neurones. These are the cells in the brain that allow people to crawl and move. Motor neurones also support movement of the arm, head and neck, and include swallowing and breathing. Across the world SMA affects one to two people in every 10, 000 people. Most recent information from the UK suggests that approximately 70 people were born with SMA in 2021. Type 1 SMA can develop between 2 to 6 months of age, but people can be diagnosed later in life (Types 2, 3 and 4 SMA). SMA causes much disability and can result in death. SMA affects families and carers, including the impact of caring for the patient. There is often a need for specialist equipment and ongoing emotional, financial, and social support for people with SMA.

**Population**

People with 5q spinal muscular atrophy (SMA)
People with types 0, 1, 2, 3, 4 5q SMA

**Intervention(s) or exposure(s)**

Nusinersen monotherapy; Risdiplam monotherapy

**Comparator(s) or control(s)**

Established clinical management;
Best supportive care;
The interventions will be compared to each other.

In addition, for children aged 12 months and under with a bi-allelic mutation in the SMN1 gene and present with a clinical diagnosis of type 1 5q SMA or pre-symptomatically with up to 3 copies of the SMN2 gene.
Onasemnogene abeparvovec

OUTCOMES TO BE ANALYSED

**Main outcomes**

• Motor function (including, where applicable, both age-appropriate gross motor milestones and fine motor skills)
• Bulbar function (including, for example, swallowing and ability to communicate)
• Frequency and duration of hospitalisation
• Respiratory function
• Complications of spinal muscular atrophy (including, for example, scoliosis and muscle contractures)
• Need for non-invasive or invasive ventilation.
• Stamina and fatigue
• Mortality
• Adverse effects of treatment
• health-related quality of life (for patients and carers).
Economic
• Cost per life-years gained (LYG) or cost per quality adjusted life years (QALYs)

**Additional outcomes**

None

DATA COLLECTION PROCESS

**Data extraction (selection and coding)**

For all included studies, the relevant data will be extracted independently by a single reviewer using a standardised data extraction form informed by the NHS Centre for Reviews and Dissemination (CRD). Extracted data will be validated by a second reviewer and discrepancies will be resolved by discussion, with the involvement of a third reviewer when necessary. Where studies do not report summary statistics (e.g., mean score, standard deviation, standard error), we will attempt to calculate these parameters if individual participant data or related effect size-level statistics are provided. Where people with several types of 5q SMA are treated in one trial, we will extract information for relevant subtypes. The extracted data will be entered into summary evidence tables. The extracted information will include: study characteristics, patient baseline characteristics, treatment characteristics, and outcome characteristics for each included outcome reported. Measures of variability and statistical tests used will also be extracted (standard deviation, 95% CI, standard error, p-values).
For the cost-effectiveness, information will be extracted from the relevant studies using an a priori pre-piloted data extraction sheet based on items outlined by CHEERS 2022 and Wijnen et al. Relevant information will be extracted on study details, characteristics, treatment strategies, analytical methods, results, discussion, and other. If data are missing or has not been clearly reported efforts will be made to contact the corresponding author. A request for the missing information will be sent, allowing authors a two-week period to respond. If no response is received within this timeframe, it will be assumed that the requested data are not available. Data extraction will be undertaken by one reviewer, then cross-checked by a second reviewer for accuracy. Any disagreements between the reviewers will be resolved by discussion or by recourse to a third reviewer.

**Risk of bias (quality) assessment**

Appraisal will be undertaken by two reviewers. Uncertainty and/or any disagreements will be crosschecked with a second reviewer and will be resolved by discussion. All primary studies will be appraised using the Cochrane risk of bias assessment tool. A quality assessment will not be performed for single-arm non-randomised studies, which will be assumed to be at high risk of bias if they are used to inform relative treatment effects.
All published economic evaluations included in this systematic review, along with any economic evaluations submitted to NICE by companies, will undergo a comprehensive appraisal. The reporting and methodological quality of each economic evaluation will be assessed using the consolidated health economic evaluation reporting standard (CHEERS) and appraised against the Philips’ checklists, respectively. The CHEERS checklist emphasises the study's relevance to policy and practice, as well as its transparency and reporting of results. The risk of bias/methodological quality will be assessed using the Philips’ checklist, which comprises 57 items under two domains structure and data. Each economic analysis will be assessed by one reviewer and cross-checked by a second health economist. Any disagreements between the reviewers will be resolved by discussion or by recourse to a third reviewer.

PLANNED DATA SYNTHESIS

**Strategy for data synthesis**

Narrative synthesis
Data extracted from identified studies will be organised into tables which comprise essential information on the clinical aspects of the studies. These tables will include detailed design of the studies, interventions and comparators, population characteristics, and key outcome measures. These will be presented as a comparison between the two (or more) treatment groups. A narrative synthesis will accompany each table, providing a comprehensive overview and critique of each study with special emphasis on the methodological quality and population diversity of the studies.
If clinically appropriate, a naïve comparison of the treatments will be performed, with the limitations of the analysis clearly described. Depending on the available data, more advanced comparison might be feasible.
A propensity score-based comparison may be feasible (e.g., propensity score weighting (PSW) or matching-adjusted indirect comparison (MAIC)) if managed access agreement or trial data are shared. If so, key variables for matching across populations will be identified through consultation with clinical experts. Estimated weightings will be assessed for suitability and the relative treatment effect estimated for the most relevant population.
Alternatively, network meta-analyses (NMA) may be most appropriate. If suitable data are available to perform a pair-wise meta-analysis between nusinersen and risdiplam, this will be done to test the effectiveness between the two treatments.
A thorough assessment of the quantity and quality of available data for each outcome will be conducted, also considering different subgroups such as number of SMN2 gene copies in people with pre-symptomatic SMA, functional status (non-sitter, sitter, walker), people who have had prior active treatment of SMA.
NMA
Statistical synthesis of the evidence will be performed if it is clinically appropriate and the number of studies permits this, i.e., if five or more relevant studies are identified and where it is meaningful to pool the data in ITCs. If a particular treatment, outcome, or studies cannot be included in statistical synthesis, a narrative synthesis will be provided. If non-comparative studies are identified, these will also be narratively summarised. Treatment effects will be presented as odds ratios (OR) for dichotomous data, weight mean differences for continuous data or hazard ratios (HR) as appropriate.
Information extracted from the included studies will be summarised and presented in a tabular form. Due to the context-specific nature of economic evaluation, the conduct and findings of studies included in the systematic review will be summarised narratively.

**Analysis of subgroups or subsets**

The relevant subgroups are:

• Number of SMN2 gene copies in people with pre-symptomatic SMA
• Functional status (non-sitter, sitter, walker) and baseline motor function and level of motor function
• People who have had prior active treatment for SMA
• SMA type
• By age
• By prior treatment (naïve or successful)
• Patients transition from childhood to adulthood

REVIEW AFFILIATION, FUNDING AND PEER REVIEW

**Review team members**

- Mr Peter Auguste, University of Warwick
- Dr Jo Parsons, University of Warwick
- Dr Mehdi Yousefi, University of Warwick
- Mr Mubarak Patel, University of Warwick
- Ms Anna Brown, University of Warwick
- Mr Amin Mehrabian, University of Warwick
- Ms Janette Parr, University of Warwick.ac.uk
- Professor Amy Grove, University of Warwick

**Review affiliation**

University of Warwick

**Funding source**

Evidence Synthesis Programme, National Institute for Health and Care Research

**Named contact**

Peter Auguste. Warwick Medical School, Coventry, CV4 7AL
[p.auguste@warwick.ac.uk](mailto:p.auguste@warwick.ac.uk)

TIMELINE OF THE REVIEW

**Review timeline** 1 change

Start date: 31 January 2024. End date: 31 January 2025

**Date of first submission to PROSPERO**

12 February 2024

**Date of registration in PROSPERO**

13 February 2024

CURRENT REVIEW STAGE

**Publication of review results**

The intention is to publish the review once completed.The review will be published in English

**Stage of the review at this submission** 1 change

| **Review stage** | **Started** | **Completed** |
| --- | --- | --- |
| Pilot work | ✓ | ✓ |
|  |  |  |
| Formal searching/study identification | ✓ | ✓ |
|  |  |  |
| Screening search results against inclusion criteria | ✓ |  |
|  |  |  |
| Data extraction or receipt of IP | ✓ |  |
|  |  |  |
| Risk of bias/quality assessment |  |  |
|  |  |  |
| Data synthesis |  |  |

**Review status**

The review is currently planned or ongoing.

ADDITIONAL INFORMATION

**PROSPERO version history**

- Version 1.1 published on 20 May 2024
- Version 1.0 published on 13 Feb 2024

**Review conflict of interest**

None known

**Country**

England

**Medical Subject Headings**

Cost-Benefit Analysis; Cost-Effectiveness Analysis; Humans; Muscular Atrophy, Spinal; Spinal Muscular Atrophies of Childhood

**Revision note** 1 change

During screening we revised the inclusion criteria for the clinical effectiveness systematic review to exclude:Case reportsCase series to where number of participants is less than 20Conference abstracts Single-arm studies when there are no comparators, where the number of participants is less than 20 and/or where there is less than 12 months results from when participants receive treatmentThese changes were made to have a manageable number of full text articles included in the systematic review. Given the clinical evidence identified, we were satisfied that these study designs would not provide any added value to the results of the systematic review.

**Disclaimer**

The content of this record displays the information provided by the review team. PROSPERO does not peer review registration records or endorse their content.

PROSPERO accepts and posts the information provided in good faith; responsibility for record content rests with the review team. The owner of this record has affirmed that the information provided is truthful and that they understand that deliberate provision of inaccurate information may be construed as scientific misconduct.

PROSPERO does not accept any liability for the content provided in this record or for its use. Readers use the information provided in this record at their own risk.

Any enquiries about the record should be referred to the named review contact

# Supplemental material Table S3: PRISMA Checklist

| **Section and Topic** | **Item #** | **Checklist item** | **Location where item is reported** |
| --- | --- | --- | --- |
| **TITLE** | | |  |
| Title | 1 | Identify the report as a systematic review. | P1 |
| **ABSTRACT** | | |  |
| Abstract | 2 | See the PRISMA 2020 for Abstracts checklist. | P2 |
| **INTRODUCTION** | | |  |
| Rationale | 3 | Describe the rationale for the review in the context of existing knowledge. | P2-4 |
| Objectives | 4 | Provide an explicit statement of the objective(s) or question(s) the review addresses. | P4 |
| **METHODS** | | |  |
| Eligibility criteria | 5 | Specify the inclusion and exclusion criteria for the review and how studies were grouped for the syntheses. | P5-7 & Table 1 |
| Information sources | 6 | Specify all databases, registers, websites, organisations, reference lists and other sources searched or consulted to identify studies. Specify the date when each source was last searched or consulted. | P5 |
| Search strategy | 7 | Present the full search strategies for all databases, registers and websites, including any filters and limits used. | P5 & Appendix 1 |
| Selection process | 8 | Specify the methods used to decide whether a study met the inclusion criteria of the review, including how many reviewers screened each record and each report retrieved, whether they worked independently, and if applicable, details of automation tools used in the process. | P5-6 |
| Data collection process | 9 | Specify the methods used to collect data from reports, including how many reviewers collected data from each report, whether they worked independently, any processes for obtaining or confirming data from study investigators, and if applicable, details of automation tools used in the process. | P7 |
| Data items | 10a | List and define all outcomes for which data were sought. Specify whether all results that were compatible with each outcome domain in each study were sought (e.g. for all measures, time points, analyses), and if not, the methods used to decide which results to collect. | P7 |
|  | 10b | List and define all other variables for which data were sought (e.g. participant and intervention characteristics, funding sources). Describe any assumptions made about any missing or unclear information. | P7 |
| Study risk of bias assessment | 11 | Specify the methods used to assess risk of bias in the included studies, including details of the tool(s) used, how many reviewers assessed each study and whether they worked independently, and if applicable, details of automation tools used in the process. | P8 |
| Effect measures | 12 | Specify for each outcome the effect measure(s) (e.g. risk ratio, mean difference) used in the synthesis or presentation of results. | P8 |
| Synthesis methods | 13a | Describe the processes used to decide which studies were eligible for each synthesis (e.g. tabulating the study intervention characteristics and comparing against the planned groups for each synthesis (item #5)). | P8 |
|  | 13b | Describe any methods required to prepare the data for presentation or synthesis, such as handling of missing summary statistics, or data conversions. | n/a |
|  | 13c | Describe any methods used to tabulate or visually display results of individual studies and syntheses. | P8 |
|  | 13d | Describe any methods used to synthesize results and provide a rationale for the choice(s). If meta-analysis was performed, describe the model(s), method(s) to identify the presence and extent of statistical heterogeneity, and software package(s) used. | P8 |
|  | 13e | Describe any methods used to explore possible causes of heterogeneity among study results (e.g. subgroup analysis, meta-regression). | P8 |
|  | 13f | Describe any sensitivity analyses conducted to assess robustness of the synthesized results. | n/a |
| Reporting bias assessment | 14 | Describe any methods used to assess risk of bias due to missing results in a synthesis (arising from reporting biases). | P12 |
| Certainty assessment | 15 | Describe any methods used to assess certainty (or confidence) in the body of evidence for an outcome. | n/a |
| **RESULTS** | | |  |
| Study selection | 16a | Describe the results of the search and selection process, from the number of records identified in the search to the number of studies included in the review, ideally using a flow diagram. | P8 |
|  | 16b | Cite studies that might appear to meet the inclusion criteria, but which were excluded, and explain why they were excluded. | n/a |
| Study characteristics | 17 | Cite each included study and present its characteristics. | P10-12 & Appendix 3 |
| Risk of bias in studies | 18 | Present assessments of risk of bias for each included study. | P12 & Supplemental Table 1,2 & 3 |
| Results of individual studies | 19 | For all outcomes, present, for each study: (a) summary statistics for each group (where appropriate) and (b) an effect estimate and its precision (e.g. confidence/credible interval), ideally using structured tables or plots. | P13- 34 & Appendix 4,5 & 6 |
| Results of syntheses | 20a | For each synthesis, briefly summarise the characteristics and risk of bias among contributing studies. | P13- 34 & Appendix 4,5 & 6 |
|  | 20b | Present results of all statistical syntheses conducted. If meta-analysis was done, present for each the summary estimate and its precision (e.g. confidence/credible interval) and measures of statistical heterogeneity. If comparing groups, describe the direction of the effect. | n/a |
|  | 20c | Present results of all investigations of possible causes of heterogeneity among study results. | n/a |
|  | 20d | Present results of all sensitivity analyses conducted to assess the robustness of the synthesized results. | n/a |
| Reporting biases | 21 | Present assessments of risk of bias due to missing results (arising from reporting biases) for each synthesis assessed. | n/a |
| Certainty of evidence | 22 | Present assessments of certainty (or confidence) in the body of evidence for each outcome assessed. | n/a |
| **DISCUSSION** | | |  |
| Discussion | 23a | Provide a general interpretation of the results in the context of other evidence. | P34- 37 |
|  | 23b | Discuss any limitations of the evidence included in the review. | P39- 40 |
|  | 23c | Discuss any limitations of the review processes used. | P39- 40 |
|  | 23d | Discuss implications of the results for practice, policy, and future research. | P37- 38 |
| **OTHER INFORMATION** | | |  |
| Registration and protocol | 24a | Provide registration information for the review, including register name and registration number, or state that the review was not registered. | P4 |
|  | 24b | Indicate where the review protocol can be accessed, or state that a protocol was not prepared. | P4 |
|  | 24c | Describe and explain any amendments to information provided at registration or in the protocol. | n/a |
| Support | 25 | Describe sources of financial or non-financial support for the review, and the role of the funders or sponsors in the review. | P64 |
| Competing interests | 26 | Declare any competing interests of review authors. | P65 |
| Availability of data, code and other materials | 27 | Report which of the following are publicly available and where they can be found: template data collection forms; data extracted from included studies; data used for all analyses; analytic code; any other materials used in the review. | On submission platform |

# Supplementary S4: Search information

Search strategies: original search

**Embase (Ovid)**

Date searched: 29/01/24

Embase <1974 to 2024 January 26>

1 exp hereditary spinal muscular atrophy/ or spinal muscular atrophy/ 14233

2 (spinal muscul* atroph* or SMA).kf,tw. 50728

3 (Werdnig adj Hoffman*).kf,tw. 423

4 (Kugelberg adj Welander*).kf,tw. 216

5 1 or 2 or 3 or 4 [disease terms] 55864

6 nusinersen/ 1913

7 (nusinersen or spinraza* or isis smn* or isis 396443).kf,tn,tw. 1699

8 6 or 7 2087

9 risdiplam/ 566

10 (risdiplam or evrysdi* or RG 7916 or RG7916).kf,tn,tw. 484

11 9 or 10 630

12 5 and 8 1879

13 5 and 11 576

14 limit 12 to dc=20171001-20241231 1814

15 limit 13 to dc=20200101-20241231 504

16 (exp animal/ or exp animal experiment/) not (exp human/ or exp human experiment/ or conference abstract.pt.) 5077905

17 editorial.pt. 794403

18 14 or 15 1980

19 18 not (16 or 17) 1908

20 limit 19 to english language 1837

Lines 1-4 are adapted from the search strategy used in: Wadman RI, van der Pol WL, Bosboom WMJ, Asselman FL, van den Berg LH, Iannaccone ST, et al. Drug treatment for spinal muscular atrophy types II and III. Cochrane Database of Systematic Reviews 2020;2020(1):CD006282. <https://dx.doi.org/10.1002/14651858.CD006282.pub5>

The Embase search strategy was peer reviewed by Naila Dracup, Information Specialist, Warwick Medical School before translation to the other databases and registers, which was undertaken by Rachel Court, Senior Information Specialist, Warwick Medical School.

**MEDLINE (Ovid)**

Date searched: 29/01/24

Ovid MEDLINE(R) ALL <1946 to January 26, 2024>

1 exp Muscular Atrophy, Spinal/ 6593

2 (spinal muscul* atroph* or SMA).mp. 33123

3 (Werdnig adj Hoffman*).mp. 399

4 (Kugelberg adj Welander*).mp. 210

5 1 or 2 or 3 or 4 [disease terms] 34967

6 (nusinersen or spinraza* or isis smn* or isis 396443).mp. 772

7 (risdiplam or evrysdi* or RG 7916 or RG7916).mp. 163

8 5 and 6 731

9 5 and 7 149

10 (201710* or 201711* or 201712* or 2018* or 2019* or 2020* or 2021* or 2022* or 2023* or 2024*).dt,ez,da,cb. 9960713

11 8 and 10 712

12 limit 8 to yr="2017 -Current" 723

13 11 or 12 724

14 (2020* or 2021* or 2022* or 2023* or 2024*).dt,ez,da,cb. 7036354

15 9 and 14 141

16 limit 9 to yr="2020 -Current" 141

17 15 or 16 141

18 (exp Animals/ or exp Animal Experimentation/) not (Humans/ or exp Human Experimentation/) 5191116

19 Editorial.pt. 680347

20 13 or 17 769

21 20 not (18 or 19) 731

22 limit 21 to english language 700

**Science Citation Index and Conference Proceedings (Web of Science)**

Date searched: 29/01/24

Database: Web of Science Core Collection

Editions searched/Entitlements: WOS.SCI: 1970 to 2024, WOS.ISTP: 1990 to 2024

|  | Search Query | Results |
| --- | --- | --- |
| #1 | TS=(("spinal muscul*" NEAR/0 atroph*) OR SMA) | 49238 |
| #2 | TS=(Werdnig NEAR/0 Hoffman*) | 413 |
| #3 | TS=(Kugelberg NEAR/0 Welander*) | 113 |
| #4 | #1 OR #2 OR #3 | 49409 |
| #5 | TS=(nusinersen OR spinraza* OR "isis smn*" OR "isis 396443") | 1231 |
| #6 | #4 AND #5  #Timespan: 2017-10-01 to 2024-12-31 (Index date) | 1061 |
| #7 | TS=(risdiplam OR evrysdi* OR "RG 7916" OR RG7916) | 257 |
| #8 | #4 AND #7  Timespan: 2020-01-01 to 2024-12-31 (Index date) | 208 |
| #9 | #6 OR #8 | 1163 |
| #10 | #9 and Editorial Material (Exclude – Document Types) | 1102 |
| #11 | (#10) AND (LA==("ENGLISH")) | 1072 |

The Ovid Medline search strategy was translated for use in Web of Science with the aid of the Polyglot Search Translator: Clark JM, Sanders S, Carter M, Honeyman D, Cleo G, Auld Y, et al. Improving the translation of search strategies using the Polyglot Search Translator: a randomized controlled trial. J Med Libr Assoc 2020;108(2):195-207. <http://dx.doi.org/10.5195/jmla.2020.834>

**Cochrane CENTRAL and Cochrane Database of Systematic Reviews (Cochrane Library, Wiley)**

Date searched: 29/01/24

ID Search Hits

#1 [mh "Muscular Atrophy, Spinal"] 128

#2 ((spinal NEXT muscul* NEXT atroph*) OR SMA):ti,ab,kw 1180

#3 (Werdnig NEXT Hoffman*):ti,ab,kw 23

#4 (Kugelberg NEXT Welander*):ti,ab,kw 45

#5 #1 OR #2 OR #3 OR #4 1212

#6 ((nusinersen OR spinraza* OR ("isis" NEXT smn*) OR "isis 396443")):ti,ab,kw 86

#7 (risdiplam OR evrysdi* OR "RG 7916" OR RG7916):ti,ab,kw 63

#8 #5 AND #6 86

#9 #5 AND #7 63

#10 #5 AND #6 with Cochrane Library publication date Between Oct 2017 and Dec 2024 84

#11 #5 AND #7 with Cochrane Library publication date Between Jan 2020 and Dec 2024 50

#12 #5 AND #6 with Publication Year from 2017 to 2024, in Trials 73

#13 #5 AND #7 with Publication Year from 2020 to 2024, in Trials 47

#14 #10 OR #12 85

#15 #11 OR #13 50

#16 #14 OR #15 in Cochrane Reviews, Trials 124

The Ovid Medline search strategy was translated for use in the Cochrane Library with the aid of the Polyglot Search Translator: Clark JM, Sanders S, Carter M, Honeyman D, Cleo G, Auld Y, et al. Improving the translation of search strategies using the Polyglot Search Translator: a randomized controlled trial. J Med Libr Assoc 2020;108(2):195-207. <http://dx.doi.org/10.5195/jmla.2020.834>

**International HTA database (INAHTA)** <https://database.inahta.org/>

Date searched: 29/01/24

(("Muscular Atrophy, Spinal"[mhe]) OR ("spinal muscular atrophy" OR "spinal muscular atrophies" OR SMA OR "Werdnig Hoffman" OR "Kugelberg Welander"))

Year: 2017 to 2024

Language: English 4 results

**ClinicalTrials.gov (classic website)** <https://classic.clinicaltrials.gov/ct2/home>

Date searched: 29/01/24

search box on home page:

Other terms: nusinersen OR spinraza OR risdiplam OR evrysdi 73 studies found

**WHO International Clinical Trials Registry Platform** <https://trialsearch.who.int/Default.aspx>

Date searched: 29/01/24

search box on home page:

nusinersen OR spinraza OR risdiplam OR evrysdi 85 trials found

**Websites** Date searched: 25/01/24

National Institute for Health and Care Excellence (NICE) <https://www.nice.org.uk/guidance/published?sp=on>

*Search for published guidance*

*Filter: Type: Guidance*

Filter by title or keyword:

Spinal muscular atrophy 4 results

- <https://www.nice.org.uk/guidance/ta755> 6 documents downloaded
- <https://www.nice.org.uk/guidance/hst15> 1 document downloaded
- <https://www.nice.org.uk/guidance/hst24> 1 document downloaded
- <https://www.nice.org.uk/guidance/ta588> 2 documents downloaded

SMA 0 results

nusinersen 1 result (already identified above)

risdiplam 1 result (already identified above)

onasemnogene 2 results (already identified above)

Total: 4 technology assessments identified; 10 potentially relevant documents downloaded

Scottish Medicines Consortium (SMC) <https://www.scottishmedicines.org.uk/>

*Search box on homepage*

spinal muscular atrophy 6 results, of which 3 relevant

- <https://www.scottishmedicines.org.uk/medicines-advice/nusinersen-spinraza-fullsubmission-131818/>
- <https://www.scottishmedicines.org.uk/medicines-advice/risdiplam-evrysdi-full-smc2401/>
- <https://www.scottishmedicines.org.uk/medicines-advice/onasemnogene-abeparvovec-zolgensma-full-smc2311/>

nusinersen 2 results (already identified above)

risdiplam 2 results (already identified above)

onasemnogene 2 results (already identified above)

Total: 3 technology assessments identified; 3 potentially relevant documents downloaded

CADTH: Canada’s Drug and Health Technology Agency <https://www.cadth.ca/search>

*All searches limited to ‘Reports’*

Spinal muscular atrophy 23 results, of which 8 projects potentially relevant:

- Nusinersen, reimbursement review 2022 <https://www.cadth.ca/nusinersen-1> (combined clinical and pharmacoeconomic review)
- Health Technology Assessment Recommendations and Managed Entry Agreements Related to Optimizing the Treatment for Pediatric Spinal Muscular Atrophy. *In progress but effective finish date was January 2023 so keep an eye out* <https://www.cadth.ca/health-technology-assessment-recommendations-and-managed-entry-agreements-related-optimizing>
- Risdiplam, reimbursement review 2021 <https://www.cadth.ca/risdiplam> (combined clinical and pharmacoeconomic review)
- Onasemnogene abeparvovec, Reimbursement review 2021 <https://www.cadth.ca/onasemnogene-abeparvovec>
  - clinical report
  - pharmacoeconomic report
- Nusinersen for Adolescents and Adults with Spinal Muscular Atrophy: A Review of Clinical Effectiveness. Rapid review 2020. <https://cadth.ca/nusinersen-adolescents-and-adults-spinal-muscular-atrophy-review-clinical-effectiveness>
- Nusinersen, reimbursement review 2018. <https://www.cadth.ca/nusinersen>
  - clinical report
  - pharmacoeconomic report
- Nusinersen for Adults with Spinal Muscular Atrophy: Clinical Effectiveness. Rapid response report; reference list. 2020. <https://www.cadth.ca/nusinersen-adults-spinal-muscular-atrophy-clinical-effectiveness>
- Nusinersen, reimbursement review 2019. <https://www.cadth.ca/nusinersen-0>
  - clinical report
  - pharmacoeconomic report

SMA 17 results, of which 0 relevant and not already identified above

nusinersen 10 results (all already identified above)

risdiplam 3 results (already identified above)

onasemnogene 6 results (already identified above)

Total: 8 projects identified; 10 potentially relevant reports downloaded, 1 ongoing project web page bookmarked

Institute for Clinical and Economic Review (ICER) <https://icer.org/explore-our-research/assessments/>

*search by keyword; no filters applied*

spinal muscular atrophy 1 result

- An assessment of Onasemnogene Abeparvovec and Nusinersen for Spinal Muscular Atrophy (SMA). 2019 <https://icer.org/assessment/spinal-muscular-atrophy-2019/> (evidence report includes clinical and cost effectiveness)

SMA 10 results, of which 0 relevant and not already identified above

nusinersen 1 result (already identified above)

risdiplam 0 results

onasemnogene 1 result (already identified above)

Total: 1 technology assessment identified; 1 report downloaded

Drugs@FDA , U.S. Food & Drug Administration <https://www.accessdata.fda.gov/scripts/cder/daf/>

nusinersen 1 review found, approval date 2016: <https://www.accessdata.fda.gov/drugsatfda_docs/nda/2016/209531Orig1s000TOC.cfm>

(separate medical, pharmacology, statistical, etc review documents but no pharmacoeconomic / cost effectiveness data). Medical review downloaded.

risdiplam 1 review found, approval date 2020: <https://www.accessdata.fda.gov/drugsatfda_docs/nda/2020/213535Orig1s000TOC.cfm> (separate clincial, statistical, etc review documents but no pharmacoeconomic / cost effectiveness data). Clinical review downloaded.

onasemnogene 0 results

zolgensma 0 results

Total: 2 drug reviews; 2 documents downloaded

MHRA products, Medicines & Healthcare products Regulatory Agency <https://products.mhra.gov.uk/>

nusinersen 5 results (SPCs for nusinersen, risdiplam & onasemnogene; 2 x PARs for risdiplam (that appear to be identical)

risdiplam 5 results, as above.

Total: 1 drug review; 1 document downloaded (risdiplam PAR)

European Medicines Agency (EMA) <https://www.ema.europa.eu/en/homepage>

*search box on homepage*

nusinersen 3 results, of which 1 relevant *downloaded 2 EPAR reports*

risdiplam 3 results, of which 1 relevant *downloaded 2 EPAR reports*

Total: 2 drug reviews; 4 documents downloaded

**Total:**

**21 technology assessments, reviews or other projects identified**

**31 documents downloaded**

**1 webpage bookmarked (ongoing project)**

Search strategies: update search:

*Note: for the update searches, all date limits were removed from the searches, and efforts were made to exclude conference abstracts where possible, in line with the updated review inclusion/exclusion criteria.*

**Embase (Ovid)**

Date searched: 13/01/25

Embase <1974 to 2025 January 09>

1 exp hereditary spinal muscular atrophy/ or spinal muscular atrophy/ 15379

2 (spinal muscul* atroph* or SMA).kf,tw. 54522

3 (Werdnig adj Hoffman*).kf,tw. 426

4 (Kugelberg adj Welander*).kf,tw. 218

5 1 or 2 or 3 or 4 [disease terms] 59966

6 nusinersen/ 2244

7 (nusinersen or spinraza* or isis smn* or isis 396443).kf,tn,tw. 1955

8 6 or 7 2430

9 risdiplam/ 785

10 (risdiplam or evrysdi* or RG 7916 or RG7916).kf,tn,tw. 656

11 9 or 10 857

12 (exp animal/ or exp animal experiment/) not (exp human/ or exp human experiment/ or conference abstract.pt.) 5237204

13 editorial.pt. 833813

14 (conference abstract or conference review).pt. 5366112

15 5 and (8 or 11) 2463

16 15 not (12 or 13 or 14) 1428

17 limit 16 to english language 1340

**MEDLINE (Ovid)**

Date searched: 13/01/25

Ovid MEDLINE(R) ALL <1946 to January 10, 2025>

1 exp Muscular Atrophy, Spinal/ 6967

2 (spinal muscul* atroph* or SMA).mp. 35673

3 (Werdnig adj Hoffman*).mp. 403

4 (Kugelberg adj Welander*).mp. 214

5 1 or 2 or 3 or 4 [disease terms] 37550

6 (nusinersen or spinraza* or isis smn* or isis 396443).mp. 931

7 (risdiplam or evrysdi* or RG 7916 or RG7916).mp. 245

8 (exp Animals/ or exp Animal Experimentation/) not (Humans/ or exp Human Experimentation/) 5298161

9 Editorial.pt. 717606

10 5 and (6 or 7) 953

11 10 not (8 or 9) 913

12 limit 11 to english language 874

**Science Citation Index (Web of Science)**

Date searched: 13/01/25

Database: Web of Science Core Collection

Editions searched/Entitlements: WOS.SCI: 1970 to 2024

|  | Search Query | Results |
| --- | --- | --- |
| #1 | TS=(("spinal muscul*" NEAR/0 atroph*) OR SMA) Editions: WOS.ISTP,WOS.SCI | 53586 |
| #2 | TS=(Werdnig NEAR/0 Hoffman*) Editions: WOS.SCI,WOS.ISTP | 459 |
| #3 | TS=(Kugelberg NEAR/0 Welander*) Editions: WOS.SCI,WOS.ISTP | 146 |
| #4 | TS=(nusinersen OR spinraza* OR "isis smn*" OR "isis 396443") Editions: WOS.SCI,WOS.ISTP | 1435 |
| #5 | TS=(risdiplam OR evrysdi* OR "RG 7916" OR RG7916) Editions: WOS.SCI,WOS.ISTP | 354 |
| #6 | #1 OR #2 OR #3 Editions: WOS.SCI | 48995 |
| #7 | #6 AND #4 Editions: WOS.SCI | 1288 |
| #8 | #6 AND #5 Editions: WOS.SCI | 328 |
| #9 | #7 OR #8 Editions: WOS.SCI | 1444 |
| #10 | (#9) NOT DT=(Editorial Material) Editions: WOS.SCI | 1376 |
| #11 | ((#10) AND LA=(English)) NOT DT=(Meeting Abstract) Editions: WOS.SCI | 976 |

**Cochrane CENTRAL and Cochrane Database of Systematic Reviews (Cochrane Library, Wiley)**

Date searched: 13/01/25

ID Search Hits

#1 [mh "Muscular Atrophy, Spinal"] 153

#2 ((spinal NEXT muscul* NEXT atroph*) OR SMA):ti,ab,kw 1320

#3 (Werdnig NEXT Hoffman*):ti,ab,kw 25

#4 (Kugelberg NEXT Welander*):ti,ab,kw 46

#5 #1 OR #2 OR #3 OR #4 1357

#6 ((nusinersen OR spinraza* OR ("isis" NEXT smn*) OR "isis 396443")):ti,ab,kw 93

#7 (risdiplam OR evrysdi* OR "RG 7916" OR RG7916):ti,ab,kw 71

#8 #5 AND #6 93

#9 #5 AND #7 70

#10 #8 OR #9 in Cochrane Reviews, Trials 148

**International HTA database (INAHTA)** <https://database.inahta.org/>

Date searched: 13/01/25

(("Muscular Atrophy, Spinal"[mhe]) OR ("spinal muscular atrophy" OR "spinal muscular atrophies" OR SMA OR "Werdnig Hoffman" OR "Kugelberg Welander"))

Language: English 12 results

**ClinicalTrials.gov** <https://clinicaltrials.gov/>

Date searched: 13/01/25

search box on home page:

Other terms: nusinersen OR spinraza OR risdiplam OR evrysdi 80 studies found

**WHO International Clinical Trials Registry Platform** <https://trialsearch.who.int/>

Date searched: 13/01/25

search box on home page:

nusinersen OR spinraza OR risdiplam OR evrysdi 91 trials found

**Websites**

Date searched: 16/01/25

National Institute for Health and Care Excellence (NICE**)** <https://www.nice.org.uk/guidance/published?sp=on>

*Search for published guidance*

*Filter: Type: Guidance*

*Filter: Last updated date: From 01/01/2024 To 16/01/2025*

Filter by keyword or reference number:

Spinal muscular atrophy 0 results

SMA 0 results

nusinersen 0 results

risdiplam 0 results

onasemnogene 0 results

Total: 0 new or updated results since original search

Scottish Medicines Consortium (SMC) <https://www.scottishmedicines.org.uk/>

*Search box on homepage*

spinal muscular atrophy 6 results, 0 new/updated since 2024

nusinersen 2 results, 0 new

risdiplam 2 results, 0 new

onasemnogene 2 results, 0 new

Total: 0 new or updated results since original search

Canada’s Drug Agency (CDA-AMC) <https://www.cda-amc.ca/search>

*All searches limited to ‘Reports’; results sorted by ‘Last updated’ so new reports can be easily identified.*

Spinal muscular atrophy 14 results, 0 new/updated since previous search

SMA 1 new result, of which 0 relevant

nusinersen 0 new results

risdiplam 0 new results

onasemnogene 0 new results

Checked ongoing project webpage identified previously: <https://www.cadth.ca/health-technology-assessment-recommendations-and-managed-entry-agreements-related-optimizing> (now redirects to: <https://www.cda-amc.ca/health-technology-assessment-recommendations-and-managed-entry-agreements-related-optimizing> ) *Project Status remains “in progress”*

Total: 0 new or updated results since original search

Institute for Clinical and Economic Review (ICER) <https://icer.org/explore-our-research/assessments/>

*search by keyword; no filters applied*

spinal muscular atrophy 2 results, 1 new but not relevant (about apitegromab)

SMA 9 results, of which 1 new but not relevant

nusinersen 0 new results

risdiplam 0 results

onasemnogene 0 new results

Total: 0 new or updated results since original search

Drugs@FDA , U.S. Food & Drug Administration <https://www.accessdata.fda.gov/scripts/cder/daf/>

nusinersen 0 new review documents since previous search

risdiplam 0 new review documents since previous search

onasemnogene 0 results

zolgensma 0 results

Total: 0 new or updated review documents since original search

MHRA products, Medicines & Healthcare products Regulatory Agency <https://products.mhra.gov.uk/>

nusinersen 7 results, 2 new but not relevant (SPCs or patient information leaflets)

risdiplam 7 results, as above

Total: 0 new or updated review documents since original search

European Medicines Agency (EMA) <https://www.ema.europa.eu/en/homepage>

*‘Find medicine’ search box on homepage*

nusinersen 1 result; EPAR has been updated in 2024 but only with minor administrative changes/changes to SmPC, see <https://www.ema.europa.eu/en/documents/procedural-steps-after/spinraza-epar-procedural-steps-taken-scientific-information-after-authorisation_en.pdf>

*0 downloaded*

risdiplam 1 result; 2 EPAR Assessment Report Variations in 2024:

- Type II variation in relation to SUNFISH study, 25 July 2024: <https://www.ema.europa.eu/en/documents/variation-report/evrysdi-h-c-005145-ii-0022-epar-assessment-report-variation_en.pdf>
- incorporating final results from FIREFISH study, 05 September 2024: <https://www.ema.europa.eu/en/documents/variation-report/evrysdi-h-c-005145-ii-0027-epar-assessment-report-variation_en.pdf>

Total: 2 documents downloaded

**Total: 2 documents downloaded relating to EMA risdiplam approval/assessment**

# Supplementary S5: Example of completed extraction form

| Study details |
| --- |
| Study ID |
| NCT number (trials) |
| First author surname:  Hahn |
| Year of publication:  2022 |
| Country:  Germany |
| Study design:  Real-world data |
| Study setting:  23 centres, including paediatric and neurologic departments |
| Duration of study:  15 months  March 12^th^, 2020 – March 30^th^, 2021 |
| Follow-up period:  Not clear. Mean duration of risdiplam treatment was 5 + 2.7 months in type 1 group, and 3.4 + 1.9 months in type 2 group  Short-term data collected |
| Sample size in each arm:  Single arm study, but grouped into type 1 and type 2 SMA:  Type 1: 31  Type 2: 80 |
| Funding:  Open access funding received. Research funded by Roche |
| Conflicts of interest stated:  Authors received institutional support, medical writing support, research grants, consultancy fees, presenting fees, etc. |

| Aim of the study |
| --- |
| To report real-world data on patient characteristics and short-term safety of risdiplam in Germany |

| Participants |
| --- |
| Inclusion criteria:  2 months old or over at enrolment  Confirmed diagnosis of SMA1 or SMA2  Non-eligible for, no longer responsive to, or not able to tolerate approved treatment options  Female patients needed to have a negative pregnancy test and consent to use highly effective contraception during treatment and 1 month after the last dose  Male patients of reproductive age consented to use highly effective contraceptives during treatment and 4 months after.  Normal liver function tests, coagulation parameters, platelets, and troponin-I at 12  weeks after administration of onasemnogene abeparvovecor at least 1  month after tapering of corticosteroids |
| Exclusion criteria:  Receiving risdiplam in an ongoing trial  Serious medical conditions or treatment precluded safe participation  Minimal treatment-free period of 120 days before starting risdiplam after nursinersen, or 12 or more weeks after onasemnogene. |
| Subtypes:  SMA type 1 and type 2 |
| Age:  Type 1: mean age 13.5 years. 52.8% of patients with SMA1 were 5–12 years old  Type 2: mean age 29 years. Included mostly adults across all age groups (range 3–60 years) |
| Gender:  SMA 1: 14 female, 17 male/31  SMA 2: 49 female, 31 male/80 |
| Total number of participants enrolled:  134 enrolled  SMA1 – 36  SMA2 - 98 |
| Sample attrition/drop out:  1 patient was not eligible, 133 entered CUP and 22 were not treated. |
| Number of participants analysed:  111 participants received 1 or more dose |
| Lost to follow-up:  none |
| Time from diagnosis of SMA to study entry:  Not reported |
| Co-morbidities:  Not reported |
| Prior treatment:  Nusinersen: type 1: 23/31 (74.2%), type 2: 37/80 (46.3%)  Onasemnagene: 0  No prior treatment: type 1: 8/31, type 2: 43/80 (53.8%) |
| Pre-symptomatic diagnosis:  n/a |
| SMA type:  Type 1: 31  Type 2: 80 |
| Number of SMN2 copies:  Not reported |
| Relapse rate:  Not reported |
| Age at symptom onset (mean):  Type 1: 2.65 years  Type 2: 11.1 years |
| Age at treatment initiation:  Age at enrolment reported:  Type 1: mean age 13.5 years.  Type 2: mean age 29 years. |
| Best motor function the person obtained:  Supports head unaided: type 1: 5/31, type 2: 22/80  Sitting unaided: type 1: 0, type 2: 36/80  Crawls on the stomach: type 1: 0, type 2: 3/80  Stands unaided: type 1: 0, type 2: 0  Walks unaided: type 1: 0, type 2: 0  None: type 1: 26/31, type 2: 19/80 |

| Intervention and comparators |
| --- |
| Type of drug:  Risdiplam |
| Method of administration:  Oral administration (self-administration/ caregiver at home) |
| Dose:  (0.75 mg/mL aqueous solution)  0.2 mg/kg risdiplam for patients  between 2 months and 2 years of age, 0.25 mg/kg for  patients > 2 years of age and < 20 kg, and a fixed dose of  5 mg for patients > 2 years of age and ≥ 20 kg |
| Frequency:  Once daily at approximately the same time each day.  Treatment was continued for as long as the treating physician considered treatment beneficial  for the patient.  Twice monthly visits were recommended to recalculate the dose based on body weight and resupply patients with medication. |
| Comparator/s (type, method of administration, dose, frequency:  n/a |
| Definition of best supportive care as described by trialists:  n/a |
| Switch between treatments:  Not reported |
| Follow-up:  Not clear. The mean duration of risdiplam treatment was 5 months in the type 1 group and 3.4 months in the type 2 group |
| Treatment duration:  15 months |

| Outcomes |
| --- |
| Primary outcomes    Not specified; only safety outcomes are reported  Adverse event documentation  Serious adverse events  Non serious adverse events |
| Secondary outcomes    Not specified |
| Method of assessing outcomes    **Patients**   - There had to be a minimal treatment-free period of 120 days before starting risdiplam therapy following the administration of nusinersen, or of ≥12 weeks following onasemnogene abeparvovectherapy.   **Treatment and Dosing**   - The decision for Risdiplam therapy was made by the attending physician based on a positive benefit/risk assessment. - Risdiplam could be self-administered by the patient or a parent/caregiver at home. - The dosing scheme was identical to the dose recommendation in the Evrysdi Summary of Product Characteristics: 0.2 mg/kg risdiplam for patients between 2 months and 2 years of age, 0.25 mg/kg for patients >2 years of age and <20 kg, and a fixed dose of 5 mg for patients >2 years of age and ≥20 kg. - Treatment with risdiplam was continued as long as the treating physician considered the treatment beneficial for the patient.   **Adverse Event Documentation**   - The adverse event (AE) severity was assessed by the NCI CTCAE v5.0 (National Cancer Institute Common Terminology Criteria for Adverse Events) grading scale. - The treating physicians were required to report all AEs, serious AEs (SAEs), special situation reports, and product complaints until 28 days after the final dose the patient received in the CUP. |
| Timing of assessment    This analysis covers reporting collected over 15 months. |
| Study endpoint    “Therefore, endpoints such as time to first AE and the number of events per participant were not captured, and potential associations of AEs with baseline characteristics were not analysed.” |
| Adverse event     - During the safety reporting period, a total of 130 AEs were observed in 44 case reports. For SMA1, 30 AEs were reported in 13 cases with 2 SAEs in 1 patient. For SMA2, 100 AEs were reported in 31 cases, including 8 SAEs in 2 patients. - No SAEs with a fatal outcome were reported in this CUP. Risdiplam treatment was permanently discontinued at the patient’s request in a single patient with SMA2, who suffered from SAEs with pre-existing diverticulitis, which was exacerbated 3 times during therapy. - Among the 3 patients with SAEs, one preschool child with SMA1 developed pneumonia twice during the reporting period. The patient recovered following hospitalizations and antibiotic therapy. - An adult patient with SMA2 and pre-existing diverticulitis, deep vein thrombosis, and pain, presented with fever, abdominal pain, two episodes of diarrhoea, constipation, and increased C-reactive protein levels. An abdominal computer tomography showed a segmented sigma diverticulitis with occult perforation and inflamed inhibition of the surrounding peritoneal lipid tissue, with the involvement of a neighbouring ileum fold. The patient recovered after hospitalization and antibiotic therapy. - In a preadolescent child with SMA2 initially treated with nusinersen, serious esophageal hypomotility was observed during risdiplam treatment approximately 2 weeks after starting risdiplam therapy, which persisted at the time of reporting. - The most common non-serious AEs in SMA1 were gastrointestinal disorders: diarrhoea was observed 3 times (10.0% of all AEs), abdominal pain and salivary hypersecretion both occurred 2 times (6.7% of all AEs). In SMA2, diarrhoea was the most common non-serious AE (9 events, 9.0% of all AEs), followed by headache (6 events, 6.0% of all AEs), and aphthous ulcer, constipation, nausea, and circumstance or information capable of leading to a medication error (all 4 events, 4.0% of all AEs). |
| Health-related quality of life: Yes/No; which measures used?    No |
| Length of follow-up    Not clear. The mean duration of risdiplam treatment was 5 months in the type 1 group and 3.4 months in the type 2 group  Treatment duration is 15 months |
| Effect size     - Between March 12, 2020, and March 30, 2021, 36 patients with SMA1 and 98 patients with SMA2 were enrolled at 23 centres. - The first patients started risdiplam treatment on June 26, 2020 (SMA1), and on September 14, 2020 (SMA2). - Mean ± SD (standard deviation) duration of risdiplam treatment was 5.0 ± 2.7 months in the SMA1 cohort and 3.4 ± 1.9 months in the SMA2 cohort. - Baseline demographic characteristics, baseline motor function, and pre-treatment characteristics of both the enrolled population and patients who were treated with risdiplam are presented in Table 1. - The distribution of patients by age and body weight is shown in Fig. 2: 52.8% of patients with SMA1 were 5–12 years old, with a range of 3–52 years, while the SMA2 population included mostly adults across all age groups (range 3–60 years). |
| Statistical tests (standard deviation, 95% CI, standard error, p-values):     - Data on patient characteristics at baseline, including sex, age, previous therapies (treatment duration and reason why patients were not eligible or could not continue to receive an approved therapy), and motor function (age at onset and highest motor function milestone achieved at baseline) were recorded. - According to legal regulations for compassionate use, no effectiveness data were systematically collected. - AEs/SAEs were classified by Medical Dictionary for Regulatory Activities (MedDRA) System Organ Class (SOC) and Preferred Term (PT). - Percentages of AEs and SAEs were calculated based on the total number of AEs. - The patient collective was analysed using standard descriptive methods. For continuous variables, the median and corresponding minimum and maximum values were reported. For categorical variables, the absolute and relative frequencies were reported. - Analyses were conducted with R Version 3.6.1 (R Foundation for Statistical Computing, Vienna, Austria) and PAIRS (Gandy soft, Henderson, Nevada, USA). |

| Other comments |
| --- |
|  |

# Supplementary Table S6: Characteristics of included studies

| **Study characteristics** | | | | | **Participants** | | | **SMA characteristics** | | **Intervention** | | | |
| --- | --- | --- | --- | --- | --- | --- | --- | --- | --- | --- | --- | --- | --- |
| **Author name and year** | **Sources/ publications reporting on trial** | **Study design** | **Country** | **Setting** | **Number of participants analysed in each arm** | **Age of participants** | **Gender of participants** | **SMA type** | **SMN2 copy numbers** | **Intervention Drug Administration and dose** | **Comparator/s** | **Study length** | **Assessment points/ follow-up** |
| Alruthia 2021 | n/a | Cross-sectional,  Questionnaire | Saudi Arabia | Telephone interview    WhatsApp | Total n=36   nusinersen n=11   Standard of Care n=25 | 1 to >25 years   <10 yrs (55.55%)   >25 yrs n=10 (27.78%) | Female 55% | 1-3:     1 (25%)   2 (52.78%)   3 (22.22%)   NB most treated patients had type 1 SMA (54.54%) | Not reported | Nusinersen    Intrathecal.  Four times over 64 days (loading phase). Maintenance dose every 4 months. | Standard of Care | May to August 2020 | No follow-up |
| AlTawari 2024 | n/a | case series (retrospective) | Kuwait | Al Sabah, Al Adan, Al Jahra, Al Farwaniya, Mubarak, and Ibn  Sina Hospitals in Kuwait (treatment initiation period between July 2016 and February 2022). | Case series; 20 paedriatric and 18 adult | aged 2-12 years (pediatric) and 18-55 years (adult) | females (45%) and adult females (28%) | Pediatric Cohort: 40% type I, 40% type II, 20% type III  Adults: 11% type II, 89% type III | Paediatric:  SMN2 Copies: 10% one copy, 50% two copies, 10% three copies  50% had a confirmed family history of SMA  Adult:  17% two copies, 44% three copies, 11% four copies  72% had a confirmed family history of SMA  94% of cases associated with parental consanguinity | Nusinersen Intrathecal  12 mg  loading phase: four 12 mg doses followed by  maintenance doses: every 4 months. | n/a | Almost 80 months | Retrospective study  Almost 80 months |
| Alves 2021 | N/A | Longitudinal cohort study | US | Not reported | Healthy Control n=10   Natural History/SMA patients (no disease-modifying therapy) n=47   Treated SMA patients n=22 (nusinersen n=9) | 0-3 years | Female no. (%)   nusinersen n=5 (55.6%)   Natural History n=19 (40.4%)   Control n=6 (60%)   onasemogene n=2 (28.6%)   Co-therapy n=3 (50%) | 1, 2, 3 and pre-symptomatic | 2, 3, 4+ | Nusinersen    Not reported | Healthy Control   Natural History (no disease-modifying therapy)   onasemnogene abeparvovec  Co-therapy (nusinersen plus onasemnogene abeparvovec early in life) | not reported | All samples available from 0 to 1,095 days old   1-9 data points per subject |
| Aragon-Gawinska 2020 | N/A | Retrospective study using patient registry data | France and Belgium | Not reported | One arm: n=47 | Mean age 22 months (range 2.5–102.8months) | Males, n=28   Females, n=27 | Not reported | ≤2 SMN2 copies, %, sitters 53, non-sitters 62 | Nusinersen    (month 0), after 2 months of treatment (month 2, end of the loading dose), and after months 6, 10, and 14 of treatment | Single arm study | 14 months | 14 months |
| Arnold 2021 | N/A | Observational   Open-label study | US | Ohio State University Wexner Medical Centre | one arm: n=24 | Adult (Ambulatory mean yrs 40±11; non-ambulatory mean yrs 38±12) | 14 women; 10 men (Ambulatory 7 men/6 women; non-ambulatory 3 men/8 women) | Not reported | 3-5 copies | Nusinersen    Intrathecal.  Days 1, 15, 29, and 60 followed by maintenance doses every 4 months | Single arm study | May 2019 to October 2020 | 14 months |
| Arslan 2023 | N/A | Prospective study | Not reported | Not reported | One arm: n=32 | 33.5 (20.0–60.0) years | Male, n=23   Female, n=9 | All SMA, n=32   SMA type 2, n=6   SMA type 3 “sitters”, n=16   SMA type 3 “walkers”, n=10 | 2 copies, n=3   3 copies, n=12   4 copies, n=17 | Nusinersen    Intrathecal injections. 12mg. Baseline (t0), day 29, day 85, and day 274 by CHERISH study protocol due      to the national policies. “First maintenance treatment was administered at 15th month following CHERISH study protocol and subsequent administrations were scheduled at 4-month intervals | Single arm study | 23 months | 23 (15–23) months |
| Audic 2020 | N/A | Retrospective multicentre study | France | 23 French centres for rare paediatric diseases in the French FILNEMUS network for rare neuromuscular disease | one arm: n=123 | Age by subgroup, under 2 years and above 2 years of age, with the second group subsequently split into two additional subgroups (2–5 years and 6–17 years) | Boys n=55   Girls n=68 | SMA type 1a/b, n=10   SMA type 1c, n=24   SMA type 2, n=89 | 2 copies: n=18  3 copies: 96  4 copies: 2 | Intrathecal injection.  Patients were evaluated after 10-, 12- or 14-months’ treatment depending on the study centre | Single arm study | May 2017-February 2019 | At least one year |
| Audic 2023 | N/A | Single arm study | France | 19 French centres dealing with rare paediatric diseases | one arm: n=57 | Age at treatment onset: 2- 34 months: Median age at the start of treatment: 16 months | 30 (53%) boys and 27 (47%) girls | Type 1: 32 (type1a/b = 13 and type 1c = 19)   Type 2: 25 | 2 SMN2 copies: 20 (SMA type 1a/b = 9, type1c = 11 and type2 = 0)   3 SMN2 copies: 37 (type1a/b = 4, type 1c = 8 and type 2 = 25) | Nusinersen    Intrathecally administered.  12 mg (5ml) regardless of age, initiated with injection on day 0, 14, 30 and 60 and then dose administered every 4 months | Single arm study |  |  |
| Axente 2022 | N/A | Retrospective observational study | Romania | The National University Centre for Children Neurorehabilitation, Romania | One arm: n=34 | Age at treatment initiation:   non-sitters: 2-76 months   Sitters: 13-196 months   Walkers: 30-185 months | Not reported | Type 1: 11   Type 2: 16   Type 3: 7 | 2 copies: 16   3 copies: 18 | Nusinersen    Injection. Baseline (treatment initiation T0), every four months up to 26 months. Assessments performed at administration times. | Single arm study | October 2018- October 2021 | Assessments taken at treatment initiation, and every four months up to 26 months |
| Belancic 2023 | N/A | Real-world retrospective study | Croatia | Retrospective and anonymous collection of relevant demographic and clinical data for all Croatian SMA patients treated with nusinersen and reimbursed by the Croatian Health Insurance Fund (CHIF) | One arm: n=52 | Median age: 13.4 years   Type 1: 6.3 years   Type 2: 8.8 years   Type 3p+pa: 12.2 years  Type 3a: 32.4 years   Type 3 total: 24.5 years  Total: 16.4 years | Male: 61.5% | Type 1: 18/52 (34.6%)   Type 2: 6/52 (11.5%)   Type 3p+pa: 11/52 (21.1%)  Type 3a: 17/52 (32.7%)   Type 3 total: 28/52 (53.8%) | Total SMN2 exon 7 copy number:   1 copy: 2   2 copies: 18   3 copies: 16   4 copies: 15   Total SMN2 exon 8 copy number:   1 copy: 5   2 copies: 18   3 copies: 16   4 copies: 13 | Nusinersen    Injection. Dose not specified | Single arm study | April 2018- February 2022 | Assessments examined at baseline, and prior to administration of each nusinersen injection from 5th dose on day 185 of treatment. |
| Berti 2022 | N/A | Longitudinal study | Italy | Not reported | Subgroups: Normal swallowing (n=8)   Tube feeding but some food by mouth (n=2)   Tube Feeding but no tracheostomy (n=4)   Tracheostomy and tube feeding (n=4) | Between 3 weeks and 15 months of age | Male, n=9   Female, n=11 | Type 1 | Not reported | Nusinersen    Not reported | Nusinersen only | Between 12 months and 62 months | Between 12 months and 62 months |
| Bjelica 2023 | N/A | Prospective longitudinal observational study | Germany | One medical school | One arm: n=38 | Mean age at the start of nusinersen: 38.4 years | Male: 24/38 (61.4%) | Type 1: 1 (2.6%)   Type 2: 14 (36.8%)  Type 3: 21 (55.3%)   Type 4: 2 (5.3%) | < 4: 18 (47.4%)   4 or over: 20 (52.6%) | Nusinersen    Intrathecally. Started with loading dose on days 0, 14, 28 and 63, followed by administration every four months | Single arm study | April 2018 and December 2022 | Assessments were taken at baseline, 10, 22 and 30 months of nusinersen treatment |
| Brakemeier 2021 | N/A | Pre/post study | Germany | Department of Neurology, University Medicine Essen | one arm: n=22 | Adult | Female no. (%)   9 (41%) | 2 and 3 (Type 2 n=12 [54%]; Type 3 n=10 [45%]) | x3 n=14 (64%); x4 n=6 (27%); x5 n=1 (4.5%); x≥6 n=1 (4.5%) | Nusinersen    Intrathecal.  12 mg initially for saturation.  Days 1, 14, 28 and 63, then, consecutively at four-month intervals | Single arm study | July 2017 until May 2021 | 14 months |
| Brakemeier 2024 | N/A | Prospective single-arm evaluation | Germany | Department of Neurology, University Hospital, Essen, Germany | one arm: n=25 | Mean age: 34.32 years | Male: 11/25 (44%)   Female: 14/25 (56%) | Type 2: 24/25 (96%)   Type 3: 1/25 (4%) | 2 copies: 3/25 (12%)   3 copies: 22/25 (88%) | Risdiplam    Oral administration. 5mg daily | Single arm study | September 2020 and October 2022 | 12 months |
| Cavaloiu 2024 | n/a | Prospective observation longitudinal study | Romania | Neurology departments of ‘Fundeni’ Clinical Institute in Bucharest and of CF Clinical Hospital in Timisoara, Romania | One cohort of 37 participants | Median of treatment onset:29 (range 18–70) | NR | 62.2% Type 3, 37.8% Type 2 | 73.0% had three copies, 24.3% had two copies, 2.7% had four copies | Nusinersen  Intrathecal  Dose  Not specified but mentions based on the protocol  Frequency  5 doses and then every 4 months thereafter | No control | Ongoing | Baseline, 6 months (after first five doses), and every 4 months thereafter/  Over 54 months follow-up |
| Cavaloiu 2024b | n/a | Prospective cross-sectional descriptive study using the SF-36 questionnaire. | Romania | Prospective cross-sectional descriptive study conducted at "Victor Babes" University of Medicine and Pharmacy Timisoara, Romania | 43 patients | Mean age: 22.52 years.  Median age: 20 years (range 9 to 30.75 years) | Males: n=23, 53.5% (higher in SMA1: 80% and SMA2: 65.2%) | Type 2: 60.4%  Type 3: 27.9%  Type 1: 11.6% | 2 copies: 67.4%  3 copies: 32.6% | Nusinersen interathecal  Dose:  NR  Frequency:  NR | n/a | Six months (August 2021 - January 2022) | Over 6 months |
| Chacko 2022 | N/A | Prospective observational study | Australia | Tertiary hospital setting- Queensland Children’s Hospital Brisbane | one arm: n=28 | Median age at study initiation:   Type 1: 0.42 years   Type 2: 11.04 years   Type 3: 9.08 years | Male:   Type 1: 1/7 (14.3%)   Type 2: 7/12 (58.3%)   Type 3: 7/7 (77.8%) | Type 1: 7   Type 2: 12   Type 3: 9 | 2 copies: type 1:  4/7, type 2: 0, type 3: 0   3 copies:  type 1: 3/7, type 2: 12/12, type 3: 8/9   4 copies: type 1: 0, type 2: 0, type 3: 1/9 | Nusinersen    Intrathecal administration.  Baseline, days 15, 29 and 64. | Single arm study | June 2018- December 2019 | 12 months   (assessments taken at nusinersen doses on days 0, 15, 29, 64, 183, 302 and 365) |
| Chen 2024 | n/a | Retrospective analysis | China | Retrospective study including all pediatric SMA patients treated with intrathecal nusinersen at the Department of Pediatric Neurology, Maternal and Child Health Hospital of Guangxi Zhuang Autonomous Region from November 2020 to September 2023 | 52 patients (1 excluded due to prior risdiplam treatment) | the median age at initiation of treatment was 44.5 months (range 5–192) | 20 females (38.5%), 32 males (61.5%) | SMA Type 2: 76.9% (40/52)  SMA Type 1: 9.6% (5/52)  SMA Type 3: 13.5% (7/52) | 2 copies: 7.7% (4/52).  3 copies: 88.5% (46/52).  4 copies: 3.8% (2/52). | Nusinersen Intrathecal  Dose:  NR  Frequency:  Administered during the loading phase (days 0, 14, 28, and 63) followed by injections every 4 months. | n/a | November 2020 to September 2023 (14 months) | Sociodemographic and clinical data recorded at baseline, including sex, age, SMA type, SMN2 gene copy number, motor status, family history, presence of dysarthria/hyperreflexia, scoliosis, arthrogryposis, and use of ventilation. Follow-up data collected at months 2, 6, 10, and 14.    Motor function assessed at each time point (baseline, months 2, 6, 10, and 14) and compared to baseline values.  Baseline-14 months of follow-up |
| CHERISH/ISIS 396443-CS4 (NCT02292537)  (Mercuri 2018) | Mercuri 2018, Dunaway Young (2023), CADTH nusinersen 2018_clinical   TA588 committee papers 2018_incl CS and ERG report | Randomised Controlled Trial | Multi-national (10 countries): Canada, China, France, Germany, Italy, Japan, Korea, Spain, Sweden, US, Hong Kong | Secondary Care | Nusinersen n=84   Control n=42 | 2-12 years    Median age at screening was 4.0 vs 3.0 years (nusinersen vs control) | Female: Overall n=67 (53%); nusinersen n=46 (55%); Control n=21(50%) | “Most likely to be classified as SMA type 2 or 3” Mercuri (2018) | Nusinersen vs Control; No (%)   2 copies = 6 (7) vs 4 (10)   3 copies = 74 (88) vs 37 (88)   4 copies = 2 (2) vs 1 (2)   Unknown copies = 2 (2) vs 0 | Nusinersen    Intrathecal. 12mg.  Day 1, 29, 85 and 274 | Sham procedure (needle prick to lower back covered with bandage to simulate the appearance of a lumbar puncture) | November 2014 to February 2017 | 15 months (9 months treatment; 6 months follow up) |
| Chiriboga 2016 | N/A | Open label, escalating dose phase 1 study | US | 4 university/ hospital study centres | Total 28:   1mg nusinersen: n=6   3mg nusinersen: n=6   6mg nusinersen: n= 6   9mg nusinersen: n=10 | Total: 6.1 years   1mg: 7.7 years   3mg: 5.3 years   6mg: 6 years   9mg:5.8 years | Male:     Total: 39%   1mg: 83%   3mg: 17%   6mg: 17%   9mg: 40% | Not reported | Not reported | Nusinersen    Intrathecally administered.  1mg, 3mg, 6mg or 9mg | Nusinersen at different doses | Days 8 and 29, and day 85 for participants in 6 and 9mg dose groups     24 of the 28 participants were enrolled in the long-term extension study 9-14 months after the initial dose. | 9-14 months |
| Chiriboga 2024 | JEWELFISH (NCT03032172) | Multicenter, open-label study | Belgium, France, Germany, Italy, Netherlands, Poland, Switzerland, UK, USA | 24 centers across Belgium, France, Germany, Italy, Netherlands, Poland, Switzerland, UK, USA | 173 one arm | 6 months to 60 years | 79 female (45%) | Type 1: n=15 (9%)  Type 2: n=108 (62%)  Type 3: n=51 (29%) | Majority had 3 copies (78%)  1 copy: n=3 (2%)  2 copies: n=13 (8%)  3 copies: n=136 (78)  4 copies: n=22 (13%) | Risdiplam Oral  Dose:  Based on body weight and age (5 mg for patients aged 2–60 years with body weight ≥ 20 kg, 0.25 mg/kg for body weight < 20 kg, 0.2 mg/kg for patients aged 6 months to < 2 years)  Frequency:  Once daily | n/a | 24 months | 24 months  Timing of assessment:  Baseline, 6, 12, 24 months |
| Cho 2023 | N/A | Real-world study | Korea | Dataset 1: Patients with SMA treated with nusinersen under the National Health Insurance coverage for reimbursement between April 2018 and December 2021.  2. Data on perceptions of functional improvements reported by patients or parents from five tertiary hospitals in Korea | one arm: n=137 | 46 (33.6%) were 18 years or over. Mean 28.3 years. | Not reported | (type 1 SMA: 21, type 2 SMA: 103, type 3 SMA: 13). | 1-2 copies (n/%): total: 17/104 (16.3%), type 1: 14/18 (77.8%), type 2: 3/79 (3.8%), type 3: 0 3-4 copies (n/%): total: 86/104 (82.7%), type 1: 4/18 (22.2%), type 2: 76/79 (96.2%), type 3: 7/7 (100%). | Nusinersen    Injection. Loading doses and then every 4 months | Single arm study | 3 years | 1,2- and 3-year follow-ups |
| Colot 2024 | n/a | Prospective multicenter pilot study | Belgium | Hôpital Universitaire des Enfants Reine Fabiola in Brussels and Universitair Ziekenhuis Gent | 53 control and 27 SMA group | 2.5 to 17 years old  Age for the control group (years): 9 (IQR: 7)  SMA group (years):  SMA 1: 4 (IQR: 3)  SMA 2: 9 (IQR: 4)  SMA 3: 15 (IQR: 3)  Total: 9 (IQR: 7) | Gender control group (male): 33  SMA group male:  SMA 1: 2  SMA 2: 8  SMA 3: 3  Total: 13 | SMA Type 1 (9 patients), SMA Type 2 (13 patients), SMA Type 3 (5 patients) | Type 1: 2 copies (majority), 3 copies (others); Type 2: 3 copies (all except one with point mutation in SMN1 and one copy of SMN2); Type 3: 3 copies or more | SMN gene modulator therapy (nusinersen, onasemnogene abeparvovec) | Healthy controls | 1 year | 1 year  Timing of assessment:  Baseline, 1 year |
| Coratti 2021 | N/A | Prospective study | Italy/USA/UK | Specialized neuromuscular sites | One arm: n=77 | Between 2-18 years old | 47 males, 30 females | Type 2 | HFMSE (Hammersmith Functional Motor Scale)    Study: 2 copies n=10, 3 copies n=49, 4+ copies n=2, unknown n=16   Untreated: 2 copies n=48, 3 copies n=448, 4+ copies n=10, unknown n=100   RULM (Revised upper limb module)   Study: 2 copies n=9, 3 copies n=47, 4+ copies n=2, unknown n=15   Untreated: 2 copies n=5, 3 copies n=27, 4+ copies n=0, unknown n=19 | Nusinersen    Not reported | Single arm study | 12 months | 12 months |
| Coratti 2024 | n/a | Retrospective study with prospectively collected data | United States, United Kingdom, Italy | International SMA consortium (iSMAc) dataset from centers in the United States, United Kingdom, and Italy | 104 patients aged 2.64 to 49.6 years, 26 patients younger than 2.5 years. | 0.6 to 49.6 years  Age at Baseline (years):  Mean (SD): 8.79 (9.44)  Median: 6.03 | Female (N,%)62 (47.7 %)  Male (N,%)68 (52.3 %) | Type II | 2 Copies: 20 (15.4%)  3 Copies: 91 (70.0%)  4+ Copies: 2 (1.5%)  Unknown: 17 (13.1%) | Nusinersen  Intrathecal | Untreated individuals (natural history data) | 12 months | Length of Follow-Up (years):  Mean (SD): 0.94 (0.12)  Median: 0.92  [Min, Max]: [0.74, 1.24]  Timing of assessment:  Baseline, 12 months |
| Cornell 2024 | N/A | Real-world data. Single-arm study | Great Britain | Treatment received at home | one arm: n=92 | 2 months to 18 years. Median 12 years | 43/92 male, 47/92 female, 2/92 unspecified | Type 1 n=20/92, 22%. Type 2 n=72/92, 78.2% | Largely unknown n=65/92, 72% | Risdiplam    Oral administration, daily. Standard dose: 2 months to < 2 years: 0.20mg/kg/day     2 or over and less than 20kg: 0.25mg/kg/day     2 or over and 20kg or over 5mg/day | Single arm study | 2-21 months (median 11 months | Not stated. Outcomes were measured at routine follow-up appointments |
| CS2/CS12 (Darras 2019) NCT01703988   (see also NCT02052791 an extension study) | NICE MAA 588 extension company submission 2020, CADTH nusinersen re-submission 2019 | Open Label   Multiple centre   Phase 1/2a   Multiple doses   Dose escalation | US | Secondary Care | Cohort 1 nusinersen 3 mg (n = 8)    Cohort 2 nusinersen 6 mg (n = 8)    Cohort 3 nusinersen 9 mg (n = 9)    Cohort 4 nusinersen 12 mg (n = 9) | 2-15 years   Mean age at screening (months): 7.1 (SD 4.7) | Male: n=15 (54%) | Later-onset SMA (have or are most likely to develop SMA type II or III)   n=11 (39%) SMA Type 2   n=17 (61%) SMA Type 3 | 2 n=1 (4%)   3 n=21 (75%)   4 n=6 (21%) | Nusinersen    Intrathecal.   Cohort 1: 3 mg, Days 1, 29, and 85   Cohort 2: 6 mg, Days 1, 29, and 85    Cohort 3: 9 mg, Days 1 and 85  Cohort 4: 12 mg, Days 1, 29, and 85 | Different cohorts are not compared - focus more on comparing types. | 1,150 days (last data point) | 6 months after day 85 |
| De Wel 2021 | N/A | N=48 Retrospective and n=16 of these patients in prospective | Belgium | Neuromuscular Reference Centre of the Neurology Department at University Hospitals Leuven, Belgium | N=48 Retrospective and n=16 of these patients in prospective | Prospective study group: mean 37.5 aged between 22 and 66 years   Retrospective study group: 37.1 aged between 20 and 66 years | Prospective study: male n=10, female n=6   Retrospective study: male n=22, female n=26 | Type 3 and 4 | Prospective study group n=16   2 copies=0   3 copies, n=13   4 copies, n=2   5 copies=1   Retrospective study group n-48   2 copies, n=5   3 copies=34   4 copies=6   5 copies, n=1 | Nusinersen    Intrathecal administration.  12mg. following the standard of care dosing schedule, for a study duration of 14 months | Single arm study | 14 months | 14 months |
| Duan 2022 | N/A | Non-randomized observational trial | China | All participants attended the Beijing Meier Advocacy & Support Centre for SMA (Meier) | Participants chose whether to receive nusinersen or not:   281 in the non-medication group    51 in the medication group | 1-12 months: non-medication: 11/281, medication: 4/51   13-24 months: non-medication: 34/281, medication: 15/51   2-4 years: non-medication: 77/281, medication: 16/51   5-7 years: non-medication: 52/281, medication: 5/51   8-12 years: non-medication: 38/281, medication: 5/51   13-17 years: non-medication: 30/281, medication: 4/51   18-25 years: non-medication: 22/281, medication: 0   >26 years: non-medication: 17/281, medication: 2/51 | Male:   non-medication: 121/281   Medication: 19/51 | Type 1: non-medication: 45/281, medication: 14/51   Type 2: non-medication: 167/281, medication: 30/51   Type 3: non-medication: 69/281, medication: 7/51 | Not reported | Nusinersen    The dosage and administration followed the instructions and medical prescription | Non-medication- no information included, just that no nusinersen was given | December 2019- September 2020 | Three surveys conducted: December 2019, April 2020 and September 2020 |
| Duong 2021 | N/A | Observational, Case Series,   Before and After | US | Not reported | One arm: n=42 | Adult | Male n=24 (57%) | Type 2 and 4 | 2, 3 and 4 (62% 3 copies) | Nusinersen    Intrathecal.  12mg (loading dose).  Baseline, day 14, day 28, day 58, and every 4 months thereafter | Single arm study | January 2017 to March 2019 | 24 months |
| Edel 2021 | N/A | Longitudinal study | UK | NIHR Great Ormond Street Hospital Biomedical Research Centre, London, U | one arm: n=20 | Median 9.57 months (4–21.6) | 7 males   13 female | Type 1 | Not reported | nusinersen   Injection. nusinersen's initial review of 1st dose had a variable time scale,  1st dose to 2nd dose:  2 weeks   2nd dose to 3rd dose: 2 weeks   3rd dose to 4th dose: 1 month   4th dose to 5th dose: 4 months   5th dose to 6th dose: 4 months   6th dose to 7th dose: 4 months   7th dose to 8th dose: 4 months | Single arm study | 18 months; between January 2017 - November 2018 | 10 months |
| EMBRACE (NCT02462759) (Acsadi 2021) | Acsadi et al. 2021, CADTH nusinersen resubmission 2019_clinical,  ICER Spinraza and zolgensma 2019, FDA nusinersen 2016_medical review | Randomized (2:1 ratio), double-blind, sham procedure–controlled study, followed by unblinded amended open-label protocol | Germany and US | 7 settings, not specified | Total 21: Randomized to sham procedure in part 1 (n = 7), Surviving, received nusinersen in part 2 (n = 6), Randomized to nusinersen (n = 14). | Mixed Infantile and Early Childhood Onset. One of the following: onset of clinical signs and symptoms consistent with SMA at ≤ 6 months of age.  onset of clinical signs and symptoms consistent with SMA at ≤ 6 months of age, > 7 months of age at screening,  onset of clinical signs and symptoms consistent with SMA at > 6 months of age, ≤ 18 months of age at screening | Both males and females | Infantile-onset SMA, n (%) sham: 4 (57) move to nusinersen: 3 (50) nusinersen: 9 (64)   Later onset SMA, n (%) sham: 3 (43) moves to nusinersen: 3 (50) nusinersen: 5 (36 | Two, three or two or three | Nusinersen    Intrathecal lumbar puncture. 12 mg nusinersen (scaled by age-equivalent dose based on cerebrospinal fluid [CSF] volume scaling)   Participants originally randomized to nusinersen:10 to 12 doses in total   Randomized to sham procedure and completed both parts of the study: 8 doses. Intrathecal lumbar puncture in four loading doses on days 1, 15, 29, and 64, followed by maintenance doses on days 183 and 302 (every 4 months). | Part 1: Sham procedure (part 2 no comparator as previously received sham received nusinersen vs initially received nusinersen). | August 19, 2015, and December 20, 2016, (data cutoff March 30, 2017, and eligible   participants transferred to Part 2).   Part 1: 14 months (halted early due to demonstrable efficacy). Part two consisted of 28 months (24 months of nusinersen and four-month follow-up from the last dose | Part two consisted of 28 months (24 months of nusinersen and a four-month follow-up from the last dose).     When part 1 was unblinded and terminated, six participants (all in the nusinersen group) had completed the day 422 final follow-up evaluation.    For the group initially randomized to nusinersen, the median (interquartile range) total time (parts 1 and 2 combined) in the study was 995 (range, 890-1010) days. For those randomized to sham procedure, the median (interquartile range) time in part 1 was 302 (230-366) days, followed by nusinersen treatment in part 2 for 656 (653-659) days. |
| ENDEAR/ISIS 396443-CS3B (NCT02193074)  (Finkel 2017) | Finkel 2017, NCT02193074, A Study to Assess the Efficacy and Safety of nusinersen (ISIS 396443) in Infants With Spinal Muscular Atrophy - Study Results - ClinicalTrials.gov | Randomised Controlled Trial | Australia, Belgium, Canada, France,  Germany,  Italy,  Japan,  Korea, Spain,  Sweden,  Turkey,  United Kingdom,  United States | Not reported | Nusinersen n=81   Control n=41 | Infants/symptom onset 6 months or less | Female no. (%): nusinersen 43 (54); Control 24 (59) | “Most likely to be classified as spinal muscular atrophy type 1” Finkel (2017) | 2 copies | Nusinersen    Intrathecal. nusinersen dose adjusted according to the estimated volume of cerebrospinal fluid for the infant’s age on the day of dosing, such that the infant received a dose that was equivalent to a 12-mg dose in a person 2 years of age or older.  Days 1, 15, 29, and 64, and maintenance doses on days 183 and 302 | Sham Procedure (needle prick to the skin over the lumbar spine, which was covered with a bandage to simulate the appearance of a lumbar-puncture injection) | August 2014 to November 2016 | 13 months (planned) |
| European registries (NICE 2020) | NICE | Observational study, analysis of registry data | Germany, Italy, Spain | Not reported | Nusinersen treated n=159   Untreated n=9 | Adults and children | Not reported | Type 3 | All n=168    (n, %)   1 copy =0 (0)   2 copies =14 (8)   3 copies =67 (40)   4 copies =53 (32)   >4 copies =1 (0.6)   Unknown copies =33 (20) | Nusinersen    Intrathecal administration, 12mg, Days 0, 14, 28, and 63 then maintenance doses every four months | Non-treated patients | Not reported | Not reported |
| Fainmesser 2022 | N/A | Prospective observational study | Israel | Tel-Aviv Medical Centre | One arm: n=37 | All: 38 years   Type 2: 38 years  Type 3: 34 years | All: female: 17/37, male: 20/37   Type 1: female: 9/15, male: 6/15   Type 2: female: 8/22, male: 14/22 | Type 2: 15   Type 3: 22 | Not reported- many participants were diagnosed many years ago without testing for SMN2 copies | Nusinersen    Intrathecally administered, 12mg. Loading doses given on days 0 (baseline), 14, 28 and 63   Followed by a maintenance dose every four months | Single arm study | Participants were treated between March 2018 and September 2020. Follow-up until 30 months | Evaluations are taken at baseline, 6 months, and then every 4 months up to 30 months |
| Finkel 2021 | N/A | Phase 2, open-label, multicentre, dose-escalation clinical trial | USA/Canada | three university hospital sites in the USA and one in Canada | 4 treated with 6–12 mg nusinersen (cohort 1)   16 were treated with 12 mg nusinersen, of which 13 had two SMN2 copies (cohort 2) | Infants aged between 3 weeks and 6 months | Male n=12   Female n=8 | Not reported | Two or three SMN2 gene copies | Nusinersen    Intrathecal loading doses.  6 mg equivalent nusinersen (cohort 1) or 12 mg dose equivalent (cohort 2), followed by      maintenance doses of 12 mg equivalent nusinersen.  Four participants were treated with 6 mg equivalent nusinersen on days 1 (Predose evaluations and administration of the first dose), 15, and 85 (loading doses), followed by 12 mg equivalent doses on day 253 and then repeated every 4 months (cohort 1). 16 participants were then treated with 12 mg equivalent nusinersen at days 1, 15, 85 (loading doses), and 253, and repeated every  4 months (cohort 2) | 4 treated with 6–12 mg nusinersen (cohort 1)   16 treated with 12 mg nusinersen, of which 13 had two SMN2 copies (cohort 2) | 3-7 years | Participants attended follow-up visits on days 16, 29, 86, 92, 169, 254, 337, and 442, then every 4 months. |
| FIREFISH (NCT02913482) (Masson 2022) | Masson 2022  Masson appendix   Darras et al 2021  Baranello 2021   EMA  CADTH 2021  Web STA | open-label trial | U.S., Belgium, Brazil, China, Croatia, France, Italy, Japan, Poland, Russia, Saudi Arabia, Serbia, Spain, Switzerland, Turkey, Ukraine: enrolled at sites in Europe (24/41, 58.5%); 1 patient (2.4%) was enrolled in the United States, and 16 patients (39.0%) were enrolled at sites in the rest of the world. The countries with the highest enrolment were China (11/41, 26.8%) and Italy (10/41, 24.4%) | 14 hospitals in 10 countries | Masson: Single arm of 41 patients for risdiplam | Aged 1-7 months.  Masson: infants had an average age of 5.2 (SD = 1.47) months. CADTH “Patients in FIREFISH Part 1 had a mean age of 5.81 (SD = 1.38) months at enrolment | 22 (54%) female and 19 (46%) males | Type 1 | Two copies | Risdiplam    Orally administered. For those who were unable to swallow it was administered as a bolus through a feeding tube. | Single arm study | EMA:” First Patient Enrolled in Part 1: 23 Dec 2016; Last Patient Enrolled in Part 1: 21 Feb 2018. First Patient Enrolled in Part 2: 13 Mar 2018; Last Patient Enrolled in Part 2: 19 Nov 2018. Clinical cutoff date (CCOD): 14 Nov 2019. Masson: “The 36-month extension period of the study is ongoing | 24 months |
| Freigang 2021 | N/A | Retrospective observational study | Germany | Nine German neurological specialist care centres | one arm- subgroups: CK n=353, Crn n=453 | Mean age 36.2 +- 12.5 (18–71) | Male: 121   Female: 85 | Type 2 and 3 | 2: n=15   3: n=85   4+ n=74   Unknown: n=32 | Nusinersen    intrathecal administration.  Loading doses: Days 0,14, 28, 63 (2 months)   Maintenance doses: 6 months, 10 months, 14 months, 18 months | Nusinersen only | 18 months | 18 months |
| Freigang 2021b | N/A | Prospective, multicentre observational study | Germany | 4 German motor neuron disease specialist care centres (Departments of Neurology in Dresden) | SMA group n=79 (Children, n=21  Adults, n=58)  Healthy controls, n=30 | 31 (17–43) years | SMA group. Male n=38, female n=41  Control group. Male n=12, female n=18 | Type 1, 2 and 3 | 2 copies, n=9  3 copies, n=31  4+ copies, n=18  Unknown, n=21 | Nusinersen    Intrathecal administration. 12mg. Patients received nusinersen treatment according to the prescribing information for up to 14 months. | The control group was age and sex-matched, was not administered with nusinersen | Between 2017 and 2020 | 14 months |
| Freigang 2023 | N/A | Prospective observational study | Germany | Secondary care- Neurology department of one hospital | one arm: n=24 | Mean age: 37.2 years (17-57 years) | Female: 13/24 (54%) Male: 11/24 (46%) | Type 2: 10/24 (42%)   Type 3: 14/24 (58%) | 2 copies: 2/24 (8.3%)   3 copies: 14/24 (58.3%)   4 copies: 8/24 (33.3%) | Nusinersen    Intrathecal administration, Dose administered every four months, after four loading doses in 2 months (days 0, 14. 28, and 63) | Single arm study | 26 months of nusinersen treatment | Follow-up visit 1: n=22, 15.5 months   Follow-up visit 2: n=19, 26.6 months   Follow-up visit 3: n=9, 38.3 months |
| Gaboli 2025 | n/a | Prospective observational study | Spain | Hospital Universitario Virgen del Rocío, Spain | 28 | NR paediatric patients | Majority male (20 out of 28) | Type 1 (11 patients: 6 type 1b, 5 type 1c), Type 2 (12 patients), Type 3 (5 patients) | Type 1: 2 copies (81.8%), 3 copies (18.2%); Types 2 and 3: 2 copies (11.8%), 3 copies (82.4%), 4 copies (5.8%) | Nusinersen  intrathecal | n/a | At least 24 months | 24 months  Timing of assessment:  Baseline, 6, 12 , 24 months |
| Gaffar 2022 | N/A | Nonrandomized, two-part, phase 2-3, open-label interventional clinical trial: dose-escalation, dose-finding study | 5 countries (unspecified) | 7 outpatient centres in 5 countries | Low dose risdiplam: 4   High dose risdiplam: 17 | Between 1 and 7 months old at enrolment | Not reported | Type 1 | 2 copies | Risdiplam    Oral administration, daily. Treatment with risdiplam (low dose 0.08 mg/kg/day vs. high dose 0.2 mg/kg/day) | Two intervention groups:  low dose 0.08 mg/kg/day vs. high dose 0.2 mg/kg/day.  Administration method and frequency are the same in both groups | 14.8 months of treatment and 24 months of follow-up | 24 months from the end of the study |
| García de la Banda 2020 | N/A | Prospective study | France | two referral centres | Nusinersen group, n=12   Control group, n=14 | Mean age 9.4 ± 2.3 | Male n=9   Female n=7 | SMA 1c and 2 | Three copies | Nusinersen    Intrathecal injection. 6 injections | Compared with 14 age-matched historical untreated controls (ration1:1). | January 2019 and June 2019 | 6 months |
| Goedeker 2021 | N/A | Retrospective study | US | Local tertiary care centres | one arm: n=58 | from 1 month to 56 years (median 6.83 years) | Not reported | Type 1-3 | Not reported | Nusinersen    Intrathecal nusinersen administration. Median of 10 doses per patient (range 2–13) | Single arm study | February 2017 and March 2020 | N/A- reviewed laboratory reports |
| Gonski 2023 | N/A | retrospective observational cohort study | Australia | Two tertiary treatment sites | one arm: n=48 | Mean age before treatment: 7.5 years. Mean age at first dose: 6.98 years + SD 5.25 | Male:   Type 1: 5/10 (50%)   Type 2: 11/23 (48%)  Type 3: 9/15 (60%)  Total: 25/48 (52%) | Type 1: 10   Type 2: 23   Type 3: 15 | Not reported | Nusinersen    Not specified in the paper | Single arm study | Participants who initiated treatment with nusinersen between May 2015 and April 2019   Total 4 years: Data was collected two years before the first dose of nusinersen, and during 2 years after starting. | Assessments taken 2 years before and 2 years after treatment started |
| Govoni 2024 | n/a | Multicenter retrospective cohort study | Italy | 18 Italian secondary or tertiary care centers for SMA | 48 | Age at baseline (years): 39.7 ± 13.5 | 56% females | SMA3a (15), SMA3b (33) | 3 copies (10), 4 copies (28), unknown (10) | Nusinersen  Intrathecal | n/a | 14 months | 14 months  Timing of assessment:  Baseline (T0), 6 months (T6), 10 months (T10), 14 months (T14) |
| Gunther 2024 | N/A | Prospective observational study | Germany, Austria, and Switzerland | Not reported | one arm: n=237 | (mean):   14 months: 36 years   26 months: 35 years   38 months: 36 years | Female: 14 months: 102/237 (43%), 26 months: 68/171 (40%), 38 months: 49/120 (41%)   Male: 14 months: 135/237 (57%), 26 months: 103/171 (60%), 38 months: 71/120 (59%) | Type 1: 14 months: 5/237 (2%), 26 months: 3/171 (2%), 38 months: 2/120 (2%)   Type 2: 14 months: 67/237 (28%), 26 months: 44/171 (26%), 38 months: 33/120 (27%)   Type 3: 14 months: 156/237 (66%), 26 months: 117/171 (68%), 38 months: 83/120 (69%0   Type 4: 14 months: 9/237 (4%), 26 months: 7/171 (4%), 38 months: 2/120 (2%) | 2 copies: 14 months: 12/237 (5%), 26 months: 12/171 (7%), 38 months: 8/120 (7%)   3 copies: 14 months: 82/237 (35%), 26 months: 55/171 (32%), 38 months: 36/120 (30%)   4 copies: 14 months: 90/237 (38%), 26 months: 66/171 (39%), 38 months: 45/120 (38%)   5 copies: 14 months: 5/237 (2%), 26 months: 3/171 (2%), 38 months: 1/120 (1%)   6 copies: 14 months: 3/237 (1%), 26 months: 2/171 (1%), 38 months: 1/120 (1%)   7 copies: 14 months: 1/237 (0%), 26 months: 1/171 (0%), 38 months: 1/120 (1%)   Unknown: 14 months: 44/237 (19%), 26 months: 32/171 (19%), 38 months: 28/120 (23%) | Nusinersen    Intrathecally administered. Loading phase, followed by maintenance dose every 4 months | Single arm study | July 2017 to May 2022   Minimum treatment time of 14 months. 171 patients with a treatment period of 26 months, and 120 patients with a treatment period of 38 months | 171 patients with a treatment period of 26 months, and 120 patients with a treatment period of 38 months |
| Hagenacker 2020 | N/A | Observational cohort study | Germany | Ten academic clinical sites | One arm. Included in 6-month analysis (n=124)   Included in 10-month analysis (n=92)   Included in 14-month analysis (n=57) | 16-65 years | Included in 6-month analysis (n=124)   Male n=67   Female n=57   Included in 10-month analysis (n=92)   Male n=53   Female n=39   Included in 14-month analysis (n=57)   Male n=37   Female n=20 | 6-month analysis    SMA1, n=2   SMA2, n=45   SMA3, n=77   SMA4, n=0   10-month analysis    SMA1, n=1   SMA2, n=30   SMA3, n=60   SMA4, n=1   14-month analysis    SMA1, n=0   SMA2, n=20   SMA3, n=37   SMA4, n=0 | 6-month analysis    2 copies, n=7   3 copies, n=48   4 copies, n=41   5 copies, n=2   6 copies, n=2   Unknown, n=24   10-month analysis    2 copies, n=7   3 copies, n=33   4 copies, n=31   5 copies, n=1   6 copies, n=0   Unknown, n=20   14-month analysis    2 copies, n=4   3 copies, n=21   4 copies, n=21   5 copies, n=0   6 copies, n=0   Unknown, n=11 | Nusinersen    Intrathecally. 12mg.  On days 1, 14, 28, and 63, with repeated maintenance injections every 4 months in accordance with the label. | Single arm study | 14 months | Minimum treatment time of 6 months to a follow-up of up to 14 months |
| Hahn 2022 | N/A | Real-world data | Germany | 23 centres, including paediatric and neurologic departments | Single arm study, but grouped into type 1 and type 2 SMA:   Type 1: 31   Type 2: 80 | Type 1: mean age 13.5 years. 52.8% of patients with SMA1 were 5–12 years old   Type 2: mean age 29 years. Included mostly adults across all age groups (range 3–60 years) | SMA 1: 14 female, 17 male/31   SMA 2: 49 female, 31 male/80 | Type 1: 31   Type 2: 80 | Not reported | Risdiplam    Oral administration. (0.75 mg/mL aqueous solution)   0.2 mg/kg risdiplam for patients    between 2 months and 2  years of age, 0.25  mg/kg for    patients > 2 years of age and < 20 kg, and a fixed dose of    5 mg for patients > 2 years of age and ≥ 20 kg | Single arm study | 15 months | Not clear. The mean duration of risdiplam treatment was 5 months in the type 1 group and 3.4 months in the type 2 group |
| Huang 2024 | n/a | Prospective observational study | China | Children's Hospital of Zhejiang University School of Medicine | One arm: 49 | Under 18 years old | Male n=28 (57.1%), Female n=21 (42.9%) | SMA2 n=33 (67.3%), SMA3 n=16 (32.7%) | 2 copies n=2 (4.1%), 3 copies n=44 (89.8%), 4 copies n=3 (6.1%) | Nusinersen  Intrathecal  Loading doses at 0 (baseline), 14, 28, and 63 days, followed by maintenance administration every 4 months | n/a | February 2022 to April 2024 | 18 months  Timing of assessment:  Baseline, 6, 14, and 18 months |
| Ip 2024 | N/A | Prospective longitudinal study | Hong Kong | Two SMA treatment institutes in Hong Kong: the Queen Mary Hospital, and the Hong Kong Children’s Hospital | one arm: n=23 | Mean age at starting nusinersen: 6.7 years | Male:   Type 1: 3/8 (38%)   Type 2: 3/7 (43%)   Type 3: 4/8 (50%)   Total: 10/23 (43% | Type 1: 8   Type 2: 7   Type 3: 8 | 2 copies: type 1: 5/8 (63%), type 2: 0, type 3: 0, total: 5 (22%)   3 copies: type 1: 3/8 (38%), type 2: 7/7 (100%), type 3: 4/8 (50%), total: 14/23 (61%)   4 copies: type 1: 0, type 2: 0, type 3: 4/8 (50%), total: 4/23 (17%) | Nusinersen    Intrathecal administration. 12mg.  Four loading doses were given on day 1, half a month, one month, and two months post-first dose.   Then maintenance dose every four months thereafter | Single arm study | 2018-2023 | Motor function evaluations were taken at baseline and at 6- and 12-month intervals after starting treatment.   Median follow-up of 3.4 years |
| JEWELFISH (NCT03032172) 2023 (Chiriboga 2023) | Chiriboga  2023 1,2,3,4,5,6 | exploratory, noncomparative, open-label study | Belgium, France, Germany, Italy, the Netherlands, Poland, Switzerland, the UK, and the US | 24 different centres in Belgium, France, Germany, Italy, the Netherlands, Poland, Switzerland, the UK, and the US | Based on previous treatments that patients received:   Olesoxime (n = 70)   MOONFISH study (“3 patients who were previously enrolled in the MOONFISH study (NCT02240355) received placebo treatment and were never switched to RG7800”) (n = 13)   nusinersen (n = 76)   onasemnogene abeparvovec(n = 14)   All patients were 173 (“1 patient withdrew from the study at baseline and therefore 173 patients received risdiplam”) | age at enrolment was 6 months-60 years | 55% male and 45% female | 62% SMA type 2 | The majority of patients (78%) had three SMN2 copies, with 13% of patients having four copies, and 7% who had 1–2 copies; the remainder were pending genotyping and were classified as unknown | Switching to Risdiplam    Orally administered. All patients received risdiplam once daily at the approved dosing regimen as follows: 0.2 mg/kg for infants aged 6 months to less than 2 years, 0.25 mg/kg for patients older than 2 years with a body weight less than 20 kg, and 5 mg for patients with a body weight of 20 kg or more | Previous treatment received | “The remaining patients enrolled before dose selection and, therefore, received 3 mg at the start of the study for a mean duration of 203 days (118–277 days) which was increased to 5 mg after dose confirmation from the SUNFISH trial (NCT02908685)”   “As of the CCOD, the mean duration (standard deviation) of exposure to risdiplam was 17.0 (7.1) months (0.9–47.0)" | 24 months |
| Jiang 2024 | PANDA (NCT04419233) | Prospective, 2-year, multicenter post-marketing surveillance (PMS) study | China | Multiple centers in China | One arm: 50 | Overall: median age 4.6 years (range 1-21)  Infantile-onset group: median age 2.6 years (range 1–5), later-onset group: median age 6.6 years (range 1–21) | Male n=22 (44%), Female n=28 (56%) | Infantile-onset (10), later-onset (40) | 2 copies n= 7, 3 copies (32 participants), 4 copies n=2, 5 copies n=1, missing data (8 participants) | Nusinersen Intrathecal  12 mg per administration (5 ml doses)  Four loading doses on Days 0, 14, 28, and 63, and maintenance doses once every 4 months thereafter | n/a | 2 years | 2 years  Timing of assessment:  At each dose visit |
| Jiang 2024b | n/a | Retrospective analysis of adverse event (AE) reports from FAERS database | United States: 4367 reports (75.66%)  Brazil: 141 reports (2.44%)  Turkey: 119 reports (2.06%)  Argentina: 97 reports (1.68%)  China: 85 reports (1.47%) | FAERS database | FAERS database collected 11,485,105 AE reports, among which 5772 were related to Nusinersen | <18 years n=2167 (37.54%), 18–45 years n=857 (14.85%), 45–65 years n=272 (4.71%), 65–75 years n=43 (0.74%), ≥75 years n=7 (0.12%), unknown n=2426(42.03%) | Female n=2657 (46.03%), Male n=2595 (44.96%), Unknown n=520 (9.01%) | n/a | n/a | Nusinersen  intrathecal | n/a | First quarter of 2017 to third quarter of 2023 | n/a |
| Kariyawasam 2021 | N/A | Single centre prospective cross-sectional and longitudinal study | Australia | Sydney Children’s Hospital New South Wales, Australia | one arm: n=20 | Median 12, (2–144) | Male, n=11   Female, n=9 | SMA1, n=6   SMA2, n=10   SMA3, n=4 | 2 copies, n=3   3 copies, n=16 | Nusinersen    Intrathecal nusinersen | Single arm study | Between June 2017 and October 2019 | Median 13.8 months (4–33.5) |
| Kokaliaris 2024 | NCT02913482, NCT02193074, NCT02594124 | Indirect treatment comparison (ITC) using unanchored matching-adjusted indirect comparison (MAIC) | n/a | Data from FIREFISH, ENDEAR, and SHINE clinical trials | 58 children in FIREFISH, 81 children in SHINE-ENDEAR | Children aged 1–7 months at enrollment | (Female), n (%):  SHINE-ENDEAR: 43 (53.8%)  FIREFISH: 33 (56.9%)  Difference: -3.1%  Post-Matching FIREFISH: -66.7%  Difference: -12.9% | Type 1 SMA | Patients with Two SMN2 Copies, n (%):  SHINE-ENDEAR: 80 (100%)  FIREFISH: 58 (100%) | Risdiplam (FIREFISH), Nusinersen (SHINE-ENDEAR)  Risdiplam (oral), Nusinersen (intrathecal) | Nusinersen | At least 36 months of follow-up | At least 36 months of follow-up  Scheduled visits |
| Kolbel 2022 | N/A | Questionnaire | Germany | Not reported | One arm: n=43 | Not reported at baseline | Not reported | Suspected to develop the clinical phenotype of SMA type 1 or 2 if left untreated | 2 copies, n=17   3 copies, n=10   ≥4 copies, n=16 | Nusinersen    Not reported | Nusinersen | January 2018 to February 2020 | Not reported |
| Konersman 2021 | N/A | Retrospective chart review | US | Rady Children’s Hospital and the University of California, San Diego | one arm: n=35 | 5–58- years | 51.4% females (n=18) | SMA 1 n=4   SMA 2 n= 14   SMA 3 n=13 | 1 copy n=2   2 copies n=5   3 copies n=21   4 copies n=3   4 copies or more n=4 | Nusinersen    Lumbar puncture. First three doses every 2 weeks, 4th dose 30 days after 3rd dose, followed by maintenance doses every four months thereafter | Single arm study | April 2017-June 2019, 22-month study period | Study period with nusinersen from April 2017-June 2019 |
| Kotulska 2022 | N/A | Patients prospectively followed | Poland | 16 paediatric sites in Poland | One arm: n=298 | Mean age: 6.9 years | Male: 156/292 (53%)   Female: 136/292 (47%) | Presymptomatic: 4 (1.4%)   Type 1: 127 (43.5%)   Type 2: 68 (23.3%)   Type 3: 93 (31.8%) | 1 copy: 1 (0.3%)   2 copies: 88 (30.1%)  3 copies: 158 (54.1%)   4 copies: 40 (13.7%)   5 copies: 3 (1%)   6 copies: 2 (0.7%) | Nusinersen    Intrathecally, 12mg. Loading dose on days 1, 15, 29 and 57, followed by a maintenance dose of 12mg every four months | Single arm study | The data of patients enrolled in the program between March 1 and September 20, 2019, were included, plus a one-year follow-up | Minimum 1-year follow-up |
| Krosmark 2023 | N/A | Prospective study | Sweden | Queen Silvia Children's Hospital, Gothenburg, Sweden | Nusinersen: 10, no nusinersen: 10 | Mean age at baseline: 9.1 years (2.4-17.6), mean age at follow-up: 10.5 years (4.4-18.9), Mean age in the nusinersen-treated group: 6.7 years (2.4 - 17.0).   Mean age in the nusinersen-treated group: 6.7 years (2.4 - 17.0).  Mean age in the untreated group: 10.8 years (4.2 to 15.3). | Not reported | SMA type 2: 11. SMA type 3: 9 | All patients with type 2 had 3 SMN2 copies     3 patients with type 3 had 3 SMN copies and 6 had 4 SMN copies | Nusinersen    Method of administration:  Intrathecally administered   Dose:   12mg (5mL)  Frequency:  First three loading doses were administered at 14-day intervals, final loading dose was 30 days after the third dose. Maintenance doses are given every 4 months after loading doses are finished. | No formal comparator, but when nusinersen was approved, the patients were started on treatment, from youngest first. At baseline, none of the patients were treated with nusinersen,    By follow-up, 10 had started treatment | 2 years | 2 years |
| Krupa 2023 | N/A | Real-world data | Poland | Polish healthcare system | 2014: 45    2019: 51   Results examined together as the total treated population | Total treated population:   Mean age:   Males: 21.7 years  Females: 21.3 years | Of total population:   Male: 53%   Female: 47% | Total treated population:   SMA 1: 218   SMA 2: 211   SMA 3: 426   Presymptomatic: 20 | Not reported | Nusinersen    Not reported by the paper | No comparator, although the group with later treatment (2014 group) are examined separately in regard to time to death or mechanical ventilation | The overall study covered 2014- 2022. One cohort started in 2014, other in 2019 | 2014-2022, 2019-2022.   Assessments taken every year (2019, 2020, 2021, first five months of 2022) |
| Kwon 2022 | NCT04256265 | Expanded Access Program (EAP) | United States | 23 non-preselected sites across 17 US states | 155 enrolled, 149 completed the EAP | Median age 11 years (range 4 months–50 years) | 73 (47.1%) males, 82 females (52.9%) | Type 1 n=73 (47.1%), Type 2 n=82 (52.9%) | 2 copies n=67 (43.2%), 3 copies n=75 (48.4%), 4 copies n=3 (1.9%), missing n=10 (6.5%) | Risdiplam  Oral  Dose:  Patients aged 2 months to <2 years received 0.20 mg/kg/day, patients aged ≥2 years (<20 kg) received 0.25 mg/kg/day, and patients aged ≥2 years (≥20 kg) received 5 mg/day  Frequency:  Once daily | n/a | April 2020 to May 2021  Median treatment duration 4.8 months (range 0.3–9.2 months) | Median treatment duration 4.8 months (range 0.3–9.2 months)  Timing of assessment:  Every 3 months for patients <2 years old and/or <20 kg, every 6 months for patients ≥2 years old and ≥20 kg |
| Landfeldt 2023 | N/A | Prospective cohort study | Sweden | Electronic questionnaire- remote | One arm: n= 27 | Mean age: 9.17 years (2.55- 17.63 years) | 59% female | Type 1: 26%, Type 2: 33%, Type 3: 41% | Not reported | Nusinersen and risdiplam    Nusinersen was administered with intrathecal injection and Risdiplam orally. | Single arm study | September 2020 to April 2022 | Baseline, 6, 12 and 18 months |
| Lavie 2021 | N/A | Descriptive study | Israel | SMA specialised referral centre in Tel Aviv( Sour sky Medical Centre) | one arm: n=20 | median age was 13.5 months | Females: 12/20   Males: 8/20 | Type 1 | Data available for 14 patients:   2 copies: 13   3 copies: 1 | Nusinersen    12mg (5ml). Four loading doses, followed by maintenance therapy every four months | Single arm study | 3 years | 3 years |
| Lee 2022 | N/A | Retrospective study | US | Neuromuscular Specialty Care Centre. | None, n=2   onasemnogene alone, n=23   nusinersen alone, n=1   risdiplam alone, n=1   nusinersen bridge to onasemnogene, n=5   risdiplam after initial treatment with onasemnogene, n=2 | Median = 34.5 days of life; range 11–180 days | Not reported | Not stated specifically.    “Early observations of prenatally identified infants with SMA type 1” | 1 copy, n=1   2 copies, n=18   3 copies, n=11   ≥ 4 SMN2 copies, n=4 | None, n=2   onasemnogene alone, n=23   nusinersen alone, n=1   risdiplam alone, n=1   nusinersen bridge to onasemnogene, n=5   risdiplam after initial treatment with onasemnogene, n=2    Not reported | Onasemnogene alone,   nusinersen alone,   Risdiplam alone,   nusinersen bridge to onasemnogene,   risdiplam after initial treatment with onasemnogene   Methods of administration, type, dose, frequency not stated | 3 years | 3 years |
| Lee 2023 | N/A | Pre-post intervention analysis | South Korea | Secondary care- two University hospitals | one arm: n=24 | The median age of patients at treatment onset was 148.8 (6.8-269.4) months | SMA type 2: 7/19 (36.8%)   SMA type 3: 2/5 (40%)   Total: 9/24 (37.5%) | SMA type 2: 19   SMA type 3: 5 | The paper suggests copies are recorded, but none reported | Nusinersen    Injection. Loading doses were administered on days 0, 14, 28, and 63, followed by maintenance dosing once every 4 months thereafter | Single arm study | 26 months of treatment | Outcomes were assessed at the time of the fourth loading dose, and every four months after, until the time of the sixth maintenance dose. |
| Lemska 2024 | n/a | Prospective observational single-center study | Poland | Single center in Poland | 23: 6 SMN2 3 Copies and 17 SMN2 2 Copies | Average age at start of therapy: 31.09 months for 3 copies, 31.24 for 2 copies. 31.09 combined | 9 females, 14 males | 2 patients with SMA type 3, 5 patients with SMA type 2, 16 patients with SMA type 1. | 2 copies (17 patients), 3 copies (6 patients) | Nusinersen  Intrathecal  12 mg per patient  Loading doses on days 1, 14, 28, and 63, followed by maintenance doses every four months | n/a | January 2019 to July 2024 | 22 months  Timing of assessment:  Routine patient visits |
| Li 2024 | N/A | Single-centre retrospective case-control study | China | Second Affiliated Hospital of Xi’an Jiaotong University | SMA, n=42   Healthy control, n=42 | 57 months (29.5 to 96 months) | Male: 24  female: 18 | Type 2, n=32   Type 3, n=10 | 2 copies, n=5   3 copies, n=36   4 copies, n=1 | Nusinersen    Intrathecal injection. 12mg.  The loading phase was completed over the first two months, consisting of a total of four intrathecal injections, each with a dose of 12 mg. Subsequently, the maintenance phase followed, with a dose of 12 mg administered every four months. This treatment cycle was continued accordingly | Healthy controls | January 2021 to December 2022 | Up to 14 months |
| Li 2024a | n/a | Retrospective clinical observational cohort study | China | Two centers in western China (Second Affiliated Hospital of Xi’an Jiaotong University and Second Hospital of West China of Sichuan University | Total Patients: 84  SMA1: 9 (10.7%)  SMA2: 57 (67.9%)  SMA3: 18 (21.4%) | Age at Baseline (years, min–max):  SMA1: 4.64 (0.33–12)  SMA2: 6.52 (0.17–16.83)  SMA3: 7.10 (2–17)  Total: 6.47 (0.17–17) | 43 males, 41 females  Male/Female:  SMA1: 3/6  SMA2: 34/23  SMA3: 6/12  Total: 43/41 | Type 1 n=9 (10.7%), Type 2 n=57 (67.9%), Type 3 n=18 (21.4%) | 2 copies n=11 (13.1%), 3 copies n=72 (85.7%), 4 copies n=1 (1.2%) | Nusinersen  Intrathecal via the L3–4 or L2–3 intervertebral space  12 mg per patient  Loading dose in the first 2 months (Days 1, 14, 28, 63), maintenance phase every 4 months | n/a | October 2019 to February 2022 | 14 months  Timing of assessment:  Before each nusinersen injection |
| Li 2024b | n/a | Retrospective pharmacovigilance study | United States, Brazil, Turkey, Germany, Japan, others | FAERS database | 4807 cases found in FAERS database | <18 years n=1916 (39.86%), 18-44 years n=659 (13.71%), 45-64 years n=223 (4.64%), 65-74 years n=27 (0.56%), ≥75 years n=4 (0.08%), unknown n=1978 (41.15%) | Female n= 2229 (46.37%), Male n=2196 (45.68%), Unknown n=382 (7.95%) | n/a | n/a | nusinersen | n/a | December 2016 to March 2023 | n/a |
| Lillien 2024 | n/a | Non-commercial multicenter prospective study | Belgium, Hungary, United States of America, Czech Republic | Six centers in four countries (Belgium, Hungary, USA, Czech Republic) | 99  Clinically significant improvement (N = 41), non-clinically significant improvement (N = 18), no improvement (N = 40) | Median age of 11.2 (0.39–57.4) years at treatment initiation | 51.5% male of 99 patients in total | Type I–III | 2 copies (9%), 3 copies (62%), 4 copies (20%), >4 copies (3%), unknown (5%) | Nusinersen  intrathecal | n/a | 15 months | 15 months  Timing of assessment:  15 months after treatment initiation |
| Lusakowska 2023 | N/A | Real-world study | Poland | 2 centres treating adults and children over 5 years | one arm: n=120 | Age at baseline (years, mean): all: 32, type 1c: 29, type 2: 24, type 3: 34 | Female: all: 53 (44%), type 1c: 4 (33%), type 2: 11 (58%), type 3: 38 (43). Male: all: 67 (56%), type 1c: 8 (67), type 2: 8 (42%), type 3: 51 (57%) | Type 1c (n=12; 10%), type 2 (n=19; 16%), type 3 (n=89;74%) | 2 copies: 4 (3%), 3 copies: 71 (59%), 4 copies: 43 (36), >4: 2 (2) | Nusinersen    Lumbar puncture. 12mg. Loading dose of 12mg at days 1, 14, 28 and 63, followed by maintenance doses every 4 months (from months 6-30) | Single arm study | Mean treatment duration: 23 months | 30 months |
| Maggi 2020 | N/A | Retrospective cohort study | Italy | 18 Italian secondary or tertiary care centres | All, n=116   SMA2, n=13   SMA3 “sitters”, n=51   SMA3, “walkers”, n=52 | Median age at first administration 34 years | All SMA: male n=68, female n=n=48 | Type 2 and 3 | Two copies, n=5   Three copies, n=36   Four copies, n=54   Unknown, n=21 | Nusinersen    Intrathecal injection. 12mg. Loading doses of 12 mg nusinersen at baseline (T0), day 14, day 28 and day 63, followed by maintenance doses every 4 months: T6 at 6 months from T0 (n=116 patients), T10 at 10 months (n=84 patients) and T14 at 14 months (n=54 patients | Nusinersen only | 6 months | 6 months |
| Mendonca 2021 | N/A | Longitudinal and observational study | Brazil | Centre in Brazil (Neuromuscular Clinic at Hospital das Clinicas, FMUSP). | one arm: n=21 | Mean: 34.1 (± 36.0) months | 11 males   10 females | Type 1 | Two or three copies of SMN2  2, n=18  3, n=3 | Nusinersen    Intrathecally injected. 12mg.  on days 1, 14, 28, and 63, with repeated maintenance injections every 4 months | Single arm study | October 2017 to July 2019 | 6-24 months |
| Mendonca 2021b | N/A | Longitudinal and observational study, with a control group | Brazil | Centre in Brazil (Neuromuscular Clinic at Hospital das Clinicas) | Nusinersen n=30   Control n=37 | Nusinersen n=30, age: mean 10.6 years   Control n=37, age: mean 10.2 years | Nusinersen n=30, males: 15 (50%)   Control n=37, males: 19 (51.4%) | Type 2 and 3:   Type 2: n=14 (group 1), n=20 (control)  Type 3: n=16 (group 1), n=17 (control) | 2 copies:   nusinersen n=30, 3/30 (10%)   Control n=37, 0/37   3 copies:   nusinersen n=30, 25/30 (83.3%)   Control n=37, 32/37 (86.5%)   4 copies:   nusinersen n=30, 2/30 (6.7%)   Control n=37, 5/37 (13.5%) | Nusinersen    On days 1, 14, 28 and 63, all patients received12 mg of nusinersen, administered intrathecally, with repeated maintenance injections every 4 months in accordance with the label instructions | Control group | July 2017 to December 2019 | 12 and 24 months |
| Meyer 2021 | N/A | Prospective, non-interventional observational study | Germany | Nine SMA centres in Germany | One arm: n=151 | At symptom onset, mean (SD; range): 5.30 (7.32; 0–42.17) | Male n=85   Female=64 | SMA type 1 n=10   SMA type 2 n=51   SMA type 3 n=90 | Not reported | Nusinersen    manual lumbar puncture | Single arm study | 19.06 months | non-interventional observational study: no follow-up |
| Mirea 2021 | N/A | observational study | Romania | National University Centre for Paediatric Neurorehabilitation | Nusinersen n=6   Combination therapy n=7 | 2-6 months | Female:   nusinersen n= 3    combination therapy n= 3 | Type 1 | 2 copies | Nusinersen    Not reported | Combination therapy (n=6 nusinersen and then onasemnogene abeparvovec-xioi; n=1 onasemnogene abeparvovec-xioi and then Nusinersen) | July 2019 to May 2021 | 24 months |
| Mirea 2022 | N/A | Retrospective observational study | Romania | National Teaching Centre for Children’s Neuro-psychomotor Rehabilitation | Those that received daily PT in addition to nusinersen (PT-N group): 39   Nusinersen only group (N group): 16 | Participants were aged 0-17 years | Female: 29/55, Male: 26/55 | Type 1: 20   Type 2: 26   Type 3: 9 | Type 1 and 2: 2 copies   Type 3: 3 copies | Nusinersen    Intrathecal administration.  The first four loading doses are given in the first two months, followed by one injection every four months for life | As not all patients could attend daily PT- due to location, the sample was split into two groups: nusinersen and PT (PT-N) compared to nusinersen only (N group)    nusineren was administered the same in both groups- the addition of PT in the PT-N group | 12 months | T0: baseline evaluation, T1: 6 months after treatment, T2: 12 months after treatment |
| Modrzejewska 2021 | N/A | Prospective, longitudinal study | Poland | Three early access programme (EAP) centres  that participated in the expanded access    programme in Poland | one arm: n=26 | Mean, 4.79 years  Median, 3.58 (2-15) | Male n=13   Female n=13 | Type 1 | 2 copies, n=16   3 copies, n=9   4 copies, n=1 | Nusinersen    Intrathecal administration. 12mg. on days 1, 15, 30, 60 and thereafter every four months | Single arm study | 18–26 months of treatment | 18-26 months |
| Moshe-Lilie 2020 | N/A | Retrospective study reviewing patient charts | Not reported | Neuromuscular Clinic | one arm: n=22 | Median age was 36 years (range 20–71) | Male, n=7   Female, n=15 | Type 2, n=9   Type 3, n=13 | 3 copies, n=13   4 copies, n=5   Unknown, n=4 | Nusinersen    Intrathecal administration. | Single arm study | Between 2017 and 2019 | 24 months |
| NURTURE (NCT02386553) (Crawford 2023) | Crawford et al. 2023, De Vivo et al. 2019, CADTH nusinersen 2018_clinical, CADTH Nusinersen resubmission 2019_clinical, EMA EPAR spinraza 2017, ICER spinraza and zolgensma 2019, SMC nusinersen 2018 and TA588 committee papers 2018_incl CS and ERG report | Phase 2, open-label, single-arm, multinational study | Centres across 10 countries in Argentina, Australia, Germany, Israel, Italy, Qatar, Taiwan, Turkey, UK, US | Secondary care | One arm: n=25 | The average age at enrolment was 20.5 days   2019: 3-year follow-up: The median (range) age at first dose of nusinersen among enrolled infants was 22.0 (3–42) days. At the time of the interim analysis, the median (range) age at the last visit was 34.8 (25.7–45.4) months and the time on study was 33.9 (25.3–45 months).    2023: further 2-year follow-up: The median (range) age was 4.9 (3.8–5.5) y as of the last visit | Two SMN2 copies: f n=7, m n=8   Three SMN2 copies: f n= 6, m n=4   52% female | were considered most likely to develop SMA Type I or Type II based upon the SMN2 gene copy   number and the expected concordance with the phenotype of an affected sibling(s) based on previous studies | Two or three copies: 65% had 2 SMN2 copies  SMN2 Copies = 15  SMN3 copies = 10 | Nusinersen    intrathecal injections by lumbar puncture. 12mg   The nusinersen treatment regimen consists of four loading doses (administered on Days 1, 15, 29, and 64), followed by a maintenance dose every 119 days over five years | Single arm study | NURTURE consists of a 5-year treatment period and a post-treatment follow-up evaluation.   Interim analysis reports data from the February 15, 2021, data cut with data reflecting the last visit most proximal to this date.   Median (range) time on study of 4.9 (3.9–5.7) y. The median (range) age was 4.9 (3.8–5.5) y as of last visit | Three years (De Vivo et al. 2019) and further two years (Crawford et al. 2023) |
| Okamoto 2020 | N/A | Questionnaire | Japan | 131 hospitals on Shikoku with paediatrics or neurology departments | one arm: n=21 | age in years:  0-4. n=6   5-9. n=3   10-14, n=2   15-19, n=3   20-29, n=1   30-39, n=1   40-49, n=2   50-59, n=2   60-69, n=1 | Male, n=12   Female, n=9 | homozygous deletion of SMN1, n=16   SMA type 1, n=21   SMA type 2, n=5   SMA type 3, n=5   SMA type 4, n=1   Unidentified, n=1 | 2 copies, n=6   3 copies, n=6 | Nusinersen    Not reported | Single arm study | 2-12 months. Between March and September 2019 | Not reported |
| Osmanovic 2021 | N/A | Single centre, Questionnaire | Germany | Department of Neurology, Hannover Medical School | One arm: n=28 | Adult   Mean 39.18; Median 36.0 years | Women n=13 (46.4%) | 2, 3 and 4   Type 2 n=13   Type 3 n=14   Type 4 n=1 | 2 - 6 copies   Median = 3 | Nusinersen    Maintenance phase | Single arm study | May 2020 to January 2021 | Not reported |
| Osmanovic 2021b | N/A | Observational   Cross-sectional   multi-Centre | Germany | Neuromuscular centres (x5) | One arm; n=91 | SMA patients aged 10–65 (mean 35.4 years)   Adolescent (n=6, <18years)  Adult (n=85, ⩾18years) | Female 36.3% | 2, 3 and 4   Type 2 36.3%   Type 3 59.3% | copy numbers 2-6   (n=12 unknown) | Nusinersen    Intrathecal. 12mg, 5ml.  Days 0, 14, 28 and 63 with subsequent maintenance administrations every 4 months | Single arm study | January 2019 and September 2019 | 2 months |
| Osredkar 2021 | N/A | Prospective, two-centre study | Slovenia and Czech Republic | Department of Child, Adolescent and Developmental Neurology at the University Children's Hospital Ljubljana, Slovenia (SI centre) or at the Department of Paediatric Neurology, Motol University Hospital, Prague, Czech Republic | Subgroups rather than arms: SMA type 1, n=16   SMA type 2, n=32   SMA type 3, n=13 | Median age (range in years), 8.6 (0.2e18.8) | Male, n=32   Female, n=29 | Type 1, n=16   Type 2, n=32   Type 3, n=13 | 2 copies, n=11   3 copies, n=38   4 copies,n=12 | Nusinersen    Intrathecal application.  5 ml (12 mg/ml), or appropriately reduced dose in smaller children, accordingly to the   company's recommendations.     Injected intrathecally on days 0, 14, 30, 60, 180, 300 and 420 in all patients | Single arm study | 14 months | 6 and 14 months |
| Pandey 2025 | n/a | Prospective parallel group non-randomized concurrent cohort study | North India | Pediatric Neurology Unit of a tertiary care research and referral institute of North India | 42 in the SMA groups: 16 in the Nusinersen group, 26 in the SOC group  72 in natural history group | Mean age of 85 ± 55 months | 62% boys | Type 2 (40%) and Type 3 (60%) | ≥2 | Nusinersen  Intrathecal  12 mg for intra-thecal administration (5 mL per administration)  4 loading doses (first three at 14-day intervals, 4th dose 30 days after the 3rd), then maintenance doses once every 4 months | Standard of care (SOC) | January 2022 to December 2023 | 12 months |
| Pane 2019 | N/A | Survey | Italy | Italian centres for trials | one arm: n=85 | 2 months to 15 years and 11 months | Not reported | Type 1 | 1 copy, n=2   2 copies, n=61   3 copies, n=18   Unavailable, n=4 | Nusinersen    Not reported | Single arm study | 13 months | 12 months |
| Pane 2020 | N/A | Prospective study | Italy | Five Italian centres previously involved in nusinersen trials | CHOP-INTEND test   non-sitter, n=47   Sitter, n=21   HINE – II test   non-sitter, n=47   Sitter, n=21   Whole cohort, sitter whole cohort, n=68 | 0.20 to 15.92 years (mean:3.96) | Not reported | Type 1 | Two patients had 1 SMN2 copy, 48 had 2, 17 had 3, and 1 had 4 | Nusinersen    11 doses, details regarding concentration and volume not stated | Nusinersen only | 2 years | 2 years |
| Pane 2022 | N/A | Real-world data | Italy | Italian centres participating in International SMA Registry | 111 patients with SMA   Unknown sample size of untreated patients | All: 12.51 years   Type 2: 6.66 years   Type 3: 17.86 years | All: male: 60/11 (54.05%), female: 51/111 (45.95%)   Type 2: male: 25/46 (54.35%), female: 21/46 (45.65%)   Type 3: male: 35/65 (53.85%), female: 30/65 (46.15%) | Type 2: 46   Type 3: 65 | Not reported | Nusinersen    Not reported | Compared to untreated group but no information provided about characteristics of comparator | 24 months | 24 months. Mean duration of follow-up: 2.56 years |
| Pane 2023 | N/A | Recruited as part of early access programme (EAP). | Italy | Recruited as part of EAP | One arm: n=48 | Mean age at baseline 3.3 years | Not reported | Type 1 | 1 copy: 1/48 (2.1%)   2 copies: 33/48 (68.7%)   3 copies: 14/48 (29.2%) | Nusinersen    Not reported by paper | Single arm study | 4 years | 4 year (results from 2 years previously reported) |
| Pane 2024 | n/a | Prospective study | Italy | Nation-wide registry | Whole cohort (N = 75) No need for tube  feeding (N = 34)  Tube feeding and  oral (N = 9)  Tube feeding  without oral  (N = 32) | 0.1 to 5 years, mean age 1.34 (years) | 38 females and 37 males | Type 1  Type 1.1: 13 (17%)  Type 1.5: 41 (55%)  Type 1.9: 11 (21%) | 1 copy: 1 patient  2 copies: 63 patients  3 copies: 11 patients | Nusinersen: 69 patients  Onasemnogene Abeparvovec only: 6 patients | n/a | At least 12 months follow-up | Regular follow-ups at least every 6 months  Follow-up duration after treatment:1 to 7.7 years  Timing of assessment:  All patients were followed regularly at least every 6 months  and information on nutritional changes were recorded; baseline |
| Pechmann 2022 | N/A | Prospective study of SmartCare registry | Germany, Austria and Switzerland | 34 neuropediatric or neurological departments | One arm total at baseline: 256   Subgroups:  Younger sitters: 107   Older sitters: 73   Lost sitters: 37   Lost walkers: 39 | (Younger sitters were younger at start of treatment than in the other three cohorts)   Age at start of treatment (baseline):   Younger sitters: 30.8 months   Older sitters: 120.9 months   Lost sitters: 98.5 months   Lost walkers: 134.7 months | Not reported | Type 2 included (217):   Younger sitters: all patients with type 2, 5 years or younger   Older sitters: > 5 years   Lost sitters: all children with type 2   Type 3 included (n= 39):   Lost walkers:   Children with type 3 | 1 copy: younger sitters: 0, older sitters: 1/73 (1.4%), lost sitters: 0, lost walkers: 0   2 copies: younger sitters: 11/107 (10.3%), older sitters: 5/73 (6.8%), lost sitters: 2/37 (5.4%), lost walkers: 0   3 copies: younger sitters: 78/107 (72.9%), older sitters: 37/73 (50.7%), lost sitters: 23/37 (62.2%), lost walkers: 26/39 (66.7%)   4 or more copies: younger sitters: 12/107 (11.2%), older sitters: 11/73 (15.1%), lost sitters: 3/37 (8.1%), lost walkers: 6/39 (15.4%)   Unknown: younger sitter: 6/107 (5.6%), older sitters: 19/73 (26%), lost sitter: 9/37 (24.3%), lost walker: 7/39 (17.9%) | Nusinersen    Not reported by paper | Single arm study | 38 months | Maximum follow-up period of 38 months   Younger sitters: baseline: 107, m14: 87, m26: 74, m38: 52   Older sitters: baseline: 73, m14: 69, m26: 55, m38: 40   Lost sitters: baseline: 37, m14: 33, m26: 27, m38: 15   Lost walkers: baseline: 39, m14: 36, m26: 32, m38: 22 |
| Pechmann 2023 | N/A | SmartCare registry of longitudinal data used | Germany and Austria | SmartCare registry data on 28 neuropediatric departments of adult and paediatric centres | 2 cohorts: cohort 1a (n=88) and cohort 1b (n=55) | Mean age at treatment start:   Cohort 1a: 8.4 months   Cohort 1b: 89.8 months | Not reported | Not reported | 1 copy: Cohort 1a: 1/88 (1.1%), Cohort 1b: 1/55 (1.8%)   2 copies: cohort 1a: 64/88 (72.7%), cohort 1b: 18/55 (32.7%)  3 copies: cohort 1a:, 17/88 (19.3%), cohort 1b: 20/55 (36.3%)  4 or more copies: cohort 1a: 1/88 (1.1%), cohort 1b: 3/55 (5.5%)   Unknown: cohort 1a: 5/88 (5.7%), cohort 1b: 13/55 (23.6%) | Nusinersen    Intrathecal administration. | Cohort 1 and cohort 2 | 38 months | Max. 38 months Mean follow-up time cohort 1a: 12.3 months, cohort 1b: 27.6 months |
| Pechmann b 2023 | N/A | Analysis of data on SmartCare registry | Germany, Austria and Switzerland | 58 centres in Germany, Austria and Switzerland using SMArtCARE registry | Not different arms, but different subgroups:   Paediatric walkers: 114  Adult walkers: 117 | Age at treatment initiation (mean):   Paediatric walker: 103.3 months   Adult walker: 443.5 months | Not reported | Any type- Did not include type 3 who had lost ambulation | 1 copy: paediatric walker: 0, adult walker: 2/117 (1.7%)   2 copies: paediatric walker: 10/114 (8.8%), adult walker: 4/117 (3.4%)   3 copies: paediatric walker: 35/114 (30.7%), adult walker: 24/117 (20.5%)  4 or more copies: paediatric walker: 57/114 (50%), adult walker: 67/117 (57.3%)   Unknown: paediatric walker: 12/114 (10.5%), adult walker: 20/117 (17.1%) | Nusinersen    Not reported by paper | Not different arms, but different subgroups | 28 months | Up to 38 months follow-up |
| Peng 2025 | n/a | single-center, prospective, observational cohort study | China | Recruited from the Department of Neurology of the Children’s Hospital of Chongqing Medical University | 55 (8 type 1, 36 type 2, 11 type 3) enrolled but 41 patients with at least 6 months of nusinersen treatment analysed | aged 0–18 years | Type 1: 1 female (25.0%)  Type 2: 15 females (50.0%)  Type 3: 2 females (28.6%)  All: 18 females (43.9%) | SMA (type 1–3)  Type 1: 4  Type 2: 30  Type 3: 7 | 2 Copies:  Type 1: 1/3 (33.3%)  Type 2: 1/21 (4.8%)  Type 3: 0 (0.0%)  All: 2/31 (6.5%)  3 Copies:  Type 1: 2/3 (66.7%)  Type 2: 20/21 (95.2%)  Type 3: 6/7 (85.7%)  All: 28/31 (90.3%)  4 Copies:  Type 1: 0 (0.0%)  Type 2: 0 (0.0%)  Type 3: 1/7 (14.3%)  All: 1/31 (3.2%) | nusinersen  intrathecal  intrathecal dose of nusinersen (5 ml/12 mg per dose) at 0, 14, 28, and 63 days and then every 4 months thereafter by a professional neurologist. | n/a | January 2019 to December 2023  At least 14 months | Baseline, 6,10 and14 months |
| Pera 2021 | N/A | Longitudinal study | Italy | The data used in this study were collected from the International SMA Registry, no administration of the drug in a clinical setting. | Type 3A n=74, type 3B n=70 | 30 months–68.27 years | 84 male, 60 female | Type 3 | 1: 0    2: 11 (4 IIIA, 7 IIIB)   3: 56 (40 IIIA, 16 IIIB)   4: 14 (8 IIIA, 6 IIIB)   4+: 29 (6 IIIA, 23 IIIB)  Unknown: 34 (16 IIIA, 18 IIIB) | Nusinersen    Administered intrathecally.  all patients should have at least one assessment after 12 months from the first dose of nusinersen, between the 6th and 7th dose of nusinersen | 144 patients with type III SMA paediatric and adult patients treated with nusinersen | 12 months | The mean duration of follow-up was 1.83 (+-0.61) years. |
| Pinar 2024 | n/a | Single-center observational cohort study | Turkey | Istanbul University-Cerrahpasa, Cerrahpasa Medical Faculty, Pediatrics Department | One arm: 37 participants | Median age of 72 months (range 48 to 132 months) | Females: 17 (45.9%)  Males: 20 (54.1%) | Types 1 (n=15), 2 (n=10), and 3 (n=12) | n/a | Nusinersen  Intrathecal  12 mg  Four initial loading doses on days 1, 15, 29, 64, followed by maintenance doses every 4 months | n/a | 2022–2023 | Before and after the study |
| RAINBOWFISH (NCT03779334) (EMA 2023) | EMA 2023 | open-label, single-arm, multicentre clinical study; BN40703 (RAINBOWFISH), an ongoing Phase II study | 14 sites including Australia, Belgium, Brazil, Russia, Taiwan, US, Italy, Poland | Not reported. Only 6/14 centres had enrolled any participants | one arm: enrolled n=18, completed 12-month follow-up n=7 | Median age at first dose 26.5 days (range: 16 – 40 days) | Not reported | Presymptomatic SMA | 2 copies | Risdiplam    Once daily, oral administration. Based on age and body weight. 16 days to < 2 years of age: 0.20 mg/kg,   ε 2 years of age, < 20 kg: 0.25 mg/kg    ε 2 years of age, ε 20 kg : 5 mg | Single arm study | screening period, a treatment phase, an open-label extension phase of at least 36 months (Month 24 up to Month 60) and a follow-up period, for a total treatment duration of at least 5 years for each infant enrolled | 12 months |
| Rich 2022 | N/A | Retrospective analysis of samples collected during two trials (NCT04139343 and   NCT04591678) | US | Ohio State University Wexner Medical Centre | 33 in nusinersen arm   6 in control group (most outcomes not collected for control group as retrospective design) | 38.5 years | Female: 18/33 (55%)   Male: 15/33 (45%) | Not specified but adult patients | 3 copies: 22/33   4 copies: 9/33   5 copies: 1 | Nusinersen    Intrathecally administered.  12mg per administration.  Loading doses at 2-weekly intervals followed by permanent maintenance doses every four months | Control group: 6 participants (3 males, 3 females; average age = 38.3 years).  CSF samples were obtained in the controls. However, given the retrospective nature of this analysis, outcomes (serum samples, motor outcomes, or electrophysiological assessments) were not taken | June 2017- January 2020 | Assessments completed prior to initial loading dose (nusinersen naive), at maintenance dose 1 (treatment day 180) and maintenance dose 3 (treatment day 420) |
| Sarikaya 2022 | N/A | Retrospective study | Turkey | Department of Paediatric Neurology of Dokuz Eylul University Faculty of Medicine | One arm: n=27 | Type 1: 9.5 months   Type 2 and 3: 72 months | Type 1: 7 female, 6 male   Type 2 and 3: 6 female, 8 male | Type 1: 13 (48.1%)   Type 2 and 3: 14 (51.8%) | Not reported | Nusinersen    Intrathecal injection. 12mg.  Four loading doses given on day 0 and the 14th, 28th, and 56th days in patients with SMA type 1, and day 0 and the 28th, 84th, and 273rd days in patients with SMA type 2 and 3.   Maintenance doses were administered every 4 months | Single arm study | Patients from department follow-up between September 2017 and June 2021 were retrospectively analysed | Assessments taken at each dose, data reported for 6 doses.   Motor scale tests performed before each dose |
| Scheijmans 2022 | N/A | Nationwide, population-based, single-centre cohort study   Historical natural history data from Dutch SMA registry | Netherlands | SMA centre in the University Medical Centre Utrecht | One arm: n=69 | Age at treatment initiation (median):   All: 54 months   Type 1: 37 months   Type 2: 55.5 months   Type 3: 68 months | Male:   All: 42/69 (60.9%)   Type 1: 18/23 (78%)   Type 2: 15/30 (50%)   Type 3: 9/16 (56%) | Type 1b: 5   Type 1c: 18   Type 2a: 20   Type 2b: 10   Type 3a: 16   Presymptomatic: 2 | (in 69 symptomatic patients):   2 copies: all: 7/69 (10%), Type 1: 6/23 (26%), Type 2: 0, Type 3: 1/16 (6%)   3 copies: all: 52/69 (75%), Type 1: 17/23 (74%), Type 2: 30/30 (100%), Type 3: 5/16 (31%)   4 copies: all: 10/69 (15%), Type 1: 0, Type 2: 0, Type 3: 10/16 (62%) | Nusinersen    Intrathecal injection.  Loading doses at day 0, 14, 28 and 63 followed by injection every four months | Single arm study | May 2017 to July 2019 | Median follow-up of 38 months (between 5-52 months)   Assessments taken at baseline and during follow-up every one to five years   Median follow up by type:   Type 1b: 41 months   Type 1c: 45.5 months   Type 2a: 34.5 months   Type 2b: 36 months   Type 3a: 33.5 months |
| Schneider 2021 | N/A | single-centre cross-sectional and longitudinal study | Germany | Patients treated nusinersen at the Department of Neurology, University Hospital of Cologne | 15 adult patients with SMA type 3, 15 age-matched healthy controls, 9 patients with amyotrophic lateral sclerosis | 5 healthy age-matched controls (seven males and eight females; mean age 39.67 years, range 21–63) and 10 patients with ALS (seven males and three females; mean age 65.6 years, range 51–81 | SMA n=15   N=10 males   N=5 females | Type 3 | Not reported | Nusinersen    Number of nusinersen courses at entry: 6.93 ± 3.06 (1–10) | n/a | not reported | Followed up after 4–8 months |
| SHINE (CS11) (NCT02594124) (Dunaway Young 2023) | Dunaway Young et al. 2023, CADTH nusinersen 2018_clinical, ICER spinraza and zolgensma 2019, CADTH nusinersen reassessment 2022_clin and econ, CADTH nusinersen resubmission 2019_clinical, FDA nusinersen 2016_medical review, TA588 final appraisal committee 2019, TA588 MAA extension company submission 2020 | An ongoing open-label extension study of nusinersen in participants who were enrolled in previous nusinersen trials (ENDEAR, CHERISH, CS3A, and CS12) | Global (Australia, Belgium, Canada, France, Germany, Hong Kong, Italy, Japan, Korea, Republic of,  Spain,  Sweden,  Turkey,  United Kingdom,  United States) | Secondary Care | Participants from CHERISH and ENDEAR who had been in previous nusinersen arms or previous sham arms. All received nusinersen in SHINE. enrolled from ENDEAR: previous nusinersen: enrolled: n=65, analysed: n= 48. previous sham: enrolled: n=24, analysed: n=12. Enrolled from CHERISH: previous nusinersen: enrolled: n=83, analysed: n=58, previous sham: enrolled: n=42, analysed: n=24 | Examining infantile and later-onset patients   Median age 4.0 range 0.6 to 19.3 years | Male and female included | Type 1 (expanded access programs had a common inclusion criterion of diagnosis of SMA type I)   SHINE included people with type 2 or type 3 SMA | 2 copies: 90   3 copies: 110   4 copies: 7 | Nusinersen    5 mL solution for intrathecal injection.  Dosing scale to a 12-mg equivalent for children under 2 and 12mg for all other children. four loading doses (days 0, 14, 28, and 63) followed by maintenance treatment of 5 mL solution every four months. | All received nusinersen in SHINE- previously some participants had received sham procedure in ENDEAR and CHERISH studies | Screening phase, a treatment period that included a loading dose and maintenance treatment, a post-treatment follow-up period, and an end-of-study evaluation.    The planned duration of participation in the study is 5 years after administration of the modified maintenance dosing regimen | Most children in this analysis had not reached adolescence at the onset of scoliosis (mean age at first nusinersen dose was approximately 4 years) and were followed for up to 30 months. Many outcomes available for 1814 days (approx. 5 years) |
| Simic 2022 | N/A | Longitudinal study | Croatia | Department of Paediatrics, University Hospital Centre Zagreb, Croatia | One arm: n=30 | 1 month- 17 years | Male: 19/30, Female: 11/30 | Type 1: 13   Type 2: 4   Type 3: 9 | SMN1 exon 8: 0 copies: 22, 1 copy: 8   SMN2 exon 7: 1 copy: 1, 2 copies: 16, 3 copies: 9, 4 copies: 3   SMN2 exon 8: 1 copy: 2, 2 copies: 15, 3 copies: 10, 4 copies: 3 | Nusinersen    Intrathecal administration.  Does not reported   Assessment at 1-5, 6-10 and 11-15 doses | Single arm study | 2017-2021 | CSF samples were taken at nusinersen administration up to 18-24 months   75-1275 days, average 675 days   Assessment at 1-5, 6-10 and 11-15 doses |
| Simsek 2024 | N/A | Retrospective study | Turkey | Clinical departments | Sub-groups. Ambulatory, n=11   non-ambulatory, n=10 | Median 36 years | Male, n=14   Female, n=7 | Type 3, n=19   Type 2, n=2 | 2 copies, n=2   3 copies, n=8   4 copies, n=11 | Nusinersen    12mg.  Day 1, Day 29, Day 85, and Day 274 | Single arm study | 15 months | Not reported |
| Sitas 2024 | N/A | Real-world data. Single-arm study | Republic of Croatia | One hospital | one arm: n=31 | 0.5-18 years, median age at symptom onset 2 years | 15/31 male, 16/31 female | Type 2 n=15/31, type 3 n= 16/31 | 2 copies: 5/31 (16.12%), 3 copies: 18/31 (58.06%), 4 copies: 8 (26.8%) | Risdiplam    Oral administration, 5mg daily | Single arm study | SMA type 2 median duration of 16 months (n=3) and 30 months (n=12), SMA type 3 median duration of 16 months (n=16) | motor function evaluated every 6 months during treatment |
| SM201 (EMA 2017) | EMA 2017   FDA 2016 | Open-label, multicentre, phase 2, single-arm study | Not reported | Not reported | n=25 (planned)   EMA 2017: n=17 | 6 weeks or younger (at the time of enrolment) | Male 65% (EMA 2017 p76) | Presymptomatic SMA | EMA 2017:    12/17 (71%) 2 copies   5/17 (29%) 3 copies | Nusinersen    Intrathecal administration, 12mg.  Loading and maintenance doses | Single arm study | Not reported | 1 year |
| SUNFISH (NCT02908685) (Mercuri 2022  Mercuri 2023  Oskoui 2023) | Mercuri2022   Mercuri2023   Oskoui2023 | A randomised, double-blind, placebo-controlled, dose-finding trial followed by 24 months of treatment | The study was conducted at 42 hospitals in Belgium, Brazil, Canada, China, Croatia, France, Italy, Japan, Poland, Russia, Serbia, Spain, Turkey, and the USA | Mercuri2022: “Recruited from 42 hospitals in 14 countries across Europe, North America, South America, and Asia.”   Mercuri2023: “Four countries (Italy [two sites], Germany, France, and Belgium [one site each]) in five subsequent cohorts.” | Risdiplam: n=120 and placebo: n=60 | aged 2–25 years   CADTH: “The mean age of enrolled patients was 9.9 (SD   = 5.8) years in the risdiplam group and 10.3 (SD = 6.1) years in the placebo group”   Age group years, N (%) (Risdiplam)   2-6 years 37 (30.8)   6-11 years 39 (32.5)   12-17 years 30 (25.0)   18-25 years 14 (11.7) | female/male, n (%)   Patients aged 2–11 years = 31 14 (45)/17 (55)    Patients aged 12–25 years = 20 13 (65)/7 (35)   All patients (n=51) 27(53)/24 (47) | Type 2 and 3 SMA type, n (%)   Type II: Risdiplam: 84 (70.0), placebo:  44 (73.3)   Type III: Risdiplam:  36 (30.0), placebo:  16 (26.7) | Mercuri2023    “the majority (90%) had three SMN2 copies”   SMN2 copy number, n (%)   2 copies aged 2–11 years 0 aged 12–25 years 1 (5) All patients 1 (2)   3 copies aged 2–11 years aged 28 (90) 12–25 years 18 (90) All patients 46 (90)   4 copies aged 2–11 years aged 3 (10) 12–25 years 1 (5) All patients 4 (8) | Risdiplam    Oral solution, dissolved in water to a concentration of 0·75mg/mL. taken at home or a clinical site. Three cohorts of patients aged 2–11 years (Group A) were administered the following dose levels: 0.02, 0.05, 0.15, and 0.25 mg/kg. Two cohorts of patients aged 12–25 years (Group B) received 3 and 5 mg of risdiplam (Figure 1). Following the selection of the pivotal dose for Part 2 (5 mg for patients with a body weight ≥20 kg, 0.25 mg/kg for patients weighing <20 kg), all patients switched to the pivotal dose” | Placebo- also dissolved and administered orally | Mercuri2023 minimum 12-week   “The placebo-controlled period ranged from 19.1 to 26.9 weeks, as all patients in a specific cohort were switched to risdiplam only when the last patient enrolled in the cohort completed 12 weeks and after IMC review of all data”   Oskoui   “All patients remaining in the study entered the open-label extension phase for ≥ 24 months of further treatment | The study had a screening period of up to 30 days, and a randomised, double-blind, 12-month placebo-controlled period, followed by a second 12-month open-label period where all individuals received risdiplam. After completion of the 24-month treatment period, individuals could continue in the open-label extension for 3 years |
| Szabo 2020 | N/A | Retrospective study | Hungary | Hungarian treatment centres | one arm: n=54 | 6.3 years (mean) (±5,4 range 0.4-17.9) | Male, n=34   Female, n=20 | Type 1-3 | 2 copies, n=11   3 copies, n=33   4 copies, n=10 | Nusinersen    Injection.  Patients were assessed before administration of the first dose of nusinersen, at the time of the 4th injection (63 days of treatment), and then every four months before the administration of the next injection | Single arm study | April 2018 and December 2019 | 408 days (440 median) |
| Tachibana 2022 | N/A | Interim analysis of ongoing post-marketing surveillance | Japan | Case report forms in post-marketing surveillance | One arm: n=401 | 78/126 (61.9%) were aged between 2-15 years at the start of treatment | 227 (56.6%) males | Infantile-onset: 126/401   Later-onset: 275/401   Type 2: 165/401   Type 3: 105/401   Type 4: 5/401 | 1 copy: infantile-onset: 2/126, later-onset: 1/275, total: 3/401   2 copies: infantile-onset: 100/126, later-onset: 16/275, total: 116/401   3 copies: infantile-onset: 23/126, later-onset: 190/275, total: 213/401   4 or more copies: infantile-onset: 0, later-onset: 62/275, total: 62/401   Other: infantile-onset: 0, later-onset: 3/275, total: 3/401   Unknown: infantile-onset: 1/126, later-onset: 3/275, total: 4/401 | Nusinersen    Intrathecally administered.  9.6-12mg based on age in days.  Infantile-onset: First dose, weeks 2, 4 and 9 and then every 4 months   Later-onset: First dose, weeks 4 and 12 and then every 6 months | Single arm study | Ongoing: August 2017- August 2025.   Patient registration until August 2024 and follow-up until August 2025. | Case report forms completed at 3 months from first nusinersen dose, and then yearly, until end of study or death.   Final follow-up will be August 2025 |
| Tachibana 2023 | N/A | Real-world data/ post-marketing surveillance | Japan | Clinical settings | One arm: n=524 | Infantile-onset (n=153):   87 (56.9%) aged between 2-15 years at the start of treatment   Later-onset (n=369):   196 (53.1%) aged between 15-64 years at start of treatment | Male:   Infantile onset Type 1: 83 (54.2%),  Later onset Type 2-4: 207 (56.1%)   All: 291 (55.5%) | Infantile onset (Type 1): 153 (29.3%)   Later onset:   Type 2: 208 (56.4%) (39.8%)   Type 3: 154 (41.7%) (29.5%)   Type 4: 7 (1.9%) (1.3%) | 1 copy: Infantile onset: 2 (1.3%), later-onset: 1 (0.3%), all: 3 (0.6%)  2 copies: Infantile onset: 121 (79.1%), later-onset: 20 (5.4%) all: 141 (26.9%)   3 copies: Infantile onset: 28 (18.3%), later-onset: 236 (64.0%) all: 264 (50.4%)   4 or more: Infantile onset: 1( 0.7%), later-onset: 105 (28.5%) all (20.2%)   Other or unknown: Infantile onset: 1 (0.7%), later-onset: 7 (1.9%) | Nusinersen    Intrathecal injection.  Initial dose: 12mg/5ml in 456 patients, 11.3mg/4.7ml in 27 patients, 10.8mg/4.5ml in 13 patients, 10.3mg/4.3ml in 15 patients and 9.6mg/4.0ml in 13 patients | Single arm study | 3 years | Data was collected at baseline and at 36 months after first dose |
| Trancho 2024 | N/A | Observational study | Not reported | Not reported | Ambulatory individuals with SMA, n=19   Age-matched controls, n=16 | 32.9 (12.7–56.8) years | 73.7% male | Not reported | Not reported | Nusinersen    Not reported | Age-matched controls | 1.0 ± 0.3 years | Not reported |
| Trucco 2023 | N/A | Multicentre cross-sectional prospective study | Italy | Two Italian highly specialized neuromuscular sites | Children treated: 45   Adults treated: 22   Adults untreated: 11 | 45/78 children 33/78 adults.   At enrolment, the median age of children was 7.42 (5.69- 9.69),  Median age of adults on nusinersen was 26.80 (24.10-18.42),   Median age of adults untreated: 32.67 (21.55-41.31) | Children: male: 21/45, female: 24/45   Treated adults: male: 13/22, female: 9/22   Untreated adults: male: 7/11, female: 4/11 | Type 2 and 3 | Children: one copy: 0, two copies: 3 (8.33%), three copies: 30 (83.33%), four copies: 1 (2.78%), more than four: 2 (5.56) (9 missing)   Adults treated: one copy: 0, two copies: 4 (20%), three copies: 10 (50%), four copies: 1 (5%), more than four: 5 (25%) (2 missing)   Adults untreated: one copy: 0, two copies: 1 (20%), three copies: 3 (60%), four copies: 0, more than four: 1 (20%) 6 Missing | Nusinersen    Intrathecal injection 12mg dose | Adults untreated with nusinersen included as well as adults treated | Two years (2019–2020) | Swallowing and mastification evaluated every 4 months for up to 16 months |
| Trucco 2024 | n/a | 5-year retrospective longitudinal observational study | Italy, US and UK | Pediatric patients (age 5–18 years) diagnosed with SMA type 2 and nonambulant type 3 treated with nusinersen for at least 3 months within the iSMAc centers (SMA REACH UK, Italian SMA network, and PNCRN UK, US, Italy). | 69 treated patients (53 SMA type 2, 16 SMA type 3).  Control/ untreated group compared (natural history control NHC) n=103 from iSMAc with SMA | Treated Patients:  SMA II: Mean (SD): 9.1 (3.1) years  SMA III: Mean (SD): 9.9 (3.7) years  Untreated Patients from NH:  SMA II: Mean (SD): 9.3 (3.7) years  SMA III: Mean (SD): 11.7 (3.0) years | Male, n (%):  Treated Patients:  SMA II: 33 (62.3%)  SMA III: 5/15 (31.3%)  Untreated Patients from NH:  SMA II: 33 (58.9%)  SMA III: 7/15 (46.7%) | SMA II (n = 53)  SMA III (n = 16)  Untreated Patients from NH:  SMA II (n = 84)  SMA III (n = 19) | Treated Patients:  SMA II:  1 copy: 1 (1.9%)  2 copies: 5 (9.4%)  3 copies: 31 (58.5%)  4 copies: 1 (1.9%)  Unknown: 15 (28.3%)  SMA III:  1 copy: 0  2 copies: 3 (18.8%)  3 copies: 8 (50.0%)  4 copies: 1 (6.2%)  Unknown: 4 (25.0%)  Untreated Patients from NH:  SMA II:  1 copy: 5 (5.9%)  2 copies: 27 (32.1%)  3 copies: 52 (62.0%)  4 copies: 0  Unknown: 0  SMA III:  1 copy: 0  2 copies: 6 (31.6%)  3 copies: 13 (68.4%)  4 copies: 0  Unknown: 0 | Nusinersen (Administered under Managed Access Agreement (UK) or as an approved medication (US, Italy).)  Intrathecal  Loading dose of 12 mg (5 mL) on days 0, 14, 28, and 63, followed by a maintenance dose of 12 mg (5 mL) every 4 months. | n/a | (December 2017–August 2022); 5 years | Treated Patients:  SMA II: Median (IQR): 1.0 (0.7–1.9) years  SMA III: Median (IQR): 1.2 (0.9–1.9) years  Untreated Patients from NH:  SMA II: Median (IQR): 2.2 (1.3–3.3) years  SMA III: Median (IQR): 2.2 (0.9–3.1) years  Timing of assessment:  V0 (first administration), end of loading dose (6 months after first injection), 12 months after first injection, and every 4 months thereafter. |
| Tscherter 2022 | N/A | observational study | Switzerland | The study used data collected by the Swiss Registry for Neuromuscular Disorders (Swiss-Reg-NMD). Patients identified in regional neuromuscular centres | one arm: n=44 | Age at treatment start (median):   Type 1 0-18 months: 0.2 years   Type 1 > 18 months: 13.1 years   Type 3: 7.8 years   Type 3 ambulatory: 15.4 years   Type 3 non-ambulatory: 18.5 years | Male: 21/44  Female: 23/44 | Type 1: 11   Type 2: 21   Type 3: 12 | 2 copies:   All: 7, Type 1: 5, Type 2:  1, Type 3:1   3 copies:   All: 23, Type 1: 4, Type 2: 16, Type 3: 3   4 or more copies:   All: 9, Type 1: 0, Type 2: 3, Type 3: 6 | Nusinersen    Lumbar puncture administration. Every 4 months after a loading period of 2 months | Single arm study | Data collected until 31^st^ August 2020 | Up to 42 months of follow-up |
| Van der Heul 2020 | N/A | Prospective study | Netherlands | Outpatient clinic of the Netherlands SMA Centre at the University Medical centre Utrecht | Palliative care group, n=11   Nusinersen group, n=5 | Palliative care group, (days) 52 (16–252)   Nusinersen group, (days) 63 (3–218) | Palliative care group, male n=8, female n=3   Nusinersen group, male n=3, female n=2 | Type 1 | 2 copies | Nusinersen    Not reported | Palliative care (control group) | September 2016 to October 2018 | Not reported |
| van der Woude 2024 | n/a | Observational prospective study | Netherlands | Netherlands SMA Center, University Medical Center Utrecht (UMCU) | One arm: 46 patients | Median age at baseline was 5.2 years (range 1.5–9.9) | Male 25 (54)  Female 21 (46) | Types 2a n=23  Type 2b n=9)  Type 3a n=14 | Type 2a:  2 copies: 1 (4.3%)  3 copies: 22 (95.7%)  4 copies: 0 (0%)    Type 2b:  2 copies: 0  3 copies: 9 (100%)  4 copies: 0    Type 3a:  2 copies: 1 (7.1%)  3 copies: 4 (28.6%)  4 copies: 9 (64.3%) | Nusinersen  Intrathecal  5 to 8 doses, majority receiving 7 doses  According to the manufacturer’s schedule | n/a | May 2017 to August 2019 | At least 20 months  Children were assessed at least once a year.  The first assessment was performed within six months, up to two weeks after the start of treatment.  The second assessment was performed after at least the full loading dose and 5th injection, provided no spinal surgery had been performed between the two assessments. |
| Vazquez-Costa 2022 | N/A | Prospective observational study | Spain | Five referral centres in Spain | Total sample: 79   Nusinersen treatment arm: 39   Untreated patients: 40 | Mean: Non-treated: 30.34 years   Treated: 33.35 years | Male:   non-treated: 17/40 (42.5%)   Treated: 20/39 (51.28%) | 2a: non-treated: 14/40 (35%), treated: 8/39 (20.51%)   2b: non-treated: 6/40 (15%), treated: 2/39 (5.13%)   3a: non-treated: 8/40 (20%), treated: 15/39 (38.46%)   3b: non-treated: 9/40 (22.5%), treated: 14/39 (35.9%)   4: non-treated: 3/40 (7.5%), treated: 0 | 1 copy: non-treated: 1/39 (2.5%), treated: 0   2 copies: non-treated: 2/39 (5%), treated: 5/40 (12.82%)   3 copies: non-treated: 27/39 (67.5%), treated: 23/40 (58.97%)   4 copies: non-treated: 10/39 (25%), treated: 11/40 (28.21%) | Nusinersen    Injection. 12mg. Loading doses at days 0, 14, 28, and 65, followed by maintenance doses every 4 months after | Untreated patients.      All patients received the same multidisciplinary care | Not reported | Mean follow-up: Non-treated: 15.8 months, treated: 16.06 months |
| Vidovic 2023 | N/A | Prospective longitudinal study | Germany | One university hospital | One arm: n=23 | Mean age at baseline:   Total: 38.1 years   Type 2: 31.9 years  Type 3: 42.1 years | Total: Male: 13/23, female: 10/23   Type 2: Male: 6/9, female: 3/9   Type 3: Male: 7/14, female: 7/14 | Type 2: n=9   Type 3: n= 14 | Total: 2 copies: 2/23, 3 copies: 1/9, 4 copies: 1/14  Type 2: 2 copies: 13/23, 3 copies: 8/9, 4 copies: 5/14   Type 3: 2 copies: 8/23, 3 copies: 0, 4 copies: 8/14 | Nusinersen    Not reported by the paper | Single arm study | 14 months | Assessments taken at baseline (before first loading dose) and 14 months after first assessments: one year after treatment initiation |
| Vill 2021 | N/A | Prospective cohort study | Germany | Treatment centre | One arm: n=43 | 0-24 months | Not reported | Not reported | 2 copies, n=17   3 copies, n=10   4 copies, n=14   5 copies, n=2 | Nusinersen    Not reported | Single arm study | January 2018 and January 2020 | Between January 2018 and January 2020, with data collection ending in April 2020 |
| Wang 2024 | n/a | Retrospective, observational | China | Huashan Hospital, Fudan University  First Affiliated Hospital of Soochow University  First Affiliated Hospital of Anhui Medical University | 54:Sitters: 22 patients  Walkers: 32 patients | 27.03 ± 9.85 years (range 13–53 years) | 64.81% male | SMA 2: 9 patients  SMA 3: 43 patients  SMA 4: 2 patients | 2:1  3:29  4:23  5:1 | Nusinersen  Intrathecal loading doses of 12 mg nusinersen at:  Baseline (V1)  Day 14 (V2)  Day 28 (V3)  Day 63 (V4)  Maintenance doses every 4 months (V5 at day 180 and V6 at day 300) | n/a | October 2022 to July 2023 | Visit 5 at day 180  and Visit 6 at day 300)  Timing of assessment:  Baseline, V4, V5, and V6 |
| Weaver 2021 | N/A | Observational cohort study | US | Outpatient neuromuscular clinic | Total n=35     No intent of treatment n=5   Intent of treatment n=8   Loading phase n=11   Maintenance phase n=11 | Children   Average age:   Type 1 =2.7 ±2.1 yrs   Type 2 =11.2 ±5.9 yrs   Type 3 =10.2 ±2.7 yrs | Not reported | 1-3   Type 1 n=15   Type 2 n=14   Type 3 n=6 | Not reported | Nusinersen    Intrathecal.  Loading: Four doses by baseline    Maintenance: Every 4 months at baseline | Non-treatment/No intent of treatment | November 2016 to September 2019 | 24 months |
| Weber 2024 | N/A | Non-interventional, longitudinal pilot study | Germany | Department of Neurology and Neuropediatric at the University Hospital at the Technische Universität Dresden | Adults, n=23   Children, n=16 | Adults (years), 38.4 (18.4–57.9)   Children (years), 10.0 (5.1–16.6) | Subgroups. Adults, male n=11, female n=12   Children, male n=8, female n=8 | Adult   Type 2, n=10   Type 3, n=13   Children    Type 2, n=12   Type 3, n=4 | Adults   2 copies, n=2   3 copies, n=12   4 copies, n=8   Unknown, n=1   Children   2 copies, n=0   3 copies, n=13   4 copies, n=3   Unknown copies, n=0 | Nusinersen    intrathecal administration | Single arm study | 12-month treatment period | 12 months |
| Weststrate 2022 | N/A | retrospective study | UK | Great Ormond Street Hospital for Children, London, UK | one arm: n=24 | Median age at baseline: 11 months | Female: 14/24   Male: 10/24 | Not reported | 2 copies:   All: 17/23 (74%), Type 1a: 2/3 (67%), Type 1b: 9/9 (100%), Type 1c: 6/11 (55%)   3 copies:   All: 6/23 (26%), Type 1a: 1/3 (33%), Type 1b: 0, Type 1c: 5/11 (45%) | Nusinersen    Not reported | Single arm study | Children treated with nusinersen between 2017 and 2020 | 24 months |
| Wohnrade 2023 | N/A | Two prospective cross-sectional multi-center studies | Germany | Five specialized motor neuron disease clinics throughout Germany | One arm. SMA patients (n = 39)   SMA patient/caregiver couples (n = 49) | 36 (19–65) | Male, n=25   Female, n=14 | Type 2, n=13   Type 3,4, n=26 | ≤3, n=17   ≥4, n=16   Unknown, n=6 | Nusinersen    Not reported | Single arm study | November 2018 to March 2020 | November 2018 to March 2020 |
| Yao 2024 | n/a | Longitudinal, multi-center registry collecting both prospective and retrospective data | China | pediatric patients with 5q-SMA across 18 centers in China | 385 were treated with nusinersen | Median age at Genetic Diagnosis:  Type I: 7 months (IQR 5–42).  Type II: 18 months (IQR 12–36).  Type III: 57 months (IQR 29–119) | 50.9% males (196), 49.1% females (189) | Type I: 10.7% (41 patients).  Type II: 56.0% (214 patients).  Type III: 33.3% (127 patients) | Type I: 44.1% (2 copies), 52.9% (3 copies), 2.9% (≥ 4 copies).  Type II: 9.2% (2 copies), 87.7% (3 copies), 3.1% (≥ 4 copies).  Type III: 10.8% (2 copies), 67.7% (3 copies), 21.5% (≥ 4 copies) | Nusinersen  Interathecal |  | 14 months | Planned at 6, 10, and 14 months post-nusinersen initiation, not earlier than April 28th, 2019  Baseline, months 6,10,14    Both prospective and retrospective data on baseline characteristics and effectiveness, with safety data collected prospectively |
| Yasar 2024 | n/a | Retrospective study | Turkey | n/a | 29 | Mean age 3.7 ± 1.1 years (range 2–6) | Male-to-Female Ratio: 1.1 | Type 1 patients | n/a | nusinersen  intrathecal  Average nusinersen administration:8.9 ± 2.9 (range 4–19). | n/a | between 2017 and 2021 | 2 to 6 years, mean 3.3 ± 1.1 years |
| Yu 2024 | N/A | Retrospective study | Not reported | Data was taken from the publicly available FAERS database, with no study setting | one arm: n=1588 | Adverse events report of target drug as the PS   < 18, n=355   > or = 18 and < 45, n=288   > or = 45 and < 65, n=96   > or = 65, n=22   Missing, n=827 | Adverse events report of target drug as the PS   Male, n=552   Female, n=755 | Not reported | Not reported | Risdiplam    Not reported | Single arm study | Q3 of 2020 to Q2 of 2023. | N/A |
| Zhang 2024 | n/a | real-world drug  safety surveillance study based on the FDA adverse event reporting system (FAERS) database | Multi-national study. The United States was the leading  reporting country, accounting for 74.8% of the total, followed  by Brazil with 2.39% and Turkey with 2.17%, among other  countries. | Analyzed adverse drug event reporting data of Nusinersen from the last quarter of 2016 to the first quarter of 2024 | 6071 reports after removing duplicates and screening.  Analyzed 12,124,854 cases from the FAERS database (last quarter of 2016 to first quarter of 2024). | Majority were children (37.34%). | Slightly higher percentage in females (46.17%) than males (44.84%). | n/a | n/a | Nusinersen | n/a | 2017-2024; 8 years | Case reports and case populations between 2017-2024 |

# Supplementary S7: Citation list of studies where nusinersen was the intervention (n=112):

AlRuthia. Proxy-reported quality of life and access to nusinersen among patients with spinal muscular atrophy in Saudi Arabia. Patient Prefer Adherence 2021;15:729-39. <http://dx.doi.org/10.2147/PPA.S305849>

AlTawari. Nusinersen treatment for spinal muscular atrophy: retrospective multicenter study of pediatric and adult patients in Kuwait. Neurol Int 2024;16(3):631-42. <http://dx.doi.org/10.3390/neurolint16030047>

Alves. Implications of circulating neurofilaments for spinal muscular atrophy treatment early in life: a case series. Mol Ther Methods Clin Dev 2021;23:524-38. <http://dx.doi.org/10.1016/j.omtm.2021.10.011>

Aragon-Gawinska. Sitting in patients with spinal muscular atrophy type 1 treated with nusinersen. Dev Med Child Neurol 2020;62(3):310-4. <http://dx.doi.org/10.1111/dmcn.14412>

Arnold. Persistent neuromuscular junction transmission defects in adults with spinal muscular atrophy treated with nusinersen. BMJ Neurol Open 2021;3(2):e000164. <http://dx.doi.org/10.1136/bmjno-2021-000164>

Arslan. Nusinersen for adults with spinal muscular atrophy. Neurol Sci 2023;44(7):2393-400. <http://dx.doi.org/10.1007/s10072-023-06698-9>

Acsadi. Safety and efficacy of nusinersen in spinal muscular atrophy: the EMBRACE study. Muscle Nerve 2021;63(5):668-77. <http://dx.doi.org/10.1002/mus.27187>

Audic. Effects of nusinersen after one year of treatment in 123 children with SMA type 1 or 2: a French real-life observational study. Orphanet J Rare Dis 2020;15:148. <http://dx.doi.org/10.1186/s13023-020-01414-8>

Audic. Effect of nusinersen after 3 years of treatment in 57 young children with SMA in terms of SMN2 copy number or type. Arch Pediatr 2023;31(2):117-23. <http://dx.doi.org/10.1016/j.arcped.2023.10.009>

Axente. Clinical and electrophysiological changes in pediatric spinal muscular atrophy after 2 years of nusinersen treatment. Pharmaceutics 2022;14(10):2074. <http://dx.doi.org/10.3390/pharmaceutics14102074>

Belancic. Effectiveness of nusinersen in type 1, 2 and 3 spinal muscular atrophy: Croatian real-world data. J Clin Med 2023;12(8):2839. <http://dx.doi.org/10.3390/jcm12082839>

Berti. Oral and Swallowing Abilities Tool (OrSAT) in nusinersen treated patients. Arch Dis Child 2022;107(10):912-6. <http://dx.doi.org/10.1136/archdischild-2022-323899>

Biogen. *Nusinersen for treating spinal muscular atrophy TA588. Managed access agreement treatment criteria evidence review: non-ambulant type III spinal muscular atrophy population: company evidence submission*. National Institute for Health and Care Excellence (NICE); 2020. URL: https://www.nice.org.uk/guidance/ta588/evidence/company-evidence-submission-pdf-9138885230 (Accessed 2025 Feb 17).

Bjelica. An observational cohort study on pulmonary function in adult patients with 5q-spinal muscular atrophy under nusinersen therapy. J Neurol 2023;270(7):3616-22. <http://dx.doi.org/10.1007/s00415-023-11711-4>

Brakemeier. Assessment of bulbar function in adult patients with 5q-SMA type 2 and 3 under treatment with nusinersen. Brain Sci 2021;11(9):1244. <http://dx.doi.org/10.3390/brainsci11091244>

Cavaloiu. Nusinersen improves motor function in type 2 and 3 spinal muscular atrophy patients across time. Biomedicines 2024;12(8):1782. <http://dx.doi.org/10.3390/biomedicines12081782>

Cavaloiu. Quality of life assessment in Romanian patients with spinal muscular atrophy undergoing nusinersen treatment. Neurol Int 2024;16(5):891-904. <http://dx.doi.org/10.3390/neurolint16050067>

Chacko. Effect of nusinersen on respiratory function in paediatric spinal muscular atrophy types 1-3. Thorax 2022;77(1):40-6. <http://dx.doi.org/10.1136/thoraxjnl-2020-216564>

Chen. Effects of nusinersen on motor function in children with spinal muscular atrophy: a retrospective study. Front Neurol 2024;15:1391613. <http://dx.doi.org/10.3389/fneur.2024.1391613>

Chiriboga. Results from a phase 1 study of nusinersen (ISIS-SMN(Rx)) in children with spinal muscular atrophy. Neurology 2016;86(10):890-7. <http://dx.doi.org/10.1212/wnl.0000000000002445>

Cho. Nusinersen demonstrates effectiveness in treating spinal muscular atrophy: findings from a three-year nationwide study in Korea. Front Neurol 2023;14:1294028. <http://dx.doi.org/10.3389/fneur.2023.1294028>

Coratti. Age related treatment effect in type II spinal muscular atrophy pediatric patients treated with nusinersen. Neuromuscul Disord 2021;31(7):596-602. <http://dx.doi.org/10.1016/j.nmd.2021.03.012>

Coratti. 197P Survival of SMA type 1 and type 0 infants at the time of disease modifying therapies: results of an 8-year nationwide registry. Neuromuscul Disord 2024;43(Suppl 1):104441.448. <http://dx.doi.org/10.1016/j.nmd.2024.07.457>

Crawford. Continued benefit of nusinersen initiated in the presymptomatic stage of spinal muscular atrophy: 5-year update of the NURTURE study. Muscle Nerve 2023;68(2):157-70. <http://dx.doi.org/10.1002/mus.27853>

Darras. Nusinersen in later-onset spinal muscular atrophy: long-term results from the phase 1/2 studies. Neurology 2019;92(21):e2492-506. <http://dx.doi.org/10.1212/WNL.0000000000007527>

De Wel. Nusinersen treatment significantly improves hand grip strength, hand motor function and MRC sum scores in adult patients with spinal muscular atrophy types 3 and 4. J Neurol 2021;268(3):923-35. <http://dx.doi.org/10.1007/s00415-020-10223-9>

Duan. Assessment of health-related quality of life in patients with spina muscular atrophy in China. Intractable Rare Dis Res 2022;11(4):189-95. <http://dx.doi.org/10.5582/irdr.2022.01094>

Dunaway Young. Nusinersen treatment of children with later-onset spinal muscular atrophy and scoliosis is associated with improvements or stabilization of motor function. J Clin Med 2023;12(15):4901. <http://dx.doi.org/10.3390/jcm12154901>

Duong. Nusinersen treatment in adults with spinal muscular atrophy. Neurol Clin Pract 2021;11(3):e317-27. <http://dx.doi.org/10.1212/cpj.0000000000001033>

Edel. A new respiratory scoring system for evaluation of respiratory outcomes in children with spinal muscular atrophy type1 (SMA1) on SMN enhancing drugs. Neuromuscul Disord 2021;31(4):300-9. <http://dx.doi.org/10.1016/j.nmd.2021.01.008>

European Medicines Agency. Assessment report: Spinraza. Procedure no. EMEA/H/C/004312/0000. 2017. URL: https://www.ema.europa.eu/en/documents/assessment-report/spinraza-epar-public-assessment-report_en.pdf-0 (Accessed 2025 Feb 17).

Finkel. Nusinersen versus sham control in infantile-onset spinal muscular atrophy. N Engl J Med 2017;377(18):1723-32. <http://dx.doi.org/10.1056/nejmoa1702752>

Fainmesser. Longer-term follow-up of nusinersen efficacy and safety in adult patients with spinal muscular atrophy types 2 and 3. Neuromuscul Disord 2022;32(6):451-9. <http://dx.doi.org/10.1016/j.nmd.2022.04.003>

Finkel. Treatment of infantile-onset spinal muscular atrophy with nusinersen: final report of a phase 2, open-label, multicentre, dose-escalation study. Lancet Child Adolesc Health 2021;5(7):491-500. <http://dx.doi.org/10.1016/S2352-4642(21)00100-0>

Freigang. Impaired diaphragmatic motility in treatment-naive adult patients with spinal muscular atrophy improved during nusinersen treatment. Muscle Nerve 2023;68(3):278-85. <http://dx.doi.org/10.1002/mus.27938>

Freigang. Increased chitotriosidase 1 concentration following nusinersen treatment in spinal muscular atrophy. Orphanet J Rare Dis 2021;16(1):330. <http://dx.doi.org/10.1186/s13023-021-01961-8>

Freigang. Serum creatine kinase and creatinine in adult spinal muscular atrophy under nusinersen treatment. Ann Clin Transl Neurol 2021;8(5):1049-63. <http://dx.doi.org/10.1002/acn3.51340>

Gaboli. Effect of nusinersen on respiratory and bulbar function in children with spinal muscular atrophy: real world experience from a single center. Neuropediatrics 2024;56(1):2-11. <http://dx.doi.org/10.1055/a-2379-7069>

Gomez-Garcia de la Banda. Assessment of respiratory muscles and motor function in children with SMA treated by nusinersen. Pediatr Pulmonol 2021;56(1):299-306. <http://dx.doi.org/10.1002/ppul.25142>

Goedeker. Laboratory monitoring of nusinersen safety. Muscle Nerve 2021;63(6):902-5. <http://dx.doi.org/10.1002/mus.27217>

Gonski. Respiratory and sleep outcomes in children with SMA treated with nusinersen - real world experience. Neuromuscul Disord 2023;33(6):531-8. <http://dx.doi.org/10.1016/j.nmd.2023.04.007>

Govoni. Six-minute walk test as outcome measure of fatigability in adults with spinal muscular atrophy treated with nusinersen. Muscle Nerve 2024;70(4):816-23. <http://dx.doi.org/10.1002/mus.28225>

Gunther. Long-term efficacy and safety of nusinersen in adults with 5q spinal muscular atrophy: a prospective European multinational observational study. Lancet Reg Health Eur 2024;39:100862. <http://dx.doi.org/10.1016/j.lanepe.2024.100862>

Hagenacker. Nusinersen in adults with 5q spinal muscular atrophy: a non-interventional, multicentre, observational cohort study. Lancet Neurol 2020;19(4):317-25. <http://dx.doi.org/10.1016/S1474-4422(20)30037-5>

Huang. Longitudinal efficacy of nusinersen treatment on health-related quality of life and independence in children with later-onset spinal muscular atrophy. Muscle Nerve 2024;71:368-76. <http://dx.doi.org/10.1002/mus.28311>

Ip. Treatment of symptomatic spinal muscular atrophy with nusinersen: a prospective longitudinal study on scoliosis progression. J Neuromuscul Dis 2024;11(2):349-59. <http://dx.doi.org/10.3233/JND-230077>

Jiang. Unveiling the adverse events of nusinersen in spinal muscular atrophy management based on FAERS database. Sci Rep 2024;14(1):17138. <http://dx.doi.org/10.1038/s41598-024-67627-0>

Jiang. A post-marketing surveillance study of nusinersen for spinal muscular atrophy in routine medical practice in China: interim results. Adv Ther 2024;41(7):2743-56. <http://dx.doi.org/10.1007/s12325-024-02852-7>

Kariyawasam. Motor unit changes in children with symptomatic spinal muscular atrophy treated with nusinersen. J Neurol Neurosurg Psychiatry 2021;92(1):78-85. <http://dx.doi.org/10.1136/jnnp-2020-324254>

Kölbel. Parental burden and quality of life in 5q-SMA diagnosed by newborn screening. Children (Basel) 2022;9(12):1829. <http://dx.doi.org/10.3390/children9121829>

Konersman. Nusinersen treatment of older children and adults with spinal muscular atrophy. Neuromuscul Disord 2021;31(3):183-93. <http://dx.doi.org/10.1016/j.nmd.2020.12.006>

Kotulska. Safety, tolerability, and efficacy of a widely available nusinersen program for Polish children with spinal muscular atrophy. Eur J Paediatr Neurol 2022;39:103-9. <http://dx.doi.org/10.1016/j.ejpn.2022.06.001>

Kroksmark. Low bone mineral density and reduced bone-specific alkaline phosphatase in 5q spinal muscular atrophy type 2 and type 3: a 2-year prospective study of bone health. Acta Paediatr 2023;112(12):2589-600. <http://dx.doi.org/10.1111/apa.16974>

Krupa. Real world evidence on the effectiveness of nusinersen within the national program to treat spinal muscular atrophy in Poland. Healthcare 2023;11(10):1515. <http://dx.doi.org/10.3390/healthcare11101515>

Lavie. Nonrespiratory complications of nusinersen-treated spinal muscular atrophy type 1 patients. Pediatr Pulmonol 2022;57(3):686-94. <http://dx.doi.org/10.1002/ppul.25795>

Lee. Impact of nusinersen on the health-related quality of life and caregiver burden of patients with spinal muscular atrophy with symptom onset after age 6 months. Muscle Nerve 2023;68(4):404-13. <http://dx.doi.org/10.1002/mus.27950>

Lemska. Efficacy of nusinersen treatment in type 1, 2, and 3 spinal muscular atrophy: real-world data from a single-center study. Neurol Int 2024;16(6):1266-78. <http://dx.doi.org/10.3390/neurolint16060096>

Li. Neurophysiological characteristics in type II and type III 5q spinal muscular atrophy patients: impact of nusinersen treatment. Drug Des Devel Ther 2024;18:953-65. <http://dx.doi.org/10.2147/DDDT.S449066>

Li. Analysis of the efficacy and adverse effects of nusinersen in the treatment of children with spinal muscular atrophy in China. Brain and Behavior 2024;14(7):e3630. <http://dx.doi.org/10.1002/brb3.3630>

Li. Disproportionality analysis of nusinersen in the Food and Drug Administration adverse event reporting system: a real-world postmarketing pharmacovigilance assessment. Pediatr Neurol 2024;158:71-8. <http://dx.doi.org/10.1016/j.pediatrneurol.2024.06.005>

Lilien. Patients' perceptions of nusinersen effects according to their responder status. J Clin Med 2024;13(12):3418. <http://dx.doi.org/10.3390/jcm13123418>

Lusakowska. Long-term nusinersen treatment across a wide spectrum of spinal muscular atrophy severity: a real-world experience. Orphanet J Rare Dis 2023;18(1):230. <http://dx.doi.org/10.1186/s13023-023-02769-4>

Maggi. Nusinersen safety and effects on motor function in adult spinal muscular atrophy type 2 and 3. J Neurol Neurosurg Psychiatry 2020;91(11):1166-74. <http://dx.doi.org/10.1136/jnnp-2020-323822>

Mendonca. Clinical outcomes in patients with spinal muscular atrophy type 1 treated with nusinersen. J Neuromuscul Dis 2021;8(2):217-24. <http://dx.doi.org/10.3233/JND-200533>

Mendonça. Real-world data from nusinersen treatment for patients with later-onset spinal muscular atrophy: a single center experience. J Neuromuscul Dis 2021;8(1):101-8. <http://dx.doi.org/10.3233/jnd-200551>

Mercuri. Nusinersen versus sham control in later-onset spinal muscular atrophy. *N Engl J Med* 2018;**378**(7):625-35. <http://dx.doi.org/10.1056/nejmoa1710504>

Meyer. Treatment expectations and perception of therapy in adult patients with spinal muscular atrophy receiving nusinersen. Eur J Neurol 2021;28(8):2582-95. <http://dx.doi.org/10.1111/ene.14902>

Mirea. Physical therapy and nusinersen impact on spinal muscular atrophy rehabilitative outcome. Front Biosci (Landmark Ed) 2022;27(6):179. <http://dx.doi.org/10.31083/j.fbl2706179>

Mirea. Combination therapy with nusinersen and onasemnogene abeparvovec-xioi in spinal muscular atrophy type I. J Clin Med 2021;10(23):5540. <http://dx.doi.org/10.3390/jcm10235540>

Modrzejewska. Nusinersen treatment of spinal muscular atrophy type 1: results of expanded access programme in Poland. Neurol Neurochir Pol 2021;55(3):289-94. <http://dx.doi.org/10.5603/PJNNS.a2021.0020>

Moshe-Lilie. Nusinersen in adult patients with spinal muscular atrophy. Neurology 2020;95(4):e413-6. <http://dx.doi.org/10.1212/wnl.0000000000009914>

Okamoto. Survey of patients with spinal muscular atrophy on the island of Shikoku, Japan. Brain Dev 2020;42(8):594-602. <http://dx.doi.org/10.1016/j.braindev.2020.05.004>

Osmanovic. Treatment satisfaction in 5q-spinal muscular atrophy under nusinersen therapy. Ther Adv Neurol Disord 2021;14:1756286421998902. <http://dx.doi.org/10.1177/1756286421998902>

Osmanovic. Nusinersen wearing-off in adult 5q-spinal muscular atrophy patients. Brain Sci 2021;11(3):367. <http://dx.doi.org/10.3390/brainsci11030367>

Osredkar. Children and young adults with spinal muscular atrophy treated with nusinersen. Eur J Paediatr Neurol 2021;30:1-8. <http://dx.doi.org/10.1016/j.ejpn.2020.11.004>

Pandey. Efficacy and safety of nusinersen among children with spinal muscular atrophy from North India: a prospective cohort study (NICE-SMA study). Eur J Paediatr Neurol 2025;54:42-9. <http://dx.doi.org/10.1016/j.ejpn.2024.12.001>

Pane. Nusinersen in type 1 spinal muscular atrophy: twelve-month real-world data. Ann Neurol 2019;86(3):443-51. <http://dx.doi.org/10.1002/ana.25533>

Pane. Type I SMA "new natural history": long-term data in nusinersen-treated patients. Ann Clin Transl Neurol 2021;8(3):548-57. <http://dx.doi.org/10.1002/acn3.51276>

Pane. Nusinersen efficacy data for 24-month in type 2 and 3 spinal muscular atrophy. Ann Clin Transl Neurol 2022;9(3):404-9. <http://dx.doi.org/10.1002/acn3.51514>

Pane. Type I spinal muscular atrophy patients treated with nusinersen: 4-year follow-up of motor, respiratory and bulbar function. Eur J Neurol 2023;30(6):1755-63. <http://dx.doi.org/10.1111/ene.15768>

Pechmann. Effect of nusinersen on motor, respiratory and bulbar function in early-onset spinal muscular atrophy. Brain 2023;146(2):668-77. <http://dx.doi.org/10.1093/brain/awac252>

Pechmann. Improved upper limb function in non-ambulant children with SMA type 2 and 3 during nusinersen treatment: a prospective 3-years SMArtCARE registry study. Orphanet J Rare Dis 2022;17(1):384. <http://dx.doi.org/10.1186/s13023-022-02547-8>

Pechmann. Improvements in walking distance during nusinersen treatment: a prospective 3-year SMArtCARE registry study. J Neuromuscul Dis 2023;10(1):29-40. <http://dx.doi.org/10.3233/JND-221600>

Peng. Motor function and compound muscle action potential amplitude in children with spinal muscular atrophy treated with nusinersen. Brain Dev 2025;47(1):104316. <http://dx.doi.org/10.1016/j.braindev.2024.104316>

Pera. Nusinersen in pediatric and adult patients with type III spinal muscular atrophy. Ann Clin Transl Neurol 2021;8(8):1622-34. <http://dx.doi.org/10.1002/acn3.51411>

Pinar. Exploring the influence of concurrent nutritional therapy on children with spinal muscular atrophy receiving nusinersen treatment. Children 2024;11(8):886. <http://dx.doi.org/10.3390/children11080886>

Rich. Neurofilament levels in CSF and serum in an adult SMA cohort treated with nusinersen. J Neuromuscul Dis 2022;9(1):111-9. <http://dx.doi.org/10.3233/JND-210735>

Sarikaya Uzan. The effect of nusinersen therapy on laboratory parameters of patients with spinal muscular atrophy. Neuropediatrics 2022;53(5):321-9. <http://dx.doi.org/10.1055/s-0042-1750719>

Scheijmans. Population-based assessment of nusinersen efficacy in children with spinal muscular atrophy: a 3-year follow-up study. Brain Commun 2022;4(6):fcac269. <http://dx.doi.org/10.1093/braincomms/fcac269>

Schneider. Motor unit number estimation in adult patients with spinal muscular atrophy treated with nusinersen. Eur J Neurol 2021;28(9):3022-9. <http://dx.doi.org/10.1111/ene.15005>

Simic. Total tau in cerebrospinal fluid detects treatment responders among spinal muscular atrophy types 1-3 patients treated with nusinersen. CNS Neurosci Ther 2022;30(3):e14051. <http://dx.doi.org/10.1111/cns.14051>

Simsek Erdem. Effect of nusinersen treatment on quality of life and motor function in adult patients with spinal muscular atrophy. Neuromuscul Disord 2024;36:28-32. <http://dx.doi.org/10.1016/j.nmd.2024.01.005>

Szabó. Efficacy of nusinersen in type 1, 2 and 3 spinal muscular atrophy: real world data from Hungarian patients. Eur J Paediatr Neurol 2020;27:37-42. <http://dx.doi.org/10.1016/j.ejpn.2020.05.002>

Tachibana. Safety and effectiveness of nusinersen, a treatment for spinal muscular atrophy, in 524 patients: results from an interim analysis of post-marketing surveillance in Japan. Int J Neurosci 2023;134(11):1185–97. <http://dx.doi.org/10.1080/00207454.2023.2251662>

Tachibana. Real-world safety and effectiveness of nusinersen, a treatment for spinal muscular atrophy, in 401 Japanese patients: results from an interim analysis of post-marketing surveillance. Int J Neurosci 2022;134(2):153–62. <http://dx.doi.org/10.1080/00207454.2022.2095270>

Trancho. Low bone mass in ambulatory spinal muscular atrophy: a proactive approach for an often-overlooked impairment. J Clin Med 2024;13(5):1336. <http://dx.doi.org/10.3390/jcm13051336>

Trucco. Therapeutic role of nusinersen on respiratory progression in pediatric patients with spinal muscular atrophy type 2 and nonambulant type 3. Neurology: Clinical Practice 2024;14(3):e200298. <http://dx.doi.org/10.1212/CPJ.0000000000200298>

Trucco. Assessing prevalence and characteristics of oro-bulbar involvement in children and adults with SMA type 2 and 3 using a multimodal approach. Dysphagia 2023;38(6):1568-80. <http://dx.doi.org/10.1007/s00455-023-10584-z>

Tscherter. Evaluation of real-life outcome data of patients with spinal muscular atrophy treated with nusinersen in Switzerland. Neuromuscul Disord 2022;32(5):399-409. <http://dx.doi.org/10.1016/j.nmd.2022.02.001>

van der Heul. Feeding and swallowing problems in infants with spinal muscular atrophy type 1: an observational study. J Neuromuscul Dis 2020;7(3):323-30. <http://dx.doi.org/10.3233/JND-190465>

van der Woude. Exploring functional strength changes during nusinersen treatment in symptomatic children with SMA types 2 and 3. Neuromuscular disorders : NMD 2024;41:1-7. <http://dx.doi.org/10.1016/j.nmd.2024.05.011>

Vazquez-Costa. Nusinersen in adult patients with 5q spinal muscular atrophy: a multicenter observational cohorts' study. Eur J Neurol 2022;29(11):3337-46. <http://dx.doi.org/10.1111/ene.15501>

Vidovic. Cognitive performance of adult patients with SMA before and after treatment initiation with nusinersen. BMC Neurol 2023;23(1):216. <http://dx.doi.org/10.1186/s12883-023-03261-z>

Vill. Newborn screening for spinal muscular atrophy in Germany: clinical results after 2 years. Orphanet J Rare Dis 2021;16(1):153. <http://dx.doi.org/10.1186/s13023-021-01783-8>

Wang. Long-term impact of nusinersen on motor and electrophysiological outcomes in adolescent and adult spinal muscular atrophy: insights from a multicenter retrospective study. J Neurol 2024;271(9):6004-14. <http://dx.doi.org/10.1007/s00415-024-12567-y>

Weaver. Quality of life outcomes according to differential nusinersen exposure in pediatric spinal muscular atrophy. Children (Basel) 2021;8(7):604. <http://dx.doi.org/10.3390/children8070604>

Weber. 'Reading the palm': a pilot study of grip and finger flexion strength as an outcome measure in 5q spinal muscular atrophy. Brain Dev 2024;46(5):189-98. <http://dx.doi.org/10.1016/j.braindev.2024.01.001>

Weststrate. Evolution of bulbar function in spinal muscular atrophy type 1 treated with nusinersen. Dev Med Child Neurol 2022;64(7):907-14. <http://dx.doi.org/10.1111/dmcn.15171>

Wohnrade. Health-related quality of life in spinal muscular atrophy patients and their caregivers: a prospective, cross-sectional, multi-center analysis. Brain Sci 2023;13(1):110. <http://dx.doi.org/10.3390/brainsci13010110>

Yao. Nusinersen effectiveness and safety in pediatric patients with 5q-spinal muscular atrophy: a multi-center disease registry in China. J Neurol 2024;271(8):5378-91. <http://dx.doi.org/10.1007/s00415-024-12442-w>

Yasar. Nusinersen therapy changed the natural course of spinal muscular atrophy type 1: what about spine and hip? J Child Orthop 2024;18(3):322-30. <http://dx.doi.org/10.1177/18632521241235028>

Zhang. Adverse events of nusinersen: a real-world drug safety surveillance study based on the FDA adverse event reporting system (FAERS) database. Expert Opin Drug Saf 2024; 10.1080/14740338.2024.2443796:1-8. <http://dx.doi.org/10.1080/14740338.2024.2443796>

# Supplementary Table S8: Risk of Bias for Randomised Controlled Trials (RCTs)

| **Author** | **Overall RoB rating** | **Bias arising from the randomization process** | **Effect of assignment to intervention** | **Effect of adhering to intervention** | **Missing outcome data** | **Measurement of the outcome** | **Selection of the reported result** |
| --- | --- | --- | --- | --- | --- | --- | --- |
| CHERISH, Mercuri 2018 | Some Concerns | Some Concerns | Low | Low | Low | Low | Some Concerns |
| EMBRACE, Acsadi 2021 | Some Concerns | Some Concerns | Not Applicable | Some Concerns | Low | Some Concerns | Some Concerns |
| ENDEAR; Finkel 2017 | Some Concerns | Some Concerns | Some Concerns | Low | Low | Low | Low |
| SUNFISH, Mercuri 2022 | Some Concerns | Some Concerns | Low | Not Applicable | Low | Low | Low |
| SUNFISH, Mercuri 2023 | Low | Low | Low | Low | Low | Low | Low |
| SUNFISH 2023 | Some Concerns | Low | Low | Not Applicable | Some Concerns | Low | Low |

# Supplementary Table S9: Risk of bias for non-randomised trials (ROBINS-1)

| **Author** | **Overall RoB rating** | **Bias due to confounding** | **Bias in selection of participants into the study** | **Bias in classification of interventions** | **Bias due to deviations from intended interventions** | **Bias due to missing data** | **Bias in measurement of outcomes** | **Bias in selection of the reported result** |
| --- | --- | --- | --- | --- | --- | --- | --- | --- |
| Alves 2021 | Critical | Serious | Critical | Serious | Low | Low | Moderate | serious |
| Chiriboga 2024 | Serious | Serious | Low | Low | Low | Low | Serious | Low |
| Chiriboga 2016 | Serious | Serious | Low | Low | Low | Serious | Moderate | Low |
| Colot 2024 | Serious | Moderate | Serious | Moderate | Low | No information | Serious | Low |
| Coratti 2024 | Serious | Serious | Serious | Low | Serious | Low | Moderate | Serious |
| Duan 2022 | Critical | Serious | Critical | Low | low | Serious | Serious | Moderate |
| Freigang 2021b | Moderate | Moderate | Low | Low | Low | No information | Low | Low |
| Gaffar 2022 | Serious | Serious | Low | Low | Low | Low | Moderate | Moderate |
| García de la Banda 2021 | Critical | Critical | Critical | Low | Low | low | Moderate | Moderate |
| Kokaliaris 2024 | Serious | Moderate | Moderate | Low | Low | Low | Serious | Low |
| Kroksmark 2023 | Serious | Serious | Serious | Moderate | No information | Low | Serious | Moderate |
| Krupa 2023 | Serious | Serious | Serious | Low | Low | Low | Low | Moderate |
| Lee 2022 | Moderate | Moderate | Moderate | Low | No information | Low | Moderate | Moderate |
| Li 2024 | Serious | Serious | Serious | Low | Low | Low | Moderate | Moderate |
| Mendonca 2020b | Serious | Serious | Low | Moderate | Low | Low | Serious | Serious |
| Mirea 2022 | Serious | Serious | No information | Serious | Low | Low | Moderate | Serious |
| Mirea 2021 | Serious | Serious | No information | Serious | Low | Low | Moderate | Low |
| Pandey 2025 | Serious | Serious | Low | Low | Low | Low | Serious | Low |
| Pane 2022 | Critical | Critical | Serious | Low | Low | Low | Moderate | Low |
| Pechmann 2023 | Serious | Moderate | Low | Low | Moderate | Serious | Serious | Moderate |
| Rich 2022 | Serious | Serious | Low | Low | Low | Low | Moderate | Low |
| Schneider 2021 | Critical | Critical | Serious/Critical | Low | Low | Low | Moderate | Moderate |
| SHINE (Dunaway Young 2023) | Serious | Low | Low | Low | Low | Low | Serious | Serious |
| Trancho 2024 | Critical | Critical | Serious/Critical | Low | Low | low | Moderate | Moderate |
| Trucco 2024 | Serious | Moderate | Serious | Moderate | Low | Low | Moderate | Serious |
| Trucco 2023 | Serious | Serious | Serious | Moderate | Low | Low | Moderate | Moderate |
| Van der Heul 2020 | Serious | Serious | Low | Moderate | Low | No information | Moderate | Low |
| Vazquez-Costa 2022 | Serious | Serious | Serious | Low | Low | Low | Moderate | Serious |
| Weaver 2021 | Critical | Serious | Critical | Moderate | Serious (or moderate) | Critical | Serious | Serious |

# Supplementary Table S10: Risk of Bias of single arm studies

| **Author and year** | **Overall RoB rating** | **Were there clear criteria for inclusion in the case series?** | **Was the condition measured in a standard, reliable way for all participants included in the case series?** | **Were valid methods used for identification of the condition for all participants included in the case series?** | **Did the case series have consecutive inclusion of participants?** | **Did the case series have complete inclusion of participants?** | **Was there clear reporting of the demographics of the participants in the study?** | **Was there clear reporting of clinical information of the participants?** | **Were the outcomes or follow up results of cases clearly reported?** | **Was there clear reporting of the presenting site(s)/clinic(s) demographic information?** | **Was statistical analysis appropriate?** |
| --- | --- | --- | --- | --- | --- | --- | --- | --- | --- | --- | --- |
| AlRuthia 2021 | Low | Yes | Unclear | Unclear | No | No | Yes | Yes | Unclear | Unclear | Yes |
| AlTawari 2024 | High | Yes | Yes | Yes | Unclear | Unclear | Yes | Yes | Yes | Yes | Yes |
| Aragon-Gawinska 2019/20 | High | Yes | Yes | Yes | Unclear | Unclear | Yes | Yes | Yes | No | Yes |
| Arnold 2021 | High | Yes | Yes | Yes | Unclear | Unclear | Yes | Yes | Yes | No | Yes |
| Arslan 2023 | High | Yes | Yes | Yes | No | No | Yes | Yes | Yes | No | Yes |
| Audic 2020 | High | Yes | Yes | Yes | Not Applicable | No | Yes | Yes | Yes | No | Yes |
| Audic 2023/24 | High | Yes | Yes | Yes | Not Applicable | Unclear | Yes | Yes | Unclear | Yes | Yes |
| Axente 2022 | High | Yes | Unclear | Yes | no | Unclear | yes | Yes | Yes | No | Yes |
| Belancic 2023 | High | Yes | Yes | Unclear | Unclear | Yes | Yes | Yes | Yes | No | Yes |
| Berti 2022 | High | Yes | Yes | Yes | Unclear | Unclear | No | No | Yes | No | Yes |
| Bjelica 2023 | High | Yes | Yes | Yes | Yes | Yes | Yes | Yes | No | No | Yes |
| Brakemeier 2021 | High | Yes | no | Yes | Unclear | No | Yes | Yes | No | No | Yes |
| Brakemeier 2024 | High | Yes | Yes | Yes | Unclear | Unclear | Yes | Yes | No | No | Yes |
| Cavaloiu 2024 | Low | No | Unclear | Unclear | Unclear | Unclear | No | No | No | Yes | Yes |
| Cavaloiu 2024b | High | Yes | Yes | Yes | Unclear | unclear | Yes | Yes | Yes | Yes | Yes |
| Chacko 2022 | High | Yes | Yes | Yes | Yes | Yes | Yes | Yes | Yes | No | No |
| Chen 2024 | High | Yes | Yes | Yes | Yes | Yes | Yes | Yes | No | Yes | Yes |
| JEWELFISH: (Chiriboga 2023) | High | Yes | Yes | Yes | Unclear | Unclear | Yes | Yes | Yes | No | Yes |
| Cho 2023 | High | Yes | Yes | Yes | Unclear | No | unclear | Yes | Yes | no | Yes |
| Coratti 2021 | High | Yes | Yes | Yes | Yes | Yes | Yes | Yes | Yes | no | Yes |
| Cornell 2024 | High | Yes | Yes | Yes | Unclear | Unclear | Yes | Yes | Yes | No | Yes |
| NURTURE: (Crawford 2023) | High | Yes | Yes | Yes | Yes | Yes | Yes | Yes | Yes | Yes | Yes |
| C2 C12: (Darras F2019) | High | Yes | Yes | Yes | Unclear | Yes | Yes | Yes | Yes | No | Yes |
| De Wel (2020/21) | High | Yes | Yes | Yes | No | No | Yes | Yes | Yes | Unclear | Yes |
| Duong (2021) | High | Yes | Yes | Yes | Yes | yes | Yes | Yes | Yes | No | Yes |
| Edel (2021) | High | Yes | Yes | Yes | Yes | Unclear | Yes | Yes | Yes | No | Yes |
| SM201 (European Medicines Agency 2017) | High | Yes | Yes | Yes | Unclear | Unclear | Unclear | Unclear | Yes | Unclear | Yes |
| European Registries (NICE 2020) | High | Yes | Yes | Yes | Yes | Yes | No | Yes | Yes | Unclear | Yes |
| Fainmesser 2022 | High | Yes | Yes | Yes | Unclear | Unclear | Yes | Yes | Yes | No | Yes |
| Finkel 2021 | High | Yes | Yes | Yes | Unclear | Unclear | Yes | Yes | Yes | Yes | Yes |
| Freigang 2021 | High | Yes | Yes | Yes | Unclear | Unclear | Yes | Yes | Yes | Yes | Yes |
| Freigang 2023 | High | Yes | Yes | Yes | Unclear | Unclear | Yes | Yes | Yes | Yes | Yes |
| Gaboli 2025 | High | Yes | Yes | Yes | Unclear | Unclear | Unclear | Yes | Yes | Yes | Yes |
| Goedeker 2021 | Low | Yes | Unclear | Unclear | Yes | Yes | No | No | Yes | No | Unclear |
| Gonski 2023 | High | Yes | Yes | Yes | yes | yes | yes | yes | yes | Yes | yes |
| Govoni (2024) | High | Yes | Yes | Yes | Unclear | Unclear | Yes | Yes | Yes | No | Yes |
| Gunther (2024) | High | Yes | Yes | Yes | Yes | Yes | Yes | Yes | Yes | No | Yes |
| Hagenacker (2020) | High | Yes | Yes | Yes | Yes | Yes | Yes | Yes | Yes | Yes | Yes |
| Hahn (2022) | High | Yes | Yes | Yes | No | No | Yes | Yes | Yes | No | Yes |
| Huang (2024) | High | Yes | Yes | Yes | Unclear | Unclear | Yes | Yes | Yes | Yes | Yes |
| Ip (2024) | High | Yes | Yes | Yes | Yes | Yes | Yes | Yes | Yes | Yes | Yes |
| Jiang (2024) | High | Unclear | Yes | Yes | Unclear | Unclear | Yes | Yes | Yes | Yes | Yes |
| Jiang (2024b) | Low | No | No | No | No | No | Unclear | No | Yes | Yes | Yes |
| Kariyawasam (2020/21) | High | Yes | Yes | Yes | Unclear | Unclear | Yes | Yes | Yes | No | Yes |
| Kölbel (2022) | High | Yes | Yes | Yes | Unclear | Unclear | No | Yes | Yes | No | yes |
| Konersman (2021) | High | Yes | Unclear | Unclear | Yes | Yes | Yes | Yes | Yes | Yes | Yes |
| Kotulska (2022) | High | Yes | Yes | Yes | Yes | Yes | Yes | Yes | Yes | Unclear | Yes |
| Kwon (2022) | High | Yes | Yes | Yes | Unclear | Unclear | Yes | Yes | Yes | Yes | Yes |
| Landfeldt (2023) | High | Yes | Yes | Yes | Unclear | Unclear | Yes | Yes | Yes | Yes- database described | Yes |
| Lavie (2021) | High | Unclear | Yes | Yes | Unclear | Unclear | Yes | Yes | Yes | Yes | Yes |
| Lee (2023) | High | Yes | Yes | Yes | Unclear | Unclear | Yes | Yes | No | Unclear | Yes |
| Lemska (2024) | High | Yes | Yes | Yes | Unclear | No | Yes | No | Yes | No | Yes |
| Li (2024a) | High | Yes | Yes | Yes | Yes | Yes | Yes | Yes | Yes | Yes | Yes |
| Li (2024b) | Low | no | no | No | no | No | yes | no | yes | no | Unclear |
| Lillien (2024) | High | Yes | Yes | Yes | Unclear | Unclear | Yes | Yes | Yes | Yes | Yes |
| Lusakowska (2023) | High | Yes | Yes | Yes | Yes | Yes | Yes | Yes | Yes | No | Yes |
| Maggi (2020) | High | Yes | Yes | Yes | Unclear | Unclear | Yes | Yes | Yes | No | Yes |
| FIREFISH: (Masson 2022) | High | Yes | Yes | Yes | Unclear | Unclear | Yes | Yes | Yes | No | Yes |
| Mendonca (2021) | High | Yes | Yes | Yes | Unclear | Unclear | Yes | Yes | Yes | No | Yes |
| Meyer (2021) | High | Yes | Yes | Yes | Unclear | Unclear | Yes | Yes | Yes | No | Unclear |
| Modrzejewska (2021) | High | Yes | Yes | Yes | Unclear | Unclear | Yes | Yes | Yes | Yes | Yes |
| Moshe-Lilie (2020) | High | yes | Yes | Yes | Unclear | Unclear | Yes | Yes | Yes | Yes | No |
| Okamoto (2020) | High | Yes | No | Yes | No | Unclear | Yes | Yes | No | Yes | Yes |
| Osmanovic (2021) | High | Yes | Yes | Yes | Yes | Yes | Yes | Yes | No | Yes | Yes |
| Osmanovic (2021b) | High | Yes | Yes | Yes | Unclear | Unclear | Yes | Yes | Yes | Yes | Yes |
| Osredkar (2020/21) | High | Yes | Yes | Yes | Yes | Yes | Yes | Yes | Yes | Yes | Yes |
| Pane (2024) | Low | yes | yes | yes | unclear | unclear | yes | yes | No | no | yes |
| Pane (2019) | Low | Yes | Unclear | Unclear | Unclear | Unclear | No | Yes | No | Unclear | Yes |
| Pane (2023) | High | Yes | Unclear | Unclear | unclear | unclear | No | Yes | Yes | Yes | Yes |
| Pane (2021) | High | No | Unclear | Unclear | Unclear | Unclear | No | Yes | No | No | Yes |
| Pechmann (2022) | High | Yes | Yes | Yes | Yes | Yes | No | Yes | Yes | No | Yes |
| Pechmann (2023b) | High | Yes | Yes | Yes | Yes | yes | No | Yes | Yes | No | Yes |
| Peng (2025) | High | Yes | Yes | Yes | Unclear | Unclear | Yes | Yes | Yes | Yes | Yes |
| Pera (2021) | High | yes | yes | yes | yes | yes | yes | yes | yes | yes | yes |
| Pinar (2024) | High | Yes | Yes | Yes | Unclear | Unclear | Yes | Unclear | No | Yes | Yes |
| RAINBOWFISH: (EMA 2023) | High | Yes | Yes | Yes | Yes | Yes | Yes | Yes | Yes | Unclear | No |
| Sarikaya (2022) | High | Yes | Yes | Yes | Yes | Yes | Yes | Yes | Yes | Yes | Yes |
| Scheijmans (2022) | High | Yes | Yes | Yes | Yes | Yes | Yes | Yes | Yes | No | Yes |
| Simic(2022) | High | no | Yes | Yes | Yes | unclear | No | Yes | Yes | Yes | Yes |
| Simsek (2024) | High | no | yes | yes | Unclear | Unclear | yes | yes | yes | yes | yes |
| Sitas (2024) | High | unclear | Yes | Yes | Yes | Yes | Yes | Yes | Yes | Yes | Unclear |
| Szabo (2020) | High | Yes | Yes | Yes | yes | Yes | Yes | Yes | Yes | Yes | Yes |
| Tachibana (2023) | High | Yes | Yes | Yes | Yes | Yes | Yes | Yes | Yes | Yes | Yes |
| Tachibana (2022) | High | Yes | Yes | Yes | Yes | Yes | Yes | Yes | Yes | Yes | Yes |
| Tscherter (2022) | High | Yes | Yes | Yes | Yes | Yes | Yes | Yes | Yes | Yes | Yes |
| van der Woude (2024) | High | Yes | Yes | Yes | Yes | Yes | Yes | Yes | No | Yes | Yes |
| Vidovic (2023) | High | Yes | Yes | Yes | Unclear | Unclear | Yes | Yes | Yes | Yes | Yes |
| Vill (2021) | High | Yes | Yes | Yes | Yes | Yes | Yes | Yes | Yes | Yes | Unclear |
| Wang (2024) | High | Yes | Yes | Yes | Unclear | Unclear | Yes | Yes | Yes | Yes | Yes |
| Weber (2024) | High | Yes | Yes | Yes | Unclear | Yes | Yes | Yes | Yes | Yes | Yes |
| Weststrate (2022) | High | Yes | Yes | Yes | Yes | Yes | Yes | Yes | Yes | Yes | Yes |
| Wohnrade (2023) | High | Yes | Yes | Yes | Yes | Yes | Yes | Yes | Yes | Yes | Yes |
| Yao (2024) | High | Yes | Yes | Yes | Yes | Yes | Yes | Yes | Yes | Unclear | Yes |
| Yasar (2024) | High | Yes | Yes | Yes | Unclear | Unclear | Yes | Yes | No | Not applicable | Yes |
| Yu (2024) | High | Yes | Yes | Yes | Yes | Yes | Yes | Yes | Yes | Yes | Yes |
| Zhang (2024) | Low | Not Applicable | Not Applicable | Not Applicable | No | No | Yes | Yes | Yes | Not applicable | yes |

# Supplementary Table S11: Motor function outcomes

| **Author name and year** | **Results of motor function** |
| --- | --- |
| Alruthia 2021 | n/a |
| AlTawari 2024 | Pediatric Cohort:  CHOP-INTEND: Evaluated in 14 patients; 13/14 showed clinically meaningful or considerable improvement in functional motor performance. 71% showed clinically meaningful improvement (≥4 points), average increase of 13.7 points.  HFMSE: Evaluated in 6 patients; 67% showed clinically meaningful improvement (≥3 points), average increase of 3.5 points.  Overall, 70% showed clinically meaningful improvement, 20% showed considerable improvement.  Adult Cohort:  RULM: Evaluated in 18 patients; 72% showed clinically meaningful improvement (≥2 points), average increase of 7.3 points.  17% showed considerable improvement, 1 patient showed slight decline due to missed doses.  Clinical Presentations and Motor Milestones  Pediatric Cohort:  11 diagnosed in infancy; 10 showed improvement, 6 could sit independently.  9 diagnosed after 12 months; 3 wheelchair-bound, 2 assisted walking, 4 unassisted walking.  Adult Cohort:  14 non-ambulant at initial presentation.  Patient 26 showed significant improvement in HFMSE and six-minute walk test scores. |
| Alves 2021 | All available maximum ulnar CMAP data for these cohorts were plotted. This data demonstrates an association between SMN2 copy number and maximum ulnar CMAP amplitude, indicating that patients with lower SMN2 copies are more likely to demonstrate objective evidence of denervation early in life  Findings: These findings validate previous evidence showing that serum pNf-H and Nf-L levels are increased in SMA patients and demonstrate that these changes are associated with SMN2 copy number and inversely associated with maximum ulnar CMAP amplitude in the first 3 years of life.  CMAP Values: There were significant differences in CMAP values among SMA patients with different SMN2 copies after correction for age (p < 0.0001), where patients with 4 SMN2 copies had higher CMAP maximum amplitude as compared with patients with 2 or 3 SMN2 copies, and patients with 3 SMN2 copies had higher CMAP maximum amplitude as compared with patients with 2 SMN2 copies (p < 0.0001 for all comparisons)  These models suggest that nusinersen treatment was associated with decreased NF levels, while onasemnogene abeparvovec was associated with increased NF levels in SMA subjects in the first 3 years of life. However, both nusinersen and onasemnogene abeparvovec therapies were associated with increased CMAP maximum amplitude, suggesting less denervation in treated patients when compared with untreated SMA patients with either 2 or 3 SMN2 copies |
| Aragon-Gawinska 2020 | N=47 completed 14 months of treatment and sitting status was known  N=2 died one after five injections (month 6) and one after six injections (month 10)  N=1 withdrawn due to lack of motor gain (month 2)  After month 14 month of treatment n=15 of 47 patients were able to sit independently N=5 patients were able to sit after only 6 months of treatment  Sitters had statistically significantly higher baseline scores in HINE-2 (2 vs 1, p<0.01) and CHOP-INTEND (35.5 vs 26.5, p<0.05) and larger changes in the median HINE-2 score after 6 months of treatment than non-sitters (3 vs 1,p<0.05)  Patients who improved by 2 or more points at month 6 were three times more likely to be sitters at month 14 than those who did not |
| Arnold 2021 | impact of nusinersen on CMAP Decrement and CMAP Amplitude:  Spinal accessory CMAP decrement during RNS and CMAP amplitude were analysed cross-sectionally (all participants with assessment at baseline and/or month 14) and longitudinally (only participants with assessments at baseline and month 14), pre-14 months and post-14 months of nusinersen treatment  There were no significant changes in the severity of CMAP amplitude decrement following treatment  After 14 months of nusinersen treatment, 9 of 15 (60%) participants had >10% CMAP amplitude decrement  Longitudinal CMAP amplitude was significantly increased at 14 months compared with baseline, and increases in CMAP were seen in all participants except one non-ambulatory participant who showed a reduction from 2.0 mV at baseline to 1.8 mV at 14 months  In two participants studied longitudinally (both ambulatory), CMAP amplitude decrement worsened (baseline to 14 months: +4.4% to −17.8% and −11.6% to −21.2%), but both of these participants showed increases in CMAP amplitude (baseline to 14 months: 5.4 mV to 6.5 mV and 3.4 mV to 4.3 mV, respectively)  In the ambulatory participants who were studied longitudinally, there was a variable increase in 6MWT distance at 14 months compared with baseline (baseline to 14 months: 374 m to 380 m, 404 m to 500 m, and 121 m to 132 m; paired t-test; p=0.3261)  Impact of Age, Disease Duration, and Ambulatory Ability on CMAP Decrement and Amplitude:  Correlation analysis between CMAP decrement versus both age and disease duration demonstrated no significant associations  The mean SMN2 copy number in participants with abnormal decrement (−10% or more) after 14 months of nusinersen treatment was similar to those with normal RNS findings (abnormal RNS decrement: 3.4±0.5 SMN2 copies vs normal RNS: 3.6±0.5 SMN2 copies; unpaired t-test, p=0.4829)  CMAP decrement was similar in non-ambulatory and ambulatory participants (all assessed following 14 months of nusinersen treatment)  CMAP amplitude did not show significant correlations with age or disease duration, but CMAP was significantly different between ambulatory and non-ambulatory patients (all at 14 months of Nusinersen treatment)  CMAP amplitude was also significantly different between ambulatory and non-ambulatory patients at baseline prior to Nusinersen treatment (ambulatory, n=7: mean=7.2±2.8 mV vs non-ambulatory, n=10: mean=2.2±2.0 mV; unpaired t-test, p=0.0005)  Muscle Strength and Function are Associated with CMAP Decrement in Ambulatory Participants with SMA:  To understand the associations between NMJ transmission defects and function in a uniform population, correlation coefficients were analysed in ambulatory participants after 14 months of Nusinersen treatment  CMAP decrement showed associations with measures of upper and lower limb muscle strength on MVICT  CMAP decrement was associated with upper limb function as assessed by RULM  CMAP decrement was associated with 6MWT distance and fatigue on the 6MWT as quantified as a ratio between the distance walked for 6 min vs 1 min of the test  CMAP decrement showed no significant associations with ulnar nerve motor unit electrophysiological testing including abductor digiti minimal CMAP amplitude (r2=0.1094, p=0.3846), average SMUP (r2=0.3053, p=0.1229) or MUNE (r2=0.3715, p=0.0814)  Figure 2 CMAP Decrement and Amplitude after 14 Months of Nusinersen Treatment:  Cross-sectional analysis of CMAP decrement demonstrated no significant difference in assessments after 14 months of Nusinersen treatment (−11.9%±8.3%, n=15, nine ambulatory) vs assessments prior to treatment (−14.2%±11.5% decrement, n=17, seven ambulatory) (unpaired t-test, p=0.5202)  Longitudinal assessment of a subset of participants (n=8, three ambulatory) demonstrated that CMAP amplitude decrement following 14 months of treatment (−13.9%±6.7%) was similar to baseline (−16.9%±13.4%) (paired t-test, p=0.5863)  Cross-sectional analysis of CMAP amplitude demonstrated no significant difference after 14 months of treatment (4.3±2.4 mV, n=15, nine ambulatory) vs assessments prior to treatment (4.3±3.4 mV, n=17, seven ambulatory) (unpaired t-test, p=0.9871)  CMAP amplitude was significantly improved with Nusinersen when analysed in a subset of participants (n=8, three ambulatory) that underwent assessment prior to treatment (2.6±2.0 mV) and after 14 months of Nusinersen (4.2±2.8 mV) (paired t-test, p=0.0383)  CMAP refers to compound muscle action potential and RNS refers to repetitive nerve stimulation.  Figure 3: CMAP Decrement at 14 Months of Nusinersen Treatment:  CMAP decrement was not associated with age (r²=0.0256, p=0.9364, n=15) or disease duration (r²=0.03323, p=0.5328, n=14)  When stratified by ambulatory ability, CMAP decrement was not significantly different in ambulatory participants (−11.8%±9.5%, n=9) versus non-ambulatory participants (11.9%±7.0%, n=6) following 14 months of Nusinersen treatment (unpaired t-test, p=0.9943)  CMAP decrement for analyses was obtained in participants at 14 months of Nusinersen treatment  R² is the square of Pearson correlation coefficients  CMAP refers to compound muscle action potential  Figure 4: CMAP Amplitude at 14 Months of Nusinersen Treatment:  CMAP amplitude did not significantly correlate with age (r²=0.1681, p=0.1290, n=15) or disease duration (r²=0.05957, p=0.4004, n=14)  When stratified by ambulatory ability, CMAP amplitude was significantly higher in ambulatory participants (5.3±2.1 mV, n=9) versus non-ambulatory participants (2.7±2.1 mV, n=6) following 14 months of Nusinersen treatment (unpaired t-test, p=0.0326)  CMAP amplitudes for analyses were obtained in participants at 14 months of Nusinersen treatment  R² is the square of Pearson correlation coefficients  CMAP refers to compound muscle action potential  Figure 6 CMAP Decrement Associations with Upper Limb and Lower Limb Functions:  Revised Upper Limb Module score: Significant correlation (r²=0.7631, p=0.0021)  6MWT distance: Significant correlation (r²=0.5012, p=0.0328)  Fatigue on the 6MWT (ratio of the distance walked during min 6 divided by the distance walked during min 1): Significant correlation (r²=0.6410, p=0.0095)  Hammersmith Functional Rating Scale Expanded score: Not significantly correlated (r²=0.1679, p=0.2735)  These correlations were observed in 9 ambulatory participants (four women and five men) at 14 months of treatment with Nusinersen. CMAP refers to compound muscle action potential. R² is the square of Pearson correlation coefficients. 6MWT refers to the 6 min walk test  Figure 5 CMAP Decrement Associations with Upper Limb and Lower Limb Strengths:  Hand grip: Significant correlation (r²=0.6599, p=0.0143)  Key pinch: Significant correlation (r²=0.7080, p=0.0088)  Elbow flexion: Significant correlation (r²=0.6893, p=0.0107)  Elbow extension: Significant correlation (r²=0.7201, p=0.0077)  Shoulder abduction: Significant correlation (r²=0.9064, p=0.0003)  Hip flexion: Significant correlation (r²=0.6863, p=0.0111)  Knee extension: Not significantly correlated (r²=0.2623, p=0.1944)  Knee flexion: Not significantly correlated (r²=0.2865, p=0.1716)  These correlations were observed in 8 ambulatory participants (four women and four men) at 14 months of treatment with Nusinersen. CMAP refers to compound muscle action potential. R² is the square of Pearson correlation coefficients |
| Arslan 2023 | median ALSFRS-R score was 36 (range 28–45) at t0 and increased to 37 (range 28–47) at t2. The ALSFRS-R score in the whole group analysis increased from baseline by a median of 1 point (range 0–4) at t2 (p < 0.001).  MRC-SS in the whole group increased from baseline with a range between 0 and 2 at t2 (p = 0.001).  At t2, SMA type 3 walkers had the highest score increase in all assessments. The median increase was 6 points by HFMSE, 1.5 points by ALSFRS-R, and 1 point by MRC-SS.  SMA Type 2 Patients: Both ALSFRS-R score and MRC-SS did not show any statistically significant difference in SMA type 2 patients comparing to baseline (p = 0.31 and p > 0.99, respectively).  In the whole group, a positive correlation between the change of ALSFRS-R and HFMSE scores from baseline to t2 (r: + 0.48, p = 0.005), but no correlation was observed between MRC-SS and HFMSE score changes (r: + 0.24, p = 0.17).  The median 6MWT distance at t0 was 377 (range 60–523) and increased to 410 (range 69–558) at t2 (p = 0.008).  Based on HFMSE criteria, 23 patients responded to Nusinersen treatment.  two ambulatory SMA type 3 patients who have high baseline scores (baseline scores were 65 and 63, respectively) were accepted as “responder” due to a 30-m increase in 6MWT distance despite the lack of a 3-point increase in HFMSE score.  78% (n = 25) of all SMA patients showed clinically meaningful improvement by HFMSE or 6MWT  except for one patient who rejected the treatment due to lumbar puncture difficulties the maintenance treatment was continued in responders.  In the cohort, SMA type 3 patients responded better to Nusinersen treatment (p = 0.012), and in post hoc analyses, it was observed that the difference between SMA type 2 and type 3 was significant due to SMA type 3 sitters.  Subgroup: HFMSE scores at t3 and t4 in 22 patients with SMA type 3 as responders and continued to maintenance treatment. One patient who received Nusinersen by conventional lumbar puncture and improved from baseline by 6 HFMSE points at t2 rejected the maintenance treatment due to the invasive procedure. No significant change was observed by HFMSE score after t3; therefore, it was concluded that the patients remained stable after this time point |
| Audic 2020 | Patients’ motor function skills at T0 and Y1 were compared using the HINE-2 (20 patients with total scores only, 17 with detailed sub scores) and/or CHOP-INTEND (n = 14) scales.  Twelve patients were assessed using both methods, two patients were evaluated using MFM 20 only, and six patients were evaluated using different methods at T0 and Y1.  There was a statistically significant increase in the total HINE-2 scores between T0 and Y1 (p < 0.001).  Analysis of the sub scores revealed improvements in head control, sitting, voluntary grasp, ability to kick supine, rolling, crawling, and standing milestones, but not for walking.  The 14 patients who were assessed with the CHOP-INTEND scale also showed overall improvement, with the mean total score increasing from 35.1 to 50.3.  Motor Function Evaluation in Children Older Than 2 Years (n = 93):  For these patients, motor function assessments at T0 and Y1 were mainly performed using the MFM scale (n = 68) (73% of the age subgroup; 5 with SMA type 1c and 63 with type 2 SMA).  The 25 remaining patients were evaluated with either the HINE-2 or CHOP-INTEND methods.  Motor function improved slightly overall after 1 year of treatment (the median total MFM score increased by 6).  In the different domains, there were significant improvements in the median scores in D2 (+ 9 percentage points, p < 0.001) and D3 (+ 7 percentage points, p < 0.001) but not in D1 (ambulation, − 1 percentage point).  These improvements in D2 and D3 are clinically relevant in terms of functional benefits.  In terms of age groups, the increases in MFM scores were more substantial in the children under 6 years old at treatment onset (n = 33, + 8 points overall, + 12 points in D2 and + 9 points in D3; p ≤ 0.001 in each case) than in those older than 6 years at treatment onset (+ 4 points overall, p = 0.099; + 6 points in D2, p = 0.457; and + 5 points in D3, p = 0.04) |
| Audic 2023 | Motor Function:    After 3 years of treatment, 93% of patients gained new motor skills. Two patients lost a motor ability and one patient remained stable.    Orthopaedic Outcomes:    Nusinersen did not prevent patients with two copies of the SMN2 gene from having early orthopaedic deformities. At baseline, three of 20 (15%) patients had scoliosis. At 3 years of treatment completion, all (100%) patients with two copies of SMN2 had scoliosis and 18 of 19 (95%) patients had a corset    After 3 years of treatment:  • 93% of patients gained new motor skills.  • Two patients (patients #10 and #33) lost a motor ability.  • One patient (patient #34) remained stable.  Before starting treatment:  • Six of 20 patients with two copies of SMN2 could hold their head.  • The other 14 patients with two copies of SMN2 had no motor ability.  Three years after starting treatment:  • Of the 20 patients with two copies of the SMN2 gene:  o 19 (95%) were able to control their head.  o 17 (85%) were able to sit.  o Seven (35%) were able to crawl or roll over.  o Four (20%) were able to stand with help.  o Eight (40%) were able to drink on their own.  o Ten (50%) were able to eat on their own.  • The only patient unable to hold his head after 3 years of treatment was classified as having SMA type 1c with two copies of SMN2, who started treatment at 24 months of age.  After 3 years on Nusinersen:  • All patients with three copies of SMN2 had acquired head control versus 32 of 37 patients (86%) at baseline.  • Nine additional patients (36/37; 97%) were able to sit compared to baseline.  • 15 additional patients (29/36; 78%) were able to crawl or roll over.  • At baseline, standing was achieved by four out of 37 patients (11%), including one patient (3%) who was able to stand without assistance.  • After 3 years of treatment completion, 23 of the 37 patients (62%) achieved standing, including 12 (32%) without help.  • Overall, 11 (30%) were able to walk and five (14%) of them without assistance at the 3-year follow-up, versus one (3%) at baseline.  • Regarding autonomy, six of the 37 patients were able to eat and/or drink alone at the initial visit compared to 35 of 36 (97%) at the final visit. |
| Axente 2022 | At the start of treatment (T0), the SMA type I group consisted of 11 non-sitter patients.  After 10 administrations of Nusinersen (T26), 45.45% became sitters, with their CMAP amplitude increasing from 0.53 ±0.23 to 1.85 ±1.05.  The other 54.55% patients who remained non-sitters showed an increase in CMAP from 0.26 ±0.23 to 1.19 ±0.66.  At T0, the type II SMA group consisted of 16 sitters. At T26, 25% of the patients became walkers, with their CMAP amplitude increasing from 2.73 ±2.08 to 3.65 ±1.55.  The other 75% of patients who remained sitters showed an increase in CMAP from 0.99 ±0.91 to 1.73 ±1.18.  The SMA type III group consisted of four sitters and three walkers, both at T0 and T26. The CMAP of the sitters increased from 1.25 ±0.55 to 2.05 ±0.66, while the CMAP of the walkers increased from 2.73 ±2.38 to 3.66 ±2.85.  There is a significant increase in CHOP, consistent with CMAP in 5 patients for SMA type 1.  In SMA type 2 patients, HFMSE is generally stationary, with only two of them having significant variations in CMAP (above the group average).  In SMA type 3 patients, CMAP/HFMSE show a stationary trend between T0 and T26.  In SMA type 1, CHOP increase was observed between T0 and T26.  In SMA type 2, there were two patients with a better outcome than the others who had differences between T0 and T26, and whose evaluations slightly increased.  In SMA type 3, no regression was registered, but very close values were obtained in both evaluations, with a single patient having the same HFMSE score at T0/T26.  CMAP Amplitude  The most significant increase in distal CMAP amplitude was recorded in SMA type 1 patients, statistically correlated with scores on CHOP scales (p < 0.001).  Most of the SMA type 2 patients started treatment long after symptom onset (13–96 months) and presented stationary CMAP amplitude and HFMSE scores.  The majority (four out of seven patients) of SMA type 3 patients were non-ambulant at T0. They started treatment several years after losing gait, remaining non-ambulant at T26. Low values of CMAP amplitude at the distal ulnar nerve were observed.  Correlation  CMAP amplitude is shown to be significantly (p < 0.005) strongly correlated (r = 0.667) with motor function evolution only in SMA type 1. For SMA types 2 and 3 there are no significant statistical correlations |
| Belancic 2023 | Motor Function Improvement:  14 SMA type 1 patients improved their CHOP INTEND motor function by ≥4 points after 5 doses of Nusinersen (33.3% ,2/6 of SMA type 1 patients on MV support and 100%, 8/8 of those who were not on MV. The change from baseline was significant at the time of the evaluation just before the 5th injection and remained significant.  A negative correlation (p = 0.001, rS = _0.77, 95% CI: _0.923– _0.403) between the age at treatment initiation and the change from baseline in motor function.  SMA Type 2 Patients:  2/6 SMA type 2 patients were eligible for the effectiveness analysis (initial HFMSE 8.5 ± 12.0). The average improvements in motor performance (measured by HFMSE) after 4,5 and 6 doses of Nusinersen were +6.0 (14.5 ± 17.7), +10.5 (19.0 ± 24.0), and +11.0 points (19.5 ±24.7).  SMA Type 3p+pa Patients:  11 were included in the motor performance improvement analysis. Motor function (HFMSE) improved in 81.8% (9/11) of patients, and the improvement was ≥4 points in 66.7% (6/9) at 6th injection.  The change from baseline became significant after 4 loading doses of Nusinersen and it remained significant during the study period.  SMA Type 3a Patients:  12/17 met the criteria to be included in the motor effectiveness analysis. However, statistically significant improvement in motor performance (measured by RHS) was not achieved with Nusinersen treatment. There was no significant improvement in terms of the 6-Minute Walk Test [N = 4 patients; before treatment: 397.5 (304–413) m vs. after 8 doses already received: 411.0 (380–519) m; p = 0.250] |
| Berti 2022 | n/a |
| Bjelica 2023 | Correlation between motor function and respiratory outcomes reported only |
| Brakemeier 2021 | Significant improvement in the HFMSE score from T0 to T1 (z = -2.236, p = 0.025, n = 22)  No significant difference in the HFMSE score from T0 to T2 (z = -1.248, p = 0.212, n = 18)  No significant pre-post differences were found for the RULM score between T0 and T1 (z = -1.932, p = 0.053, n = 22) or between T0 and T2 (z = -0.256, p = 0.798, n = 18) |
| Brakemeier 2024 | Significant improvement in the HFMSE score from T0 to T1 (z = -2.236, p = 0.025, n = 22)  No significant difference in the HFMSE score from T0 to T2 (z = -1.248, p = 0.212, n = 18)  No significant pre-post differences were found for the RULM score between T0 and T1 (z = -1.932, p = 0.053, n = 22) or between T0 and T2 (z = -0.256, p = 0.798, n = 18) |
| Cavaloiu 2024 | RULM Scores: Median score of 28 (interquartile range 19-33).  HFSME Scores: Median score of 13 (interquartile range 6-28).  Comparative Analysis of RULM and HFSME Scales  RULM Scores:  Higher for SMA Type 3 (median 38) than Type 2 (median 22).  Higher for patients who dropped out (median 34) than those who did not (median 27.5).  Higher with more SMN2 copies (26 for two copies, 30 for three copies, 31 for four copies).  HFSME Scores:  Higher for SMA Type 3 (median 30) than Type 2 (median 3).  Higher for patients who dropped out (median 38) than those who did not (median 13).  Higher with more SMN2 copies (8 for two copies, 16.5 for three copies, 23 for four copies).  Longitudinal Analysis of Motor Function for RULM and HFSME  RULM Scores:  RULM score increases by 0.05 points per month.  SMA Type 3 patients have 13.10 points higher RULM scores.  Age at onset increases RULM score by 0.05 points per month.  HFSME Scores:  HFSME score increases by 0.08 points per month.  SMA Type 3 patients have 16.43 points higher HFSME scores.  Age at onset increases HFSME score by 0.18 points per month |
| Cavaloiu 2024b | n/a |
| Chacko 2022 | All SMA type 1 and 75% of SMA types 2 and 3 showed an improvement in peripheral muscle function greater than predefined response threshold  All SMA type 1 and 75% of SMA types 2 and 3 showed an improvement in peripheral muscle function greater than predefined response threshold  For Type 1: The number of peripheral muscle function responders was 5 for CHOP INTEND and the median after 12 months from baseline has increased by 16.5 (5/6).  Type 2: The number of peripheral muscle function responders was 1 for CHOP INTEND and the median after 12 months from baseline has increased by 9 (1/12).  Type 1: The number of peripheral muscle function responders was 1 for RULM and the median after 12 months from baseline has increased by 1 (1/6).  Type 2: The number of peripheral muscle function responders was 7 for RULM and the median after 12 months from baseline has increased by 1 (9/12).  Type 2: The number of peripheral muscle function responders was 72 for HFMSE and the median after 12 months from baseline has increased by 2 (3/12).  Type 3: The number of peripheral muscle function responders was 6 for HFMSE and the median after 12 months from baseline has increased by 4 (8/8) |
| Chen 2022 | Children’s Hospital of Philadelphia Infant Test of Neuromuscular Disorders: Mean increase of 4.6 points (p = 0.173).  Hammersmith Functional Motor Scale Expanded (HFMSE): Mean increase of 4.7 points (p = 0.021). 76.9% showed improvements at 14 months.  Revised Upper Limb Module (RULM): Mean increase of 2.7 points (p = 0.013). 90.9% improved by month 14.  WHO Motor Milestones:  Baseline: 7 patients with SMA type 3 achieved all milestones.  SMA Types 1 and 2:  Sit without support: 51.1% (23/45).  Hands-and-knees crawling: 4.4% (2/45).  Stand with assistance: 6.7% (3/45).  Walk with assistance: 6.7% (3/45).  Stand alone: 2.2% (1/45).  Improvement at M14:  Sit without support: 100% (11/11).  Hands-and-knees crawling: 36.4% (4/11).  Stand with assistance: 45.5% (5/11).  Walk with assistance: 36.4% (4/11).  Stand alone: 18.2% (2/11).  Achieved all milestones: 1 patient.  CHOP-INTEND:  Baseline: 14 non-sitters (3 with SMA type 1, 11 with SMA type 2).  Improvement:  M2: 36.4% (4/11). M6: 28.6% (2/7). M10: 66.7% (4/6). M14: 83.3% (5/6).  Clinically Meaningful Improvement (≥4 points):  M2: 27.3%. M6: 28.6%. M10: 33.3%. M14: 66.7%.  Scores:  Baseline: 30.9 points. M14: 33.7 points.  Mean change: 4.6 points by 14 months.  Correlations: No significant correlation with disease duration or age at treatment initiation.  HFMSE:  Baseline: 39 patients (32 with SMA type 2, 7 with SMA type 3).  Improvement:  M2: 59.1% (13/22). M6: 50.0% (8/16). M10: 69.2% (9/13). M14: 76.9% (10/13).  Clinically Significant Improvement (≥3 points):  M2: 27.3%. M6: 43.8%. M10: 53.8%. M14: 61.5%.  Scores:  Baseline: 23.4 points. M14: 28.2 points.  Mean change: 4.7 points by 14 months.  Correlations: Negative correlation with disease duration (r = -0.567, p = 0.043) and age at treatment initiation (r = -0.771, p = 0.002).  RULM:  Baseline: 36 patients (29 with SMA type 2, 7 with SMA type 3).  Improvement:  M2: 50.0% (11/22). M6: 60.0% (9/15). M10: 81.8% (9/11). M14: 90.9% (10/11).  Clinically Meaningful Improvement (≥2 points):  M2: 40.9%. M6: 53.3%. M10: 81.8%. M14: 90.9%.  Scores:  Baseline: 19.3 points.  M14: 24.4 points.  Mean change: 2.7 points by 14 months. |
| CHERISH/ISIS 396443-CS4 (NCT02292537) (Mercuri 2018) | Interim Analysis  Primary Outcome  5.9 points (HFMSE least-squares mean difference in change; Nusinersen vs control)  Final Analysis  Primary Outcome  4.9 points (HFMSE least-squares mean difference in change; Nusinersen vs control)  Secondary (Nusinersen vs control)  Children who had an increase from baseline to month 15 in the HFMSE score of at least 3 points: 57% vs 26%; Difference 30.5 percentage points; OR 6  Children who achieved at least one new World Health Organization (WHO) motor milestone: No. 13 vs 2; % 20 vs 6; Difference 14 percentage points  Change from baseline in number of WHO motor milestones achieved — least squares mean: 0.2 vs -0.2; Difference 0.4  Change from baseline in the Revised Upper Limb Module (RULM) score: 4.2 vs 0.5; Difference 3.7  Children who achieved ability to stand alone: 1 (2%) vs 1 (3%); Difference -1 percentage points  Children who achieved ability to walk with assistance: 1 (2%) vs 0 (0%); Difference 2 percentage points |
| Chiriboga 2016 | HFMSE total score at Day 85 (9 mg):  Mean increase from baseline of 3.1 points (17.6%) p=0.016  7/10 participants exhibited a clinically meaningful improvement of ≥3 points in the HFMSE (range 3-7 points; 5/7 Type 2 participants and 2/3 Type 3 participants)  CS10:  Mean change in HFMSE score from CS1 baseline was 5.8 points (32.8% increase; p = 0.008; n = 8).  Six of 8 participants had an increase of ≥3 points.  No participants in the 9-mg cohort declined in HFMSE score  Range of improvement was 1–14 points  (From Chiriboga 2016)  CS10; Day 85  Mean change HFMSE score from CS1 baseline:  3mg (n=6) 1.2 , 6mg (n=4) 2.0, 9mg (n=8) 5.4  Subjects with ³3 point increase in HFMSE score at day 85:  3mg (n=1/6) 17%, 6mg (n=1/4) 25%, 9mg (n=6/8) 75%  (From FDA 2016 p114)  CS10; Day 169  Mean change HFMSE score from CS1 baseline:  3mg (n=6) 0.2, 6mg (n=4) 1.5, 9mg (n=8) 6.1  Subjects with ³3 point increase in HFMSE score:  3mg (n=1/6) 17%, 6mg (n=1/4) 25%, 9mg (n=7/8) 88%  (NB median change and more detail available in FDA, 2016, p 114)  “Significant improvement (p=0.008) relative to baseline in HFMSE scores was maintained  throughout CS10 for subjects who received 9 mg in CS1” (From EMA, 2017, p75)  CS10; Day 169; 9mg cohort only n=8:  Mean change from CS1 baseline -0.6 mV  Mean % change from CS1 baseline -2.2  (FDA 2016, p118)  CS10; Day 169; 9mg cohort only (n=8):  MUNE Mean change from CS1 baseline 11.4  MUNE Mean % change from CS1 baseline 24.7  (FDA 2016, p118) |
| Chiriboga 2024 | Motor Function in Patients Aged 2–60 Years: Stabilization of the MFM32 total score from baseline to month 24, with a mean change of –0.17 (95% CI –1.03 to 0.68, n = 137). Stabilization in upper limb function as shown by the mean RULM total score from baseline to month 24, with a mean change of 0.71 (95% CI 0.17–1.26, n = 133). HFMSE total score stable at month 24 compared with baseline, with a mean change of –0.11 (95% CI –0.98 to 0.76, n = 132). Increase in caregiver-reported SMAIS–ULM total score from baseline to month 24, with a mean change of 1.90 (95% CI 0.70–3.10, n = 110). Respiratory function, as measured by the sniff nasal inspiratory pressure test, showed a mean change of 0.14% (95% CI –2.64 to 2.92, n = 113) from baseline to month 24.  Analyses of Motor Function Measures by Previous Treatment: For patients previously treated with nusinersen, the mean (95% CI) change from baseline to month 24 in the MFM32 total score was –1.25 (–2.55 to 0.04)  6MWT in Ambulant Patients  Total Patients: 15 with Type 3 SMA were ambulant at baseline.  Patients Aged ≥ 6 Years: 14  Assessed with 6MWT: 13  Maintained Ability to Walk: All 13  Evaluated at Baseline and Month 24: 8 (previously treated with nusinersen)  Mean Increase in Distance Walked: 30.88 m (95% CI: -5.54 to 67.29, n = 8)  Patients Not Performing 6MWT: 2  Assessed with HFMSE: Achieved a score of 2 points on Item 20 ('stepping')  Maintained Ability to Walk: Both patients  Motor Function in Patients Aged < 2 Years  Total Patients: 6  Previously Received Nusinersen: 3  Previously Received Onasemnogene Abeparvovec: 3  Sitting for 5 Seconds (BSID-III Gross Motor Scale Item 22):  Baseline: 1 patient (33.3%) with nusinersen, 2 patients (66.6%) with onasemnogene abeparvovec  Month 24: All 6 patients (100%)  Sitting Without Support for ≥ 30 Seconds (BSID-III Gross Motor Scale Item 26):  Baseline: 2 patients (33%)  Month 24: 3 patients (50%)  Motor Milestone Responders at Month 24: All 6 patients (100%) |
| Cho 2023 | Hammersmith Infant Neurological Examination-2 (HINE-2).  Type 2 or 3 SMA patients, the Expanded Hammersmith Functional Motor Scale (HFMSE) is used. Patients unable to sit at the time of the initial Nusinersen treatment, the HINE-2 was used. The HINE-2 and HFMSE scores were collected at baseline, month 2, and every 4 months thereafter for submission for reimbursement approval. Annual follow-up assessments were conducted at 14, 26, and 34 months.  Motor Function Changes in Type 2 SMA:  Included 103 patients with type 2 SMA 96 completed the 1-year, 61 reached the 2-year, and 12 reached the 3-year follow-up  12 patients who reached the 3-year follow-up, changes in mean HFMSE score from baseline scores at 1-, 2-, and 3-year were 4.7, 6.9, and 9.1  Duration from symptom onset to treatment for this group was 14.3 (10.0) years.  Motor function changes in patients with type 3 SMA:  Number of patients enrolled in the study: 13  Number of patients who reached the 2-year follow-up: 6  Mean period between symptom onset and Nusinersen treatment: 23.1 (12.0) years  Number of patients who demonstrated a decrease in motor function during Nusinersen therapy due to infection: 1 (with a baseline HFMSE score of 48) |
| Colot 2024 | n/a |
| Coratti 2021 | In the study cohort, the mean HFMSE scores ranged between 0 and 43 before treatment (T0) (mean: 10.32, SD: ±7.80) and between 0 and 48 (mean:12.05, SD: ±9.26) at T12.  The HFMSE changes ranged between −7 and 15 (mean: 1.90, SD: ±3.85).  On the t-test, there was a difference in the mean HFMSE scores when comparing T0 and T12 (p < 0.001; 95%CI −2.608; −0.892).  Revised Upper Limb Module (RULM):  Seventy-three of the 77 patients in the study cohort had a RULM available at both T0 and T12.  The mean RULM scores ranged between 0 and 29 (mean: 13.50, SD: ±7.38) before treatment (T0) and between 0 and 32 (mean:15.54, SD: ±8.15) at T12.  The RULM changes ranged between −7 and 11 (mean: 1.59, SD: ±3.60).  On the t-test, there was a difference in mean RULM scores when comparing T0 and T12 (p < 0.001; 95%CI −1.721; −0.198).  Predictive Variables:  The assessment of possible predictive variables (age, SMN2 copy number) on functional changes using linear regression analysis showed that age and the baseline score were predictive of both 12-month HFMSE (p < 0.001) and RULM change (p < 0.001) while SMN2 copies was not (p = 0.312 for HFMSE and p = 0.218 for RULM).  HFMSE and RULM Clinical Meaningful Changes:  Eight of the 77 patients in the study cohort (10.39%) had a loss of HFMSE scores greater than −2 points, 43 (55.84%) remained stable (within ±2 points) and 26 (33.77%) had a gain in scores greater than 2 points.  Seven of the 73 patients in the study cohort (9.59%) had a loss of RULM scores greater than −2 points, 39 (53.42%) remained stable (−2; 2 points) and 27 (36.99%) had a gain in scores greater than 2 points.  Study Cohort vs Natural History Studies:  The mean scores of the study cohort were compared to the available untreated patient data for HFMSE and RULM.  On the 12-month mean changes, there were differences between study cohort and natural history on both HFMSE (p < 0.001) and RULM (p < 0.001).  There were no differences between study cohort and the untreated cohorts at baseline (p > 0.05).  When analysing the changes (outside ±2 points), considered to be clinically meaningful, there were differences between study cohort and untreated patient cohort on both HFMSE (p < 0.001) and RULM (p < 0.001) |
| Coratti 2024 | Shift Analysis in Main Study Cohort: 36 out of 104 individuals (34.6%) older than 2.5 years experienced a loss in one or more activities, 19/36 (18.3%) had a loss in two or more activities. 60 out of 104 individuals (57.7%) older than 2.5 years experienced a gain in one or more activities, 38/60 (36.5%) had a gain in two or more activities.  Shift Analysis of Individual HFMSE Items: Positive shift in scores (gain) noted across 30 items, negative shift in scores (loss) occurred in 22 items. No significant differences between groups with 2 and 3 SMN2 copies.  Total HFMSE Score at Baseline and Shift Analysis: Weak correlation between baseline HFMSE level and loss of individual items (r = 0.28, p = 0.010), moderate correlation between baseline HFMSE level and gain of items (r = 0.50, p < 0.001). Excluding items 21 and 22, moderate correlation for item loss (r = 0.56, p < 0.001) and strong correlation for item gain (r = 0.82, p < 0.001).  Comparison of Treated vs Natural History Data: Significant association between natural history group and treated group for loss of at least one activity (χ²(1) = 16.269, p < 0.001) and gain of at least one activity (χ²(1) = 13.794, p < 0.001). Significant associations remained for age subgroups 2 to 5 years and 5 to 13 years.  Item-Level Analysis: Differences in individual distribution between item loss, stable, or gained for specific items in age subgroups 2.5–5 years and 5–13 years |
| Cornell 2024 | n/a |
| CS2/CS12NCT01703988  (see also NCT02052791 an extension study)  Darras 2019 CS12 & CS2 | Hammersmith Functional Motor Scale–Expanded  SMA type II Mean (SE) scores improving by 10.8 (4.3) points from baseline to day 1,150  9/11 (82%) demonstrated clinically meaningful improvements (an increase of ≥3 points from baseline) by day 253  7/9 (78%) demonstrated clinically meaningful improvements by day 1,050  SMA type III Mean (SE) scores improving by 1.8 (0.9) points from baseline by day 1,150  3/16 (19%) demonstrated clinically meaningful improvements by day 253  4/11 (36%) demonstrated clinically meaningful improvements by day 1,050  Ambulant children with SMA type III Mean (SE) HFMSE scores improved by 2.6 (0.8) points from baseline by day 1,150  Clinically meaningful improvements were demonstrated by 2 of 12 (17%) children by day 253 and 4 / 9 (44%) children by day 1,050  From FDA 2016 p139: Expanded Hammersmith Functional Motor Scale Total Score Day 624 n=34 Change from baseline; Mean (SD): 0.47 (3.86) % change from baseline; Mean (SD): -0.2 (12.0)  Day 715 n=23  Change from baseline; Mean (SD): -0.65 (8.49)  % change from baseline; Mean (SD): -0.5 (19.3)  Upper Limb Module  Mean (SE) scores improved by 4.0 (2.4) points from baseline by day 1,150  5/11 (45%) children demonstrated clinically meaningful improvements (≥2-point increase from baseline) by day 253  5 of 9 (56%) children demonstrated clinically meaningful improvements by day 1,050  4/6 (67%) with Type 2 SMA demonstrated ≥2-point increase from baseline at day 1,050 (ICER 2019 pES13)  No ambulant children with SMA type III  At the day 350 visit had reached the maximum score of 18 points and maintained this score through to day 1,150  (n=4; 100% NICE 2020, p63)  From FDA 2016 p139:  Upper Limb Function  Day 624 n=19  Mean change from baseline 0.58 (1.26)  % change from baseline; Mean (SD): 4.8 (9.5)  Day 715 n=14  Mean change from baseline 1.0 (1.11)  % change from baseline; Mean (SD): 8.1 (8.3)  6MWT SMA type II 1/11 gained the ability to walk independently  The child first completed the 6MWT at the day 650 visit (total distance walked was 25.5 meters)  By day 1,150, the child’s 6MWT distance was 180 meters  SMA type III  Mean (SE) distances improving by 92.0 (21.5) meters by day 1,150  2/4 children who had lost ability to walk before the baseline regained the ability to walk independently  6/12 (50%) assessed at the day 253 visit demonstrated clinically meaningful improvements in 6MWT distance (≥30- meter increase from baseline)  8 / 8 (100%) demonstrated clinically meaningful improvements by day 1,050  From FDA 2016 p139: 6 Minute Walk Test Day 533 n=17  Change from baseline; Mean (SD): 35 (71.96)  % change from baseline; Mean (SD): 23.9 (61.7); n=15  Day 624 n=16  Change from baseline; Mean (SD): 26.13 (70.55)  % change from baseline; Mean (SD): 8.9 (28.6)  Day 715 n=8  Change from baseline; Mean (SD): -10.88 (82.55)  % change from baseline; Mean (SD): -0.0 (36.6)  CMAP By day 1,150: SMA type II  Mean (SE) CMAP amplitude increased by 0.4 (0.8) mV  Mean (SE) CMAP area increased by 3.0 (2.4) mV/ms  SMA type III Mean (SE) CMAP amplitude increased by 0.3 (0.5) mV  Mean (SE) CMAP area increased by 0.1 (2.6) mV/ms  MUNE By day 1,150: SMA type II  Mean (SE) MUNE value increased by 2.0 (14.5) from baseline  SMA type III  Mean (SE) MUNE values decreased by 29.6 (15.0) |
| De Wel 2021 | Prospective study  Hand grip strength (p=0.03), hand motor function (p=0.04) as assessed by a sub-score of the Revised Upper Limb Module (RULM) and the Medical Research Council (MRC) sum score (p=0.04) improved significantly at month 14  On average, hand grip strength in the left hand increased by 37% (2.3 kg) at month 6 and 30% (1.9 kg) at month 14  The right hand showed a similar evolution, with an increase of 35% (2.4 kg) at month 6 and 29% (2.0 kg) at month 14  Dominant hand scores were significantly higher, by on average 0.7 kg or 11% (p=0.01)  MRC sum scores improved on average 2.4–2.5 points at months 6–14, respectively  6MWD increased by 5% (average of 16 m) at month 6, which failed to reach significance (p=0.06) for n=7 ambulatory patients. At month 14, the increase from baseline was only 2% (7 m on average).  N=8 patients improved as the HFMSE increased 8% at month 6 from baseline (on average 2.1 points), n=2 deteriorated and n=6 remained stable which maintained at month 14, not significant  N=9 numerically improved and n=2 deteriorated by month 6. By month 14 n=10 improved and n=3 deteriorated compared to baseline  Subdomains of the RULM showed a significant improvement of this sub-score at month 14 (p=0.04). This indicates that not only hand grip strength but also hand function improved with Nusinersen treatment  Forced Vital Capacity (FVC) transiently increased at month 6 (p=0.01), whereas the Peak Expiratory Flow (PEF) did not  Retrospective study  Non-ambulatory patients had a significantly lower MRC sum score at any age (p<0.01)  Patients with an SMN2 copy number of 3, 4 or 5 had significantly higher MRC sum scores than those with two SMN2 copies (p=0.02, p<0.01) and p=0.04, respectively)  Patients with SMA type 3 or 4 had significantly higher MRC sum scores than SMA2 p<0.01 for both |
| Duan 2022 | n/a |
| Duong 2021 | improvement was seen with the CHOP-ATEND (mean slope = 3.59 points/year, 95% CI 0.67–6.51)  In the small subset of participants who performed the TUG test (n = 8), the mean slope on the log scale was −0.10 (95% CI −0.21 to 0.01) with a negative value indicating improvement in the time to complete this task |
| EMBRACE (NCT02462759) (Acsadi 2021) | Of participants originally randomized to sham procedure two of seven (29%) were classified as HINE-2 milestone responders at last available assessment in part 1, and five of six (83%) were classified as responders at last available assessment in part 2 where they received Nusinersen.  HINE-2 results for infantile-onset (Type 1) SMA (p16 ICER doc): @14 months 7(78%) receiving Nusinersen responded compared to 0 in sham control.  ‘Mean weight, body length, head circumference, and chest circumference increased over time for participants in all groups. Weight-for-age percentile, length-for-age percentile, and head-to-chest circumference ratios were similar between treatment groups and appeared to show decreased growth rates at later timepoints. Mean (standard deviation)  changes from baseline to day 183 in body length and weight were 4.5 (4.9) cm and 0.6 (0.6) kg for the group treated with sham procedure in part 1 (n = 7), from baseline to day 659 were 10.8 (4.12) cm and 3.1 (1.4) kg for Nusinersen in part 2 (n = 6), and from baseline to day 898 were 17.2 (5.6) cm and 3.9 (2.9) kg for Nusinersen in parts 1 and 2 (n = 12).’  CGI-C investigator and caregiver assessments at the last observed visit: higher proportion of participants initially treated with Nusinersen improved, compared with those initially treated with sham procedure  Of the eight children in EMBRACE with later-onset SMA, 2/5 (40%) of those who received Spinraza and 2/3 (66%) of those who received sham achieved standing.  Of 5 intervention participants: 4 achieved sitting, 3 crawling, 2 standing and 1 walking. Compared to control participants: 1 achieved sitting, 1 crawling, 2 standing and 0 walking |
| ENDEAR/ISIS 396443-CS3B (NCT02193074(Finkel 2017) | Nusinersen vs Control, no/total no. (%)  Primary Outcomes  Interim Analysis  Motor Milestone Response: 21/51 (41) vs 0/27  Final Analysis  Motor Milestone Response: 37/73 (41) vs 0/37  No death or use of permanent assisted ventilation: 49/80 (61) vs 13/41 (32); Hazard Ratio 0.53  51% of the infants in the nursinersen group had a motor milestone response.  Secondary Outcomes:  Children’s Hospital of Philadelphia Infant Test of Neuromuscular Disorders (CHOP INTEND) Response: 52/73 (71) vs 1/37 (3)  Compound muscle action potential (CMAP) Response: 26/73 (36) vs 2/37 (5) |
| European registries (NICE 2020) | HFMSE Score, Slopes  Treated patients = 0.015 pts/week (95% CI: 0.003–0.027)  Untreated patients = −0.109 pts/week (95% CI: −0.144 to −0.074)  Statistically significant difference (p<0.001)  RULM Score, Slopes  Treated patients = 0.018 pts/week (95% CI: 0.007–0.028)  Untreated patients = −0.009 pts/week (95% CI:−0.039–0.021)  NB A before and after sub-cohort analysis is also available see NICE 2020 p68. |
| Fainmesser 2022 | Significant changes were seen at specific time intervals in MMT, RHS, and ALSFRS-R in the entire cohort and patients with SMA2, but not in patients with SMA3.  The MMT showed significant increase at 6 and 14 months of treatment, but not thereafter.  The median rate of change of MMT was 1.85% a year (n = 35), with a median increase of 1.85 points on MRC scale per year (0.15 points per month).  The median baseline score on the Revised Hammersmith Scale (RHS) was 14/69 points (range 1–58) and there was a significant increase only at 6 months of treatment, but not thereafter.  The median annual rate of change on the RHS was 0 points and the mean change was −0.4 points (n = 35).  The baseline median FEV1 was 87% (range 18–119%), with no significant change at follow up visits.  The ALSFRS-R showed an increase of one point in patients with SMA2 at 6 months of treatment, but not thereafter. |
| Finkel 2021 | In the per-protocol efficacy evaluable population, 12 (63%) of 19 participants met the primary endpoint by having an incremental improvement in HINE-2 developmental motor milestones at the last visit relative to baseline.  Median time to reach a motor milestone in the total efficacy evaluable population was 11·6 months (95% CI 0·92–18·63).  Motor Milestones:  Eight (42%) of 19 participants had full head control at last visit, eight (42%) could sit (three could stable sit and five could pivot), seven (37%) could roll, two (11%) could crawl, four (21%) could stand (three with support and one unaided), and two (11%) could walk (one with support and one unaided).  A developmental motor milestone was fully achieved by one (25%) of four participants in cohort 1 versus eight (53%) of 15 participants in cohort 2.  The proportion who had an improvement in motor milestone achievement was 12 (60%) of 20 participants in the safety population and nine (69%) of 13 participants in the cohort 2 subset with two SMN2 copies.  HINE-2 Motor Milestone Total Score:  In the cohort 2 subset with two SMN2 copies, the HINE-2 motor milestone total score increased steadily over time from a baseline mean of 1·46 (SD 0·52) to 11·86 (6·18) at day 1135, representing a clinically significant change of 10·43 (6·05).  CHOP INTEND Total Score:  In the safety population, mean CHOP INTEND total score increased from 29·7 (SD 10·5) at baseline to 48·3 (12·7) at the last study visit for the 13 surviving participants, a relative improvement of 17·3 points (12·2)  CMAP Amplitudes:  Among the 13 participants in cohort 2 with two SMN2 copies, mean peroneal nerve CMAP amplitudes increased steadily over time, whereas mean ulnar nerve CMAP amplitudes plateaued after day 568 |
| FIREFISH (NCT02913482) (Masson 2022) | After 24 months of treatment, all 12 infants who had previously attained the ability to sit without support for ≥5 seconds at Month 12 maintained this ability. Furthermore, 14 infants (34%) achieved a phase angle reduction of at least 30 degrees from baseline, as measured by RP. At Month 24, 35 infants (60%) met the endpoint of sitting without support for ≥5 seconds and 23 infants (40%) were sitting without support for ≥30 seconds. The first secondary endpoint included in the statistical hierarchy at month 24 was met, with 18 infants (44%) able to sit without support for at least 30 s. However, no infants could stand alone or walk alone after 24 months of treatment.  Improvements were reported for most endpoints included in the statistical hierarchy at month 12. An increase was observed in the proportions of infants achieving sitting without support for at least 5 s and of infants achieving a CHOP-INTEND score of at least 40 points. The proportion of infants who achieved an increase of at least 4 points from baseline on the CHOP-INTEND was maintained at month 24.  Although no infants could walk or stand independently at month 24, more infants achieved a higher motor milestone category in the HINE-2 compared with month 12. For example, more infants achieved “standing with support” and one infant (2%) achieved “cruising”. Furthermore, more infants were recorded as able to achieve the highest motor milestone category. For instance, more infants were able to “pivot (rotate)” as recorded within the sitting milestone, more infants were able to “roll from supine to prone”, and two infants were recorded for the crawling milestone as able to “crawl on their hands and knees” at month 24.  After 12 months of treatment, 23 out of 41 infants (56%) achieved a CHOP-INTEND score of 40 or higher. Additionally, 37 out of 41 infants (90%) showed an increase of at least 4 points from baseline in the CHOP-INTEND score. In the high-dose cohort, 7 infants (33%) were able to sit without support for at least 5 seconds, and 9 infants maintained upright head control. One infant in the high-dose cohort was able to bear weight in standing. CHOP-INTEND scores indicated that 11 infants (52%) had a score of 40 or higher, which is rarely observed in patients with type 1 spinal muscular atrophy.  At month 12, 19 out of 21 infants (90%) had not experienced a clinical event as defined in the Outcomes section. In the applicant’s MMRM analyses of the MFM32 total score at Month 12 by age group in the Sunfish Part 2 study, the greatest improvement from baseline with Risdiplam compared to placebo was observed in the youngest patients aged 2 to <6 years, followed by those aged 6 to 11 years, and those aged 12 to 17. No improvement from baseline in MFM32 was observed in the oldest patients aged 18 to 25.  • Patients’ weight-for-age values were at or below the 50th percentile at baseline, based on World Health Organization (WHO) Child Growth Standards, for the majority of patients (29/41, 70.7%) with a median weight-for-age percentile of 24.0 (range: 0.2–99.0th percentile). Patients’ length/height-for-age values were above the 50th percentile at baseline for the majority of patients (26/41, 63.4%), with a median length/height-for-age percentile of 66.2 (range: 0.3–100th percent) (EMA).  •The median change from baseline in weight-for-age percentiles was 3 (range: -66 to 89), and the median weight-for-age percentile was the 27th (range: 7 to 90). The median change from baseline in length- or height-for-age percentiles was -11 (range: -57 to 69), and the median length- or height-for-age percentile was the 69th (range: 9 to 100) (Baranello) |
| Freigang 2021 | CK and Motor Function: Creatine Kinase (CK) data from 148 patients were available at baseline before starting therapy with Nusinersen. CK was strongly correlated with HFMSE score (q = 0.786, p < 0.001), with RULM score (q = 0.736, p < 0.001), and with ALSFRS-R score (q = 0.742, p < 0.001). CK and Creatinine (Crn) showed moderate correlation (q = 0.424, p < 0.001).  Crn and Motor Function: For the baseline analysis of Crn, datasets of 192 patients were available. Crn moderately correlated with HFMSE score (q = 0.558, p < 0.001), with RULM score (q = 0.511, p < 0.001), and with ALSFRS-R score (q = 0.494, p < 0.001).  CK and Crn Correlation with MUNIX and Hand Grip Strength: In a subgroup of the study cohort, CK and Crn were strongly correlated with APB MUNIX (CK: q = 0.627, p < 0.01; Crn: q = 0.638, p < 0.01), APB MUSIX (CK: q = -0.614, p < 0.01; Crn: q = -0.697, p < 0.001), and hand grip strength (CK: q = 0.694, p < 0.0001; Crn: q = 0.586, p < 0.001).  CK and Crn during Nusinersen Treatment: CK declined in 70.6% of the patients between the first and the last time points of the observation period (ΔCK: -17.6%, p < 0.0001). Crn marginally increased between the first and the last time point in 60% of the patients.  CK and Crn as Predictive Biomarkers: After adjustment for weight, height, and age, mean values of baseline CK and Crn in the group of treatment responders were remarkably higher compared to the group of non-responders. ROC curve analysis revealed a cut-off point for CK ≥ 99.5 U/L and for Crn ≥ 19.3 μmol/L predicting treatment response |
| Freigang 2021b | CHIT1 levels in treatment-naïve patients were not  associated with disease severity (as assessed by HFMSE,  RULM, ALSFRS-R), baseline age, disease onset or disease  duration after correction for patients’ height (see Additional  file 2: Table S2).  CHIT1 dynamics did not differ significantly between  patients with an increase on HFMSE or CHOP score  and patients who lost points on those motor scores  within 14 months.  The correlation of CSF CHT1 with RULM, HFMSE, ALSFR-S are reported and provided in the Additional Table 2 |
| Freigang 2023 | n/a |
| Gaboli 2025 | Improved functional status at 6, 12, and 24 months posttreatment initiation. Type 1b and 1c patients showed progress in independent sitting, and type 2 patients showed progress in walking unassisted. No changes in type 3 patients |
| Gaffar 2022 | Head control n (%) 2 (50) 9 (53) 11 (52)  Bear weight standing n (%) 0 (0) 1 (6) 1 (5)  High CHOP-INTEND score n (%) 1 (25) 10 (59) 11 (52)  Improvement in CHOP-INTEND score n (%) 3 (75) 15 (88) 18 (86)  HINE-2 motor milestone responder n (%) 1 (25) 13 (76) 14 (67)  Improvement in electromyography n (%) 0 (0) 13 (76) 13 (62) |
| García de la Banda 2020 | Motor performance  HINE‐2 was assessed in 14 patients at M0 and M14 and showed a significant improvement with a score increasing by more than 2 points in 7 patients  Total MFM improved also significantly as well as the D3 subscale (55% vs. 61%, p = .021)  Results did not reach statistical significance due to the small size of the cohorts (3 patients in MFM‐20; 13 in MFM 32) |
| Goedeker 2021 | n/a |
| Gonski 2023 | Multiple motor function measures were collected.  There was significant improvement for scores in the WHO (mean difference 1.5 (SD 1.4) (p = 0.02)) and CHOP INTEND (mean difference 17 (SD 17) ( p = 0.02)) in the SMA type 1 group post Nusinersen.  Other motor function measures remained unchanged pre- and post Nusinersen |
| Govoni 2024 | 6MWT Distance Analysis: Total meters covered at 6MWT significantly increased at T6 (306.4 ± 140.3 m vs. 321.5 ± 134.6 m, p < 0.05), T10 (315.7 ± 136.5 m, p < 0.05), and T14 (333.9 ± 127.3 m, p < 0.05). 23 of 27 (85.2%) patients improved or remained stable at T14. No difference in improvement, stability, or worsening at T14 between SMA type 3a and 3b.  Fatigability Analysis: Fatigability detected at baseline in 26/38 (68%) patients, confirmed at subsequent time points without significant change over the treatment period. No correlation between fatigability and SMN2 copy number, sex, age at disease onset, age at baseline, HFMSE values, or 6MWT distance at baseline.  Correlation Analysis: HFMSE score and 6MWT distance moderately correlated at baseline (r = 0.66, p = 0.02), T6 (r = 0.63, p = 0.15), T10 (r = 0.65, p = 0.02), and strongly correlated at T14 (r = 0.79, p = 0.01). No correlation between RULM score and 6MWT distance |
| Gunther 2024 | HFMSE  N=2 patients had a full score on HFMSE compared with baseline. mean HFMSE scores were significantly higher at 14 months (mean difference 1.72 [95% CI 1.19–2.25]), 26 months (mean difference 1.20 [95% CI 0.48–1.91]), and 38 months (mean difference 1.52 [95% CI 0.74–2.30]) after initiation of treatment with Nusinersen  N=68 had clinically meaningful improvements, (29%; mean difference 6.66 [95% CI 5.70–7.62]) patients at 14 months, n=49 (29%; mean difference 6.86 [95% CI 5.75–7.96]) patients at 26 months, and n=36 (30%; mean difference 6.64 [95% CI 5.43–7.85]) patients at 38 months  N=28 with clinically meaningful improvement at 14 months maintained the improvement for ≥38 months of treatment  RULM  N=74 s had a full score (37 points) and n=17 patients had 0 points on the RULM at baseline  Compared with baseline, the RULM was also significantly higher at 14 months (mean difference 0.75 [95% CI 0.43–1.07]), 26 months (mean difference 0.65 [95% CI 0.27–1.03]), and 38 months (mean difference 0.72 [95% CI 0.25–1.18]) after initiation of treatment with Nusinersen  N=68 had clinically meaningful improvements (≥2 points) in the RULM score (29%; mean difference 3.6 [95% CI 3.02–4.18])) patients at 14 months, 48 (28%; mean difference 3.65 [95% CI 3.01–4.28]) patients at 26 months, and 32 (26%; mean difference 3.91 [95% CI 2.97–4.85]) patients at 38 months  6MWT  Clinically meaningful improvements (≥30 m) in 6 MWT were seen in n=39 (48%; mean difference 75.83 [95% CI 64.03–87.63]) patients at 14 months, n=26 (42%; mean difference 85.50 [95% CI 70.11–100.89]) patients at 26 months, and n=21 (48%; mean difference 87.76 [95% CI 58.89–116.63]) patients at 38 months  Mean differences in RULM scores vs. baseline were significantly higher in non-ambulatory patients compared to ambulatory patients at all three timepoints (p < 0.05 at 14 months; p = 0.05 at 26 months; p = 0.02 at 38 months) |
| Hagenacker 2020 | HFMSE 6-month analysis N=124  Mean score= 22·47 (22·41)  Mean difference versus baseline (95% CI)= 1·73 (1·05–2·41)  P value= <0·0001  10-month analysis  N=92  Mean score= 25·52 (22·97)  Mean difference versus baseline= 2·58 (1·76–3·39)  P value= <0·0001  14-month analysis  N=57  Mean score= 27·77 (23·47)  Mean difference versus baseline= 3·12 (2·06–4·19)  P value= <0·0001  RULM  6-month analysis  N=120  Mean score= 21·53 (13·28)  Mean difference versus baseline= 0·66 (0·26–1·05)  P value= 0·0007  10-month analysis  N=90  Mean score= 23·27 (12·46)  Mean difference versus baseline= 0·59 (0·15–1·03)  P value= 0·0014  14-month analysis  N=58  Mean score= 23·95 (12·42)  Mean difference versus baseline= 1·09 (0·62–1·55)  P value= <0·0001  6-minute walk test distance, m  6-month analysis  N=47  Mean score= 366·8 (200·8)  Mean difference versus baseline= 22·1 (8·7–35·6)  P value= 0·0022  10-month analysis  N=37  Mean score= 363·2 (224·2)  Mean difference versus baseline= 31·1 (15·2–47·1)  P value= <0·0001  14-month analysis  N=25  Mean score= 403·0 (225·7)  Mean difference versus baseline= 46·0 (25·4–66·6)  P value= <0·0001  Summary of results  Mean HFMSE scores were significantly increased compared with baseline at 6 months (mean difference 1·73 [95% CI 1·05–2·41], p<0·0001)  Clinically meaningful improvements (≥3 points increase) in HFMSE scores were seen in 35 (28%) of 124 patients at 6 months, 33 (35%) of 92 at 10 months, and 23 (40%) of 57 at 14 months  Except for two patients, all patients with an increase of 3 points or more (i.e., clinically meaningful improvement) at 10 months maintained the improvement up to and after 14 months of treatment |
| Hahn 2022 | n/a |
| Huang 2024 | Correlation Between Motor Function and PROMs: Significant improvements in HFMSE (p < 0.001) and RULM scores (p = 0.025). Positive correlations between HRQoL and motor function scales. Strong correlation between neuromuscular disease module and HFMSE scores. Moderate correlation between total score, neuromuscular disease module, family resources module, and RULM scores.  HFMSE scores: Baseline 15.00 (2.50–38.00), follow-up times 16.00 (4.50–44.00), 22.00 (5.50–46.00), 25.00 (5.50–49.00) (p < 0.001).  RULM scores: Baseline 22.50 (16.75–31.00), follow-up times 26.00 (18.00–31.25), 27.00 (20.00–33.50), 31.00 (22.75–36.00) (p = 0.025) |
| Ip 2024 | N=23  CHOP-INTEND  N=6 (75%) achieved higher total CHOP-INTEND scores in their latest reviews compared to the baseline. All these six patients had varying degrees of scoliosis progressions  Median CHOP-INTEND score improved from 25.5 to 28  HINE  All patients achieved higher or the same total HINE scores at the latest assessments compared to the baseline  N=8 SMA1 patients who were non-sitters before treatment n=2 (25%) became independent sitters, and n=3 (38%) became supported sitters  HMSE  The median HFMSE increased from 4 (range 0–21) to 8 (range 0–30)  N=6 patients (86%) achieved higher HFMSE scores at the latest assessment  In group of 9 SMA3 patients e median HFMSE score improved from 41.5 to 47.5  N=7 (88%) achieved improvements in their HFMSE scores at the latest assessment  RULM  The median RULM scores increased from 8 (range 1–22) at baseline to 11 (range 2–26) at the latest assessment  N=6 patient (86%) achieved higher RULM scores at the latest assessment  N=1 SMA type 2 patient (14%) who was an independent sitter before treatment became supported walker at the latest assessment.  Improved in 5/7 patients with reported scores, from a baseline median score of 32 to 35  6MWT  N=5  4/5 patients recorded an improvement in the walking distances in their 6MWT after Nusinersen, improving from a baseline median distance of 87 meters (range 33.5–243 meters) to 112 meters (87–355 meters) at the latest assessment  Found a strong correlation between the increase in CHOP-INTEND score after Nusinersen treatment and the rate of scoliosis progression in SMA1 patients, correlation coefficient of rs = 0.741 (p = 0.041) |
| JEWELFISH (NCT03032172) 2023 (Chiriboga 2023) | n/a |
| Jiang 2024 | Stability or gain of WHO motor milestones observed, mean HINE-2 scores improved in both subgroups. No serious respiratory events, no permanent ventilation support initiated. Pre-dose nusinersen CSF concentrations increased steadily through the loading-dose period, no accumulation in plasma after multiple doses.  Motor Milestones: In the later-onset group, each of the six motor milestones had been achieved by at least one participant. Three participants gained the ability to "sit without support" during the study. Participants who demonstrated motor milestones at baseline also gained additional milestones during the study. In the youngest subgroup (aged ≤2 years), four individual participants gained one, two, two, and five milestones, respectively. Single participants in each of the older two subgroups (aged >2 to ≤6 years, >6 years) gained the motor milestone of "hands and knees crawling." All milestones achieved at baseline in the later-onset group were maintained as of each participant’s last observed visit, except for one participant in the oldest subgroup (aged >6 years at enrollment) who demonstrated "sitting without support" at baseline and all subsequent visits except the last visit (Day 423). All milestones gained throughout the study by the later-onset group were demonstrated at all subsequent visits except for one participant (aged ≤2 years at enrollment) who demonstrated "standing with assistance" at Day 183 through Day 663 (last visit), with the exception of non-demonstration of this milestone at the Day 543 visit.  HINE-2 Scores: Gradual improvement in HINE-2 total motor milestone scores over time in both infantile- and later-onset SMA groups. Baseline mean HINE-2 scores were 8.4 (infantile-onset) and 13.3 (later-onset). Mean change from baseline in HINE-2 scores was 7.3 (infantile-onset) and 3.2 (later-onset) at Day 423. Proportion of HINE-2 responders: 77.8% (infantile-onset) and 93.8% (later-onset) |
| Jiang 2024b | n/a |
| Kariyawasam 2021 | Clinical characteristics  Prior to the start of treatment, 5 children (25 %) were classified as non-sitters, 13 (65%) as sitters and 2 children (10%) walked either independently or with support  N=20 y children (median age 99 months, range 4–193) were followed for a median of 13.8 (4–33.5) months  Longitudinal changes in electrophysiological measures with therapeutic intervention  Therapeutic intervention with Nusinersen was an independent and significant contributor to an increase in compound muscle action potential (CMAP) (p=0.005), motor unit number estimation (MUNE) (p=0.001) and N50 (p=0.04)  Electrophysiological measures and their association with change in motor function  The magnitude of this electrophysiological response was increased in children with shorter disease durations (p<0.05)  Of the 18 children who completed functional motor assessments, the majority remained clinically stable (n=12, 67%) or showed clinically significant improvements (as validated by change in the HFSME/CHOP-INTEND scores) in motor scores (n=6, 33%) |
| Kokaliaris 2024 | Motor Function Outcomes: Risdiplam showed a 45% higher rate of achieving a HINE-2 motor milestone response (HR 1.45) and a 186% higher rate of achieving a ≥4-point improvement on the CHOP-INTEND (HR 2.86) compared to nusinersen.  Assessment Schedule Matching (ASM): Results from MAIC with ASM continued to support the suggestion that children treated with risdiplam had a higher rate of achieving a response on the HINE-2 and CHOP-INTEND compared with children who received nusinersen. The HR for the achievement of a HINE-2 motor milestone response increased to 1.58 (95% CI 1.28–2.06) following MAIC with ASM. The HR for the time to achievement of a ≥4-point improvement in CHOP-INTEND score decreased to 2.43 (95% CI 1.90–3.61), but CIs overlapped, still suggesting superiority of risdiplam over nusinersen |
| Kolbel 2022 | n/a |
| Konersman 2021 | Non-sitters  Scores on the CHOP INTEND increased by about 0.30±0.10 (SE) over time (0–22 months).  Patients within the non-sitter cohort improved by approximately 6 points on the CHOP INTEND for the duration of the study period, p = .01  Improvement for Hammersmith Infant Neurological Examination-2 by 2.6% (p=.008) over the 22-month study period  Sitters  RULM increased by about 0.20±0.07 (SE) across time (0–22 months  Patients within the sitter cohort improved by approximately 4.4 points on the RULM for the duration of the study period, p = .0002  Improvement for Hammersmith Functional Motor Scale-Expanded by 3.3% (p=.00005) post treatment with Nusinersen |
| Kotulska 2022 | Functional Status Assessment:  The functional status was assessed using the CHOP-INTEND scale in 170 patients; it ranged from 0 to 60. Fifty-two patients (30.6%) had a score of 0–10, while 16 patients (9.4%) scored 46 points or more.  The HFMSE was used in 121 patients and the scores ranged from 6 to 52. Sixty-one patients (50.4%) scored 36 points or more, whereas 16 patients (13.2%) had up to 10 points.  Treatment Response:  No patient met the inefficiency criteria. At V6, all patients assessed by the CHOP-INTEND scale had improved or remained stable. The mean change in CHOP-INTEND score was an increase of 8.9 points between V0 and V6 (p < 0.001).  After 1 year of treatment, some improvement was reported in 87% of the patients in the CHOP-INTEND subgroup and in 90% of the patients in the HFMSE subgroup. Improvement for at least 4 points was observed in 129 out of 170 (75.9%) patients assessed in the CHOP-INTEND score and in 88 out of 121 (72.7%) patients assessed in the HFMSE score.  In the CHOP-INTEND score, the improvement of at least 4 points was observed in 82 (48.2%) patients at V4 and in 106 (62.3%) patients’ at V5  Regression Analysis:  The regression analysis revealed that the baseline functional status of patients was a major determinant of response to treatment in both the CHOP-INTEND and HFMSE subgroups. Patients with higher baseline scores responded significantly better than those with low baseline scores.  In the CHOP-INTEND subgroup, the response to treatment was also significantly better among SMA type 2 or 3 patients in comparison to individuals with SMA type 1. Moreover, younger age was associated with better response in patients assessed in the CHOP-INTEND scale. In the HFMSE subgroup, no such correlations were found.  4 Patients who started treatment at the presymptomatic stage of the disease did not develop any SMA symptoms during the 1- year follow up  Table results  Factors influencing the change in CHOP-INTEND score (n = 170) between baseline and Visit 4, 5, and 6.  Variable Level Coefficient 95% CI p-value  Visit 4 vs 0 3.7 2.6–4.8 <0.001 5 vs 0 6.8 5.5–8.0 <0.001 6 vs 0 8.9 7.3–10.6 <0.001  Age - 0.5 - 0.7– - 0.3 <0.001  Gender M vs F - 0.1 - 1.8 - 1.6 0.876  SMN2 copies 3 vs (1–2) 0.1 - 2.1 - 2.3 0.926 4+ vs (1–2) 2.0 - 2.5 - 6.5 0.384  SMA type: SMA2 vs SMA1 3.9 1.3–6.4 0.004 and SMA3 vs SMA1: 4.8 0.7–8.8 0.022  V0 score 11-20 vs 0-10: 12.5 10.0–15.1 <0.001 21-35 vs 0-10 22.6 20.0–25.2 <0.001 36-45 vs 0-10 32.5 29.0–36.0 <0.001 45+ vs 0-10 42.7 38.9–46.5 <0.001  Factors influencing the change in HFMSE score (n = 170) between baseline and Visit 4, 5, and 6.  Variable Level Coefficient 95% CI p-value  Visit 4 vs 0 3.1 2.4–3.8 <0.001 5 vs 0 4.8 4.0–5.6 <0.001 6 vs 0 6.1 5.0–7.2 <0.001  Age 0.1 - 0.1 - 0.3 0.3  Gender M vs F - 0.3 - 2.5 - 1.9 0.8  SMN2 copies 3 vs (1–2) 2.4 - 1.9 - 6.7 0.3 4+ vs (1–2) 1.8 - 2.8 - 6.4 0.4  SMA type SMA2 vs SMA1 - 0.5 - 7.0 - 6.1 0.9 SMA3 vs SMA1 1.3 - 5.2 - 7.9 0.7  V0 score 11-20 vs 0-10 10.7 6.5–14.9 <0.001 21-35 vs 0-10 22.6 18.5–26.7 <0.001 36+ vs 0-10 41.0 36.6–45.3 <0.001 |
| Krosmark 2023 | Ten patients increased their HFMSE score from baseline - follow up, three remained stable and six decreased their score  The mean change from baseline was 1.0 ranging from −7 to 17, (p = 0.48)  8/10 Nusinersen-treated patients increased their motor function (i.e., HFMSE), than in the non-treated group (2 out of 9)  The mean difference between the groups was 4.0 (p = 0.067). However, Hedge’s g effect size was high (0.82)  Significant correlation at baseline for BMD measured as THL Z-score and motor function (HFMSE), rs = 0.67 (p = 0.0035), and for TBLH Z-score and motor function (HFMSE), rs = 0.57 (p = 0.026)  Significant correlation was also found for the change in BMD measured as THL Z-score and change in motor function (HFMSE), rs = 0.51 (p = 0.044) |
| Krupa 2023 | n/a |
| Kwon 2022 | n/a |
| Landfeldt 2023 | n/a |
| Lavie 2021 | n/a |
| Lee 2022 | n/a |
| Lee 2023 | n/a |
| Lemska 2024 | CHOP-INTEND score: Significant improvement over 22 months. Average increase of 4.2 points at 6 months, 17.8 points at 22 months. 100% of patients showed either stabilization or improvement.  CHOP-INTEND Scores by SMA Type:  SMA Type 1: Improvement of 3.9 points at 6 months, 7 points at 10 months, 11.9 points at 14 months, 16.3 points at 18 months, 19.1 points at 22 months.  SMA Types 2 and 3: Combined analysis due to small sample size. Improvement of 5 points at 6 months, 11.4 points at 10 months, 12.4 points at 14 months, 13.1 points at 18 months, 14.7 points at 22 months. |
| Li 2024 | All n=42 patients received 6 months of treatment  Of which 20 cases (47.6%) showed an improvement in Hammersmith score ≥3 but <10 compared to baseline  6 cases (14.3%) showed an improvement of over 10 points  21 patients who received 14 months of treatment  Of which 16 cases (80%) showed an improvement in Hammersmith score ≥3 compared to baseline  Changes in Peripheral Motor Nerve Amplitude in SMA Patients After Nusinersen Treatment  “After Nusinersen treatment, there were no statistically significant differences in the amplitudes of the median nerve, ulnar nerve, axillary nerve, tibial nerve, peroneal nerve, and femoral nerve among 42 cases of 5q SMA patients, regardless of whether it was compared to baseline or after 6 months or 14 months of treatment |
| Li 2024a | HFMSE score: Mean baseline score 20.99 ± 16.95, V4 score 22.86 ± 16.29. Significant improvement over time, with 100% of children showing ≥3 points improvement by V6.  CHOP INTEND score: Mean baseline score 35.18 ± 18.34. Significant improvement over time, with 83.6% of children showing ≥3 points improvement by V6.  RULM score: Mean baseline score 22.72 ± 8.57. Significant improvement over time, with 66.6% of children showing ≥3 points improvement by V6.  6MWT: Baseline distance 172.38 ± 136.80 m, increased to 216.00 ± 52.08 m by V6.  Factors influencing HFMSE scores: Age at treatment initiation significantly influenced improvement, with better outcomes for children starting treatment before age 5. |
| Li 2024b | n/a |
| Lillien 2024 | Functional Assessments (15 Months Post-Treatment)  HFMSE:  Prioritized for patients older than 2 years (51/99 patients)  NR: 24 patients (13 adults, 11 children)  RCS: 27 patients (22 children)  CHOP-INTEND:  Equal distribution across groups (RCS: 5, RNCS: 3, NR: 3)  Score Differences:  Higher in children than adults in RCS group |
| Lusakowska 2023 | HFMSE n=73: At least 1-point improvement in 72% patients (52/72) at T6 versus T0, and in 86% patients (24/28) at T30 versus T0.  Clinically meaningful improvement (≥ 3 points) in the HFMSE in 36% of the patients (26/72) after six months of treatment (T6)  Responders= 71% (20/28) at T30 versus T0  10-point improvement in 15% (11/73) Of those 8 were still able to walk, 6 had 4 copies of SMN2, and 5 had 3 copies of SMN2.  4/73 had a score of ≥ 60 points at T0  Worsening was observed in 8% of patients at T6 and 4% of patients at T30  A mean HFMSE score at T0 was 34.0 points and 40.9 points at T30 (mean value of differences 2.5 points and doubled to 5.1 points at T30) p<0.001 statistically significant differences between ambulant and not-ambulant patients was not found in any point of treatment (p > 0.2)  CHOP INTEND n=47: An improvement by at least 1 point was noted in 77% (34 of 44) of patients at T6 and in 94% (16 of 17) of patients at T26 vs. baseline  Clinically meaningful improvements (≥ 4 points) in the CHOP-INTEND score was observed in 20.5% (9 of 44) at T6 and in 65% (11 of 17) at T26  At T30, only 5 patients were assessed and improvement versus baseline was noted in 4 (all SMA1)  The mean CHOP-INTEND score increased significantly between T0 (24.2 points) and subsequent time points up to T26 (28.3 points)  The mean value of differences between T0 and T6 was 2.23 points and increased to 5.59 points at T26  The mean differences between T0 and each time point of treatment up to T30 reached the statistical significances (p < 0.001)  RULM: n=51. Of the 51 patients, 11 (21.5%) had the maximum score (37 points) at T0 and maintained.  At T6 and T10, 20/51 (30%) Improvement seen at both time points  The mean RULM score significantly increased between T0 and subsequent time points up to T30, except between T0 and T10  The mean RULM score changes between baseline and each time point of treatment assessed separately for ambulant (26) and non-ambulant (25) patients significant improvement is observed only after T22  The data showed that non-ambulant patients gained better improvement.  6MWT n=27. Clinically meaningful improvement (change in 6MWT ≥ 30 m) was observed in 33% (5 of 15) at T6, and these values gradually increased to 50% (6 of 12) at T30  Of patients with any worsening At T6 was 40% (6 of 15) and at T30 was 33% (4 of 12)  Statistically significant improvement in 6MWT results for T10 (22.1, p = 0.007), T14 (16.6, p = 0.041), and T18 (18.1, p = 0.028) versus T0. At other time points, no significant differences were found |
| Maggi 2020 | HFMSE  HFMSE score in SMA3 increased from baseline by a median of +1 point (range −5 to 8) at T6 (p<0.0001)  HFMSE changes were independently significant in ‘sitter’ and ‘walker’ SMA3 subgroups: at T14  3-point median increase was observed in sitters (p=0.0014)  2-point median increase in walkers (p=0.00016)  In SMA2, no significant HFMSE change was found between T0-T6  RULM  In SMA3, median RULM remained unchanged between T0–T6 and T6–T10  Increased by a median +0.5 points (−6 to 6) between T0 and T14 (nominally significant, p=0.012)  Sitter patients with SMA3, nominally significant changes were observed at T10 (+1, range −6 to 5, p=0.021) and T14 (+2, range −6 to 5, p=0.018)  In SMA2, median RULM did not change between baseline and T6, T10 and T14, however positive trend was observed (median +2 points at T14, range 0–3  6MWT and timed tests  Distance increase was strongly significant at T6 (median +11m; p=0.0005), T10 (+25m; p=0.00019) and nominally significant at T14 (+20m; p=0.016)  Rise from chair velocity increase was nominally significantly at T6 (+0.02s –1; p=0.026) and T10 (0.04s –1; p=0.016) and strongly significant at T14 (0.06s –1; p=0.0067)  10 meter run/walk speed increased only at T6, with nominal significance (+0.07 m/s; p=0.02).  The rate of patients showing clinically meaningful improvements (as defined during clinical trials) increased from 53% to 69% from T6 to T14 |
| Mendonca 2021 | Mean change in CHOP-INTEND score was 4.9 points after 6 months,5.9 points after 12 months, 6.6 points after 18 months and 14 points after 24 months of treatment. For statistical analysis, only patients who had assessments at 6 and 12 months after treatment were included  Univariate analysis showed no association between CHOP-INTEND response and the variables: CHOP-INTEND score at baseline, age of symptoms onset, SMN2 copies, and sex.  Change in CHOP-INTEND scores from baseline to6 and 12 months after treatment was influence by disease duration (p = 0.006) and baseline ventilatory support (p = 0.018) as well as an interaction between these variables (Fig. 1; Supplemental Table). Regard-less of the baseline ventilatory support, the patients treated before 12 months of disease duration presented a satisfactory response, while those treated after 24 months improved less than 4 points on the CHOP-INTEND. Patients treated between 12 and 23months of disease duration had an outcome that was largely impacted by the baseline ventilatory support.  Among the 21 patients, six (28.6%) acquired some motor milestone or gained at least three points on theHINE-2 (Table 1); two children were able to sit independently, one sat with support, and three achieved head control. Univariate analysis showed no association between HINE-2 response and the variables: age of symptoms onset, SMN2 copies and CHOP-INTEND score at baseline. Multivariable analysis for the predictors of HINE-2 response in three different models showed that sex (female), disease duration and baseline ventilatory support were predictors of response |
| Mendonca 2021b | • Mean change is +1.47 points (SD = 0.4) and +1.60 points (SD = 0.6) after 12 and 24 months of treatment, respectively  • Control group presented a mean loss of −1.71 points (SD = 0.02) and −3.93 (SD = 0.55) after 12 and 24 months of follow-up, respectively  • Both treated patients with SMA type 2 and type 3 benefitted from Nusinersen therapy (p < 0.001)  CHOP-INTEND measurement  • Mean change was +2.37 points (range: −5 to 10) at 12months of follow-up among the patients overall.  • For two years (n = 7), the mean change was +1.0 point (range: −5 to 8) and +3.42 (range: 0 to 14) after 12 and 24 months of follow-up, respectively.  • Younger patients between 1.5 and 4 years scored higher but due to low number of patients statistical analysis was unviable |
| Meyer 2021 | n/a |
| Mirea 2021 | The CHOP-INTEND score of one patient (7NZ) increased from 8 to 29 points at 6 months after T1 (five doses of Nusinersen) and reached 36 points 12 months after T1 (six doses of Nusinersen). The score was maintained at 36 points 2 months later, when this patient received GT infusion. There were no changes in ventilation hours and number of cough assist sessions before GT dosing and 1 week later.  Only in two patients did the dose of prednisone need to be increased and require prolonged treatment. Probably due to a longer (5 months) corticosteroids treatment period, one of the patients presented with demineralization fractures, for which treatment was recommended with supplementary vitamins (C, D) and minerals (calcium, magnesium, phosphorus).  For the only patient who received first onasemnogene abeparvovec-xioi (Patient 6ZN), CHOP increased four points after 6 months from T1 and nine points 6 months after T2 (before the fifth dose of Nusinersen). Ventilation time was reduced by 2 h/day for 6 months after T1, while in the same period after T2, no change in NIV hours was needed. For cough assist, a decrease was registered—1 time for T1, but no change after T2.  In the other five patients who received Nusinersen first (Patient 1NZ-5NZ), higher improvements in CHOP-INTEND scores were observed 6 months after T1 compared to the same period after T2. Ventilation hours were not modified 6 months after T1, but before T2, the median for the ventilation hours dropped. The median for cough assist number of sessions decreased after 6 months from T1 and 6 months after T2.  In the control group, for the 6-month period evaluated, slight improvements in ventilation were observed and insignificant changes in the cough assist session.  The statistical analysis for CHOP-INTEND score evolution 6 months after therapy initiation for both groups is presented in Table 3. The CHOP-INTEND score evolution showed a rapid increase in the first 6 months from T1; then, in the following 6–12 months, the trajectory of the curve grew at a slower pace. The same score progression is observed 6 months later after T2.  Table 3:  CHOP Evolution in control group after 6 months  Mean: 13.33, Median: 13.00, Min: 8 , Max; 20  CHOP Evolution in study group at 6 months after T1  Mean: 15.17, Median: 14.00, Min: 4.00, Max: 29.00  CHOP Evolution in study group at 6 months after T2  Mean: 9.33, Median: 9.00, Min: 4.00, Max: 16.00 |
| Mirea 2022 | The evolution of motor function for the two groups (PT-N group and N group) was assessed using CHOP for type I patients and HFMSE for type II and type III patients.  Calculated Yield for CHOP/HFMSE Score Evolution:  At T1, for the PT-N group:  CHOP yield for SMA type I patients was between 4.17% and 21.05%, mean value 13.62%.  HFMSE for SMA type II was between 0 and 10.53%, mean value 5.86%.  HFMSE for SMA type III patients was between 10% and 33.33%, mean value 19.83%.  For the N group:  CHOP yield for SMA type I patients was 3.45%.  HFMSE for SMA type II was between 0 and 4.55%, mean value 2.81%.  HFMSE for SMA type III patients was 0.00%.  At T2, for the PT-N group:  CHOP yield for SMA type I patients was between 15.38% and 55.56%, mean value 33.22%.  HFMSE for SMA type II was between 5.26 and 12.96%, mean value 10.16%.  HFMSE for SMA type III patients was between 12.5% and 44.44%, mean value 34.28%.  For the N group:  CHOP yield for SMA type I patients was 6.90%.  HFMSE for SMA type II was between 0 and 7.41%, mean value 4.35%.  HFMSE for SMA type III patients was between 0 and 14.29%, mean value 7.15%.  Higer yields were present for SMA type 1 patients, evaluated using CHOP than for SMA type II patients for whom HFMSE was performed.  Comparison of Means of Assessments:  At 6 months after Nusinersen initiation, the calculated yield for the PT-N group was 11.23 ± 7.44% and at 12 months 23.89 ± 15.93%.  The analysis has shown a strong (0.802) and significant (p < 0.0001) correlation between yields.  There is a statistically significant (p < 0.001) improvement in motor functionality of PT-N group patients at 12 months after Nusinersen initiation, from 11.23 ± 7.44% at 6 months, to 23.89 ± 15.93%, respectively an improvement of 12.66 ± 10.91%.  At 6 months after Nusinersen initiation, the calculated yield for N group was 2.19 ± 1.88% and at 12 months 5.37 ± 4.39%.  The analysis has shown no significant correlation between yields.  There was a statistically significant improvement in the health condition of N group patients at 12 months after Nusinersen initiation, from 2.19 ± 1.88% at 6 months after Nusinersen initiation, to 5.37 ± 4.39% (p < 0.05), respectively an improvement of 3.18 ± 4.73%.  Comparison of Progress in Motor Milestones:  Compared to the N group, patients in the PT-N group had statistically significant (p < 0.001) higher progress (almost 4 times) in motor milestones, respectively 12.66%, compared to 3.18%  Table 2. Calculated yield for CHOP/HFMSE scores evolution after T1.  yield 1 (T1–T0)  PT-N group: N Mean Minimum Maximum /N group: N Mean Minimum Maximum  SMA type I CHOP: 18 13.62% 4.17% 21.05% /2 3.45% 3.45% 3.45%  SMA type II HFMSE: 16 5.86% 0.00% 10.53% /10 2.81% 0.00% 4.55%  SMA type III HFMSE: 5 19.83% 10.00% 33.33% /4 0.00% 0.00% 0.00%  Table 2. Calculated yield for CHOP/HFMSE scores evolution after T1.  yield 1 (T1–T0)  PT-N group: N Mean Minimum Maximum /N group: N Mean Minimum Maximum  SMA type I CHOP: 18 13.62% 4.17% 21.05% /2 3.45% 3.45% 3.45%  SMA type II HFMSE: 16 5.86% 0.00% 10.53% /10 2.81% 0.00% 4.55%  SMA type III HFMSE: 5 19.83% 10.00% 33.33% /4 0.00% 0.00% 0.00%  Calculated yield for CHOP/HFMSE score evolution after T2.  yield 2 (T2–T0)  PT-N group: N Mean Minimum Maximum /N group: N Mean Minimum Maximum  SMA type I CHOP 18 33.22% 15.38% 55.56% /2 6.90% 6.90% 6.90%  SMA type II HFMSE 16 10.16% 5.26% 12.96% /10 4.35% 0.00% 7.41%  SMA type III HFMSE 5 34.28% 12.50% 44.44% /4 7.15% 0.00% 14.29%  Paired Samples Statistics for yield 1 (after T1) and yield 2 (after T2) for PT-N group.  Paired Samples Statistics  Pair 1: Mean N Std. Deviation Std. Error Mean  yield 1 11.23% 39 7.44% 1.19%  yield 2 23.89% 39 15.93% 2.55%  Paired Samples Correlations for yield 1 and yield 2 for PT-N group.  Paired Samples Correlations: N Correlation Sig.  Pair 1 yield 1 & yield 2: 39 0.802 0.000  Paired Samples Test for yield 1 and yield 2 for PT-N group.  Paired Samples Test  Paired differences  Pair 1 yield 1 & yield 2 MEAN–12.66% Std. deviation10.91% Std. Error Mean 1.75% 95% CI(–16.20% –9.12%)  t –7.25 df 38 Sig. (2-tailed)0.000  Paired Samples Statistics for yield 1 (after T1) and yield 2 (after T2) for N group.  Paired Samples Statistics  Mean N Std. Deviation Std. Error Mean  Pair 1 yield 1 2.19% 16 1.88% 0.47%  Pair 1 yield 2 5.37% 16 4.39% 1.10%  Paired Samples Correlations for yield 1 and yield 2 for N group.  Paired Samples Correlations for Pair 1 yield 1 & yield 2  N Correlation Sig.  16 0.024 0.929  Paired Samples Test  Paired Differences  Pair 1 yield 1 & yield 2: mean –3.18% std. deviation4.73% std. error mean 1.18% CI95% (–5.70% –0.66%)  t df Sig. (2-tailed)  –2.70 15 0.017 |
| Modrzejewska 2021 | N=26  N=16 patients (61.54%) improved, measured by the clinically meaningful threshold of 4 or more points in CHOP-INTEND  Mean score in CHOP-INTEND at last follow-up was 26.50 ± 18.04, which is a mean improvement of 7.38 points from baseline (p < 0.001; 95% CI, 4.69–10.07)  Patients with three or more SMN2 copies had higher CHOP INTEND scores (15.25, 95% CI: 4.02–26.47, p = 0.013) and tended to improve in functional tests more than the rest of the patients, but this effect was non-significant (2.78, 95% CI: –2.63–8.20, p = 0.324)  The effect of time remained significant (p = 0.015), patients with CHOP INTEND baseline score above median had higher scores (p < 0.001) and improved significantly more than the rest of the patients (5.53, 95% CI: 0.62–10.44, p = 0.037)  Mean improvement in CHOP-INTEND from baseline to the last follow-up was 7.38 points (p < 0.001)  Shorter disease duration and higher CHOP-INTEND baseline score were associated with a better response (p = 0.015)  Patients with a CHOP-INTEND score above the median had higher scores overall than the rest (p < 0.0013), and they improved significantly more than the rest (p = 0.037) |
| Moshe-Lilie 2020 | N=10 treated with Nusinersen for a period ranging from 6 to 24 months (median 12 months)  % MRC change was 2.5% at 12 months and 3.9% at 24 months  Most patients in the untreated group had stable % MRC; 3 showed decline in % MRC strength ranging from 2.5% to 3.8% at 12–24 months  Hammersmith Functional Motor Scale score was recorded in n=3 patients treated for 12 months  N=2 patients remained stable, n=1 patient in her early 20s demonstrated improvement of 12 points (scored higher in rolling, lying to sitting, crawling, ability to prop on extended arms)  N=1 endorsed muscle movement in her left arm  N=3 (2 younger than 30) reported an increase in hand strength leading to improved manoeuvring at 12 months  No significant change in grip strength on handheld manometer in patients followed up to 24 months  Summary of results  In the treated group, on average, % Medical Research Council change was 2.5% at 12 months and 3.9% at 24 months |
| NURTURE (NCT02386553 (Crawford 2023) | • Secondary Outcomes: Motor Milestones:  o All 25 children achieved and maintained the WHO motor milestone of sitting without support.  o One child (two SMN2 copies) achieved crawling, walking with assistance, standing alone, and walking alone since 2019 analysis.  o All children who achieved a WHO motor milestone retained this achievement at the last study visit.  o Overall, all children with three SMN2 copies achieved all WHO motor milestones within normal developmental timeframes, apart from walking with assistance in one child.  o In children with two SMN2 copies, all achieved sitting without support and standing with assistance, 14 achieved hands and knees crawling and walking with assistance, and 13 achieved standing alone and walking alone, with some attaining milestones within normal developmental timeframes.  o Seven children who previously developed protocol-defined SMA symptoms by 24 mo11 (all with two SMN2 copies) continued to grow and gain weight. The maximum milestones achieved were the ability to walk alone (n = 5), standing with assistance (n = 1), and walking with assistance (n = 1).  • Changes in Motor Function:  o Mean CHOP INTEND total scores rose steadily from baseline until approximately Day 183 and then remained stable over time.  o At the last visit, mean CHOP INTEND total score was 62.1 in those with two SMN2 copies and 63.4 in those with three SMN2 copies.  o At the time of this interim analysis, 10/15 (67%) of participants with 2 SMN2 copies and 10/10 (100%) of those with 3 SMN2 copies had achieved a maximum score of 64.  o At the last observed visit up to and including Day 778, all NURTURE participants had the ability to suck and swallow as measured by the HINE-1 neurological assessment.  o Twenty two participants (12/15 with two SMN2 copies, 10/10 with three SMN2 copies) achieved the maximum score of 3 (good sucking and swallowing) on the HINE-1.  o 3 (with two SMN2 copies) achieved a score of 1 (poor sucking and/or swallowing) and had gastrostomy tubes placed.  o All children continued to grow and gain weight. Mean weight-for-age stabilized over time, and half (12/25 [48%]) were between the 25th and 75th percentiles of weight-for-age at last visit while 7/25 (28%) were in the 75th percentile.  o Mean ulnar nerve CMAP amplitudes at baseline were 2.69 mV and 3.11 mV in participants with two and three SMN2 copies, respectively, and remained stable over time. |
| Okamoto 2020 | 3.1. SMA Patient Data Collected via Questionnaires  • 131 institutions were contacted (87 paediatric departments, 44 neurology departments).  • 117 institutions responded (82 paediatric departments, 35 neurology departments).  • 21 patients were diagnosed with and treated for SMA.  • 16 of these patients were identified as 5q-SMA with homozygous deletion of SMN1 as found by genetic testing.  • The remaining 5 patients were clinically diagnosed with SMA but were not included as 5q-SMA in this study.  3.2. Clinical Course of 14 5q-SMA Patients  • Detailed clinical information was collected on 14 of the 21 patients in the second questionnaire.  • All 14 patients had SMN1 homozygous gene deletion and were diagnosed with 5q-SMA.  • All but one (Case 10) had received Nusinersen therapy.  Cases 4 and 5  • Monochorionic diamniotic twins.  • Born at 37 weeks gestation with body weights of 1895 g and 1835 g, respectively.  • Cared for in the neonatal intensive care unit until 31 days.  • At 3 months, they suffered from respiratory syncytial virus infection, warranting tracheostomy and tracheostomy positive pressure ventilation.  • Genetic testing showed no copies of the SMN1 gene and two copies of SMN2 in both; thus, diagnosed as 5q-SMA and classified as type 1.  • Nusinersen therapy was started at 3 months 29 days.  • One year after its start, CHOP-INTEND scores improved from 16 to 44 points and from 17 to 45 points, respectively.  Case 7  • Diagnosed with 5q-SMA type 1.  • Lacked SMN1 and had three copies of SMN2 as found by genetic testing.  • Received Nusinersen therapy at 3 years and 8 months.  • One year and 7 months after starting therapy, he gained the ability to sit independently, and CHOP-INTEND scores improved from 21 to 48 points.  Cases 1 and 8  • Both patients were bedridden at the time of therapy.  • After 1 year of Nusinersen administration, treatment effectiveness remained unclear.  3.3. Prevalence of SMA in Shikoku  • Shikoku’s population was 3,756,000 as of October 1, 2018.  • The prevalence of SMA was estimated as 0.56 (95% CI, 0.32–0.80) for all types of SMA, 0.24 (95% CI, 0.08–0.40) for type 1, 0.13 (95% CI, 0.02–0.25) for type 2, 0.13 (95% CI, 0.02–0.25) for type 3, and 0.03 (95% CI, -0.03–0.08) for Type 4 per 100,000 people as of July 1, 2019 |
| Osmanovic 2021 | n/a |
| Osmanovic 2021b | Treatment discontinuation  35.7% (n = 5) of the motor non-responder group versus none in the motor-responder group terminated Nusinersen treatment.  The total RULM score by the time of enrolment was, on average, lower in patients who discontinued Nusinersen therapy compared with patients still undergoing treatment.  A trend toward a longer disease duration in those five patients compared with patients still on treatment was identified.  A mean decrease of −2.40 (SD 1.67) points in the RULM score during a median of 14 months of treatment was recorded in the group of patients who discontinued treatment.  Performance in motor-function scores (RULM and HFMSE) at the time point of Nusinersen treatment initiation was significantly better in the ambulatory patients (HFMSE and RULM p < 0.0001).  HFMSE Score and Disease Duration: The HFMSE score mildly influenced satisfaction with ‘convenience’ (β = −0.68, 95% CI -1.19 to -0.18, p = 0.009) and the disease duration (β = −0.43, 95% CI -0.81 to -0.06, p = 0.024). The HFMSE score also mildly influenced the dimension ‘side effects’ (β = −0.22, 95% CI -0.40 to -0.04, p = 0.017). |
| Osredkar 2021 | Of 61 patients, 33 were evaluated using CHOP-INTEND scale, 17 were evaluated with HFMS, 10 were evaluated with HFMSE, and 1 was evaluated with MFM  After 14 months of treatment, SMA type I (p ¼ 0.002) and type II (p ¼ 0.002) patients had significantly better outcomes, while type III patients showed a trend towards improvement (p ¼ 0.051) on motor scales.  Of all patients, 43/59 patients (72.9 %) showed improvement on the motor scales after 14 months of treatment with Nusinersen; 7/ 59 (11.9 %) patients showed no overall improvement; and 8/59 (13.6%) showed a decline  Ambulation  Altogether, only 6/61 patients (9.8 %) were ambulatory at the first application of Nusinersen. In the 14-month study period, none of the patients lost ambulation.  Overall younger age which is related to an earlier initiation of Nusinersen treatment in the cohort of patients and a higher number of SMN2 copies were related to a better motor outcome |
| Pandey 2025 | Significant improvement in RHS and RULM scores in Nusinersen group compared to SOC group over 12 months.  Nusinersen Group  RHS Scores:  Baseline: 35 ± 18  6 months: 38.9 ± 19  12 months: 39.9 ± 17.3  Statistically significant improvement across time points (p < 0.001).  RULM Scores:  Baseline: 29 ± 8  6 months: 30.1 ± 8.3  12 months: 31.3 ± 6.6  Statistically significant improvement across time points (p < 0.022).  CMAPs (Median and Ulnar Nerve):  No significant cSOC Group  RHS Scores:  Baseline: 28.9 ± 15.7  6 months: 29.6 ± 16.6  12 months: 29.9 ± 17.2  No significant change across time points (p = 0.35).  RULM Scores:  Baseline: 25.7 ± 7.5  6 months: 25.8 ± 7.9  12 months: 26.3 ± 7.2  No significant change across time points (p = 0.54).  CMAPs:  Median nerve: Significant reduction from baseline to 1 year (p = 0.03).  Ulnar nerve: No significant change from baseline to 1 year (p = 0.36).  hange from baseline to 1 year (p > 0.05).  Comparison Between Groups  RHS Scores:  Statistically significant improvement in the Nusinersen group compared to the SOC group (p = 0.02).  RULM Scores:  Significant improvement in the Nusinersen group across time*group interaction (F = 3.527, t = 0.035).  CMAPs and ACEND Scores:  No significant difference between groups from baseline to 12 months.  Secondary Outcomes  2-Point Gain in RHS Scores:  Nusinersen group: 50% at 6 months, 75% at 12 months.  SOC group: 31% at 6 months, 50% at 12 months.  Odds of 2-point gain not statistically significant between groups |
| Pane 2019 | CHOP INTEND  At baseline, the CHOP INTEND scores ranged between 0 and 52 (mean = 15.66, SD = -+-13.48)  n=12 patients (14.11%) showed negative changes between −1 and − 6 (mean = −2, SD = +-1.47)  n=20 (23.52%) remained stable or had a 1-point improvement. Of the remaining 53 (62.35%), 9 improved 2 points, 44 > 2 points, and 38 ≥ 4 points  HINE-2  At baseline, the HINE-2 scores ranged between 0 and 5 (mean = 0.69, SD = +-1.23)  n=4 patients (4.70%) showed negative changes  n=53 (62.35%) remained stable  n=7 had 1-point improvement (8.23%)  n=5 (5.88%) improved 2 points  n=16 (18.82%) improved more than 2 points (sitting independently)  Patients classified as 1.9 and treated at 8 months achieved standing  There was a significant difference between baseline and the 12-month scores on both the CHOP INTEND and the HINE-2 for the whole group (p < 0.001), the subgroups with 2 SMN2 copies (p < 0.001), and those with 3 SMN2 copies (p < 0.001)  Significant difference between baseline and 12 months on the HINE-2 for both the patients with 2 SMN2 copies (p = 0.002) and for those with 3 SMN2 copies (p = 0.012). |
| Pane 2021 | CHOP-INTEND  mean score in the whole cohort was 18.09 (+-14.22) at baseline, 24.81 (+-18.85) at 12 months, and 26.75 (+-19.45) at 24 months  mean changes were 6.72 (+-8.33) between baseline and 12 months, 1.94 (+-3.69) between 12 and 24 month, and 8.66 (+-9.35) between baseline and 24 months  HINE-2  mean score was 0.88 (+-1.33) at baseline, 2.75 (+-3.87) at 12 months, and 3.50 (+-4.96) at 24 months  mean changes were 1.87 (+-3.18) between baseline and 12 months, 0.75 (+-1.92) between 12 and 24 months, and 2.62 (+-4.39) between baseline and 24 months  For both CHOP and HINE2 showed a significant difference between baseline and 24- month scores for the whole group (P < 0.001)  the difference was also significant between baseline and 12-month scores (P < 0.001) and between 12 and 24 months (P < 0.001)  When age subgroups were considered, on the CHOP INTEND the difference was significant between baseline and 24 months in all age subgroups  HINE 2, the difference between baseline and 24 months, between baseline and 12 months was significant in patients < 210 days and between 210 days and 2 years but not in those older than 2 years.  Sitting position  N=21 out of 68 reached the sitting position  N=16 of the 21 sitting reached in the first year of treatment and 6 in the second year  Sitting was achieved in all the seven patients treated before 210 days of age (100%), in 11 of the 23 treated between 210 days and 2 years (55%), and in 3 of the 14 treated between 2 and 4 years of age (17.64%)  Other motor progresses  Out of the 21 “patients who reached the sitting position, 10 were also able to half roll to both sides independently, 5 to roll from prone to supine and/or vice versa, 1 was able to maintain “prone on elbows” position independently, 1 was able to maintain four-point kneeling independently, 1 was able to stand with support, 1 was able to walk with support, and 1 was able to walk independently.” |
| Pane 2022 | HFMSE Results: There was a significant increase between baseline and 12-month in type 2 patients (p = 0.009) but not in type 3 patients (p = 0.130). The increase between baseline and 24 months was significant in both type 2 and 3 patients (p = 0.019; p = 0.017).  SMA type 2, median (All)  Baseline score: 10.6  12 months: 1.6  24 Months: 1.9  SMA type 3, median (All)  Baseline score: 39.5  12 months: 0.9  24 months: 1.5  RULM Results: In type 2 patients, there was a significant increase between baseline and 12 months (p = 0.017) and between baseline and 24 months (p = 0.018). In type 3 patients, the increase was not significant at any time interval (p > 0.05).  SMA type2, median (All)  Baseline score: 14.2  12 months: 1.2  24 months: 1.6  SMA type 3, median (all)  Baseline score: 31.5  12 months: 0.0  24 months –0.0  Comparison with untreated patients with Type 2  When comparing treated and untreated type 2 patients there was no difference for age or HFMSE/RULM score at baseline (p > 0.05). The difference between treated and untreated patients was significant on both HFMSE and RULM both at 12 months (p < 0.001, p = 0.008) and at 24 months (p < 0.001, p < 0.001).  Comparison with untreated patients with type 3  When comparing treated and untreated type 3 patients there was no difference for age or HFMSE/RULM score at baseline (p > 0.05), with the exception of age distribution in patients older than 20 years (p < 0.001). The difference between treated and untreated patients was significant on both HFMSE and RULM at 12 months (p = 0.0002, p < 0.001). At 24 months the difference was significant on HFMSE (p = 0.0002) but not on RULM (p = 0.9225) |
| Pane 2023 | On the CHOP INTEND scale, the 48-month changes ranged between -5 and 40 (mean: 10.6, SD: 12.1). Four patients (8.3%) showed negative changes between -2 and - 5. Another 7 (14.6%) remained stable. Of the remaining 37 (77.1%), 3 improved 2–3 points and 33 improved 4 points or more.  On the HINE scale, the 48-month changes ranged between -2 and 20 (mean: 4.3, SD: 5.7). Two patients (4.2%) showed negative changes between -2 and - 1. Another 24 (50%) remained stable or had a 1-point improvement. Of the remaining 22 (45.8%), 3 improved 2–3 points and 19 improved 4 points or more.  The CHOP INTEND and HINE-II scores significantly increased between baseline and 48 months (p < 0.001)  24 month: 15/48 patients (Mean HFMSE score: 14.7, SD 8.6)  Month 36 (Mean HFMSE score: 18.8, SD 10.6)  48 months follow up (Mean HFMSE score: 18.8, SD 12.4)  There was a significant difference at baseline between patients with different severity of disease (1.1, 1.5, 1.9) in both CHOP INTEND (H (2):17.007, p < 0.001) and HINE-II (H (2):13.804, p = 0.001).  The CHOP INTEND and HINE-II scores did not significantly increase between baseline and 48 months in the 1.1 population (p = 0.144, p = 0.655) but was significantly increased in the 1.5 population (p < 0.001, p = 0.008) and in the 1.9 population (<0.001, <0.001).  There was no difference in the magnitude of changes between patients with different severity of disease (1.1, 1.5, 1.9) in both CHOP INTEND and HINE-II at any time point (p >0.005)  There was a significant difference at baseline between patients with different SMN2 copy number (2 SMN2, 3 SMN2) in the CHOP INTEND (p = 0. 012) but not in the HINE-II (p = 0.111).  The CHOP INTEND and HINE-II scores were significantly increased between baseline and after 48 months in the population with 2 (p < 0.001, p < 0.001) and 3 SMN2 copy number (p < 0.001, p = 0.005).  There was no difference in the magnitude of changes between patients with 2 and 3 SMN2 copy number in both CHOP INTEND and HINE-II at any time point (p > 0.005)  There was a significant difference at baseline between patients with different ages at treatment in the CHOP INTEND but not in the HINE-II.  The CHOP INTEND and HINE-II scores were significantly increased between baseline and after 48 months in the population aged < 210 days and <2 years and for the CHOP INTEND only for the patients aged 2–5 years at the time treatment started.  There was not a significant increase in the HINE-II for the patients aged 2–5 years and in both measures when patients were aged 5–12 years.  There was a significant difference in the magnitude of changes between patients with different ages at first dose of Nusinersen in both CHOP INTEND and HINE-II at any time point, with patients treated at age <210 days having major changes at any time point in respect of the other age categories.  Mixed-Model Analysis: After adjustment for the predefined confounders, the mean CHOP INTEND score in treated patients significantly increased by 1.96 points per visit, and the HINE-II score significantly increased by 0.92 points.  The 6MWT could not be performed at baseline, as none of the  patients were able to walk independently. One patient was able to  perform the 6MWT at 36 months (90 m), at 40 months (143 m) and  at 48 months (165 m). |
| Pane 2024 | CHOP INTEND at Treatment Initiation: Significant difference among feeding outcome groups (p < 0.001). |
| Pechmann 2022 | During the observation period, 16 younger sitters (14.9%) and one older sitter (1.3%) gained the ability to walk independently according to WHO criteria.  Of these, 14 children (82.3%) gained the ability to walk between baseline and m14.  None of the lost sitters or lost walkers gained the ability to walk unassisted.  Four lost sitters (10.8%) and one lost walker (2.6%) gained the ability to sit independently.  In all cohorts, no motor milestones were lost under Nusinersen treatment, in particular, no child lost the ability to sit.  HFMSE  After 38 months of treatment, changes in HFMSE scores were mean + 7.0 points in younger sitters, + 0.1 points in older sitters and + 2.9 points in lost walkers.  After 14 months of treatment, mean change in HFMSE was + 2.5 points in lost sitters.  Clinically meaningful changes in HFMSE score were observed in 63 children (24.6%): 37 younger sitters (34.6%), 11 older sitters (15.1%), five lost sitters (13.5%), and 10 lost walkers (25.6%).  Eleven children (4.3%) lost ≥ 3 points in HFMSE score during the observation period.  Inferential analysis revealed SMN2 copy number as the only covariate having a significant influence on changes in HFMSE score. Further, higher baseline score was associated with smaller improvements in HFMSE score.  RULM  RULM scores improved in all cohorts. At m38, mean changes in RULM score were + 9.1 points in younger sitters, + 2.2 points in older sitters, + 7.3 points in lost sitters, and + 3.3 points in lost walkers.  Clinically meaningful changes in RULM score were observed in 83 children (32.4%): 30 younger sitters (28.0%), 30 older sitters (41.1%), eight lost sitters (21.6%) and 15 lost walkers (38.5%).  Only three older sitters (4.1%) lost ≥ 2 points during the observation period.  Inferential analysis revealed the following covariates to have a significant influence on changes in RULM score: Children with higher baseline score showed smaller improvements than children with lower RULM scores.  HFMSE inferential analysis results  (Intercept) value1.386 SE 0.697 DE 833 t-value 1.987 p-value 0.05  Baseline_m14 versus m14_m26 value0.659 SE0.366 DE833 t-value 1.797 p-value 0.07  Baseline_m14 versus m26_m38 value0.550 SE0.608 DE833 t-value 0.905 p-value 0.37  Age at symptom onset value0.014 SE0.028 DE170 t-value 0.492 p-value 0.62  Baseline score value− 0.044 SE0.021 DE170 t-value − 2.119 p-value 0.04  SMN2 copy number 1.785 SE0.624 DE170 t-value 2.861 p-value 0.005  Age at start of treatment value− 0.009 SE0.005 DE170 t-value − 1.667 p-value 0.1  Gender (m) value0.704 SE0.460 DE170 t-value 1.528 p-value 0.13  Loss of sitting before start of treatment value0.170 SE0.742 DE170 t-value 0.229 p-value 0.82  Loss of walking before start of treatment value0.335 SE0.741 DE170 t-value 0.452 p-value 0.65  RULM inferential analysis  respectively Value SE DF t-value p-value  (Intercept) Value 3.427 SE 0.776 DF 849 t-value 4.416 p-value0.00  Baseline_m14 versus m14_m26 Value 1.122 SE 0.235 DF 849 t-value4.777 p-value< 0.001  Baseline_m14 versus m26_m38 Value 1.509 SE 0.364 DF 849 t-value4.146 p-value< 0.001  Age at symptom onset Value 0.033 SE 0.027 DF 169 t-value1.235 p-value0.22  Baseline Score Value − 0.146 SE 0.033 DF 169 t-value− 4.431 p-value< 0.001  SMN2 copy number Value 0.465 SE 0.621 DF 169 t-value0.749 p-value0.46  Age at start of treatment Value − 0.004 SE 0.005 DF 169 t-value− 0.742 p-value0.46  Gender m Value 0.396 SE 0.456 DF 169 t-value0.869 p-value0.39  Loss of sitting before start of treatment Value − 1.533 SE 0.664 DF 169 t-value− 2.308 p-value0.02  Loss of walking before start of treatment Value 0.805 SE 0.733 DF 169 t-value1.099 p-value0.27 |
| Pechmann 2023 | up to 38 months: 29 children in cohort 1a (33.0%) and six children in cohort 1b (10.9%) gained the ability to sit independently.  In cohort 1a, four children (4.5%) gained the ability to stand, and two children (2.3%) gained the ability to walk.  The probability to gain the ability to sit increased to 44.8% in cohort 1a and 12.9% in cohort 1b at last observation.  In both cohorts, no motor milestones were lost under treatment with Nusinersen.  The greatest improvements were observed within the first 14 months: of all children in cohort 1a who gained the ability to sit, 23 patients (79.3%) achieved the motor milestone between baseline and m14, four (13.8%) between m14 and m26, and only two children (3.4%) between m26 and m38.  CHOP INTEND scores improved in both cohorts, improvements in cohort 1a were greater than in cohort 1b.  Only including patients with CHOP INTEND scores at baseline and m38, in cohort 1a improvements in CHOP INTEND score were mean 12.8 points (n=14), and in cohort 1b mean 3.3 points (n=8).  In untreated children with SMA type 1, CHOP INTEND score is expected to decrease within the first 12 months after diagnosis.  Main improvements were observed in both cohorts between baseline and m14: Between baseline and m14, 32 children (43.8%) in cohort 1a and seven children (14.0%) in cohort 1b experienced an improvement in CHOP INTEND score >4 points. Between m14 and m26, eight children (14.3%) in cohort 1a and four children (9.5%) in cohort 1b, and between m26 and m38 four children (11.8%) in cohort 1a and two children in cohort 1b (6.7%) showed greater improvements than four points in CHOP INTEND score, respectively.  Inferential analysis revealed the following covariates to have a significant influence on changes in CHOP INTEND score: improvements in CHOP INTEND score were significantly smaller in time period m26—m38 compared with the time-period baseline—m14. Further, children with higher baseline CHOP INTEND score showed significant smaller improvements than children with lower CHOP INTEND scores—most likely due to a ceiling effect of the CHOP INTEND. Younger age at start of treatment was associated with larger improvement in CHOP INTEND score. SMN2 copy number was not revealed to have a significant influence on changes in CHOP INTEND. Thus, the better outcome in cohort 1a can be explained by earlier treatment initiation |
| Pechmann b 2023 | Both paediatric and adult patients improved their walking distance in the 6MWT, mean walking distance changed from baseline to m38 by 39.3m in paediatric walkers and 24.4m in adult walkers.  Only considering patients who performed 6MWT at baseline and m38, mean walking distance changed by 83.0m (n = 18) in paediatric walkers and 46.0m (n = 27) in adult walkers  During the observation period, 31 paediatric patients (27.2%) and 31 adult walkers (26.5%) experienced a clinically meaningful improvement of >30m  A walking distance in the 6MWT > 500m was observed in nine paediatric (7.9%) and 18 adult walkers (15.4%) at baseline, and 10 paediatric (18.2%) and nine adult walkers (16.4%) at 38m, respectively  During the first 14 months of treatment, 20 paediatric (19.4%) and 23 adult walkers (21.1%0 experienced improvements in the 6MWT >30m.  Inferential analysis revealed a higher SMN2 copy number as only covariate to have a significant influence on improvements in the 6MWT.  The absence of fatigue and a lower walking distance at baseline were associated with greater improvements in the 6MWT.  HFMSE and RULM: During the observation period of 38 months, mean HFMSE scores improved in paediatric walkers (+5.3 points) and remained stable in adult walkers (–1.4 points). A HFMSE score > 60 points was observed in 22 paediatric (19.3%) and 14 adult walkers (12.0%) at baseline and 17 paediatric (31.0%) and four adult walkers (7.3%) at m38, respectively. Mean RULM scores improved in paediatric patients (+2.8 points) and remained stable in adult patients (–0.5 points). A RULM score > 35 points was observed in 34 paediatric (29.8%) and 79 adult walkers (67.5%) at baseline and 24 paediatric (43.6%) and 31 adult walkers (56.4%) at m38, respectively |
| Peng 2025 | 2 improved motor function  CHOP INTEND Score:  Increased by 5.4 (95% CI -0.6 to 11.4) at 6 months.  Increased by 6.6 (95% CI -1.3 to 14.4) at 10 months.  Increased by 5.5 (95% CI -2.4 to 13.4) at 14 months.  Proportions of responders (increase ≥4 points): 80.0% (6 months), 71.4% (10 months), 50.0% (14 months).  6 completed CHOP INTEND and increased after 6 months and remained stable thereafter  HINE-2 Score:  Increased by 1.2 (95% CI 0.2 to 2.2) at 6 months.  Increased by 0.8 (95% CI 0.2 to 1.3) at 10 months.  Increased by 0.8 (95% CI -0.2 to 1.9) at 14 months.  Proportions of responders: 50.0% (6 months), 28.6% (10 months), 50.0% (14 months).  HFMSE Score:  Increased by 3.2 (95% CI 1.1 to 5.4) at 6 months.  Increased by 3.5 (95% CI 1.8 to 5.1) at 10 months.  Increased by 5.0 (95% CI 2.5 to 7.4) at 14 months.  Proportions of responders (increase ≥3 points): 48.0% (6 months), 51.6% (10 months), 59.1% (14 months).  RULM Score:  Increased by 1.4 (95% CI 0.1 to 2.7) at 6 months.  Increased by 1.4 (95% CI 0.3 to 2.6) at 10 months.  Increased by 2.4 (95% CI 0.7 to 4.1) at 14 months.  Proportions of responders (increase ≥2 points): 33.3% (6 months), 37.9% (10 months), 54.5% (14 months).  CMAP Amplitude  Proximal Left Median Nerve: Increased by 0.69 ± 1.63 mV at 6 months.  Proximal Left Tibial Nerve: Increased by 0.3 ± 0.4 mV at 10 months.  Proximal Right Ulnar Nerve: Increased by 0.64 ± 0.98 mV at 10 months.  Bilateral Tibial, Median, and Ulnar Nerves: Statistically significant increases at 14 months.  Greatest improvements: 0.87 ± 1.41 mV (tibial), 1.08 ± 1.71 mV (median), 0.59 ± 1.01 mV (ulnar). |
| Pera 2021 | HFMSE 12-Month Changes:  Complete data both at baseline and at 12 months were available in 104/144 (72.22%) patients for the HFMSE.  In the 104 patients with HFMSE scores available at baseline and after 12 months/6 infusions, the HFMSE scores significantly increased between the two assessments (mean difference = 1.18 (95%CI: 0.37–1.99), p = 0.004).  RULM 12-Month Changes:  Complete data both at baseline and at 12 months were available in 100/144 (69.44%) patients for the RULM.  In the 100 patients with RULM score available after 12 months/6 infusions, RULM scores significantly increased from baseline to 12 months (mean difference = 0.58 (95%CI: 0.12–1.04)).  6MWT 12-Month Changes:  Complete data both at baseline and at 12 months were available in 51/79 (64.56%) walker patients for the 6MWT assessment.  In 51 patients with 6MWD available at baseline and after 12 months/6 infusions, the 6MWT did not significantly increase from baseline to 12 months (mean difference = 6.65 (95%CI: -7.08–20.37)).  Longitudinal Data Analysis:  In a multivariable analysis including 130/144 (90.28%) patients with at least 6-months follow-up, no significant differences between the different SMA III subtypes (p for interaction between time and SMA type = 0.541).  In a multivariable analysis including the 79 walker patients who had at least 6-months follow-up, there was a difference in the slope according to the SMA subtype (p for interaction between time and SMA type = 0.002). |
| Pinar 2024 | Increase in HFMSE: 29.5 to 30.5 (p = 0.023).  Significant increase in HFMSE for SMA type 2 patients: 28 to 29 (p = 0.011). |
| RAINBOWFISH (NCT03779334) (EMA 2023) | Motor Function Results (HINE-2 Assessment):  Proportion of patients achieving a maximum score (26) on HINE-2 – 28.6%  Proportion of patients able to sit as assessed by HINE-2 (stable sit) 14.3%  Proportion of patients able to sit as assessed by HINE-2 (Pivots, rotate) 85.7%  Proportion of patients able to roll as assessed by HINE-2 (Prone to supine) 14.3%  Proportion of patients able to roll as assessed by HINE-2 (Supine to prone) 85.7%  Proportion of patients able to crawl as assessed by HINE-2  On Elbow – 14.3%  Crawling flat on abdomen – 14.3%  Crawling on hands and knees – 71.4%  Proportion of patients able to stand as assessed by HINE-2  Does not support weight – 28.6%  Stands with support – 28.6%  Stands unaided – 42.9%  Proportion of patients able to walk as assessed by HINE-2  Bouncing – 14.3%  Walking independently – 42.9%  Cannot test 28.6%  Not done 14.3%  At Month 12, all patients were sitting without support.  Six of 7 patients achieved the highest levels of sitting (pivot/rotate); the remaining 1 patient with 2 SMN2 copies and baseline CMAP <1.5 mV achieved a stable sit.  Six of 7 patients achieved the highest levels of rolling (supine to prone) and the remaining 1 patient with 2 SMN2 copies and baseline CMAP <1.5 mV achieved prone to supine rolling.  Five of 7 patients could stand with support or stand unaided, the other 2 patients, both with 2 SMN2 copies and baseline CMAP <1.5 mV did not support weight.  Three of 7 patients (including 1 patient with 2 SMN2 copies and baseline CMAP >1.5 mV) were walking independently and 1 patient was able to bounce.  Motor Function Results (CHOP-INTEND Assessment):  Proportion of patients achieving a maximum CHOP –INTEND score (64) - 57.1%  Proportion of patients who achieve a score of 40 or higher – 100% (95%CI 65.2 - 100.0)  Proportion of patients who achieve a score of 50 or higher – 100% (65.2 - 100%)  Proportion of patients who achieve a score of 60 or higher – 71.7% (34.1 - 94.7%)  Patients achieved high levels of motor function prior to Month 12, with 10 of 13 patients (76.9%; 90% CI: 50.5, 93.4) achieving scores of ≥60 at Month 4 (Week 16).  Four of 7 patients achieved the maximum score of 64 at Month 12.  CMAP Amplitude:  At baseline, the median CMAP amplitude for all patients (n=18) was 3.6 mV (range: 0.5-6.7 mV).  Of the 7 patients who had reached Month 12 by the time of the CCOD, 5 of 7 patients showed an increase in CMAP amplitude between baseline and Month 12. The remaining 2 patients (both with 2 SMN2 copies) had the same CMAP amplitude at baseline and Month 12  Achievements after 12 months of treatment with Risdiplam:  All patients developed complex motor abilities as assessed by the HINE-2 scale, including achieving the ability of sitting without support (HINE-2 categories of ‘Stable sit’ and ‘Pivots [rotates]’).  Patients achieved high levels of motor function illustrated by the median score on the CHOP-INTEND equal to the maximum score of the assessment at Month 12. Even the 2 patients with a baseline CMAP amplitude < 1.5 mV increased their motor function over time  Patients with 2 SMN2 Copies:  Of the 4 patients with 2 SMN2 copies, 1 could stand unaided and walk independently, and 1 could stand with support and bounce at Month 12. The latter patient gained the ability to stand unaided and walk while holding on (“cruising”) by Month 18 of treatment (at the time of the CCOD). The remaining 2 patients with 2 SMN2 copies had low CMAP amplitude values at baseline (0.46 and 0.6 mV). Despite this finding, both patients were developing complex motor abilities, such as rolling and crawling (1 patient crawled on elbow and 1 crawled flat on abdomen) after 12 months of Risdiplam treatment (their last visit before the CCOD).  Patients with 3 or ε4 SMN2 Copies:  Among the patients with 3 or ε4 SMN2 copies, 2 patients could stand unaided and walk independently, and 1 patient could stand with support at Month 12. The latter patient (with ε4 SMN2 copies) gained the ability to stand unaided and walk independently by Week 64 (approximately Month 15) of treatment. |
| Rich 2022 | Correlations Between Nf Levels and Functional Outcomes:  In the non-ambulatory cohort, multiple negative correlations were observed between baseline serum pNfH and measures of extremity strength  Prior to Nusinersen treatment, serum pNfH was negatively correlated with RULM (r = -0.822, p = 0.04), upper extremity strength (r = -0.828, p = 0.042), lower extremity strength (r = -0.860, p = 0.028), and total strength (r = -0.870, p = 0.024)  In the ambulatory cohort, negative correlations were observed in three baseline functional outcomes; CMAP with serum NfL (r = -0.959, p = 0.04), SMAFRS with CSF NfL (r = -0.887, p = 0.045), and HFMSE with CSF pNfH (r = -0.919, p = 0.027)  Correlation Between SMN2 Copy Number and CSF NfL:  A positive correlation was identified between SMN2 copy number and CSF NfL at baseline and at maintenance dose 1 (baseline r = 0.461, p = 0.04; 180 days r = 0.490, p = 0.02)  When stratified by ambulatory status, significant correlations were observed between SMN2 copy number and CSF NfL in the non-ambulatory cohort (baseline r = 0.572, p = 0.004, maintenance dose 1 r = 0.606, p = 0.01), but not in the ambulatory cohort (baseline r = 0.448, p = 0.450, maintenance dose 1 r = 0.697, p = 0.191)  Ambulatory patients had significantly greater SMN2 copy number than non-ambulatory patients (ambulatory SMN2 copy number mean = 3.75, SD = 0.622; non-ambulatory SMN2 copy number mean = 3.10; SD = 0.388; p = 0.004)  Nf levels correlate with functional outcomes in adult SMA patients  In the non-ambulatory cohort, we observed multiple negative correlations between baseline serum pNfH and measures of extremity strength. In addition, in the non-ambulatory cohort baseline serum pNfH was negatively correlated with upper extremity strength (r = -0.828, p = 0.042) lower extremity strength (r = -0.860, p = 0.028) and total strength (r = -8.70, p = 0.024). None of these correlations remained significant at subsequent times.  Ambulatory cohort: negative correlations were identified in three baseline functional outcomes; CMAP with serum NfL (r = –0.959, p = 0.04),, SMARFRS with CSF NfL (r = –0.887, p = 0.045), and HFMSE with CSF pNfH (r = –0.919, p = 0.027). None of these correlations remined significant |
| Sarikaya 2022 | Median values of CHOP-INTEND and HMSF-E scores for each dose  Observation: CHOP-INTEND and HMSF-E scores increased significantly as patients received treatment  A statistically significant negative correlation was observed between the age of treatment onset and the increase of CHOP-INTEND, after the first four loading doses  A statistically significant negative correlation was found between the increase in HMSF-E at all Nusinersen doses and the age at onset of treatment in group 2  Conclusion: The earlier the treatment was initiated, the greater was the increase in HMSF-E and CHOP-Intend scores  Change in maximum points on motor scales between the pre-first and pre-seventh doses  Tables:  the effect of age at first application on increase of motor scale scores  Spearman’s rho: CHOP-INTEND /HMSFE 2–1; CHOP-INTEND /HMSFE 3–1; CHOP-INTEND /HMSFE 4–1; CHOP-INTEND /HMSFE 5–1; CHOP-INTEND /HMSFE 6–1; CHOP-INTEND/HMSFE 7–1  Group 1 Correlation coefficient 0.152/0.003/ 0.001/ _0.693/ _0.663/ _0.817  Sig. (two-tailed) 0.620/ 0.993/ 0.996/ 0.018/ 0.073/ 0.025  Patient number (n) 13/ 13/ 13/ 11/ 8 /7  Group 2 Correlation coefficient: _0.400/ _0.656/ _0.664/ _0.740/ _0.747/ _0.739  Sig. (two-tailed) 0.156/ 0.011/ 0.010/ 0.006/ 0.008 /0.009  Patient number (n) 14/ 14 /14 /12 /11/ 11 |
| Scheijmans 2022 | SMA Type 1: Twenty-three patients with SMA type 1 were included in the study. Nine patients learned to sit after 3–13 injections. CHOP-INTEND scores for 21 patients with SMA types 1b and 1c are shown in figure 1A. 18 (85%) showed an improvement of ≥4 points. There was a mean increase in CHOP-INTEND scores from baseline:  of 3.3 (CI 2.7 - 4.0) points after 6 months of treatment  6.0 points after 12 months of treatment  8.0 points after 18 months of treatment  9.6 points after 24 months of treatment  10.4 points after 30 months of treatment  10.6 points after 36 months of treatment  HINE-2 scores of 17 patients did not deteriorate during follow-up. Shorter disease duration at treatment initiation was associated with better treatment response (p < 0.001). None of the children showed a decline of motor function during treatment.  SMA Type 2: 30 patients with SMA type 2 were included in the study. Four children with SMA type 2a regained the ability to sit independently, one child with type 2a gained the ability to walk with assistance, and two children with SMA type 2b learned to stand without support. HFMSE scores increased in a non-linear fashion with a mean increase of 1.8 points after 6 months, 3.1 points after 12 months, 3.9 points after 18 months, 4.3 points after 24 months and 4.1 points after 30 months of treatment.  SMA Type 3: Twelve out of 16 children (75%) with SMA type 3a were ambulant at the start of treatment. One child regained the ability to stand alone after seven injections but lost this milestone 4 months later. Increases in HFMSE scores were non-linear, with a mean increase of 1.8 points after 6 months, 3.2 points after 12 months, 4.1 points after 18 months, 4.6 points after 24 months and 4.6 points after 30 months of treatment, all in comparison to baseline scores. After 24 months, motor function reached a relative plateau. Shorter disease duration at treatment initiation was associated with better treatment response. Ten (63%) children showed an improvement of ≥3 HFMSE points. Four patients (25%) showed a decline of motor function, losing up to 3 HFMSE points.  Two infants presymptomatic showed improvements in motor function after starting treatment |
| Schneider 2021 | The numbers of motor units recorded from the abductor pollicis brevis muscle were lower in patients with SMA compared to healthy controls (p < 0.001), even though CMAP amplitude did not differ between the two groups (p = 1).  Compared to patients with ALS, the number of motor units and peak CMAP amplitude were higher in patients with SMA (motor units, p = 0.03; CMAP, p = 0.005).  Axonal Loss and Re-innervation:  Axonal loss was increased, reflected by a decrease of the D50 value, and measures of re-innervation showed larger motor unit amplitudes in patients with SMA compared to healthy controls (D50, p = 0.007; mean unit amplitude, p < 0.001; largest amplitude, p < 0.001; median amplitude, p < 0.001).  Compared to patients with ALS, axonal loss reflected by the D50 value was less pronounced in patients with SMA (p = 0.32). The median amplitude expressed as the percentage of the CMAP was higher in ALS (p = 0.03); other variables did not differ significantly.  Correlation with Clinical Scores:  Electrophysiological measures of patients with SMA showed a moderate to strong statistical correlation with clinical scores (HFSME, RULM). In particular, the number of motor units showed a strong correlation with both HFMSE (Rho = 0.67, p = 0.009) and RULM (Rho = 0.63, p = 0.01).  Analysis of MScanFit Variables and Clinical Scores:  The analysis of MScanFit variables and clinical scores showed no significant differences between the first (study entry) and second investigation (follow-up) (n = 10).  However, the subgroup analysis of patients with SMA with the ability to walk (n = 6) versus wheelchair users (n = 4) demonstrated an improvement of the number of motor units at follow-up in patients with an ability to walk (p = 0.046), accompanied by a reduced median amplitude (p = 0.03). Other variables showed no relevant changes.  Figure 2:  MScanFit Variables of Patients with SMA, ALS, and Healthy Controls:  Peak CMAP Amplitudes (a): The peak CMAP amplitudes are not significantly different between patients with SMA and healthy controls. The values are as follows:  SMA: 7.64 [4.05, 10.88]  NC (Healthy Controls): 8.1 [6.11, 9.17]  ALS: 3.31 [1, 4.54]  Number of Motor Units (b): The number of motor units show significant changes of patients with SMA and ALS compared to controls. The values are as follows:  SMA: 43 [25, 66]  NC (Healthy Controls): 92 [67, 120]  ALS: 9 [5, 42]  D50 Values ©: The D50 values show significant changes of patients with SMA and ALS compared to controls. The values are as follows:  SMA: 30 [15, 35]  NC (Healthy Controls): 34 [32, 38]  ALS: 17 [6.5, 32]  Mean Unit Amplitude (d): The mean unit amplitude shows significant changes of patients with SMA and ALS compared to controls. The values are as follows:  SMA: 165 [131.3, 211.7]  NC (Healthy Controls): 74.9 [62.9, 111.9]  ALS: 166.7 [126.4, 301.45]  Please note that one of the 10 investigated patients with ALS could not be included in the data analysis as no reproducible motor unit potential could be evoked. The horizontal solid lines indicate medians, and dashed lines represent first and third quartiles (Q1, Q3). Asterisks indicate the p values (*p < 0.05, **p < 0.01, ***p < 0.001, ****p < 0.0001, *****p < 0.00001).  Figure 3:  Correlations of Clinical Scores (HFMSE and RULM) and Electrophysiological Measures:  Number of Motor Units (a, b): The number of motor units shows a moderate to strong correlation with clinical scores. The correlation values are as follows:  HFMSE: Rho = 0.67, p = 0.009  RULM: Rho = 0.63, p = 0.01  CMAP Amplitude (c, d): The CMAP amplitude shows moderate to strong correlations with clinical scores. The correlation values are as follows:  HFMSE: Rho = 0.54, p = 0.046  RULM: Rho = 0.75, p = 0.001  Please note that results with p < 0.05 were considered significant. This indicates that there is a statistically significant correlation between the number of motor units, CMAP amplitude, and the clinical scores (HFMSE and RULM).  Figure 4:  Prospective Analysis of MScanFit Variables of Patients with SMA Treated with Nusinersen:  An overall analysis of the number of motor units and CMAP amplitude showed no significant changes between study entry and follow-up:  Number of Motor Units (a): p = 0.09  CMAP Amplitude (b): p = 0.24  A subgroup analysis identified patients with an ability to walk to have significantly more motor units (MSF Units) at follow-up:  Study Entry: 55 [35.75, 66.5]  Follow-up: 66.5 [37.5, 82.5]  Significance: p = 0.046  This change was accompanied by a significant reduction of median amplitude (MSF Median Amp):  Study Entry: 1.5 [1.3, 2.6]  Follow-up: 1.23 [0.83, 2.15]  Significance: p = 0.03  Please note that results with p < 0.05 were considered significant. The asterisks indicate p < 0.05.  This included extra analysis of clinical scores in this subgroup, also adding the 6-min walk test (p = 0.17). There was no significant difference in the number of Nusinersen courses between wheelchair users and patients with the ability to walk |
| SHINE (CS11) (NCT02594124 (Dunaway Young 2023) | • HFMSE and RULM: Children without scoliosis at baseline (Cobb angle <10°) had the greatest mean improvement from baseline in HFMSE (6.0-point increase) and RULM (6.1-point increase) at Day 930. Those with mild scoliosis (Cobb angle >10° to ≤20°) had a smaller mean improvement from baseline in HFMSE (3.9-point increase) and RULM (4.6-point increase) at Day 930. Children with moderate scoliosis (Cobb angle >20° to <40°) showed stabilization of HFMSE (a 0.7-point mean increase from baseline at Day 930) and RULM (a 2.3-point mean increase from baseline at Day 930). The proportions of HFMSE and RULM responders at Day 930 were 80% (20/25), 59% (22/37), 42% (14/33), and 80% (20/25), 72% (26/36), and 55% (18/33) for participants with no, mild, and moderate scoliosis, respectively.  • WHO Motor Milestones: The proportion of participants achieving WHO motor milestones at baseline and up to Day 930 was assessed according to scoliosis severity at baseline. In all subgroups and across all milestones, the proportion of participants achieving a given motor milestone was stable or higher at subsequent visits. Seven participants lost WHO motor milestones—three in the no scoliosis group, two in the mild scoliosis group, and two in the moderate scoliosis group. Participants with no scoliosis (Cobb angle <10°) or mild scoliosis (Cobb angle >10° to ≤20°) were more likely to achieve WHO motor milestones of hands-and-knees crawling, standing with assistance, or walking with assistance by Day 930 than those with moderate scoliosis (Cobb angle >20° to <40°). At baseline and at Day 930, all participants were able to sit without support. The proportion of children able to crawl on hands and knees increased across all three subgroups, with the proportion both at baseline and at Day 930 higher in subgroups with no scoliosis and mild scoliosis compared with the subgroup of moderate scoliosis. The proportion of participants who were able to stand with assistance also increased across all subgroups, with the greatest increase among those in the mild scoliosis subgroup. The proportion of participants achieving the WHO motor milestones of walking with assistance or standing or walking alone increased slightly or remained stable in the no scoliosis or mild scoliosis subgroups, whereas participants in the moderate scoliosis subgroup never achieved these motor milestones at any visit in the analysis.  Specific motor milestones:  Long-term follow-up data shows additional motor milestone achievements for infants receiving Spinraza who transitioned from ENDEAR to SHINE. Data from the interim analysis (June 15, 2017) show after 576 days, approximately 45% of infants achieved full head control and 29% achieved sitting independently.  ENDEAR to SHINE motor milestones:  Among patients who received sham control in ENDEAR and began Nusinersen in SHINE, new improvements in total HINE-2 motor milestones and general motor function as measured by CHOP INTEND occurred in SHINE.  Among those who were protocol-defined responders at the last available assessment for motor milestones and general motor function, some of them were achieved as late as day 578 and 818, respectively. Supporting that some patients may take considerable time to respond to therapy.  % full head control: Baseline; 0, day 64; 7, day 183; 17, day 302; 25, day 394; 33, day 578; 45, day 689; 35  % independent sitting: Baseline; 0, day 64; 1, day 183; 5, day 302; 10, day 394; 15, day 578; 29, day 689; 24.  ‘A total of 89 patients transitioned from ENDEAR, 65/81 previously randomised to Nusinersen and 24/41 to sham control.  Patients who received Nusinersen for the first time in SHINE had 2 SMN2 copies (except 1 patient), a median age of 18 months, and a lower mean CHOP INTEND score at baseline in SHINE compared with those treated with Nusinersen in ENDEAR.  The mean (95% CI) change in HINE-2 total score from Nusinersen initiation to last observed visit was 1.1 (0.20–1.90) for patients who received sham control in ENDEAR and Nusinersen in SHINE (n=20/24) and 5.8 (4.58–7.04) for those who received Nusinersen in ENDEAR and SHINE (n=74/81; pooled ENDEAR/SHINE data). NB, these data are based on the last observed visit available for each participant, including those who died or discontinued treatment.  For patients who received Nusinersen in ENDEAR and SHINE, 23/81 (28%) had achieved full head control and 12/81 (15%) independent sitting as their highest motor milestone (overall cohort; at the last available assessment); no patients had yet achieved standing unaided or walking independently, although patients were gaining HINE sub-milestones in both categories. The percentages of patients who received Nusinersen in ENDEAR and SHINE and achieved full head control and independent sitting at different study visits (based on patients who attended those study visits only) |
| Simic 2022 | A statistically significant difference in the number of points on the CHOP INTEND scale was seen between SMA type 1 patients who received a different number of Nusinersen doses (H test = 17.9; df = 8; p = 0.02).  The most significant differences were seen between SMA type 1 patients who received the 6th, 7th, 8th, and 9th doses of Nusinersen compared to baseline values.  Patient data were also analysed in four groups (before treatment, 1–3 doses group, 4–6 doses group, and 7–9 doses group).  CHOP INTEND values differed significantly in patients who received different numbers of Nusinersen doses (H test = 11.74; df = 3; p = 0.01).  The CHOP INTEND value was significantly higher in patients in the 7th to 9th dose group (second year of therapy or 12–24 months after therapy onset) compared to baseline values, in patients in the group from 1st to 3rd dose, and in the group from 4th to 6th dose.  A statistically significant difference was also observed in HFMSE scores between patients with SMA types 2 and 3 who were administered a different number of Nusinersen doses.  The most significant differences in HFMSE scores were seen in patients who received the 8th dose (U = 1; Z = −2.37; p = 0.02), the 6th to 10th dose (U = 32.5; Z = −2.02; p = 0.04), and after 12 months of total treatment duration (U = 1; Z = −2.37; p = 0.02). |
| Simsek 2024 | The median HFMSE was 14 at V1 and 18 at V2 in the total SMA patient group  Clinically meaningful improvements (≥3 points increase) in HFMSE scores were observed in n=12 (57.1 %) of the n=21 patients at V2.  The median change in the HFMSE score at V2 compared to V1 was 3 points (p < 0.001)  There was a significant improvement in the HFMSE after treatment which was observed in patients with three SMN2 gene copies and patients with four SMN2 gene copies (p = 0.02, p = 0.008, respectively)  Summary of motor function  The physical component score and the mental component score of the SF-36 were significantly lower in the SMA patient group at baseline than in the healthy group  The median HFMSE scores significantly improved at V2 in both ambulatory and non-ambulatory SMA patients (p < 0.05)  The median MRC score significantly increased at V2 in the ambulatory SMA patient group (p = 0.04) but not in the non-ambulatory SMA patient group (p = 0.19  The loading Nusinersen treatment significantly improved motor function scores, and muscle strength |
| Sitas 2024 | In the 6MWT, a change of 30 m is considered to be clinically meaningful.  Improved on at least one motor scale = 7/31 (22.6%)  Increase in RULM = 5 patients  Increase in RHS = 2 patients  All of these patients have SMA 3 (3 ambulant and 4 sitters)  Of six patients who were tested with RHS, three had a meaningful improvement.  4 functionally weaker patients: Meaningful improvement in upper limb function.  2 patients: Significant decline in one motor function outcome measure but remained stable in the other two tested outcomes.  3 Patients: lost more than 1 point on RULM, and 1 patient lost a point on RHS as well.  If patients’ motor function doesn’t stabilize or improve in the future, they will be considered non-responders (5/31, 16.1% of patients in the cohort, one SMA2 and four SMA3). |
| SM201 (EMA 2017) | summary of the results:  • CHOP INTEND TOTAL SCORE: An increase of at least 4 points compared to the Baseline was observed on Day 64 (7/13 subjects), Day 183 (8/10 subjects), and Day 302 (3/5 subjects). One subject showed a decrease of at least 4 points on Day 64.  • MODIFIED SECTION 2 – HINE: All subjects had age-appropriate motor development. Additional HINE motor milestones compared to Baseline were achieved on Day 64 (12/13 subjects), Day 183 (10/10 subjects), and Day 302 (5/5 subjects).  • HINE more categories with improvement than with worsening: Improvement was seen in 69% of categories on Day 64, and 100% of categories on Day 183 and Day 302.  • Motor Milestones: At least 7 months at data cut-off, 5/7 subjects were sitting independently. At least 8 months at data cut-off, 1/5 subjects were standing unaided. At less than 8 months of age, 2 subjects were standing with support. At least 11 months of age, 1 subject was walking with support (cruising).  • Weight: All but 1 subject gained weight during the study.  • CMAP increases: Increases of at least 0.5 mV compared to the Baseline were observed on Day 64 (7/11 subjects), Day 183 (9/10 subjects), and Day 302 (3/5 subjects).  • Updated results provided during the review (EMA 2017 p78): From Baseline to the last study visit, 13/18 subjects achieved maximum scores for HINE motor milestones in the categories of Head control and kicking, and 10/18 subjects for Sitting. 12/18 achieved independent sitting, 3/18 subjects achieved independent standing, and 2/18 subjects achieved independent walking.  • CHOP INTEND total score: 16/18 subjects in the Efficacy Set achieved and maintained improvements. 7/18 achieved the highest attainable score at the data cut-off date for this interim analysis.  • From FDA 2016: No subject met the primary endpoint of death or respiratory intervention. 4/10 subjects developed clinically manifested SMA. No subjects had died by the time of data reporting. The mean HINE score increased from 3.1 at baseline to 25 after 1 year. 5 subjects became able to sit independently, 1 became able to stand unaided, 2 other subjects became able to stand with support, and 1 subject became able to walk with support. |
| SUNFISH (NCT02908685) (Mercuri 2022, Mercuri 2023, Oskoui 2023) | 1. Motor Function Measure (MFM) Results: At month 24 of Risdiplam treatment, 32% of patients demonstrated improvement from baseline in MFM32 total score, and 58% showed stabilization. A treatment difference of 3.12 in favour of Risdiplam was observed in MFM-derived scores compared with an external comparator.  2. Motor Function Improvement: Patients showed improvement or stabilization in motor function scores (MFM32, RULM, and HFMSE) at Month 24 of Risdiplam treatment. Greater improvements were observed in younger patients.  3. Marked Improvements at Month 24:  o MFM32 total score: 66.7% of patients aged 2–11 years and 47.4% of patients aged 12–25 years achieved ≥3-point improvement.  o HFMSE total score: 45.2% of patients aged 2–11 years and 26.3% of patients aged 12–25 years achieved ≥2-point improvement.  o RULM total score: 58.1% of patients aged 2–11 years and 57.9% of patients aged 12–25 years achieved ≥2-point improvement.  4. Age-Related Trends in Improvement: The youngest patients (2–5 years) showed the most significant improvement, while no improvement was observed in the oldest age group (18–25 years).  5. SMN2 Copy Number: Patients with two SMN2 copies declined in motor function, while patients with three or four SMN2 copies improved from baseline in MFM32, RULM, and HFMSE total scores.  6. CADTH: 38.3% of patients in the Risdiplam arm were considered responders, compared to 23.7% in the placebo group.  7. Change from Baseline in RULM Total Score at Month 12 (Family 3): The mean change from baseline was 1.61, with a treatment difference of 1.59 in favour of Risdiplam.  8. Change from Baseline in Total Score of the HFMSE at Month 12 (Family 4): The mean change from baseline was 0.95, with a treatment difference of 0.58 in favour of Risdiplam.  9. Subgroup Analysis of Change from Baseline in MFM32 at Month 12 by Region: Treatment differences varied by region, with the highest observed in Europe and the lowest in Japan.  10. CADTH: A total of 69.6% of patients in the Risdiplam arm achieved a change from baseline of 0 or more in the MFM-32 total score at month 12, compared to 54.2% of patients in the placebo group. The difference translates into an odds ratio of 2.00 for Risdiplam versus placebo |
| Szabo 2020 | SMA type 1  Motor function improved in all n=7 patients who performed the motor evaluation on Day 307 and the improvement was >4 points in each case  Average improvement was 14.9 points (±5.1, range 7-21) (p =0.016)  SMA type 2  N=16 completed the follow-up visit at Day 307. The average motor performance measured by HFMSE improved significantly  On Day 307 of the treatment, the average improvement was 7.2 points (±5.0, range -2-17) point increase from baseline (p < 0.001) on the Hammersmith Functional Motor Scale Expanded (HFMSE) and 4.3 (range: 2-9) point increase (p =0.031) on the Revised Upper Limb Module (RULM) were found.  Statistically significant correlation between the age at treatment initiation and the change in HFMSE score at Day 307 of the treatment (p=0.008, R2 0.409, Slope -0.984 (95% Confidence Intervals -1.7 to -0.31)  SMA type 3  N=15 s completed the follow-up visit on Day 307, of whom 12 children were ambulant  Average motor performance measured by HFMSE improved significantly by 5.3 points (±4.4, range -1-13, p=0.001)  The distance walked during the 6MWT improved in most of the patients, a significant increase of 33.9 m was found on the 307th day of treatment (±44.0range -16.3e106.5 m, p =0.007) |
| Tachibana 2022 | in patients with infantile-onset SMA, HINE-2 score increased from 0.66 ± 2.23 at the first assessment to 1.60 ± 3.59 at the last assessment (change= 0.94 ± 2.18), and HFMSE score increased from 0.5 ± 1.3 to 1.4 ± 3.6 (change= 0.9 ± 2.4).  In patients with later-onset SMA, HINE-2 score increased from 8.20 ± 6.69 at the first assessment to 9.03 ± 6.89 at the last assessment (change= 0.83 ± 2.06), HFMSE score increased from 16.7 ± 17.7 to 18.6 ± 19.0 (change= 1.9 ± 4.6), and 6MWT increased from 192.04 ± 31.12 meters to 219.13 ± 33.54 meters (change= 27.09 ± 9.81 meters).  Nusinersen was assessed to be ‘effective’ in 398 of 398 patients (100.0%, including 62.3% ‘improved’ and 37.7% ‘unchanged’) for CGI-I I and 397 of 398 (99.7%, including 61.6% ‘improved’ and 38.1% ‘unchanged’) for CGI-I II |
| Tachibana 2023 | The HINE-2 score in patients with infantile-onset SMA w improved at all assessment time points.  - A negative correlation was observed in the HINE-2 score assessed at 10, 14, and 18 months.  - HFMSE score and 6MWT in patients with later-onset SMA improved until 27 months and 33 months.  - A negative correlation was observed with type II SMA patients between SMA onset to treatment and change in HFMSE score assessed at 9, 15, and 21 months.  - A correlation between time from SMA onset and change in HFMSE score was not observed in patients with type III SMA.  A negative correlation was observed between the time from SMA onset to treatment and change in 6MWT assessed at 9, 15, and 21 months in patients with type III SMA.  - Type II (28.9%) and III (37.1%) SMA patients with ≥3 HFMSE scores increased from baseline  - Patients whose HFMSE score increased from baseline by ≥3 was observed in almost all age groups.  Most patients experienced improvement (gain) or stabilization (no change) in individual items on the HFMSE over time.  - Nusinersen was ‘effective’ in 520/520 patients (100%, including 67.3% ‘improved’ and 32.7% ‘unchanged’)  - CGI-I I (degree of improvement was ‘unchanged or better’ at any time point) and 512/520 patients (98.5%, including 65.4% ‘improved’ and 33.1% ‘unchanged’)  - CGI-I II (the total of the values at each assessment was ≥0) |
| Trancho 2024 | The average age for the SMA and control groups was 32.9 years old (Range 12.7–56.8, SD = 15.4) and 28.1 years old (Range 13.3–53.5, SD = 14.4), respectively  participants in each group ere predominantly male  There were no significant differences in age or sex between the groups  In the 13 participants receiving Nusinersen, the average duration of treatment was 1 year (SD = 0.3)  Motor function  Participants with SMA walked approximately half the distance of control participants during the 6MWT (p < 0.001)  Participants with SMA completed the TUG in an average of 21.3 s, while control participants completed it in an average of 4.3 s (p < 0.001).  Participants with SMA reported a median Total METmin/week nearly half that of controls on the IPAQ-SF (p = 0.05).  Median sitting time in participants with SMA was 480 min (IQR = 240–600), while median sitting time in controls was 420 min (IQR = 360–480).  Participants with SMA had significantly lower bone mineral density (BMD) than control participants in most regions measured |
| Trucco 2023 | n/a |
| Trucco 2024 | Motor Function Status:  96% of SMA type 2 patients were functionally sitters at V0 and maintained their motor function status throughout the study.  All SMA type 3 patients were type 3A and functionally sitters.  Comparison Cohort: 103 patients from the iSMAc natural history cohort (84 SMA type 2, 19 SMA type 3).  Baseline Similarity: No significant differences in age, SMA type, and FVC% pred. at baseline between treated and untreated cohorts |
| Tscherter 2022 | Motor Function Evaluation in SMA Patients:  Motor Assessments Used: CHOP–INTEND, HINE, HFMSE, RULM, and 6MWT.  Motor Abilities Assessed: 13 different qualitative motor functions (e.g., holding head up without support, walking with assistance, raising hands to mouth in a sitting position).  Patients: All 11 SMA type 1 patients were assessed. Six patients were < 18 months old at treatment initiation (5 patients < 4 months old, type 1b and one 1.45 years old, type 1c), and 5 patients were 5.4–16.1 years old (one type 1b, 4 types 1 c).  At Treatment Start:  One individual was able to hold their head unsupported.  One was able to roll onto their side.  Three could raise their hands to mouth in a sitting position.  During Nusinersen Treatment:  8 of 11 patients acquired between 1 and 7 motor abilities (median 4).  One patient lost a motor ability (“raising hands to mouth in a sitting position”).  The highest motor ability, which three children achieved, was standing with assistance.  The children aged < 18 months at treatment start acquired the most motor abilities.  At Last Assessment:  All 6 patients were able to roll onto the side.  4/6 were able to sit without support (at the age of 11–46 months, one type 1c > 12 months old at treatment start).  All were able to raise their hands to mouth in a sitting position.  Three were able to reach overhead in a sitting position.  CHOP-INTEND Scores:  At treatment initiation, patients achieved a median total score of 25 (range 2–29).  All achieved higher total scores at the last assessments than at treatment initiation (an increase of 2–42 points; median 25).  The patients treated earlier (treatment start < 18 months of age) achieved larger improvements (an increase of 25–42 points; median 29.5) than patients treated later (an increase of 2–8 points; median 5).  Of the three patients with a CHOP INTEND of < 10 at the start of treatment, only the child with treatment started at an age of < 18 months gained motor functions.  One of two patients with CHOP INTEND between 10 and 20 at treatment start, gained motor functions (both treated at < 18 months of age).  Five of six patients with CHOP INTEND of > 20 at treatment start gained motor functions, including two patients treated late in the disease.  Correlations:  A correlation was found between the age at treatment initiation and the degree of motor improvement measured by the change in CHOP-INTEND.  The disease duration before treatment initiation (defined as the time between the onset of first symptoms and treatment start) correlated with the change in CHOP-INTEND (r s = −0.85 (95% CI: −0.96 to −0.48; p = 0.002)).  There was no correlation between SMN2 copy number and motor improvement.  Motor Function and Assessments of SMA Type 2 Patients:  Patients: Motor abilities were assessed in 21 SMA type 2 patients.  At Treatment Start: Most patients could hold their head unsupported, sit without support, raise their hands to their mouth in a sitting position, and have a useful function of their hands. No patient was able to walk.  During Nusinersen Treatment: 8 of 21 patients acquired between 1 and 5 motor abilities and one patient lost 2 motor abilities. Five patients gained the motor ability standing with assistance, two achieved walking with assistance and one could walk without assistance.  RULM Scores: At treatment start, patients obtained a median total score of 14 (range 0–24). Five patients reached higher total scores at the last assessment than at the initiation of treatment (an increase of 1–5 points) and 5 patients lost 1–3 points. In two patients, the achieved scores remained unchanged.  HFMSE Scores: At treatment start, patients achieved a median total score of 5.5 (range 0–25). Five patients achieved higher total scores at the last assessment than at the treatment start (an increase of 1–15 points; time of follow-up: median 23 months, range 6.4–29.7; 3 patients gained > 2 points) and 4 patients lost 1–5 points, of those, 2 patients lost > 2 points. Seven patients maintained the same scores.  Correlations: Age at treatment initiation was not correlated with the degree of motor improvement by HFMSE. However, there was some evidence for a correlation between the SMN2 copy number and motor improvement.  Motor Function and Assessments of SMA Type 3 Patients:  Patients: Motor abilities were assessed in 12 SMA type 3 patients.  At Treatment Start: All could sit without support, 8 could stand, and 7 walk.  During Nusinersen Treatment: 3 of 12 patients acquired between 2 and 4 motor abilities and one patient lost a motor ability (crawling on hands and knees). Three patients gained the ability to climb stairs and 2 of those the ability to walk 10 metres unaided.  RULM Scores: At treatment start, patients obtained a median total score of 31 (range 18–37). Two patients reached higher total scores at the last assessment than at the initiation of treatment (an increase of 4–6 points), two patients obtained the same scores and one patient lost 2 points.  6MWT Scores: At treatment start, patients achieved a median score of 387 m (range 169–576 m). All reached longer distances at the last assessments than at the treatment start (an increase of 72–146 m; a median score of 466 m).  HFMSE Scores: At the treatment start, patients achieved a median score of 41 (range 6–62) and 53 (range 6–64) at the last assessment. Eight patients achieved higher total scores at the last assessments (increase of 1–19 points, 6 patients gained > 2 points) than at the treatment start, and three patients had lower total scores (loss of 1–2 points).  Motor Function in Older Patients:  16 patients were at least 14 years old at treatment start (SMA type 1: n = 2; SMA type 2: n = 4; SMA type 3: n = 10;). Their median age at the start of treatment was 19.8 years (range 14.7–44.6) and the median treatment duration was 1.9 years (range 0.5–2.9).  During Nusinersen Treatment: One patient lost a motor ability (raising hand to mouth) while two gained at least one motor ability (rolling onto the side, sitting without support, and climbing stairs).  Reported for 14 patients. Twelve patients achieved at least one motor assessment higher total score at the last assessments (an increase of 1–19 points) than at treatment start. Two patients had a lower score in the HFMSE (decrease of 1–2 points). |
| Van der Heul 2020 | Relationship between functional motor scores and bulbar problems  median CHOP INTEND score at baseline was 19 (range 4–35) in the palliative group and 34 (range 17–46) in the Nusinersen group  infants with CHOP-INTEND scores in the range of 4–35 points all coughed or showed wet breathing during or after drinking  n=2 pre-symptomatic infants with CHOP INTEND scores of 43 and 46 at inclusion in the study showed no symptoms of fatigue or unsafe swallowing  All n=5 treated infants showed a deteriorating swallowing function between the ages of 8 to 12 months  Improvement of motor function did not imply similar gains in bulbar function |
| van der Woude 2024 | The study aimed to explore changes in HFMSE item-scores in children with SMA types 1c-3a treated with nusinersen.  The majority of children, 42 of 46 (91%), showed improvement in at least one item.  28% (13 of 46) showed a score decrease in one or more items.  26% (12 of 46) showed both improvement in some item scores and decreases in others.  Most changes in item scores were from 0 to 1 (105 of 201 changes, 52%).  Decreases usually involved a change from 1 to 0 (10 of 18 changes, 56%).  A score change from 2 to 0 was only found in one patient, in item 13 (props on extended arms), due to hip flexion contractures.  Number of doses of nusinersen and SMN2 copy number did not influence the pattern of score changes.  Item 7 (rolling from prone to supine over left) was most sensitive for score improvement (14 of 46 patients, 30%).  Items 8 and 9 (rolling from supine to prone over both sides) showed improvement in 12 of 46 patients (26%).  Items 21 (3 of 46 patients, 7%) and 22 (4 of 46 patients, 9%) (right and left hip flexion, respectively) were most sensitive for a decline in score.  No changes were observed in item 18, and only one patient showed an improvement in items 16, 19, and 20 (crawling, standing without support, and stepping).  Non-sitters:  Of the 4 children, 2 (50%) improved in one or more HFMSE items.  None showed a decrease in HFMSE items.  Items 1 (sitting) and 3 (bringing one hand to the head) were most sensitive to improvement, with 2 of 4 (50%) non-sitters improving on these items.  Sitters:  Of the 30 children, 28 (93%) improved in one or more HFMSE items.  11 (37%) had a decrease in score in at least one item.  Items 6 and 7 (rolling from prone to supine over right and left side) showed improvement in 10 (33%) and 12 (40%) of the 30 children, respectively.  Items 8 and 9 (rolling from supine to prone over right and left side) showed improvement in 11 of 30 (37%) children.  Most improvement in score was seen in item 2 (long-sitting), item 5 (supine to side-lying), and items 8 and 9.  Items most sensitive to a decline in score were items 21 and 22 (right and left hip flexion), with 3 (10%) and 4 (13%) of the 30 children showing a decline.  Items 4 (two hands to the head) and 14 (lying to sitting) showed a decline in 2 of 30 (6%) children.  Walkers:  All twelve children improved in one or more HFMSE items.  In two (17%), the score decreased in at least one item.  Items 23 and 24 (high-kneeling to right and left half-kneel) showed improvement in 10 (83%) and 8 (75%) of the 12 children, respectively.  Item 30 (ascending stairs with handrail) showed improvement in 7 of 12 (58%) patients.  In the group who did not achieve maximal item score at the start, all (n=10) improved in item 23, and all but one in item 24 (89%).  Items 25 and 26 (high-kneeling to stand leading with right and left leg) showed improvement in 6 of 12 (50%) children.  Item 30 (ascending the stairs with handrail) showed improvement in 7 of 10 (70%) children.  Item 29 (jumping 12 inches forward) showed improvement in 6 of 12 (50%) children. |
| Vazquez-Costa 2022 | At 6 months, an improvement in treated patients was predominant in all scales and tests, while in untreated patients scores usually worsened or remained stable except for the 6MWT.  Nusinersen treatment improved by 2 points (±0.46) in RULM (p < 0.001) according to the model, after adjusting by baseline scores. Differences in other scales were not statistically significant.  Both treated and untreated patients were followed up for a mean of 16 months, although more visits were performed for treated patients (3.21 vs. 2.6).  At the last visit, after adjusting for the baseline values and follow-up time, the effect of treatment was associated with a significant improvement in HFMSE (OR = 1.15, 95% CI 1.04, 1.27, p = 0.006), 6MWT (OR = 1.07, 95% CI 1.06, 1.08, p < 0.001) and EK2 (OR = 0.81, 95% CI 0.71, 0.92, p = 0.001). A non-statistically significant improvement was found in all other scales.  According to the MCID of each scale, a variable percentage of treated (25%–80%) and untreated (0%–57%) patients experienced clinically meaningful improvements at the last visit.  Clinically meaningful improvements were more frequent in treated patients in all scales, although these differences were statistically significant only for RULM, ALSFRS-R, and EK2.  According to the CGI-C and PGI-C, 64.1% and 61.5% of treated patients improved, while 0% and 2.5% of patients respectively deteriorated.  There was a high agreement between CGI-C and PGI-C (unweighted agreement 0.6, weighted agreement 0.8).  A CGI-C of 3 (very much improved) was scored in two SMA type 3a patients.  According to the multivariable model, being a non-sitter (compared with a walker) was associated with less response to treatment, as assessed with the CGI-C, while a longer time of treatment was associated with better response |
| Vidovic 2023 | slight improvement in the ALSFRS-R (median score before treatment initiation: 32.0 points, median score after treatment initiation: 33.0 points; p = 0.015) and the RULM (median score before treatment initiation: 17.0 points, median score after treatment initiation: 20.0 points; p = 0.005); No significant improvement was observed in the HFMSE |
| Vill 2021 | The outcome in treated children with 2 SMN2 copies  N=15 of the 17 children with 2 SMN2 copies were treated with Nusinersen from age 14–39 days  N=8 who were considered to be pre-symptomatic, have remained symptom-free so far and have achieved motor milestones  N=7 already had overt or subtle signs of disease in their first days or weeks of life  In all of them, CHOP INTEND and HINE-2 results improved under therapy  Untreated children with 2 SMN2 copies died  The outcome in treated children with 3 SMN2 copies  N=6 of ten children with 3 SMN2 copies were treated with Nusinersen from age 20–29 days  All remained asymptomatic, as far as the observation period allows to state  N=1 showed a delay in motor milestones  All other treated patients in this cohort remained asymptomatic with normal milestone  The outcome in untreated patients with 3 SMN2 copies  N=4 of the 3 patients could not be treated immediately due to fear of treatment  N=1 was misdiagnosed with 4 SMN2 copies and developed proximal weakness at the age of 8 months  Therapy and outcome in patients with≥4 SMN2 copies and secondary diagnosis for siblings  N=14 patients had 4 SMN2 copies and n=2 patients had 5 SMN2 copies  Untreated children with 3 SMN2 copies developed proximal weakness in their first year |
| Wang 2024 | HFMSE (Hammersmith Functional Motor Scale Expanded):  Walker subgroup:  V4: Mean change +2.32 points (P = 0.004)  V5: Mean change +3.09 points (P = 0.004)  V6: Mean change +4.21 points (P = 0.005)  Sitter subgroup:  V4: Mean change +1.12 points (P = 0.040)  V5: Mean change +2.32 points (P = 0.051)  V6: Mean change +3.21 points (P = 0.067)  Clinically meaningful improvement (≥3 points) in HFMSE:  V4: 32.50% (14/43)  V5: 34.21% (13/38)  V6: 42.42% (14/33)  RULM (Revised Upper Limb Module):  No significant changes between baseline and V4, V5, V6, but a positive trend observed  Clinically meaningful improvement (≥2 points) in RULM:  V4: 11.36% (5/44)  V5: 20.51% (8/39)  V6: 26.47% (9/34)  6MWT (6-Minute Walk Test):  V4: Mean change +18.44 meters (P = 0.002)  V5: Mean change +45.40 meters (P = 0.001)  V6: Mean change +51.70 meters (P = 0.001)  Clinically meaningful improvement (≥30 meters):  V4: 33.33% (9/27)  V5: 66.67% (12/18)  V6: 76.47% (13/17)  CMAP (Compound Muscle Action Potential) Amplitude:  Significant increases observed in:  ADM: Mean change +0.92 mV (P < 0.001)  TRAP: Mean change +0.86 mV (P < 0.001)  APB: Mean change +0.81 mV (P < 0.001)  DEL: Mean change +0.61 mV (P = 0.004)  TA: Mean change +0.44 mV (P = 0.017)  More significant improvement in upper limbs than lower limbs  Patients with baseline RULM ≥ 36 showed significant CMAP improvement in:  ADM: Mean change +1.25 mV (P < 0.001)  TRAP: Mean change +1.23 mV (P < 0.001)  APB: Mean change +1.09 mV (P < 0.001)  DEL: Mean change +1.09 mV (P < 0.001)  TA: Mean change +0.64 mV (P = 0.012)  Subgroup analysis:  TRAP and APB improvements significant in both sitters and walkers  DEL, ADM, and TA improvements significant only in walkers |
| Weaver 2021 | n/a |
| Weber 2024 | GFFS of patients with SMA strongly correlated with motor function and disease severity  All GFFS parameters (II-V4, II-V2, III4, III2) were significantly reduced in patients with SMA compared to age- and sex-matched healthy controls (p < 0.001)  The paediatric patients achieved 9.3 % − 17.4 % of GFFS of the age-matched healthy controls  Adult patients revealed 11.9 % − 15.6 % of GFFS of age-matched-healthy controls  Adult patients revealed higher GFFS than children with SMA  Female patients with SMA showed lower GFFS (59.3 – 81.5 %) compared to male patients, however without statistical significance  GFFS showed moderate to high associations with clinical parameters and motor scores  The strength of II-V4 showed high or very high positive correlation coefficients in children and adults respectively and the strength of III2 correlated moderately to highly with motor function (HFMSE, RULM, MRC sum score) on both hands in adults and children  Children with four SMN2 copies achieved higher strength values (IIV4, II-V2 both hands; III2, III4 left hand) compared to children with less than four SMN2 copies  In children, GFFS did not differ regarding SMA type  In adults, all GFFS parameters, except for III4 of the left hand, revealed significant differences comparing the subgroups SMA type 2 versus type 3  GFFS of patients with SMA increased during Nusinersen treatment  GFFS (II-V2 R, III2 R, III2 L, III4 L, III2LR) increased in the entire cohort on all follow-up time points within the 12-month observation period under Nusinersen treatment  In 18 out of 25 patients of the entire cohort (72 %), GFFS increased between baseline and 12-month follow-up  In children, the strength of III2LR and III4LR increased between baseline and 12-month follow-up  In adults, the strength of III2LR increased between baseline and 12-month follow-up  strength of III2 of the left hand improved significantly. Strength of II-V2  LR and III4 LR as well as III2 of the right hand showed trends to higher forces but were not significantly changed  Motor scores RULM and HFMSE did not significantly change in the study cohort during the 12-month observation period “(HFMSE: Wilcoxon signed-rank test; baseline (17.4 ± 16.5) versus 12 month follow up (16.9 ± 16.0) (p = 0.214); RULM: Wilcoxon signed-rank test; baseline (23.0 ± 10.5) versus 12 month follow up (22.5 ± 11.0) (p = 0.052)” |
| Weststrate 2022 | CHOP INTEND scores were available for 14, 14, 15, and 18 patients at baseline, 6, 12, and 24 months after initiation of Nusinersen respectively. Median CHOP INTEND scores across all available patients’ observations increased from 32 at baseline, to 39, 42, and 42 at 6, 12, and 24 months after initiation of Nusinersen respectively |
| Wohnrade 2023 | n/a |
| Yao 2024 | Score Changes (mean):  Type I:  CHOP-INTEND: 6.7 (6 months), 13.5 (10 months), 14.3 (14 months).  HINE-2: 0.6 (6 months), 1.3 (10 months), 4.0 (14 months).  Type II:  HFMSE: 4.4 (6 months), 4.1 (10 months), 5.1 (14 months).  RULM: 2.4 (6 months), 2.3 (10 months), 4.2 (14 months).  Type III:  HFMSE: 3.9 (6 months), 4.3 (10 months), 4.2 (14 months).  RULM: 2.1 (6 months), 1.5 (10 months), -0.7 (14 months).  Response to Treatment:  Type I: CHOP-INTEND improvement 33.3% (6 months), 100.0% (10 months), 66.7% (14 months).  Type II: HFMSE improvement 62.5% (6 months), 54.6% (10 months), 62.5% (14 months). RULM improvement 52.5% (6 months), 46.9% (10 months), 54.6% (14 months).  Type III: HFMSE improvement 60.7% (6 months), 57.1% (10 months), 61.5% (14 months). RULM improvement 33.3% (6 and 10 months), 45.5% (14 months).  WHO Motor Milestones/Motor Function  Type I: 4 out of 27 patients exhibited 'sitting without support' after nusinersen initiation.  Type II: 1 out of 80 patients achieved 'walking alone' post-nusinersen. |
| Yasar 2024 | Mean CHOP-INTEND Score: 45.2 ± 11.7 (range 25–62) at last follow-up.  Correlation:  Positive correlation between CHOP-INTEND score and number of nusinersen administrations (r = 0.539, p = 0.05).  No significant correlation between CHOP-INTEND score and patient age (r = 0.361, p = 0.076) or time of first nusinersen dose (r = 0.39, p = 0.054). |
| Yu 2024 | n/a |
| Zhang 2024 | n/a |

# Supplementary Table S12: Other outcomes

| **Study characteristics** |  | **Frequency and duration of hospitalisation** | |  |  | |  | **HRQoL (carers and/or patients)** |
| --- | --- | --- | --- | --- | --- | --- | --- | --- |
| **Author name and year** | **Results of bulbar function** | **Frequency of hospitalisation** | **Duration of hospitalisation** | **Results of respiratory function** | **Results of complications of SMA** | **Results of ventilation needed** | **Results of mortality** | **Time points and measures used** |
| Alruthia 2021 | n/a | n/a | n/a | n/a | n/a | n/a | n/a | Arabic version of the EuroQol five-dimensions-3-level (EQ-5D-3L) |
| AlTawari 2024 | n/a | n/a | n/a | n/a | n/a | n/a | n/a | n/a |
| Alves 2021 | n/a | n/a | n/a | n/a | n/a | n/a | n/a | n/a |
| Aragon-Gawinska 2020 | n/a | n/a | n/a | n/a | n/a | n/a | n/a | n/a |
| Arnold 2021 | n/a | n/a | n/a | n/a | n/a | n/a | n/a | n/a |
| Arslan 2023 | n/a | This is reported also as part of the AEs; the time points are in the motor function section. | Only one SMA type 2 patient (16.6%) required hospitalization and intravenous hydration due to severe headache after the second dose. | n/a | n/a | n/a | n/a | n/a |
| Audic 2020 | There was no significant change in the number of patients requiring ventilatory or nutritional support between the start (T0) and after 1 year (Y1) of treatment.     Among the youngest patients (under 2 years old, SMA type 1), there was a non-significant increase in the number requiring nutritional support (three patients at T0 and five at Y1) and in the number requiring ventilatory support (five patients at T0 and eight at Y1) | n/a | n/a | n/a | n/a | There was no significant change in the number of patients requiring ventilatory or nutritional support between the start (T0) and after 1 year (Y1) of treatment.     Among the youngest patients (under 2 years old, SMA type 1), there was a non-significant increase in the number requiring nutritional support (three patients at T0 and five at Y1) and in the number requiring ventilatory support (five patients at T0 and eight at Y1) | n/a | n/a |
| Audic 2023 | Nusinersen failed to prevent gastrostomy for the two patients who started treatment with a nasogastric tube. | n/a | n/a | n/a | n/a | Following 3 years of Nusinersen therapy, non-invasive ventilatory support was required for 15 of 20 (75%) SMA patients with two copies of the SMN2 gene, versus six of 19 (32%) at baseline; and four of 36 (11%) for patients with three copies, versus three of 35 (9%) at baseline.     No patients required tracheostomy. | n/a | n/a |
| Axente 2022 | n/a | n/a | n/a | n/a | n/a | n/a | n/a | n/a |
| Belancic 2023 | Feeding: Worsening occurred in one SMA type 1 patient (who initially had a nasogastric tube) in whom PEG was introduced after 13 doses were received. | n/a | n/a | Respiratory Function: Worsening occurred only in one SMA type 2 patient in whom BiPAP was introduced after five doses of Nusinersen. | n/a | n/a | n/a |  |
| Berti 2022 | N=12 of the 20 infants had normal swallowing and there was no need for tube feeding      at the time treatment started  n=10  of the 12 had consistently normal swallowing with no need for tube feeding on follow-up     n=2 required tube feeding but they regained the ability to eat some food by  mouth   The remaining n=8 infants already had tube feeding inserted at the time treatment started. N=4 of them also had tracheostomy and they showed no changes on the OrSAT Scale. The remaining 4 had tube feeding but no tracheostomy had partial functional improvement.     N=3 of the 20 patients were classified as SMA type 1.1, 12 as type 1.5, and the remaining 5 as 1.9     Results suggest that the treatment with Nusinersen may reduce the expected deterioration      of swallowing abilities in children with SMA 1 | n/a | n/a | n/a | n/a | n/a | n/a | n/a |
| Bjelica 2023 | n/a | n/a | n/a | Pulmonary Function and its Correlation with Motor Function: No significant differences between mean FVC, FEV1, and PEF as well as HFMSE scores at baseline and at different time points during Nusinersen treatment (p > 0.05).   Change in FVC and FEV1 at month 10 positively correlated with the change in HFMSE score at month 10.   Change in FEV1 showed a positive correlation with the change in RULM at month 10.   No significant differences were detected in mean change of FVC, FEV1, and PEF at any timepoint during the follow-up period according to SMA type (2 vs. 3 and 3a vs. 3b), SMN2 copy number (< 4 vs. ≥ 4), baseline HFMSE score (< 35 vs. ≥ 35), previous spinal fusion (spondylosis), use of NIV and use of PEG.   Ambulatory patients showed statistically significant improvement in mean PEF at month 30 vs non-ambulatory patients (+ 0.8 ± 0.5 vs. − 0.0 ± 0.5, p < 0.05). | trend toward improvement of mean PEF at month 30 under Nusinersen treatment in patients without scoliosis as compared to patients with scoliosis (+ 0.8 ± 0.3 vs. + 0.0 ± 0.5, p = 0.053).     in patients with presence of fatigue at baseline showed that statistically significant improvement of mean PEF at month 10 vs patients without fatigue at baseline (+ 0.6 ± 0.9 vs. − 0.4 ± 0.5, p < 0.05).   Regarding fatigue, ambulatory and non-ambulatory patients showed no statistically significant difference (p > 0.05).   Patients with and without fatigue at baseline showed no statistically significant differences in age at therapy start, SMN2 copy number (< 4 vs. ≥ 4), baseline mean HFMSE score, presence of scoliosis, or previous spinal fusion (spondylosis).   FSS measuring fatigue showed an increased score of 0.9 points at month 10, a change of 0 at month 22, and an increase of 3.4 at month 30. | n/a | n/a | Quality of Life (SF-36); Sociodemographic and clinical data were recorded at baseline. Follow-up data were collected at month 10, 22 and 30 of Nusinersen treatment |
| Brakemeier 2021 | Bulbar Function (ALS-FRS-R):   No significant change between T0 and T1 (z = -0.302, p = 0.763, n = 15)   No significant change between T0 and T2 (z = -1.406, p = 0.160, n = 13)     Mean bulbar sub scores: 10.50 (SD = 1.21, n = 16) at T0, 10.50 (SD = 1.32, n = 20) at T1, and 10.94 (SD = 0.97, n = 18) at T2 10.94 (0.97)   From T0 to T1: 5 patients improved, 3 worsened, 7 unchanged   From T0 to T2: 5 patients improved, 2 worsened, 6 unchanged     SSQ Score:   No significant change between T0 and T1 (z = -0.210, p = 0.834, n = 13)   No significant change between T0 and T2 (z = -0.392, p = 0.695, n = 12)     Mean SSQ scores: 23.59 (SD = 18.85, n = 18) at T0, 24.12 (SD = 19.32, n = 15) at T1, and 19.95 (SD = 17.86, n = 13) at T2     From T0 to T1: 8 patients improved, 5 worsened   From T0 to T2: 6 patients improved, 6 worsened     Additional Analyses:   No significant changes in bulbar function for type 2 and type 3 SMA patients separately   No new bulbar impairments developed over 14 months for 23 SMA patients with no documented bulbar dysfunction at treatment initiation   Significant positive correlation between the baseline bulbar sub score of the ALSFRS-R and baseline limb motor function measured by the RULM (r = 0.434, p = 0.043, n = 16)   No significant correlation between the baseline SSQ score and baseline bulbar sub score of the ALSFRS-R (r = -0.304, p = 0.336, n = 12)   Strong positive correlation between baseline HFMSE and baseline RULM scores (r = 0.925; p < 0.001, n = 22)   No associations of the bulbar or extremity motor scores with age and SMN2 copy number | n/a | n/a | n/a | n/a | n/a | n/a | n/a |
| Brakemeier 2024 | Bulbar Function (ALS-FRS-R):     No significant change between T0 and T1 (z = -0.302, p = 0.763, n = 15)     No significant change between T0 and T2 (z = -1.406, p = 0.160, n = 13)     Mean bulbar sub scores: 10.50 (SD = 1.21, n = 16) at T0, 10.50 (SD = 1.32, n = 20) at T1, and 10.94 (SD = 0.97, n = 18) at T2 10.94 (0.97)     From T0 to T1: 5 patients improved, 3 worsened, 7 unchanged     From T0 to T2: 5 patients improved, 2 worsened, 6 unchanged     SSQ Score:     No significant change between T0 and T1 (z = -0.210, p = 0.834, n = 13)     No significant change between T0 and T2 (z = -0.392, p = 0.695, n = 12)     Mean SSQ scores: 23.59 (SD = 18.85, n = 18) at T0, 24.12 (SD = 19.32, n = 15) at T1, and 19.95 (SD = 17.86, n = 13) at T2     From T0 to T1: 8 patients improved, 5 worsened     From T0 to T2: 6 patients improved, 6 worsened     Additional Analyses:     No significant changes in bulbar function for type 2 and type 3 SMA patients separately     No new bulbar impairments developed over 14 months for 23 SMA patients with no documented bulbar dysfunction at treatment initiation     Significant positive correlation between the baseline bulbar sub score of the ALSFRS-R and baseline limb motor function measured by the RULM (r = 0.434, p = 0.043, n = 16)     No significant correlation between the baseline SSQ score and baseline bulbar sub score of the ALSFRS-R (r = -0.304, p = 0.336, n = 12)     Strong positive correlation between baseline HFMSE and baseline RULM scores (r = 0.925; p < 0.001, n = 22)     No associations of the bulbar or extremity motor scores with age and SMN2 copy number | n/a | n/a | n/a | n/a | n/a | n/a | n/a |
| Cavaloiu 2024 | n/a | n/a | n/a | n/a | n/a | n/a | n/a | n/a |
| Cavaloiu 2024b | n/a | n/a | n/a | n/a | n/a | n/a | n/a | SF-36 Questionnaire Scores Stratified by SMA Type  Physical Functioning:  Significant difference (p = 0.0002).  Higher in SMA Type 3.  Role Functioning (Physical and Emotional):  No significant differences.  Energy, Fatigue, Emotional Well-being:  No significant differences.  Social Functioning and Pain:  No significant differences.  General Health:  SMA Type 1: Highest average score (60.41 ± 16.79).  SMA Type 2: 48.73 ± 15.68.  SMA Type 3: 59.82 ± 15.08.  SF-36 Questionnaire Scores Stratified by Environment  Urban vs. Rural:  Better health outcomes in urban areas.  Significant difference in general health (p = 0.014).  Urban residents: Higher median scores, narrower IQR.  Rural residents: Lower median scores, more variability (p = 0.016).  SF-36 Questionnaire Scores Correlated with Age  General Health Scores:  Younger individuals report higher scores.  Highest density of scores in 20 to 40 age range (60 to 80).  Decline in health scores with age.  Age Groups:  Under 14: Proxy responses.  Over 14: Self-reported.  Younger group: Better role functioning, emotional well-being (p = 0.0217), and general health (p = 0.0072).  Physical and social functioning: Consistent across age groups.  Significant difference in general health scores between age groups (p = 0.0375). |
| Chacko 2022 | n/a | n/a | n/a | Over the course of the study, four children with SMA type 1 had respiratory-related hospitalisations     There were no respiratory-related admissions for children with SMA type 2 or type 3     One participant with SMA type 2 and four participants with SMA type 1 started NIV during the study period     Rate of Lung Function Decline:     The combined rate of annual decline in FVC z-score in the 2 years prior to the start of Nusinersen was -0.58 compared with -0.25 in the year post-initiation     Lung Function:     Only children with SMA types 2 and 3 performed lung function     No clinically important changes in lung function were observed over the study period     Polysomnography:     There was agreement in the scoring between the two sleep physicians     Total and REM stage AHI and central apnoea index improved at both a clinically and statistically significant level in SMA type 1     All SMA type 1 and 75% of SMA types 2 and 3 showed an improvement in peripheral muscle function greater than the predefined response threshold | n/a | n/a | n/a | n/a |
| Chen 2024 | n/a | n/a | n/a | n/a | n/a | n/a | n/a | n/a |
| CHERISH/ISIS 396443-CS4 (NCT02292537) (Mercuri 2018) | n/a | n/a | n/a | n/a | n/a | n/a | n/a | n/a |
| Chiriboga 2016 | n/a | n/a | n/a | n/a | n/a | n/a | n/a | Paediatric Quality of Life Inventory (Generic Core Scales and Neuromuscular Module)     Timing of assessment     CS1: Days 29 and 85     CS10: Day 169 |
| Chiriboga 2024 | n/a | n/a | n/a | Sniff Nasal Inspiratory Pressure Test: Mean change of 0.14% (95% CI –2.64 to 2.92, n = 113) from baseline to month 24. | n/a | n/a | n/a | n/a |
| Cho 2023 |  |  |  |  |  |  |  |  |
| Colot 2024 | Intraoral Pressure Measurements: Mean oral normalized pressure index lower in SMA patients compared to healthy controls (all sites p < 0.001). Pressure evolution over 1 year in SMA patients for all three oral sites did not show significant differences. Mean oral Pmax significantly higher for healthy children compared with SMA children in each anatomical region (all p-values < 0.003).  SFAQ Scores: Mean SFAQ scores higher in SMA patients compared to healthy controls (p < 0.001). SFAQ score significantly higher in the SMA group with more variability observed between the scores (median = 9.0; IQR = 21.00) compared with the healthy group (median = 1.5, IQR = 2.0). Notably, this difference was enhanced in SMA type 1 children who had the highest scores. SFAQ scores correlated negatively with oral pressures at all three sites in SMA patients.  Analysis in SMA Patients: Majority of SMA type 2 (78%) and all SMA type 3 patients had mild swallowing impairment (total SFAQ score ≤20). SMA type 1 group had moderate to severe swallowing impairment. Highest SFAQ scores found among SMA type 1 patients (median = 33.5, IQR = 26.5). Non-sitter group had the highest median SFAQ score (28.5, IQR = 11.3) compared to sitter (median = 9.0, IQR = 15.3) and walker (median = 6.0, IQR = 3.9) groups. Patients treated with nusinersen presented a lower oral pressure index compared with those treated with risdiplam. | n/a | n/a | n/a | n/a | n/a | n/a | n/a |
| Coratti 2021 | n/a | n/a | n/a | n/a | n/a | n/a | n/a | n/a |
| Coratti 2024 | n/a | n/a | n/a | n/a | n/a | n/a | n/a | n/a |
| Cornell 2024 | n/a | n/a | n/a | n/a | n/a | n/a | n/a | n/a |
| CS2/CS12NCT01703988   (see also NCT02052791 an extension study)  Darras 2019 CS12 & CS2 | n/a | n/a | n/a | n/a | n/a | n/a | n/a | n/a |
| De Wel 2021 | n/a | n/a | n/a | FVC values were higher in patients who retained ambulation (p < 0.01), patients with four SMN2 copies (p=0.03, but not those with three copies), and patients with SMA types 3 and 4 (p<0.01 for both) | n/a | n/a | n/a | SF-36 quality of life questionnaire      “The SF-36 quality of life questionnaire comparison between months 6 and 14 (no baseline data available) |
| Duan 2022 | More patients in the medication group used nasal or gastric tube feeding for nutrition support, and more patients used ventilators. | n/a | n/a | n/a | n/a | More patients in the medication group used nasal or gastric tube feeding for nutrition support, and more patients used ventilators. | n/a | PedsQL, Peds NMM, and PedsQL GCS are reported    changes in health conditions in the past six months for two treatment groups” have been overviewed.   “The first measurement was considered as the baseline. We concentrated our further study on the third measurement in order to reflect the effectiveness of treatment more properly.” |
| Duong 2021 | n/a | n/a | n/a | SMAFRS (mean slope = 1.44 points/year, 95% CI 0.04–2.83, p = 0.04)    Overall, respiratory measures exhibited a mean rate of change indicating potential improvement after Nusinersen treatment, most prominently with respiratory muscles of MEP (mean slope = 6.38 cm H2O/year, 95% CI 2.52–10.25) and to a lesser extent MIP (mean slope = −5.50 cm H2O per year, 95% CI −11.47 to 0.47) | n/a | n/a | n/a | n/a |
| Edel 2021 | n/a | n/a | n/a | Changes in GSR scores over the course of treatment     The GSR score increased from baseline (month 0) to end of loading doses of Nusinersen (month 2) for the majority of patients, except 3 patients     No clear difference between subtypes of SMA1 at Month 2 (p=0.17)     By month 18 (dose 8), SMA Type 1B had a higher median GSR Score of 21 compared to SMA Type 1C, which was 12, the whole cohort appeared to stabilise in terms of the GSR Score     By the end of the loading dose, neither SMA 1B nor 1C patients demonstrated any improvement in GSR score, 46% (6/13) of SMA 1B patients remained unchanged in their respiratory status while 86% (6/7) of SMA 1C patients showed variable degree of worsening in their GSR scores     Between the end of the loading dose and post 5th dose, all of the SMA 1C patients stabilised with no changes in their GSR scores while 61% (8/13) of SMA 1B patients either stabilised or showed improvement in GSR scores     By dose 8 all SMA1B patients (n=6) showed consistent improvement in the GSR score compared to their GSR scores at the end of loading doses     The change in SMA1C patients (n=5) was more variable | n/a | n/a | n/a | n/a |
| EMBRACE (NCT02462759) (Acsadi 2021) | n/a | n/a | n/a | n/a | n/a | (Results taken from Acsadi et al. 2021). ‘The mean percentage of time on ventilator support was lower for individuals with later-onset SMA (mean 0% for those treated with Nusinersen in parts 1 and 2 [n = 5]; 11.1% for the sham procedure in part 1 [n = 3]; 8.5% for Nusinersen initiated in part 2 [n = 3]) compared with infantile-onset SMA (mean 17.6% for those treated with Nusinersen in parts 1 and 2 [n = 9]; 43.8% for sham procedure in part 1 [n = 4]; 48.6% for Nusinersen initiated in part 2 [n = 3]).’ | n/a | n/a |
| ENDEAR/ISIS 396443-CS3B (NCT02193074 (Finkel 2017) | n/a | n/a | n/a | n/a | n/a | n/a | No death: 67/80 (84) vs 25/41 (61); HR 0.37     No use of permanent assisted ventilation: 62/80 (78) vs 28/41(68); HR 0.66   No death or use of permanent assisted ventilation amongst those with a disease duration of ≤13.1 weeks at screening: 30/39 (77) vs 7/21 (33); HR 0.24     No death or use of permanent assisted ventilation amongst those with disease duration >13.1 weeks at screening: 19/41 (46) vs 6/20 (30); HR 0.84 | n/a |
| European registries (NICE 2020) | n/a | n/a | n/a | n/a | n/a | n/a | n/a | n/a |
| Fainmesser 2022 | n/a | n/a | n/a | The baseline median FEV1 was 87% (range 18–119%), with no significant change at follow-up visits.     The ALSFRS-R showed an increase of one point in patients with SMA2 at 6 months of treatment, but not thereafter. | n/a | n/a | n/a | n/a |
| Finkel 2021 | n/a | n/a | n/a | n/a | n/a | n/a | At study closure, 15 (75%) of 20 participants who received Nusinersen in the safety population were alive.     11 (55%) of 20 participants were alive without the need for permanent ventilation; thus, a median time to death or permanent ventilation was not reached.     None of these 11 participants received a tracheostomy.     All 13 participants in cohort 2 with two SMN2 copies required some ventilators use during the study; two were alive and met the endpoint of permanent ventilation. | n/a |
| FIREFISH (NCT02913482) (Masson 2022) | At baseline, none of the infants were able to sit without support. 5 out of 21 infants (24%) were receiving respiratory support, with 4 of these receiving this support prophylactically. One infant was fed by tube at baseline due to inadequate weight gain. The ability to swallow had not been assessed after enrolment in the study. However, 39 infants (95%) were able to swallow.    Over the course of the study, there was an improvement in the infant's ability to feed orally. At month 12, 34 infants (82.9%) were able to feed orally. This included 28 infants (68.3%) who were feeding orally, 4 infants (9.8%) who were feeding via a tube, and 6 infants (14.6%) who were feeding via a combination of oral and tube feeding. No infant lost the ability to swallow over the 12-month period.    By month 24, the number of infants able to feed orally increased to 35 (85%), showing a slight improvement from month 12. However, it's important to note that no infants could sit without support or stand independently at any point during the study. |  | • Overall, there were 72 hospitalisations by Month 24 (defined as hospital admissions observed by Month 24 that spanned at least 2 days).  • The rate of hospitalisations per PY was 0.94 (90% CI 0.76–1.14).  • Between Months 12 and 24, there were 22 hospitalisations.  • At Month 24, infants who were hospitalised were admitted for a median of 15 nights (IQR: 8.8–35.0), including planned/elective hospital admissions.  • 14 of 41 infants did not require any overnight hospitalisation by Month 24.  Web STA:  • Patients who were hospitalised were admitted for a median of 17 nights (range: 2.0–151.0) in total. These numbers also include planned / elective hospital admissions. | n/a | n/a | At month 12, a total of 35 out of 41 infants (85%) remained event-free, exceeding the performance criterion of 42%. At this stage, 70.7% of the infants did not require any form of ventilatory support. However, a proportion of patients were receiving ventilation support, with 14.3% receiving bilevel positive airway pressure (BiPAP) for fewer than 16 hours per day and 19.0% receiving ventilation prophylactically. Additionally, 10 infants (24%) were without invasive or non-invasive respiratory support.    By month 24, the event-free survival rate slightly decreased to 83%, with 34 of 41 infants remaining event-free. One additional infant required permanent ventilation between month 12 and the current clinical cutoff date. The percentage of infants without any form of ventilatory support remained the same at 70.7%. The number of infants without invasive or non-invasive respiratory support decreased to 8 (20%).    Throughout the study, 29 infants (71%) received no pulmonary care, defined as no ventilatory support or airway clearance. Of the 12 infants receiving pulmonary care, 11 received this prophylactically. | • Following 24 months of Risdiplam treatment, 38 of 41 infants (93% [90% CI 82–97]) were alive (Masson).  • In the pooled population (part 1 high-dose cohort, and part 2), 605 AEs were reported up to the CCOD (12 November 2020). Up to this CCOD, a total of six deaths occurred; all had occurred before the CCOD of the primary analysis (14 November 2019) (Post-hoc analyses).  • Following 24 months of Risdiplam treatment, 38 of 41 infants (93%) were alive (Masson).  • In the pooled population (part 1 high-dose cohort, and part 2), 605 AEs were reported up to the CCOD (12 November 2020). Up to this CCOD, a total of six deaths occurred; all had occurred prior to the CCOD of the primary analysis (14 November 2019) (Post-hoc analyses).  • The proportion of infants alive at Month 12 was 92.7% (38/41; 90% CI: 82.2%, 97.1%). This proportion is significantly higher than the pre-defined performance criterion of 60% based on natural history data (p<0.0005). The median time to death was not estimable as few patients had an event (Web STA). | Masson Appendix:  • The ITQOL-SF47 is a parent/caregiver measure used to assess the health status and health-related quality of life (HRQoL) of children/infants between 2 months and 5 years of age, and HRQoL of the parent/caregiver.  • Items are scored using a Likert type scale with five levels (except for parent time limitations which has four levels).  Parent-/caregiver-reported outcomes:  • For three domains of the ITQOL-SF47 (parent impact – emotional health, temperament and mood, growth and development), an improvement from baseline in the median score was observed at Month 24.  • For two domains and two single items (bodily pain/discomfort, parent impact – time limitation, family cohesion, overall health), there was no change from baseline in the median score at Month 24.  • A decline from baseline in the median score was observed for two domains (physical abilities and general health perceptions).  • For one domain (combined behaviour scale) and two single items (change in health item and global behaviour item), the assessment was not carried out at baseline, as individuals must be ≥12 months of age at the time of assessment for these domains/items to be completed. Thus, change from baseline to Month 24 could not be calculated for these items. |
| Freigang 2021 | n/a | n/a | n/a | n/a | n/a | n/a | n/a | n/a |
| Freigang 2021b | n/a | n/a | n/a | n/a | n/a | n/a | n/a | n/a |
| Freigang 2023 | n/a | n/a | n/a | Diaphragm Thickness and Excursion of Patients with SMA:   At baseline, DUI measurements were performed in 23 patients.   in 1 patient, left-sided parameters were not measurable due to severe scoliosis. Most values were within the reference range some values showed an increased thickness of the diaphragm, whereas none of the values were below the lower limit of normal diaphragm thickness.   DTF and excursion were reduced in approximately 25% of the measurements and the excursion increased only in single cases.   Right-sided Diaphragm Excursion Correlated with Disease Severity: Although there was a difference between SMA subtypes in spirometry parameters, none of the DUI parameters distinguished patients with SMA types 2 and 3. DUI parameters did not correlate with surrogate markers for disease severity with the exception of the right-sided diaphragm excursion, which correlated moderately with SMN2 gene copy number, PEF, and ALSFRS-R score.   Diaphragmatic Motility Increased during 26 Months of Nusinersen Treatment: diaphragm thickness in the end-expiratory position did not change during 26 months of Nusinersen treatment, it did change in end-inspiratory position on the left. During 26 months of Nusinersen treatment, diaphragm excursion and DTF improved and FVC remained stable.  All dynamic parameters exceeded the lower limit of the reference range within 38 months.  1 exhibited a paradoxical end-expiratory diaphragm thickening before the start of Nusinersen treatment which normalized within the observation period. | n/a | n/a | n/a | n/a |
| Gaboli 2025 | Type 1c patients retained the ability to feed orally, while type 1b patients gradually reduced their capability. All type 1b patients transitioned to exclusive feeding support by the end of the study. VFSS showed severe dysphagia in type 1b patients and mild/moderate dysphagia in type 1c patients. | n/a | n/a | Significant improvement in FVC and FEV1 z-scores in type 2 patients at 6, 12, and 24 months. No significant changes in type 3 patients. | Increase in scoliosis observed in type 1b and type 2 patients. One type 1c patient underwent surgery. | Type 1b and 1c patients had a higher rate of MV use compared to type 2/3 patients. Age at first nusinersen administration was significant, indicating a higher likelihood of needing MV with older age at treatment start. | n/a | n/a |
| Gaffar 2022 | low dose 3/4 (75) 15/17 (88) 18/21 (86) | n/a | n/a | n/a | n/a | Did not require respiratory support n (%) 1/4 (25) 3/17 (18) 4/21 (19) | For the sake of comparison, event-free survival at 12 months in the natural history cohort can be roughly approximated at 38%, by taking the average of the given median of 10.5 months (50% event-free) with an interquartile range of 8.1–13.6 months.     After 12 months of treatment, 19 infants did not have a clinical event of death or need for permanent ventilation.     At the study cut-off date, only 18 infants were available for follow-up, as one died in the interim.     Overall, of the 21 total infants enrolled, 86% (18 of 21) were followed up: 75% (3 of 4) of the low-dose cohort and 88% (15 of 17) of the high-dose cohort.     Three infants passed away from SMA-related complications. | n/a |
| García de la Banda 2020 | n/a | n/a | n/a | One patient with SMA 2 refused the respiratory muscle tests and the recording quality was too low in two patients (1 with SMA 1c and 1 with SMA 2). Therefore, data from 12 patients with SMA 2 treated with Nusinersen were retained for analysis.  1. Inspiratory muscle strength: The global inspiratory muscle strength assessed on sniff Pes (both absolute values and % predicted) and MIP (absolute values) in the patients with SMA 2 treated with Nusinersen were significantly better than the historical controls.  2. MIP % predicted: There was a trend for a better MIP % predicted.  3. FVC (% predicted): FVC (% predicted), as an indirect marker of inspiratory and expiratory muscle strength, was also significantly better.  4. Diaphragm and Expiratory muscle strength: The specific effect on the diaphragm was less pronounced with a nonsignificant improvement in sniff Pdi, as well as for expiratory muscle strength (MEP and Pgas cough).  5. Patient with SMA 1c (#3): The patient with SMA 1c (#3) treated with Nusinersen had lower values of lung function (FVC 43%) and respiratory muscle strength (SNIP 18 cmH2O, sniff Pes 31, sniff Pdi 67, and Pgas cough 26) than the SMA 2 treated patients.    Correlations between motor and respiratory muscles performance     “The strength of the global inspiratory muscles of SMA 2 treated with Nusinersen, assessed on maximal static inspiratory pressure, forced vital capacity, and oesophageal pressure during a maximal sniff was significantly better compared with historical controls (p < .05)”     A significant improvement in MFM and HINE‐2 was observed in the patients with 16 SMA treated with Nusinersen after 14 months as compared with baseline         Overall in children with SMA Type 2, the global inspiratory muscle performance was significantly better after six injections of Nusinersen as compared with age‐matched SMA Type 2 historical controls | n/a | n/a | n/a | n/a |
| Goedeker 2021 | n/a | n/a | n/a | n/a | n/a | n/a | n/a | n/a |
| Gonski 2023 | n/a | Frequency of Respiratory Hospitalisations and Length of Stay:   No. of unplanned respiratory admissions including days of admission and PICU admissions were collected in the 2-year periods before and after initiation of Nusinersen for SMA Type 2 and 3 cohort only.   Pre-initiation hospitalisation data was not collected in the Type 1 group as they were initiated on Nusinersen at < 12 months of age. | Frequency of Respiratory Hospitalisations and Length of Stay:   A total of 13 unplanned respiratory admissions (Type 2 n = 6, Type 3 n = 2) in the 2-year period before and 15 admissions (Type 2 n = 4, Type 2 n = 2) in the 2-year period after starting Nusinersen in the SMA 2 and 3 cohort.   The mean length of stay of admission for unplanned respiratory admissions in this group was 2.88 (SD 6.34) days pre-nusinersen and 11.23 (SD 37.28) days post (paired t-test; p-value 0.93).   No. of ICU admissions for unplanned respiratory admissions pre and post-nusinersen were 4 and 5 (paired t-test, p = 0.74) with the duration of ICU admissions being 34 days pre-Nusinersen and 23 days post-Nusinersen ( p = 0.64). | Spirometry:   22 children performed acceptable and reproducible spirometry testing prior to starting Nusinersen (Mean ±SD age 10.01 years (±3.3)) and 26 patients in the 2-year period after starting Nusinersen (Mean age 12.21 yr. (±3.86)).   No SMA type 1 patient had spirometry data due to their age being < 5 years old during the study period (unable to reliably perform acceptable and reproducible spirometry).   22 patients had spirometry measured both before and after starting Nusinersen (SMA2 n = 13, SMA 3 n = 8).   There was a significant increase in the paired individual data for mean absolute FVC (L) between pre and post-nusinersen with the mean FVC increasing from 1.37 L to 1.55 L ( p = 0.01).   However, the mean FVC%P trended lower post-treatment (p = 0.07).   16 participants had >2 spirometry readings pre- and post-starting Nusinersen. A non-significant reduction in annual decline in FVC%P (change in slope 3.9) and FVC Z-score (change in slope 0.4) (p = 0.21, p = 0.11).   Table 5 FVC (L) mean 1.16 (pre) – 1.37 (post)   FVC% Predicted mean 65.52% (pre) - 59.24% (post)   FVC Z score mean -3.09 (pre) -3.66 (post)   FEV1 (L) mean 1.03 (pre) - -1.23 (post)   FEV1% Predicted mean 65.05% (pre) - 60.48 (Post)   FEV1 z score means –2.78 (pre) - -3.13 (post) | n/a | Non-invasive Ventilation:   22 (44%) (SMA1 = 3, SMA2 = 14, SMA3 = 4) children were on nocturnal NIV (< 16 h / day) prior to starting Nusinersen, and of these 6 (SMA2 = 5, SMA3 = 1) ceased NIV during the 2-year period post Nusinersen  1 (SMA2) ceased NIV against medical advice.   No children with SMA 1 were weaned from NIV during the study period.   3 children (SMA 1 = 2, SMA 2 = 1) commenced on NIV in the 2-year period after starting Nusinersen and 17 (35%) (SMA1 = 5, SMA2 = 9, SMA3 = 3) children were prescribed nocturnal NIV at the end of the study period.   Those who remained on NIV had no significant change in pressures after starting Nusinersen. | n/a | n/a |
| Govoni 2024 | n/a | n/a | n/a | n/a | n/a | n/a | n/a | n/a |
| Gunther 2024 | n/a | n/a | n/a | n/a | n/a | n/a | n/a | n/a |
| Hagenacker 2020 | n/a | n/a | n/a | n/a | n/a | n/a | n/a | n/a |
| Hahn 2022 | n/a | n/a | n/a | n/a | n/a | n/a | n/a | n/a |
| Huang 2024 | n/a | n/a | n/a | n/a | n/a | n/a | n/a | HRQoL of Caregivers: Significant improvements in all fields of PedsQL FIM scores over time (p < 0.001). Significant correlation between children's HRQoL and caregiver's HRQoL  HRQoL of SMA Patients: Significant improvements observed across all domains of PedsQL NMM, including total, neuromuscular disease, communication, and family resources (p < 0.001).  Independence Assessment: Significant increase in SMAIS-ULM scores for all patients after 18 months of nusinersen treatment (p < 0.001).  Subgroup Analysis: Patients with shorter disease duration scored higher in the domain of neuromuscular disease (p = 0.003). No significant differences in communication or family resources domains between groups. Higher independence scores after treatment in patients with shorter disease duration (p = 0.044). |
| Ip 2024 | n/a | n/a | n/a | n/a | scoliosis worsening is reported as part of the AEs | n/a | n/a | n/a |
| JEWELFISH (NCT03032172) 2023 (Chiriboga 2023) | n/a | n/a | n/a | n/a | n/a | n/a | n/a | n/a |
| Jiang 2024 | n/a | n/a | n/a | n/a | n/a | One participant required ventilation 24 h/day at two study visits (baseline and Day 63) prior to study withdrawal. Another participant reported BiPAP use of 8 h/day at one visit (Day 183). No other participants reported ventilator use in the 7 days preceding their study visits. No participants were placed on permanent ventilation or underwent tracheostomy during the study. | n/a | n/a |
| Jiang 2024b | n/a | n/a | n/a | n/a | n/a | n/a | n/a | n/a |
| Kariyawasam 2021 | n/a | n/a | n/a | n/a | n/a | n/a | n/a | n/a |
| Kokaliaris 2024 | n/a | n/a | n/a | n/a | n/a | n/a | Risdiplam showed a 78% reduction in the rate of death (HR 0.22), 81% reduction in the rate of death or permanent ventilation (HR 0.19) compared to nusinersen. | n/a |
| Kolbel 2022 | n/a | n/a | n/a | n/a | n/a | n/a | n/a | Two questionnaires were used: the “Family Burden Questionnaire” (FaBel) and the “Quality-of-Life Inventory for Parents of Chronically Ill Children” (ULQIE) |
| Konersman 2021 | n/a | n/a | n/a | n/a | n/a | n/a | n/a | n/a |
| Kotulska 2022 | n/a | n/a | n/a | n/a | n/a | n/a | n/a | n/a |
| Krosmark 2023 | n/a | n/a | n/a | n/a | Spine deformities:  At the start, 19/20 performed a spinal X-ray   5 previous spinal surgery due to severe scoliosis (3 SMA type 2, 2bSMA type 3)   6 scoliosis with a Cobb’s angle >20 degrees, (21 to 94 degrees)   At follow-up:  18 performed a spinal X-ray. Seven = scoliosis >20 degrees (27 to 94 degrees), the scoliosis had deteriorated with increased Cobb’s angle 7 to 45 degrees during the study  1 SMA type 2 patients had spinal surgery during the study period   6 had still no scoliosis, the majority with SMA type 3 (n = 5)   Hip Deformities:  At the start, 19/20 performed a hip X-ray, 1 (SMA type 2) had bilateral total hip luxation   7 (SMA type 3) had a bilateral normal hip status   7 SMA type 2 patients and 1  SMA type 3 had bilateral subluxation (Reimer’s index 40–85 degrees)   2 (SMA type 2) had a normal hip status on one side and a subluxation on the other side   1(SMA type 2) had a normal hip status on the left side, while the right side was dislocated. This status stayed unchanged for the patient throughout the study   At follow-up, 18/20 performed a hip X-ray. In the group with normal hip status at baseline (all SMA type 3), 1 patient had developed a bilateral subluxation at follow-up   1 (SMA type 2) with subluxated hips at baseline developed bilateral total dislocation during the study period   No hip extension contracture was measured in 6 of 8 SMA type 3 patients. Range of motion 0 to 20 degrees (median 10 degrees), while all 11 SMA type 2 patients had hip extension contractures with a range of motion from −5 to −60 degrees (median−27 degrees)   2 SMA type 3 patients had hip extension contractures of −10 and −25 degrees     7 of 8 SMA type 3 had normal hip status on X-ray.  In all 11 SMA type 2 patients, 1 or 2 subluxated or luxated hips at baseline deteriorated in one patient during the study period   These findings corresponded well with decreased hip joint movement in most SMA type 2 patients, who likewise were non-ambulant.  SMA type 3 patients, with the highest HFMSE scores, were also the patients with normal hips and normal - less severe scoliosis   1 SMA type 3 (non-ambulant) patient with a low HFMSE score was the very same patient with subluxated hips and prior spine surgery | n/a | n/a | n/a |
| Krupa 2023 | n/a | n/a | n/a | n/a | n/a | Mechanical Ventilation: 20 patients from the 2014 cohort were administered any duration/day of mechanical ventilation compared to 23 from the 2019 cohort. | Up to the data lock date set on 31 May 2022, 8 deaths in the 2014 cohort and two in the 2019 cohort.  Survival Rate:  72% of children born in 2014 of the cohort survived past their fourth birthday. No events were recorded for the children between the ages of four and eight. In the younger cohort, 97% reached the 3-year mark.   Median Survival: For patients in the 2014 cohort, median survival was 823 days, or 2 years and 3 months.  2019 cohort, median survival was not observed as of the cutoff date of day 1382 since birth. |  |
| Kwon 2022 | n/a | n/a | n/a | n/a | n/a | n/a | n/a | n/a |
| Landfeldt 2023 | n/a | n/a | n/a | n/a | n/a | n/a | n/a | Data collection: September 2020 - April 2022. Caregivers’ Basic Demographic Characteristics, Health-Related Quality of Life (HRQoL), Paediatric Quality of Life Inventory 4.0 Generic Core Scales (PedsQL 4.0 GCS), Visual Analogue Scale (VAS), Parental Involvement. 6, 12, and 18 months of follow-up |
| Lavie 2021 | Feeding Method and Related Complications: The feeding methods varied before treatment initiation: 7 patients received oral feeding, 12 patients were fed by a per gastrostomy (PG), and 1 patient was fed by a nasogastric tube (NGT). After completing 3 years of follow‐up, 15 out of 17 patients were fed PG, 1 was fed orally, and 1 by an NGT because of the family’s refusal of gastrostomy. The cohort’s mean BMI z‐score after 3 years of treatment was −0.79 ± 2.17. | n/a | n/a | At treatment initiation, 16 of the 20 study patients were using respiratory support. After 3 years of treatment, all 17 surviving patients were ventilated. Eleven patients had daily routine MIE before treatment initiation, and all 17 patients were routinely using daily MIE after 3 years | Facial Weakness, Hypoplasia, or Deformity: Four patients had facial deformities before treatment initiation. After 3 years of treatment, 15 of the 17 surviving patients displayed facial changes which included mandibular retraction, nasal flattening, midface hypoplasia, facial asymmetry, open mouth, and extreme facial weakness.    Scoliosis: Five patients had already been diagnosed with scoliosis at treatment initiation, two of whom were invasively ventilated and three were NIV patients. At the 3‐year follow‐up, 13 of 17 patients were diagnosed with scoliosis. Four of those 13 patients had already undergone scoliosis corrective surgery that included magnetic‐controlled growing rods, while an additional three are planned for surgery. All four patients who underwent corrective surgery recovered uneventfully. | n/a | n/a | n/a |
| Lee 2022 | n/a | n/a | n/a | n/a | n/a | n/a | n/a | n/a |
| Lee 2023 | n/a | n/a | n/a | n/a | n/a | n/a | n/a | HRQoL was assessed:  - Paediatric Quality of Life Inventory 4.0 Generic Core Scale (PedsQL GCS)  - Paediatric Quality of Life Inventory 3.0 Neuromuscular Module (PedsQL NMM).    The changes in the activities of daily living and caregiver burdens were evaluated using  - Paediatric Evaluation of Disability Inventory Computer Adaptive Test (PEDI-CAT)  - Assessment of Caregiver Experience with Neuromuscular Disease (ACEND) |
| Lemska 2024 | n/a | n/a | n/a | n/a | n/a | n/a | n/a | n/a |
| Li 2024 | n/a | n/a | n/a | n/a | n/a | n/a | n/a | n/a |
| Li 2024a | n/a | n/a | n/a | n/a | n/a | n/a | n/a | n/a |
| Li 2024b | n/a | n/a | n/a | n/a | n/a | n/a | n/a | n/a |
| Lillien 2024 | n/a | n/a | n/a | n/a | n/a | n/a | n/a | Quality of Life (QoL):  No statistical difference in general impression of change in QoL across groups  NR Group: 78% reported improvement in QoL (PGIC scores >4)  RCS Group: 12% perceived no change in QoL  No statistical difference in patient perception of treatment benefits in 17 out of 22 domains across patient groups. Significant differences in PGIC scores for balance while sitting (RCS > NR, p adjusted = 0.04), function involving shoulder muscles (RCS > NR, p adjusted = 0.04), and loudness of voice (RCS > NR, p adjusted = 0.04). Patients classified as RCS improved not only on functional scales but also according to their perception. |
| Lusakowska 2023 | n/a | n/a | n/a | n/a | n/a | n/a | n/a |  |
| Maggi 2020 | n/a | n/a | n/a | Pulmonary function tests      Only a nominally significant FVC% increase at T14 (median +7%; p=0.031) in the SMA3 ‘walker’ subgroup     To a lesser degree, FEV1% improved between baseline and T14 in the whole SMA3 population (+3%; p=0.0499). | n/a | n/a | n/a | n/a |
| Mendonca 2021 | n/a | n/a | n/a | Among the 21 patients, seven (33.3 %) were using NIV at baseline. At the last evaluation, five of these children had the daily time of ventilatory support reduced, while one child had no change, and another one had a slight increase in the time of the daily use of ventilation. Fourteen patients (66.6%) were using permanent IMV (24 hours a day) at baseline; in only one, the daily time of ventilation was reduced by 4 hours after treatment. | Children who were feeding orally at the first dose, whether together with gastrostomy or not, continued to use it         Only four patients (19%) acquired speech, and all of these were under NIV. | n/a | n/a | n/a |
| Mendonca 2021b |  |  | Group 1 had a hospitalization rate of 0.1 hospitalisations per patient (SD = 0.4) for causes other than the administration of Nusinersen during 24 months of follow-up, whereas in the control group, the rate was 0.49 hospitalizations per patient (SD = 0.98). Although the reduction in the number of hospitalisations favoured treatment with nusinersen, the difference between the two groups was not statistically significant |  | N=12 non-ambulant patients treated with nusinersen, eight SMA type 2 and four SMA type 3) who had 18- or 24-month follow-up showed a progressive decline of disease |  |  | n/a |
| Meyer 2021 | n/a | n/a | n/a | n/a | n/a | n/a | n/a | TSQM-9    The Net promoter score (NPS) was analysed for the total group and the following cohorts of treatment duration: (i) less or equal to 12 months; (ii) 13 to 24 months; (iii) over 24 months. |
| Mirea 2021 | n/a | n/a | n/a | n/a | n/a | In the other five patients who received Nusinersen first (Patient 1NZ-5NZ), higher improvements in CHOP-INTEND scores were observed 6 months after T1 compared to the same period after T2. Ventilation hours were not modified 6 months after T1, but before T2, the median for the ventilation hours dropped. The median for cough assist number of sessions decreased after 6 months from T1 and 6 months after T2.     In the control group, for the 6-month period evaluated, slight improvements in ventilation were observed and insignificant changes in the cough assist session. | n/a | n/a |
| Mirea 2022 | n/a | n/a | n/a | n/a | n/a | n/a | n/a | n/a |
| Modrzejewska 2021 | n/a | n/a | n/a | n/a | n/a | n/a | n/a | n/a |
| Moshe-Lilie 2020 | n/a | n/a | n/a | n/a | n/a | n/a | n/a | n/a |
| NURTURE (NCT02386553) (Crawford 2023) | o For most PASA items, participants were consistently rated, on average, as never to rarely experiencing difficulty swallowing over a mean of 2.6 y.  o As of their last assessment, 92% (23/25) maintained the ability to swallow.  o At the participant’s last PASA assessment, 76% (19/25) of parents/caregivers “disagreed” or “strongly disagreed” with being concerned about their child choking, and 80% (20/25) “disagreed” or “strongly disagreed” with being concerned about their child aspirating on food while eating.  o All assessed children (10/10) with three SMN2 copies and the majority (67% [10/15]) with two copies were identified by PASA as not being tube-fed as of the last assessment.  o Of the five with two SMN2 copies ever identified by PASA as being tube-fed, two were “always” tube-fed, and three were “often” tube-fed in the 7 days preceding the last assessment.    o Two additional children had percutaneous gastrostomy tube placement, giving a current total of five with gastrostomy tubes, all with two SMN2 copies. | n/a | n/a | n/a | n/a | none required permanent ventilation.  o Four (16%) infants (all with two SMN2 copies) utilized respiratory intervention for ≥6 h per day continuously for ≥7 days (all during an acute, reversible illness).  o At the last study day prior to data cutoff, two of these infants no longer utilized respiratory intervention.  o The remaining two infants continued to receive respiratory intervention for two and 10 h per day, respectively, at the last study day prior to data cutoff.  o No other infants received respiratory intervention during the course of the study. | • Primary Outcomes: Survival and Respiratory Intervention:  o All 25 infants are alive; | n/a |
| Okamoto 2020 | n/a | n/a | n/a | n/a | n/a | n/a | n/a | n/a |
| Osmanovic 2021 | n/a | n/a | n/a | n/a | Symptom improvement under Nusinersen treatment: Reported following 64% of 51 Nusinersen administrations     Stable clinical condition and symptom deterioration: Stated in 18% each     Gradual decline in treatment effects: Stated subsequent to 23 (45.1%) of 51 Nusinersen administrations     Perceived wearing-off events: 23 reported by 16 of 28 (50.0%) enrolled patients     Patients consistently reporting a wearing-off phenomenon: 7     Patients who repeatedly reported no wearing-off at all: 8     Significantly higher BMI in patients reporting wearing-off: Mean 27.36 kg/m2 (SD 5.07) compared to those without wearing-off phenomena (mean 20.69 kg/m2, SD 5.08; p = 0.011)     Non-ambulatory patients in the group of patients with wearing-off phenomena: 85.7% vs. 25.0% in those with no wearing-off (p = 0.041)     Grade of self-reported treatment effect: Did not vary between the two groups (mean 5.29 (SD 2.14) vs. mean 5.75 (SD 2.12); p = 0.766)     Patients who experienced a wearing-off tended to receive the next Nusinersen dose a few days ahead of schedule: Compared to those with no wearing-off, but this observation missed statistical significance | n/a | n/a | n/a |
| Osmanovic 2021b | n/a | n/a | n/a | n/a | n/a | n/a | n/a | TSQM-1.4© results were correlated to changes in motor-function outcome measures     The change of motor function (RULM, HFMSE) was calculated as the difference between motor function at TSQM-1.4© assessment and motor function at Nusinersen treatment initiation (or, if missing, the first available score during treatment)     Treatment Satisfaction Questionnaire for Medication German version 1.4, TSQM-1.4©    Treatment satisfaction was first assessed after 2 months of Nusinersen treatment (end of loading-dose period) and followed up at least once again during the treatment course in this subgroup |
| Osredkar 2021 | n/a | n/a | n/a | n/a | Before the first application, 36/61 (59.0 %) patients had scoliosis     In the 14-month study period, 2 more patients developed significant scoliosis (1 SMN type I, 2 SMN2 copies, and 1 SMN type III, 3 SMN2 copies) | In the 14-month study period, 3 patients who did not need any kind of ventilatory support at baseline, started needing NIV during the night (1 patient was SMA type I, 2 SMN2 copies; 2 patients were SMN type II, 2 and 3 SMN2 copies) | n/a | n/a |
| Pandey 2025 | n/a | n/a | n/a | n/a | n/a | n/a | n/a | Nusinersen group:  ACEND Scores:  No significant change from baseline to 12 months (p = 0.80).  SOC group:  ACEND Scores:  No significant change over 1 year (p = 0.51).  Comparison: No significant difference between groups from baseline to 12 months. |
| Pane 2019 | n/a | n/a | n/a | n/a | n/a | n/a | n/a | n/a |
| Pane 2021 | At baseline, 32 patients were fed orally, and 36 had tube  feeding.  At 24 months, 24 patients remained orally fed, and 44  had tube feeding. Seven of the eight who had a tube  inserted after baseline were still able to swallow and eat  solids or semi-solids orally. There was no difference  between baseline and 24 months (P = 0.222). | n/a | n/a | n/a | n/a | n/a | Of the 125 patients enrolled in the Italian EAP project, six  died within the first year and one in the second year (Fig. 5).  At baseline, of the 68 patients reported in this study,  20 patients had a tracheostomy and seven used non-invasive  ventilation for more than 16 h/day. At 24 months,  23 patients had a tracheostomy and eight used  non-invasive ventilation for more than 16 h/day. There  was no difference between baseline and 24 months  (P = 0.862). | n/a |
| Pane 2022 | n/a | n/a | n/a | n/a | n/a | n/a | n/a | n/a |
| Pane 2023 | At baseline, 18/48 infants had a gastrostomy (G-tube) inserted, 5 had a nasogastric tube (NG-tube), another 2 were fed orally but had an indication for the G-tube at baseline, and the remaining 23 were fed orally without any problems.     At 48 months of the 23 fed orally at baseline, 20 remained stable and 3 showed a deterioration requiring tube feeding. The two patients with an indication of tube feeding at baseline and the 5 who already had an NG tube at baseline all had a G-tube inserted within 4 weeks from the first infusion. The 20 patients with G-tube at baseline remained on G-tube, with 2 of them regaining the ability to be partially fed orally. | n/a | n/a | n/a | n/a | At baseline 13/48 had a tracheostomy, 2 used non-invasive ventilation (NIV) >10 h and 1 > 16 h, 18 used NIV less than 10 h and 14 were on spontaneous breathing.     Of the 14 on spontaneous breathing at baseline, 4 indicated NIV already at the time of referral and started NIV within 4 weeks from the first infusion. At 48 months, the remaining 10 on spontaneous breathing at baseline all remained stable but 4 started NIV < 10 h for prophylactic reasons. Fifteen of the 18 already using NIV <10 h at baseline remained stable, 2 showed an increase in the number of hours on NIV, and 1 was able to discontinue NIV.     One of the 2 patients already using NIV >10 h at baseline remained stable and 1 had a tracheostomy inserted. All 13 patients who had a tracheostomy at baseline remained overall stable but 2 were also able to be in spontaneous breathing 4–6 h/day. | n/a | n/a |
| Pane 2024 | Feeding at Last Follow-Up:  No need for tube feeding: 34 patients.  Tube feeding and oral feeding: 9 patients.  Tube feeding only: 32 patients.  Overall Need for Support at Treatment Initiation: Significant association with feeding outcomes (p < 0.001).  OrSAT Levels at Treatment Initiation: Significant association with feeding outcomes (p < 0.001).  CHOP INTEND at Treatment Initiation: Significant difference among feeding outcome groups (p < 0.001).  Type of Treatment: Unable to perform meaningful statistical analysis due to small sample size for certain treatments. | n/a | n/a | n/a | n/a | n/a | n/a | n/a |
| Peng 2025 | 20/41 (48.8%) reported improvement in cough/expectorate and/or swallowing | n/a | n/a | FVC, FEV1, FEV1/FVC, and PEF remained stable.  reduction in respiratory infections by 1–6 per year. | Scoliosis: Present in 20/39 (51.3%) before treatment; 18/31 (58.1%) after 14 months.  Median increase in Cobb's angle: 1° (range -11° to 39°). | n/a | n/a | n/a |
| Pechmann 2022 | At baseline, 14 children (5.5%) required tube feeding due to bulbar dysfunction.     During the observation period, five children (2.0%) additionally required tube feeding despite Nusinersen treatment. | n/a | n/a | n/a | n/a | At baseline, 39 children (15.2%) used part-time (< 16 h per day), non-invasive ventilator support.     During the observation period, one older sitter was able to discontinue part-time ventilator support.     Twenty-three children (9.0%) additionally started to use occasional ventilator support.     None of the children required permanent or invasive ventilator support. | n/a |  |
| Pechmann 2023 | The probability of the need for tube feeding increased from 23.9% at baseline to 52.5% at m38 in cohort 1a and from 43.6% at baseline to 51.4% in cohort 1b at the last observation. | n/a | reported as part of the AEs | n/a | n/a | the probability of the need for any ventilator support increased from 29.5% at baseline to 59.7% at m38 and from 70.9 to 79.0% in cohorts 1a and 1b, respectively.     Five children (3.5%) [three children in cohort 1a (3.4%) and two children in cohort 1b (3.6%)] became additionally dependent on invasive ventilation. Of these, two children required permanent ventilation (>16 h/day). | In addition to the five children who became dependent on invasive or permanent ventilation, three children (2.1%) died during the observation period (two children in cohort 1b and one child in cohort 1a): mean age at death was 46.3 months (range 13–74 months) and mean treatment duration was 24.3 months (range 11–36 months).     The cause of death was acute respiratory tract infection in one child and clinical deterioration due to previous respiratory tract infections in the remaining children. | n/a |
| Pechmann b 2023 | At baseline, none of the patients required ventilator support or tube feeding. | n/a | n/a | At baseline, none of the patients required ventilator support or tube feeding. Mean FVC (%) was normal at baseline and remained stable in both cohorts. FVC (%) at month 38 was on average 93.3 [86.1; 100.5] (n = 17) in paediatric walkers and 96.3 [93.5: 99.1] (n = 39) in adult walkers | The symptoms of fatigue decreased in both cohorts during the 38-month follow-up period. | n/a | n/a | n/a |
| Pera 2021 | n/a | n/a | n/a | n/a | n/a | n/a | n/a | n/a |
| Pinar 2024 | n/a | n/a | n/a | n/a | Improvement in HFA: −0.95 to −0.65 (p = 0.015).  Decrease in BMI: 0.08 to −0.54 (p = 0.015). | n/a | n/a | n/a |
| RAINBOWFISH (NCT03779334) (EMA 2023) | All patients (100%) had the ability to feed orally and were fed exclusively by mouth.     All patients (100%) had the ability to swallow and were able to swallow purees.     At the time of the CCOD, 6 of 7 patients (85.7%) were reported to be able to swallow solid food. One patient (14.3%) with ≥4 SMN2 copies were reported to not be able to swallow solid food; however, after the CCOD, this was determined to be a data error and this patient was able to swallow solid food    Achievements after 12 months of treatment with Risdiplam: All patients maintained their abilities to feed orally and to swallow. |  | No patients required any overnight hospitalization.    No patients required any overnight hospitalization throughout the 12 months of treatment. |  | n/a | No patients required permanent ventilation. All patients (100%) were alive without permanent ventilation at the time of the CCOD. | n/a | n/a |
| Rich 2022 | n/a | n/a | n/a | n/a | n/a | n/a | n/a | n/a |
| Sarikaya 2022 | n/a | n/a | n/a | n/a | n/a | n/a | n/a | n/a |
| Scheijmans 2022 | Three children had to start tube feeding during treatment, all after six injections.     None of the children with SMA type 3a required tube feeding or mechanical ventilation prior to treatment, which did not change during treatment. | n/a | n/a | n/a | n/a | Ten children with SMA type 1 did not receive mechanical ventilation at treatment initiation. Three of them started non-invasive mechanical ventilation (NIV) during treatment, despite improvements in motor function in all three. Two out of 4 children with SMA type 1c who received invasive ventilation prior to treatment, reduced their ventilation time from 22h/d to 13 and 12h/d, respectively.   One child with SMA type 2 started nocturnal NIV after four injections at the age of 9.5 years. None of the children with SMA type 3a required tube feeding or mechanical ventilation prior to treatment, which did not change during treatment. | n/a | n/a |
| Schneider 2021 | n/a | n/a | n/a | n/a | n/a | n/a | n/a | n/a |
| SHINE (CS11) (NCT02594124) (Dunaway young 2023) |  | Number of participants hospitalized [Time Frame: Up to Day 1814]     Duration of hospitalizations [Time Frame: Up to Day 1814] | NR | NR | Motor function outcomes were analysed according to the Cobb angle/scoliosis status  of participants at baseline.    • No apparent difference in scoliosis trajectories was seen among the three patient subgroups.    • The variability in Cobb angle was greatest at Day 930. Over time, the mean Cobb angle increased in all three subgroups by age.    • At Days 450 and 930, the distribution was skewed, and the mean Cobb angle was greater than the median.  • This was particularly evident for the equal more than 10 degrees subgroup, in which a few patients had increases in Cobb angle to >40 degrees at later time points.  • Cobb angle decreased for some participants, possibly attributable to variability in the assessment or following surgery.  • Between baseline and Day 930, Cobb angle increased to equal more than 40 degrees in 25 children, and 4 of the 25 underwent scoliosis surgery (spinal rod insertion, scoliosis surgery, or spinal fusion surgery).  • The majority were in the >20 degrees to <40 degrees (moderate scoliosis) Cobb angle at the baseline subgroup.  • Of these participants, four developed a Cobb angle equal to more than 40 degrees by Day 450, and the remaining participants reached that level before Day 930.  • As a reference, the median change in Cobb angle from the baseline of CHERISH to a baseline of SHINE was 5.9 degrees in the sham control arm and 1.2 degrees in the Nusinersen arm (Supplementary Materials, Figure S2). |  | Median time to death or permanent ventilation for infants who received Spinraza in ENDEAR and SHINE was 73.0 weeks (95% CI: 36.3, NA).     The median time to death or permanent ventilation in patients treated with Nusinersen in SHINE and ENDEAR was 73.0 (95% CI, 36.3–not available) weeks, and 22.6 (95% CI, 13.6–31.3) weeks in those who received either sham control in ENDEAR or sham control in ENDEAR and Nusinersen in SHINE.     Of the patients who received sham control in ENDEAR and Nusinersen in SHINE (n=24), 12 were alive without permanent ventilation at baseline in the SHINE study. Of these 12 patients, 7 (58%) were alive and without permanent ventilation at the time of the data cut-off (median time on study in SHINE: 9.2 months).’ | Change from Baseline in Quality of Life (QOL) Questionnaires [Time Frame: Up to Day 1814] |
| Simic 2022 | n/a | n/a | n/a | n/a | n/a | n/a | n/a | n/a |
| Simsek 2024 | n/a | n/a | n/a | n/a | n/a | n/a | n/a | V1 and V2 (15 months of treatment) |
| Sitas 2024 | All patients reported mouth-opening limitations indicative of temporomandibular joint (TMJ) contracture.  13 patients reported swallowing difficulties.   0 patients reported bulbar function worsening over the course of treatment.   9 patients (60% SMA2 patients) reported improvement in at least one tested item.   Improvements were most frequently registered in swallowing, talking, and chewing. Further improvements were registered in facial expressions, easier singing followed by better yawning and easier laughter.   2 patients (SMA type 3) reported swallowing difficulties during QOL evaluation, of which one reported improvement with treatment. | n/a | n/a | (10/15) SMA type 2 patients reported subjective respiratory improvement:   Most frequently are:  easier breathing (5 patients)  Better cough (3 patients)  Stronger voice (3 patients)  Reduced frequency of respiratory infections. | Weight:   9/31 = significant weight gain (more than 5% of body weight)  6 patients = subjective feeling of an increased appetite.     No correlation between initial body weight and Risdiplam efficacy (Pearson correlation coefficient, PCC –0.02), or SMA3 weight change and RULM change (PCC –0.062).   Moderate negative correlation between SMA2 weight change and RULM change (PCC –0.545).     Weight gain was not registered in walkers.   40% of SMA2 patients gained weight (6/15)  18.8% of SMA3 patients (3/16, all sitters). | n/a | n/a | Individualized Neuromuscular Quality of Life questionnaire (INQoL) assessed changes in quality of life.   Reported before and after starting treatment. |
| SM201 (EMA 2017) | n/a | n/a | n/a | n/a | n/a | n/a | No subjects died or had respiratory intervention     n=1 ventilation for ≥6 hours/day continuously for ≥1 day (4 to 6 hours for 9     continuous days) to treat an SAE of respiratory distress | n/a |
| SUNFISH (NCT02908685) (Mercuri 2022, Mercuri 2023, Oskoui 2023) | n/a | n/a | n/a | • No clinically significant changes in respiratory function over 24 months. | n/a | n/a | n/a | n/a |
| Szabo 2020 | n/a | n/a | n/a | n/a | n/a | n/a | n/a | n/a |
| Tachibana 2022 | n/a | n/a | n/a | n/a | The number of patients with gastrostomy before the start of Nusinersen treatment was 63 of 401 (15.7%), including 50 of 126 (39.7%) with infantile-onset SMA and 13 of 275 (4.7%) with later-onset SMA. baseline data only- no results post-treatment | PAV was introduced in 6 (2.0%) of 303 patients after the first administration of Nusinersen treatment on days 7, 9, 98, 104, 168, and 316 respectively.  This includes 5 out of 42 patients with infantile-onset SMA and 1 out of 261 patients with later-onset SMA.  Of the 104 patients who received PAV (starting before or after Nusinersen initiation), it was stopped in two patients with infantile-onset SMA on days 2 and 68 after the first administration. | n/a | n/a |
| Tachibana 2023 | n/a | n/a | n/a | n/a | n/a | A Kaplan–Meier analysis of time to death or permanent assisted ventilation was conducted; however, the median time to death or permanent assisted ventilation could not be determined due to an insufficient number of events. Excluding those who were already receiving permanent assisted ventilation at the start of Nusinersen, it was introduced in 9/409 (2.20%) patients on specific days after initiating Nusinersen. patient with infantile-onset SMA who had permanent assisted ventilation introduced on day 174, it was stopped 62 days after    2/113 patients who received permanent assisted ventilation starting before Nusinersen, stopped and 10 days after initiating Nusinersen. | n/a |  |
| Trancho 2024 | n/a | n/a | n/a | n/a | n/a | n/a | n/a | n/a |
| Trucco 2023 | Tongue strength: children:  Assessed in 32/45 (71%) children.  Below normative data in 15/32 (47%) patients.  Children 8 and 10 years had the highest prevalence of reduced tongue strength.  The median tongue strength for the whole paediatric cohort was at the very low end of normal (z-score −1.93)   The measure of active mouth opening was available in 44/45 (98%) paediatric patients.  19/44 (43%) children had an active mouth opening below the age-adjusted normative data.   Median active mouth opening for the whole paediatric cohort was at the lower end of normative values (z-score −1.65). Sitters had an abnormally reduced mouth opening (z-score −2.67).  Walkers had a normal mouth opening (z score −0.36)   TOMASS was available in 38/45 (84%) paediatric patients.  All children required a normal number of bites to eat a standard cracker.  The median number of masticatory cycles in the paediatric population considered was normal (z-score −0.30).  Mastication was more effective in children who were ambulant than in sitters.  The number of swallows required to eat a standard cracker was abnormally elevated (z-score 4.60) in both walkers and sitters. The time to eat a cracker and clear the mouth was at the higher range of normality in the whole paediatric cohort (1.83). Patients who were functionally sitters required a prolonged time to eat a standard cracker (z-score 3.26), whereas walkers required a normal time to eat a cracker (z-score −1.58)   The SMA-HI questionnaire was available in 17 children. Swallowing issues were reported as “burdensome” in 2 of them (12%).  NB. MORE OUTCOMES BUT NEEDS CONDENSING | n/a | n/a | n/a | n/a | n/a | n/a | Patient-Reported Outcome Measures (PROMs):  - The Spinal Muscular Atrophy Health Index (SMA-HI)  - Italian Version was used. This scale has been developed as a patients’ reported disease burden outcome using FDA guidelines for SMA patients from 12 to 85 years (recently translated and validated in Italian). The higher the SMA-HI score, the worse is the patient perception of burden |
| Trucco 2024 | n/a | n/a | n/a | Progression of Respiratory Function  FVC% Pred. Decline:  SMA type 2: 2.3% annually in treated vs 3.9% in NH (p = 0.08).  SMA type 3: 2.6% annually in treated vs 3.4% in NH (p = 0.59).  Age at FVC% Pred. <60%: Treated patients reached this threshold later than untreated (12.1 vs 10 years, p = 0.05).  FVC Absolute:  SMA type 2: Annual increase of 0.07 L in treated vs 0.05 L in NH (p = 0.32).  SMA type 3:Annual increase of 0.13 L in treated vs 0.11 L in NH (p = 0.67).  Changes in Respiratory Outcomes at 12 and 24 Months  12-Month Follow-Up:  FVC% Pred. Stable/Improved: 64.7% of treated SMA type 2 vs 35.7% of untreated.  Median Change in FVC%: +2% in treated vs −5% in untreated.  24-Month Follow-Up:  FVC% Pred. Stable/Improved: 50% of treated SMA type 2 vs 24.1% of untreated.  Median Change in FVC%: −1% in treated vs −9% in untreated.  SMA Type 3: Trend toward lower decline in FVC% over 1-month follow-up, but no further comparison due to low sample size.  NIV Use  1-Year Post-Treatment:  4.2% of treated SMA type 2 patients discontinued NIV.  3.6% of treated SMA type 2 patients started using NIV.  NH Cohort:  All SMA type 2 patients on NIV at baseline remained on NIV.  6.4% of SMA type 2 patients not on NIV at baseline started using NIV. | n/a | n/a | n/a | n/a |
| Tscherter 2022 | Ventilatory Support and Swallowing Abilities in SMA Type 1:     Patients aged < 18 months at treatment start (5 type 1b and 1 type 1c):     None required non-invasive ventilation (NIV) or tube feeding at treatment initiation.     After 8–30 months of treatment, 4/6 patients needed tube feeding.     Three of these four also started NIV < 16 h/day (nocturnal NIV), almost at the same time as the tube feeding.     Tube feeding and ventilation became necessary during or after a respiratory infection at the age of 12–23 months.     Swallowing was regained, but gastrostomy feeding became necessary.     Two of the patients required neither ventilation nor tube feeding at the last assessment.     No statistically significant difference was found between patients with or without the need for ventilation, or tube feeding, at the last assessment and the change in CHOP-INTEND under treatment.     Patients with a disease duration of 5–16 years before the start of treatment:     One patient required NIV, one patient needed tube feeding and three required both ventilation and tube feeding at treatment start.     One of those with 23–24 h/day NIV at treatment start was able to tolerate breaks of up to 2 h under treatment and showed a marked reduction of respiratory infections.     The other four patients had no change in ventilation support during the observation period. | n/a | n/a | n/a | n/a | Ventilatory Support and Swallowing Abilities in SMA Type 1:     Patients aged < 18 months at treatment start (5 type 1b and 1 type 1c):     None required non-invasive ventilation (NIV) or tube feeding at treatment initiation.     After 8–30 months of treatment, 4/6 patients needed tube feeding.     Three of these four also started NIV < 16 h/day (nocturnal NIV), almost at the same time as the tube feeding.     Tube feeding and ventilation became necessary during or after a respiratory infection at the age of 12–23 months.     Swallowing was regained, but gastrostomy feeding became necessary.     Two of the patients required neither ventilation nor tube feeding at the last assessment.     No statistically significant difference was found between patients with or without the need for ventilation, or tube feeding, at the last assessment and the change in CHOP-INTEND under treatment.     Patients with a disease duration of 5–16 years before the start of treatment:     One patient required NIV, one patient needed tube feeding and three required both ventilation and tube feeding at treatment start.     One of those with 23–24 h/day NIV at treatment start was able to tolerate breaks of up to 2 h under treatment and showed a marked reduction of respiratory infections.     The other four patients had no change in ventilation support during the observation period. | n/a | n/a |
| Van der Heul 2020 | Feeding problems in the palliative care group     N=3 infants (27%) coughed when drinking, but showed no wet breathing     N=7 (64%) demonstrated a combination of coughing and wet breathing     N=6 (55%) demonstrated sweating when feeding     N=4 s (36%) showed symptoms of regurgitation     N=3 s (27%) had  respiratory infections     Feeding problems in the Nusinersen group n=5     N=2 were clinically presymptomatic at enrolment and did not show symptoms      After the start of treatment with Nusinersen, sucking and swallowing improved     n=1 was able to breast-feed for a few minutes without symptoms of unsafe swallowing     n=3 showed symptoms of fatigue and unsafe swallowing at inclusion in the study. These symptoms initially decreased after the start of the treatment, but (re)appeared in all five infants between the ages of 8 to 12 months, requiring the start tube of feeding      All n=5 treated infants showed a deteriorating swallowing function between the ages of 8 to 12 months | n/a | n/a | n/a | n/a | n/a | n/a | n/a |
| van der Woude 2024 | n/a | n/a | n/a | n/a | n/a | n/a | n/a | n/a |
| Vazquez-Costa 2022 | n/a | n/a | n/a | At baseline: Mean (SD) untreated: 59.58 (39.33) treated: 72.86 (37.73)  Median (1st, 3rd quartile) for untreated: 44.5 (28.9, 87.75) for treated: 76.5 (39.5, 104.75)    between baseline and 6-month  visits: FVC% (n = 40) raw scores untreated:−1.09 (5.65) raw scores treated 2.6 (8.29) estimate 3.19 SE 2.11 P value 0.139    Effect of the interaction “treatment and follow-up  time” in the different outcomes at the last visit available for each  scale: FVC% (n = 48) OR 1.002 lower and upper 95% 0.9-1.116 p-value 0.975 | n/a | n/a | n/a | n/a |
| Vidovic 2023 | n/a | n/a | n/a | n/a | n/a | n/a | n/a | n/a |
| Vill 2021 | n/a | n/a | n/a | n/a | n/a | n/a | n/a | n/a |
| Wang 2024 | n/a | n/a | n/a | FVC% (Forced Vital Capacity):  No significant improvement in walkers and sitters, but a positive trend observed in sitters | n/a | n/a | n/a | n/a |
| Weaver 2021 | n/a | n/a | n/a | n/a | n/a | n/a | n/a | pre- and post-nusinersen exposure, Baseline difference with follow-up    PedsQL 3.0 Neuromuscular Module (NMM) with 25 items |
| Weber 2024 | n/a | n/a | n/a | n/a | n/a | n/a | n/a | n/a |
| Weststrate 2022 | p-FOIS Longitudinal Scores: The median p-FOIS declined from baseline. Median p-FOIS for type 1a remained at 1 from baseline and at all follow-up points. Median p-FOIS for type 1b declined from 3 at baseline to 1 at 6 months, and 2 at 12 and 24 months respectively. For patients with type 1c, median p-FOIS was 5 at baseline, 2 at 6 months after initiation of Nusinersen, and 3 at 12 and 24 months. | n/a | n/a | n/a | n/a | Median age at the start of NIV was 11 months for those with type 1a SMA, 6 months for type 1b, and 1 year 6 months for type 1c. Thirteen patients required NIV at baseline. At 24 months after initiation of nusinersen 21 patients required NIV. | n/a | n/a |
| Wohnrade 2023 | n/a | n/a | n/a | n/a | n/a | n/a | n/a | EQ-5D-5L  SF-36    HADS-D self-report tool for anxiety and depression    caregiver burden or CGB was measured using the ZBI, a structured interview |
| Yao 2024 | n/a | n/a | n/a | n/a | n/a | n/a | n/a | n/a |
| Yasar 2024 | n/a | n/a | n/a | n/a | Scoliosis  Prevalence: 93.1% (27 patients).  Development: Before 2 years of age in all patients with scoliosis.  Mean Cobb Angle: 39.3° ± 19.9° (range 14–88) at last follow-up.  ΔCobb: 12.7° ± 7.2° per year (range 3.5–27).  Curve Patterns: Single long c-shaped curve in 59.3% (16 patients), double thoracolumbar curve in 40.7% (11 patients).  No Significant Relationships: Between ΔCobb and age, CHOP-INTEND scores, age at first nusinersen dose, or total number of nusinersen administrations.  Pelvic Obliquity  Prevalence: 69% (20 patients).  Mean Value: 12.1° ± 5.5° (range 5–22).  ΔPO: 4.1° ± 2.6° per year (range 1.4–10).  Association: Always associated with concomitant scoliosis deformity.  No Significant Differences: In patient age, total nusinersen administrations, time of first dose, or CHOP-INTEND scores between patients with or without pelvic obliquity.  Hip Status  Prevalence: 60.7% (17 patients) with hip subluxation.  Mean MI: 83.4 ± 21.8 (range 40–100).  ΔMI: 31.3° ± 13.6° per year (range 10–50).  No Significant Differences | n/a | n/a | n/a |
| Yu 2024 | n/a | n/a | n/a | n/a | n/a | n/a | n/a | n/a |
| Zhang 2024 | n/a | n/a | n/a | n/a | n/a | n/a | n/a | n/a |

# Supplementary Table S13: Adverse events

| **Author name and year** | Adverse events of treatment |
| --- | --- |
| Alruthia 2021 | n/a |
| AlTawari 2024 | Pediatric Cohort:  Chest infections in 3 patients, severe scoliosis in 1 patient, mild/moderate scoliosis in 6 patients.  Mild elevations in prothrombin and activated partial thromboplastin time in 1 patient, resolved naturally.  Post-injection headaches in 1 patient, fibrous tissue formation at lumbar puncture site in 1 patient.  No serious adverse events related to nusinersen.  Adult Cohort:  Well-tolerated overall, mild restrictive lung disease in 1 patient. |
| Alves 2021 | n/a |
| Aragon-Gawinska 2020 | N=2 died due to respiratory failure  N=1 removed due to respiratory degradation |
| Arnold 2021 | n/a |
| Arslan 2023 | The most common: were the procedure injection site pain and post-lumbar puncture headache which were reported at least once in 53.1% (n = 17) and 43.75% (n = 14) of patients, respectively.  Headache intensity was mild (less than 4 according to the visual analogue scale score), and mostly resolved within 1–3 days.  Only one SMA type 2 patient (16.6%) required hospitalization and intravenous hydration due to severe headache after the second dose.  32.2% of the patients (n = 10) showed proteinuria at least once. Proteinuria was detected in seven patients at t3 and in three patients at t4. Proteinuria was mild (150–500 mg/g creatinine) in all cases and did not require discontinuation of the treatment. |
| Audic 2020 | Five patients with SMA type 1a/b died before completing a full year of treatment and one patient with SMA type 1c died after 1 year of treatment  One patient died after 14 months of treatment due to a viral cardiomyopathy.  There were 95 adverse events in 25 patients.  These consisted of technical difficulties for lumbar puncture (n = 55) with fluoroscopic guidance required in 17 cases, headache (n = 23), post lumbar puncture syndrome (n = 6), nausea and vomiting (n = 4), asthenia (n = 4), back pain (n = 2), and fever (n = 1). |
| Audic 2023 | n/a |
| Axente 2022 | n/a |
| Belancic 2023 | 437 injections of Nusinersen were administered.  ADRs profile showed the same safety and tolerability profile for Nusinersen as demonstrated in clinical trials; no new safety concerns have emerged. Headache, back pain, and vomiting were the only ADRs identified; their frequencies (≥1/10 patients) were similar to those presented in the summary of product characteristics  Lethal Event: There was one non-drug-related lethal event during the study period. It was an SMA type 1 patient (who was on BiPAP and needed a nasogastric tube for feeding) who died at 3.9 yr. of age and in whom CHOP INTEND improved from 12 to 44 after 13 doses of Nusinersen were received. |
| Berti 2022 | n/a |
| Bjelica 2023 | n/a |
| Brakemeier 2021 | n/a |
| Brakemeier 2024 | n/a |
| Cavaloiu 2024 | n/a |
| Cavaloiu 2024b | n/a |
| Chacko 2022 | n/a |
| Chen 2024 | n/a |
| CHERISH/ISIS 396443-CS4 (NCT02292537) (Mercuri 2018) | Nusinersen vs control  Overall incidence 93% vs 100%  Severe Adverse Events 5% vs 7%  Serious Adverse Events 17% vs 29%  Common AEs: lumbar puncture (back pain, headache, nausea, post lumbar puncture syndrome, procedural pain |
| Chiriboga 2016 | 89% of participants reported AEs  No SAEs  No AE led to discontinuation  2 AEs were considered potentially related to the study drug; a mild paraesthesia in the 1-mg group and a report of palpitations in the 3-mg group.  Most frequently observed AEs:  Headache (39.3% of participants)  post-LP headache (21.4% of participants)  Back pain (17.9% of participants)  Post-LP headache incidence 10.9%  “No clinically significant changes in vital signs, neurologic or physical examinations, clinical laboratory tests, or ECGs were reported”. |
| Chiriboga 2024 | Most common AEs: pyrexia (24%), upper respiratory tract infection (URTI, 21%), headache (18%). Most common SAEs: pneumonia (3%). No treatment-related SAEs in patients previously treated with nusinersen or onasemnogene abeparvovec.  Median duration of exposure to risdiplam: 26.8 months. 96% of patients reported ≥ 1 AE, 20% reported ≥ 1 SAE. Most common AEs: pyrexia (24%), URTI (21%), headache (18%). Most common SAEs: pneumonia (3%). No treatment-related SAEs in patients previously treated with nusinersen or onasemnogene abeparvovec. AE and SAE rates decreased over the duration of the study. No treatment-related safety findings led to withdrawal. Comparing the rates of the common AEs per 100 PY between the first and fourth 6-month treatment periods (0 to ≤ 6 months vs. > 18 to ≤ 24 months) showed declines in pyrexia (31.58 to 13.93), URTI (37.43 to 13.93), and headache (44.45 to 7.60); the rate of the most common SAE of pneumonia also decreased (4.68 to 1.27). A review of all available safety laboratory results, vital signs, electrocardiograms, and ophthalmologic assessments did not show any clinically significant adverse findings compared with baseline. As of the CCOD, there have been no safety findings in patients reflective of potential risks previously identified from non-clinical toxicology studies (effects on epithelial tissues, retinal toxicity, or hematologic effects).  Post Hoc Analyses of Safety Data  Comparison Between Non-Treatment-Naïve and Treatment-Naïve Patients  Type 1 SMA (JEWELFISH Study):  Overall AE Rate: 323.40 events per 100 PY (95% CI: 263.69–392.58)  FIREFISH Study (Treatment-Naïve): 465.53 events per 100 PY (95% CI: 436.20–496.39)  Types 2 and 3 SMA (JEWELFISH Study):  Overall AE Rate: 335.63 events per 100 PY (95% CI: 317.11–354.95)  SUNFISH Part 2 Study (Treatment-Naïve): 542.31 events per 100 PY (95% CI: 513.00–572.87)  Decline in AE and SAE Rates: Observed for both non-treatment-naïve and treatment-naïve patients over a 24-month period. |
| Cho 2023 | n/a |
| Colot 2024 | n/a |
| Coratti 2021 | n/a |
| Coratti 2024 | n/a |
| Cornell 2024 | No of AE: 60, in 34 patients, 15% (9/60) occurred in Type 1 SMA patients, and 85% (51/60) occurred in Type 2 SMA patients.  Causality of AEs:  64% (38/60) not related to Risdiplam  16% (10/60) related to Risdiplam  20% (12/60) causality was not specified  Most common AEs:  Respiratory tract infections: 35%  Gastrointestinal disturbance: 20%  Skin and hair abnormalities: 10%  4 severe life-threatening - Not related to Risdiplam by treating clinicians.  Two subsequent deaths.  Permanent cessation of Risdiplam treatment during EAMS: 3% of patients enrolled (3/92).  Two cessations were due to death.  Permanently cease treatment after seven months of taking Risdiplam due to two months of missed menstruation.  Risdiplam treatment was temporarily stopped in 6 patients during some adverse events (AEs). |
| CS2/CS12NCT01703988   (see also NCT02052791 an extension study)  Darras 2019 CS12 & CS2 | ≥1 AE while on study: n = 28 (100%)  Most common AEs (≥20% of children) were:  Post-LP syndrome (n = 16; 57%)  Headache (n = 13; 46%)  Nasopharyngitis (n = 12; 43%)  Upper respiratory tract infection (n = 12; 43%)  Puncture site pain (n = 11; 39%)  Back pain (n = 9; 32%)  Scoliosis (n = 8; 29%)  Pyrexia (n = 7; 25%)  Joint contracture (n = 6; 21%)  Rhinorrhoeas (n = 6; 21%)  Vomiting (n = 6; 21%)  Serious AEs (SAEs): n= 5 (18%)  SAEs included:  Post-LP syndrome (n = 2)  Lower respiratory tract infection, respiratory distress, and viral pneumonia (n = 1)  Acute respiratory failure and respiratory syncytial viral pneumonia (n = 1)  And vesicoureteral reflux and pyelonephritis (n = 1)  No SAEs were considered related to the study drug  No children discontinued treatment due to AEs |
| De Wel 2021 | At month 14 of the nusinersen administration  Back pain n=12  Headache n=3  Fatigue n=6  Increased appetite n=2  Myalgia n=7  Agitation n=3 |
| Duan 2022 | n/a |
| Duong 2021 | Nusinersen was well tolerated with 2 reported cases of mild thrombocytopenia (platelet counts between 100,000 and 150,000) that resolved spontaneously, and 3 cases of transient limb pain not directly related to the injection procedure that resolved within a few months  The most common adverse event post-LP: is a transient headache that in all cases resolved spontaneously. This occurred following 16 of 234 injections (6.8%) and affected 6 patients (14.3%)  Other infrequently reported adverse events included injection site pain, nausea/vomiting, light-headedness, and anxiety. No cases of hydrocephalus, bleeding/bruising, or renal compromise were identified. All adverse events resolved quickly without intervention  Four patients discontinued treatment: 1 for personal reasons despite continued successful injections, 2 for a combination of insurance limitations and lack of perceived ongoing effectiveness, and 1 due to bony overgrowth at the only thecal access site |
| Edel 2021 | n/a |
| EMBRACE (NCT02462759) (Acsadi 2021) | AEs, severe AEs, related to study treatment, leading to discontinuation, withdrawal recorded:  No AEs considered by investigators as related to study treatment and no study discontinuations due to Nusinersen-related AEs.  The most common AEs were consistent with conditions associated with childhood in general and SMA specifically (pyrexia, cough, pneumonia, upper respiratory tract infection  the most common AE occurring within 72 hours after lumbar puncture was vomiting (noted in 4 of 14 [29%] of the participants treated with Nusinersen in parts 1 and 2 vs 1 of 7 [14%] of participants in part 1 who were treated with the sham procedure; vomiting was not observed in any of the participants initially receiving sham procedure who received Nusinersen in part 2)  One patient discontinued the study in the control group due to a fatal AE  Primary endpoints:  AEs:  There were no clinically significant Nusinersen-related changes or patterns in electrocardiograms, vital signs, clinical laboratory testing results, neurological examination, or physical examination. No participants developed hydrocephalus or liver failure, clinically meaningful thrombocytopenia or urinalysis results, or detectable antibodies against Nusinersen during the study  Secondary endpoints:  Nusinersen concentration in plasma and CSF. The exploratory safety endpoint was the development of plasma antibodies to Nusinersen:  (Taken from Acsadi et al. 2021). ‘In participants treated with Nusinersen in both study parts, mean pre-dose CSF concentration ranged from 3.9 ng/mL at day 15 to 11.32 ng/mL at day 898. In those originally randomized to sham procedure who received Nusinersen during part 2, the mean range was 4.8 ng/mL at day 29 to 8.4 ng/mL at day 540 (Figure 2A). The pre-dose CSF concentrations and concentration-time profiles in participants with infantile- and later-onset SMA were consistent with each other. |
| ENDEAR/ISIS 396443-CS3B (NCT02193074)  (Finkel 2017) | Nusinersen vs Control  Overall incidence of AEs: 96% and 98%  Severe AE: 56% vs. 80%  Serious AE: 76% vs. 95%  Adverse event that led to discontinuation: 16% vs. 39% |
| European registries (NICE 2020) | Treatment discontinuation due to AE n (%) n=2 (1.3%) |
| Fainmesser 2022 | The only adverse event reported was post-lumbar puncture headache. 3 patients were seen in the emergency department and treated with IV analgesics with resolution of symptoms.  Few patients described increased appetite and mild weight gain.  One patient was diagnosed with Crohn’s disease and one patient was diagnosed with pre-diabetes, which was most likely not treatment-related.  No proteinuria, hypercoagulability, or abnormal blood count was detected. |
| Finkel 2021 | In the safety population, steady growth was observed in arm and chest circumference.  Changes in growth parameters for participants in cohort 2 with two SMN2 copies are shown in the appendix (pp 2 and 8).  Correlation analyses between outcome measures are also shown in the appendix (pp 2–3 and 10).  In the safety population, 101 serious adverse events were reported in 16 (80%) of 20 participants.  All five deaths that occurred during the study (one in cohort 1 and four in cohort 2) were due to serious adverse events that were likely related to spinal muscular atrophy disease progression.  None of the serious adverse events or deaths were considered related to study treatment or lumbar puncture procedure.  Data on ancillary medical procedures and mortality among participants in cohort 2 by SMN2 copy number are shown in the appendix (p 11).  The most frequently reported serious adverse events concerned the respiratory system, consistent with events typically observed in children with spinal muscular atrophy type I.  In cohort 2, the incidence of adverse events and serious adverse events tended to decrease over time.  Incidence of adverse events and serious adverse events (including related hospitalisations) of the respiratory, thoracic, and mediastinal system organ class decreased from 69% (11 of 16 participants) and 31% (five of 16 participants) between days 1 and 90 to 20% (two of ten participants) and 10% (one of ten participants) between days 991 and 1080.  Similarly, the incidence of adverse and serious adverse events of the gastrointestinal system decreased from 63% (ten of 16 participants) and 6% (one of 16 participants) between days 1 and 90 to 30% (three of ten participants) and 0% between days 991 and 1080 |
| FIREFISH (NCT02913482) (Masson 2022) | A total of 86 disease-related AEs was experienced by 32 infants (78%) at Month 24 in the Masson Appendix study.  • In the FIREFISH part 2 study (Masson), a total of 356 AEs were reported up to the 24-month clinical cutoff date.  • The most frequently reported AE was upper respiratory tract infection, in 22 infants (54%).  • In the FIREFISH Part 2 study (CADTH), at least 1 AE was reported in all enrolled infants.  • The most common AEs were upper respiratory tract infection (46.3%), followed by pneumonia (39.0%), pyrexia (39.0%), and constipation (19.5%).  • In the Baranello study, serious AEs included pneumonia, respiratory tract infection, and acute respiratory failure.  • The most frequent AE was upper respiratory tract infection (n=32, 55%) and the most frequent SAE was pneumonia (n=20, 34%) followed by respiratory distress (n=5, 9%) (post-hoc analyses).  Serious Adverse Events (SAEs):  • A total of 68 SAEs were experienced by 28 infants (68%) in the FIREFISH part 2 study (Masson).  • The most frequently reported SAE was pneumonia in 16 infants (39%).  • In the Serious Adverse Events study, a total of 48 SAEs were reported; the most common events included pneumonia (in 13 infants) and bronchiolitis, hypotonia, and respiratory failure (in 2 infants each).  • In the FIREFISH Part 2 study (CADTH), SAEs were reported in 58.5% of the infants (24 out of 41 infants); most of the SAEs were related to respiratory problems or respiratory infections.  • In the Baranello study, a total of 24 SAEs had been reported as of the clinical data-cutoff date.  • The most common SAEs were pneumonia (in three infants) and respiratory tract infection, viral respiratory tract infection, acute respiratory failure, and respiratory distress (in two infants each).  Deaths:  • Three infants died during the study in the FIREFISH Part 2 study (CADTH). Two were attributed to pneumonia and 1 to respiratory failure.  • In the Baranello study, 4 infants had died of respiratory complications at the time of publication.  • Three infants had died at the time of the data cutoff.  • After the data-cutoff date, one infant was withdrawn from Risdiplam treatment on day 585 at the request of the parents, and the infant died approximately 3.5 months after withdrawal owing to a respiratory complication related to spinal muscular atrophy.  • No additional deaths have been reported in part 1 of the study.  • One infant had a serious adverse event of grade 4 neutropenia in the context of serious pneumonia in the Baranello study.  • Laboratory results, vital signs, and electrocardiograms that were assessed at prescheduled time points did not show clinically significant adverse findings as compared with baseline in the Baranello study.  • Hematologic variables remained stable over time; any abnormal results were present at baseline or returned to within normal ranges without change to the dose of study medication.  • Bone marrow suppression was not observed.  • One event of eczema was ongoing at the clinical data-cutoff date.  • Ophthalmologic assessments showed age-related immaturity of the retina (isolated retinal cysts), which resolved with increasing age.  There were no findings suggestive of Risdiplam-induced retinal toxic effects, as had been observed in a study in monkeys treated with Risdiplam (at higher exposures than were tested in this study) |
| Freigang 2021 | n/a |
| Freigang 2021b | n/a |
| Freigang 2023 | n/a |
| Gaboli 2025 | Two type 1c patients discontinued treatment due to drug administration challenges and limited benefits. One type 1b patient required surgery for severe symptomatic hydrocephalus. No other adverse events or deaths reported |
| Gaffar 2022 | A total of 202 adverse events and 24 serious adverse events occurred in 14.8 months of Risdiplam treatment.  The most common serious adverse events were respiratory related in the form of pneumonia, respiratory tract infection, respiratory distress, and respiratory failure. Some of these adverse events could not be differentiated from SMA-related illnesses.  There was one Risdiplam-specific toxicity of the skin in the form of an eczema.  No retinal or bone marrow toxicity was reported. |
| García de la Banda 2020 | n/a |
| Goedeker 2021 | N=1 treatment-related serious adverse event (emesis leading to intubation) occurred during general anaesthesia  N=2 children had asymptomatic increased intracranial pressure  No patients discontinued treatment due to adverse events  Summary of laboratory abnormalities  Any: n=45 of which were significant n=21 (0.7%)  Proteinuria: n=20 of which were significant n=18 (3.8%)  Thrombocytopenia: n=3 of which were significant n= 0 (0%)  Thrombocytosis: n=14 of which were significant n= 0 (0%)  PT elevation: n=13 of which were significant n=1 (0.2%)  INR elevation: n=5 of which were significant n= 1 (0.2%)  PTT elevation: n=16 of which were significant n= 1 (0.2%) |
| Gonski 2023 | n/a |
| Govoni 2024 | n/a |
| Gunther 2024 | 732 adverse drug reactions or procedure-related complications were documented in 353/389 (91%) screened patients who received at least one injection. Fifty-one (7%) patients had adverse drug reactions without a detailed description. No adverse drug reactions were reported in 103 (26%) patients, while 250 (64%) patients had at least one adverse reaction. There was no hint of more AEs in the group of worsening patients (improving: 75% vs. worsening: 64% ≥ 1 AE). Most adverse reactions were post-lumbar puncture syndrome, headache, back pain, and infections |
| Hagenacker 2020 | Included in 14-month analysis (n=61)  Total adverse reactions n=8  Total patients with adverse reactions n=6  Headache n=4  Back pain n=2  Nausea n=1  constipation n=1  To 14-month follow-up, the most frequent adverse effects among 173 patients were headache (61 [35%] patients), back pain (38 [22%]), and nausea (19 [11%]). No serious adverse events were reported |
| Hahn 2022 | Serious adverse events, n (%) (Percentages are based on the total number of AEs (30 in SMA1 and 100 in SMA2). MedDRA, Medical Dictionary for Regulatory Activities)  SMA1 SMA2 Total  Total number: 2 (6.7) 8 (8.0) 10 (7.7)  Gastrointestinal disorders: NR 5 (5.0) 5 (3.8)  General disorders and administration site conditions: NR 1 (1.0) 1 (0.8)  Infections and infestations: 2 (6.7) 2 (2.0) 4 (3.0)  Results  During the safety reporting period, a total of 130 AEs were observed in 44 case reports. For SMA1, 30 AEs were reported in 13 cases with 2 SAEs in 1 patient. For SMA2, 100 AEs were reported in 31 cases, including 8 SAEs in 2 patients.  No SAEs with a fatal outcome were reported in this CUP. Risdiplam treatment was permanently discontinued at the patient’s request in a single patient with SMA2, who suffered from SAEs with pre-existing diverticulitis, which was exacerbated 3 times during therapy.  Among the 3 patients with SAEs, one preschool child with SMA1 developed pneumonia twice during the reporting period. The patient recovered following hospitalizations and antibiotic therapy.  An adult patient with SMA2 and pre-existing diverticulitis, deep vein thrombosis, and pain, presented with fever, abdominal pain, two episodes of diarrhoea, constipation, and increased C-reactive protein levels. An abdominal computer tomography showed a segmented sigma diverticulitis with occult perforation and inflamed inhibition of the surrounding peritoneal lipid tissue, with the involvement of a neighbouring ileum fold. The patient recovered after hospitalization and antibiotic therapy.  In a preadolescent child with SMA2 initially treated with Nusinersen, serious oesophageal hypomotility was observed during Risdiplam treatment approximately 2 weeks after starting Risdiplam therapy, which persisted at the time of reporting.  The most common non-serious AEs in SMA1 were gastrointestinal disorders: diarrhoea was observed 3 times (10.0% of all AEs), and abdominal pain and salivary hypersecretion both occurred 2 times (6.7% of all AEs). In SMA2, diarrhoea was the most common non-serious AE (9 events, 9.0% of all AEs), followed by headache (6 events, 6.0% of all AEs), and aphthous ulcer, constipation, nausea, and circumstance or information capable of leading to a medication error (all 4 events, 4.0% of all AEs). |
| Huang 2024 | n/a |
| Ip 2024 | Scoliosis progressed across all subtypes, with mean annual progression rates of 5.2, 11.9, and 3.6 degrees in SMA1, SMA2, and SMA3 respectively |
| JEWELFISH (NCT03032172) (Chiriboga 2023) | • 92% of patients reported at least one adverse event (AE), with the most common being upper respiratory tract infection and pyrexia (fever), affecting 17% of patients.  • 14% of patients reported serious AEs (SAEs), with pneumonia being the most common, affecting 2% of patients.  • The occurrence of AEs and SAEs per 100 patient-years decreased during the second 6-month period compared to the first.  • Six patients had at least one SAE that led to dose modification or interruption, but none were related to Risdiplam.  • One patient, previously treated with olesoxime, had an SAE of supraventricular tachycardia, which was considered related to Risdiplam treatment but resolved without changes to the study drug.  • The overall exposure to Risdiplam was 17.0 months.  • In the first 6 months of treatment, the AE rate was 648.0 events per 100 patient-years, and in the 6–12 months period, it decreased to 270.2 events per 100 patient-years.  • The SAE rate also decreased from 33.9 events per 100 patient-years in the first 6 months to 18.3 events per 100 patient-years in the 6–12 months period.  • Safety laboratory results, vital signs, and electrocardiograms showed no clinically significant adverse findings compared to baseline.  • No significant safety issues related to non-clinical toxicology risks were found.  • Ophthalmologic monitoring did not reveal retinal toxicity and no drug-related safety issues led to patient withdrawal.  • No deaths were reported. |
| Jiang 2024 | 45 participants (90%) experienced AEs, mostly mild/moderate. Common AEs: upper respiratory tract infections (26%), pneumonia (22%), procedural pain (22%). Hematuria reported in 3 participants (6%). 11 participants experienced SAEs, most commonly pneumonia (9 participants), none related to nusinersen. No deaths reported. |
| Jiang 2024b | 218 PT signals involving 27 SOCs identified. High-frequency AEs included metabolism and nutrition disorders, psychiatric disorders, cardiac disorders, increased CSF pressure, positive CSF red blood cell count, neuromuscular scoliosis, cerebrospinal fluid reservoir placement, renal function abnormalities (abnormal urine protein/creatinine ratio, protein urine presence).  Table 3:  Serious Outcomes  Death: 598 (10.36%)  Disability: 24 (0.42%)  Hospitalization—Initial or Prolonged: 1803 (31.24%)  Life-Threatening: 74 (1.28%) |
| Kariyawasam 2021 | n/a |
| Kokaliaris 2024 | Reduction in the rate of SAEs with risdiplam compared to nusinersen  SAEs: Risdiplam showed a 57% reduction in the rate of SAEs (HR 0.43) compared to nusinersen. |
| Kolbel 2022 | n/a |
| Konersman 2021 | 5–18y old Cohort (n=19) >18y old Cohort (n=16)  Procedure-related headaches. 5–18y old n=4, >18y old n=8  Procedure-related nausea/vomiting. 5–18y old n=4, >18y old n=1  Pyrexia (all causes). 5–18y old n=5, >18y old n=1  Procedure-related back pain. 5–18y old N/A, >18y old n=7  Procedure-related meningitis. 5–18y old N/A, >18y old n=1  Elevated prothrombin time. 5–18y old n=12, >18y old n=4  Elevated activated partial thromboplastin time. 5–18y old n=5, >18y old n=7  Elevated cystatin C. 5–18y old n=1, >18y old N/A  Hospitalizations (all non-surgical causes). 5–18y old n=12, >18y old n=1  Pneumonia. 5–18y old n=3, >18y old N/A  Acute Respiratory failure. 5–18y old n=3, >18y old N/A  Hypoxia. 5–18y old n=2, >18y old N/A  Influenza. 5–18y old n=2, >18y old N/A  Otitis media. 5–18y old n=1, >18y old N/A  Enterovirus. 5–18y old n=1, >18y old N/A  Hematemesis. 5–18y old n=1, >18y old N/A  Anxiety/tachycardia. 5–18y old n=1, >18y old N/A  Dysphagia. 5–18y old n=1, >18y old N/A  Meningitis. 5–18y old N/A, >18y old n=1  G-tube and Tracheostomy. 5–18y old N/A, >18y old n=1 |
| Kotulska 2022 | Apart from the 2 fatal cases mentioned above, no serious adverse events were reported |
| Krosmark 2023 | n/a |
| Krupa 2023 | n/a |
| Kwon 2022 | Most common AEs: diarrhea (6.5%), pyrexia (4.5%), upper respiratory tract infection (3.2%). 73 (47.1%) patients reporting at least one AE and 14 (9%) reporting at least one SAE. Most common SAEs: pneumonia (1.9%), acute respiratory failure, constipation, deep vein thrombosis (DVT), hypokalemia (1.3% each). No deaths reported  Safety: 173 AEs and 31 SAEs reported. Most common AEs: diarrhea (6.5%), pyrexia (4.5%), upper respiratory tract infection (3.2%). Most common SAEs: pneumonia (1.9%), acute respiratory failure, constipation, DVT, hypokalemia (1.3% each). No deaths reported. Treatment-related AEs: constipation, diarrhea, headache (3 patients each), dizziness, insomnia, nausea (2 patients each). Two patients reported three SAEs related to risdiplam (constipation, systemic inflammatory response syndrome, DVT). Three patients withdrew due to AEs (abnormal behavior, vomiting, abdominal discomfort, constipation).  Diarrhea: Three out of 10 diarrhea events were assessed as related to risdiplam. These events occurred within the first month after starting treatment and resolved during the first 4 months with no changes to risdiplam treatment. One diarrhea event was an SAE caused by an enterovirus infection and deemed unrelated to risdiplam.  Constipation: Four events of constipation were reported, all occurring and resolving within the first month after starting treatment. Three events were deemed related to risdiplam. Two related events were non-serious and did not require changes to the study drug. One related event was an SAE requiring temporary interruption of risdiplam treatment. A fourth constipation event was an SAE deemed unrelated to risdiplam. |
| Landfeldt 2023 | n/a |
| Lavie 2021 | n/a |
| Lee 2022 | n/a |
| Lee 2023 | n/a |
| Lemska 2024 | Post-lumbar puncture headache, lower back pain  Nusinersen was generally well-tolerated. Common adverse events included post-lumbar puncture headache (reported in 10 patients, 13 times out of 138 punctures) and lower back pain (reported in 4 patients within 24 hours following the injection). One patient passed away due to respiratory failure from severe pneumonia, not classified as a complication of nusinersen administration |
| Li 2024 | n/a |
| Li 2024a | No significant adverse effects affecting treatment and quality of life  805 intrathecal injections performed. No significant adverse effects affecting treatment and quality of life. Some minor adverse events (back pain, vomiting, infections, alopecia, facial rash) mostly during the loading period |
| Li 2024b | 106 positive AE signals identified. Highest frequency reported SOC: general disorders and administration site conditions. Common clinical AEs: pneumonia, vomiting, back pain, headache, pyrexia, post-lumbar puncture syndrome. Unexpected serious AEs: sepsis, seizure, epilepsy, brain injury, cardiorespiratory arrest, cardiac arrest. |
| Lillien 2024 | n/a |
| Lusakowska 2023 | The procedure was generally well tolerated. Post lumbar puncture syndrome (PLPS) was observed in 198 of 1023 (19%) LPs  Patients with PLPS reported headaches, mainly of mild intensity.  - Back pain in 111 LP procedures (11%). Nausea in 41 patients (4%)  vomiting in 12 (1%)  1 (SMA1) = single hospitalization for severe back pain after LP  The LP procedure supported by CT or the C-arm fluoroscopy system was associated with a lower risk of PLPS compared with conventional intrathecal drug administration (11% vs. 22%, p < 0.00001)  A 26-year-old woman with SMA2 and a history of scoliosis surgery had a cerebrospinal fluid leak observed after a CT-guided injection at T10. The leak stopped within 1 h without intervention |
| Maggi 2020 | AEs were reported in 48 (41.4%) patients (6 SMA2 and 42 SMA3); the most frequent AE was post-procedure headache, observed at least once in 43/116 (37.1%) patients. Lumbar pain was reported in 10/116 (8.6%) patients, 7 of whom underwent imaging-guided lumbar punctures. Two patients with SMA3 reported transient (1–2 months) worsening of existing hand tremors, one after T0 and one after T14. One renal colic requiring hospitalisation occurred in a patient with SMA2 the day following T10. No relevant changes related to Nusinersen treatment were observed in laboratory tests, including serum creatinine. Except for cases requiring hospitalisation, AEs were mild or moderate and were judged not related to Nusinersen itself, but rather to the administration procedure |
| Mendonca 2021 | A small incidence of adverse events was reported; post-puncture headache was reported by different patients in three out of 140 procedures (2.4%) |
| Mendonca 2021b | • Complication rate = 4.2% (n=306)  • Mainly Included post-puncture headaches and lower back pain  • Sedation group other complications occurred, two episodes of respiratory depression and two episodes of extreme tachycardia (>180 bpm). |
| Meyer 2021 | n/a |
| Mirea 2021 | Before adding GT to the study group, no adverse reactions were observed after Nusinersen treatment was administered. In the control group, mild agitation occurred in two patients, probably due to headache post-LP. In the combined therapies group, one patient (Patient 7NZ) died 2 months after GT, after developing serious adverse reactions (bleeding, respiratory arrest, renal failure needing peritoneal dialysis for one month, intubation, and admission to the Intensive Care Unit). After GT, all other patients presented with fever, vomiting, lack of appetite, mild thrombocytopenia, and liver enzyme elevation. |
| Mirea 2022 | n/a |
| Modrzejewska 2021 | “The adverse events observed in patients were post-lumbar puncture syndrome in four patients (15.38%), respiratory tract infection in four patients (15.38%), increased liver enzymes after gastrointestinal infection in two patients (7.69%), and unsealed puncture site with temporary CSF leakage in two patients (7.69%)” |
| Moshe-Lilie 2020 | N=1 died of respiratory failure in the setting of pneumonia, shortly after treatment initiation  N=3 stopped treatment due to complications/lack of improvement  N=5 lumbar puncture headache  “Patients who declined treatment cited comorbidities such as kidney insufficiency from diabetes and thrombocytopenia, as well as unclear outcomes in adults with SMA treated with Nusinersen” |
| NURTURE (NCT02386553) (Crawford 2023) | (Taken from De Vivo et al. 2019). ‘TAEs were reported in 25/25 (100%) NURTURE participants. Twenty of 25 (80%) participants had mild or moderate AE.  No AEs attributed definitely to study/ intervention. 8/25 infants possibly attributed to study- all but one case resolved without treatment.’  (Taken from Crawford et al. 2023). ‘No new safety concerns were identified with 2 additional years of follow-up. No AE was considered by investigators to be study-drug related (Table S5). Ten (40%) participants had an AE considered possibly related to the study drug; all resolved despite continued treatment, except for proteinuria in one child and clonus in another. Twelve (48%) had one or more serious adverse events (SAEs); none were considered by investigators to be study-drug-related |
| Okamoto 2020 | n/a |
| Osmanovic 2021 | n/a |
| Osmanovic 2021b | Not applicable although there are some outcomes of the questionnaires addressing the side effects that have been affecting the satisfaction of patients that are extracted as the effect size and results |
| Osredkar 2021 | N=1 patient died in the study period and one discontinued treatment |
| Pandey 2025 | Most adverse events were mild and related to the intrathecal procedure  119 doses of Nusinersen administered to 16 patients. Mild adverse effects related to intrathecal procedure and drug. One death in Nusinersen group due to severe respiratory infection.  Procedure-related: Headache (22.7%), vomiting (23.5%), lumbar puncture site pain (20%).  Nusinersen-related: Thrombocytopenia (13.3%), raised urine spot protein creatinine ratio (33.3%), deranged coagulation profile (60%).  Severe respiratory infection in one child with SMA type 2, leading to death. |
| Pane 2019 | n/a |
| Pane 2021 | Of the 125 patients enrolled in the Italian EAP project, 6 died within the first year and 1 in the second year |
| Pane 2022 | n/a |
| Pane 2023 | no treatment-related serious adverse events  Headaches, pain, and nausea occurred in 10 of the 48 patients (20.8%). |
| Pane 2024 | n/a |
| Pechmann 2022 | In total, 144 AEs among 64 patients were reported during the observation period.  Of all AEs, 122 (84.7%) were AEs with hospitalization and 22 (15.3%) without hospitalization.  The most common type of AEs were respiratory tract infections (45.8%), followed by gastroenteritis (20.8%), post-lumbar puncture syndrome (9.0%), other type of infections (8.3%), fractures (4.1%), respiratory symptoms including respiratory distress and transient cyanosis (4.2%), pain (3.5%), abdominal symptoms including abdominal pain (1.4%), and others (2.8%).  No children died during the observation period.  None of the AEs were considered as related to drug treatment, but 31 (25.4%) were rated as possibly related to drug treatment by the treating physician. The latter included procedure-related symptoms such as post-lumbar puncture syndrome but also acute infections or respiratory symptoms. |
| Pechmann 2023 | In total, 239 AEs among 74 patients were reported during the observation period: 173 AEs in cohort 1a and 66 AEs in cohort 1b. Of all AEs, 179 (74.9%) were AEs with hospitalization and 58 (24.3%) without hospitalization.  The most common type of AE were respiratory tract infections (64.9%), followed by acute respiratory failure (5.9%), gastroenteritis (6.3%), abdominal symptoms including constipation, abdominal pain and gastritis (5.4%), epileptic seizures (3.3%) or febrile seizures (3.3%), other type of infections (2.9%), post-lumbar puncture syndrome (2.1%), aspiration (1.3%), nephrocalcinosis (0.8%) and others (3.3%). |
| Pechmann 2023b | 50 AE among 40 patients  The most common: are post-lumbar puncture syndrome (26.0%), fractures and accidents (36.0%), infectious diseases (16.0%), cardiac symptoms (6%), pain (2%), and others (14.0%). No patient died during the observation period. 16 AEs (32%) were considered as possibly related to drug treatment by the treating physician |
| Peng 2025 | Adverse Events: Reported by 20 patients; most common were lower back pain, headache/dizziness, and nausea/vomiting.  CSF Abnormalities: Reported by 3 patients; elevated white blood cell count or total protein level, but no associated neurological symptoms |
| Pera 2021 | No serious adverse events (S-AE) were reported at the time of data collection, most frequent AE were all related to the procedure (headache, nausea, back pain), and have not been reported consistently by all patients |
| Pinar 2024 | n/a |
| RAINBOWFISH (NCT03779334) (EMA 2023) | • One subject had a Grade 3 AE of cystoid macular edema, which was assessed as unrelated to Risdiplam treatment.  • 38.9% of patients had at least 1 AE related to Skin and subcutaneous tissue disorders, and 50.0% had at least 1 AE related to Gastrointestinal disorders.  • Two patients experienced a total of 2 treatment-related AEs: Diarrhoea and Skin discoloration, both were Grade 1 and non-serious.  • The overall AE rate was 558.2 events per 100 patient years.  • The safety profile of Risdiplam was comparable in patients who initiated therapy at different ages.  • Haematological parameters were stable over time.  • No Hy’s law cases were observed, and no AEs suggestive of liver abnormalities were reported.  • Isolated shifts in diastolic blood pressure, systolic blood pressure, temperature, and pulse rate were observed but were not clinically significant.  • Seven of 18 patients were followed up with an SD-OCT assessment until at least 12 months.  • The proportion of patients with at least 1 AE was 77.8%, with a total of 81 AEs reported.  • No deaths, no Serious Adverse Events (SAEs), and no Adverse Events (AEs) of Grade ε4 were reported up to the CCOD.  • A higher proportion of AEs in patients with ≥4 copies of SMN2 had an unresolved outcome, compared to patients with a lower number of SMN2 copies.  • Two patients experienced a total of 2 treatment-related AEs: Diarrhoea and Skin discoloration.  • One retinal abnormality was reported as an AE and attributed to retinal immaturity.  • An event of non-serious skin discoloration was considered by the Investigator to be related to Risdiplam.  • In the RAINBOWFISH study, there were 6 AEs of diarrhoea reported in 6 patients.  • One SAE of gastroenteritis norovirus was reported that was not discussed in the interim CSR. The MAH clarified that gastroenteritis was downgraded to non-serious.  Exposure-safety analysis  • The overall Adverse Event (AE) rate was 681.0 events per 100 patient-years (PY) during the first 6 months of treatment, which decreased to 401.1 events per 100PY during the second 6 months.  • An analysis of AEs, Serious Adverse Events (SAEs), and safety laboratory parameters was provided based on exposure quartiles. Only 2 Grade 3 AEs and only 2 SAEs occurred in patients in the highest exposure quartile.  • A lower dose (0.15 mg/kg) for infants under 2 months of age was proposed based on updated population pharmacokinetics (popPK) analysis.  • At the time of the cut-off date, 4 patients (22.2%) were reported to have received more than 10% above the planned dose (defined as an overdose). Adverse Events of Accidental overdose were reported for 2 of these patients, but these did not lead to any other AEs.  • Risdiplam is currently approved in the U.S., E.U., and over 90 international markets for the treatment of Spinal Muscular Atrophy (SMA). An estimated total of 2728 patients received Risdiplam from marketing experience.  • One new identified risk (cutaneous vasculitis) not categorized as important was identified. The benefit-risk profile of Risdiplam in the authorized indication remains favourable.  The worldwide exposure in patients below 20 days of age is unknown. However, in the United States, as of 8 May 2023, 47 babies below 20 days of age have been prescribed Risdiplam. |
| Rich 2022 | n/a |
| Sarikaya 2022 | The most common adverse event in post-treatment follow-up was restlessness in group 1 (n=42; 26%) and low back pain in group 2 (n=50; 58%) |
| Scheijmans 2022 | Scheijmans 2022 There were 82 treatment-related adverse events in 45 patients on a total of 934 injections. The most reported side effects were headache, back pain, pyrexia, and discomfort.  One patient developed aseptic meningitis one day after his first injection. He fully recovered and continued treatment with Nusinersen without further complications.  One child had an allergic skin reaction with angioedema of the lips shortly after the second and fourth injections.  Two children showed an increased intracranial pressure measured before their injection of Nusinersen. Both children were without concomitant clinical symptoms. Investigation with MRI and fundoscopy showed no signs of hydrocephalus. Both children continued treatment with Nusinersen.  One child died due to a non-treatment-related event after 10 injections.  Reported adverse events during treatment; Number of reported AE (n of patients):  (Allergic) skin reaction 5 (3)  Aseptic meningitis 1 (1)  Increased intracranial pressure (without hydrocephalus) 3 (2)  Headache 24 (20)  Back pain 10 (9)  Tingling feeling in back/leg 4 (4)  Discomfort 13 (6)  Pyrexia 13 (10)  Proteinuria 9 (8) |
| Schneider 2021 | n/a |
| SHINE (CS11)  (NCT02594124)  (dunaway Young 2023) | n/a |
| Simic 2022 | n/a |
| Simsek 2024 | n/a |
| Sitas 2024 | n/a |
| SM201 (EMA 2017) | At least 1 AE: 16/20 (80%)  2 subjects (10%) with 3 events experienced severe AEs  Most common AEs:  upper respiratory tract infection  pyrexia  nasopharyngitis  No deaths were reported, and no subjects discontinued study treatment or withdrew from the study  No subject experienced SAEs that led to discontinuation of study treatment or withdrawal from the study  More results are available in EMA 2017 p8  Included in integrated safety analysis Pool A |
| SUNFISH (NCT02908685)  (Mercuri 2022, Mercuri 2023, Oskoui 2023) | Summary of AEs: • Adverse Events (AEs): The majority of patients experienced at least one adverse event (AE) during the study. In the Risdiplam Arm, 91.7% of patients experienced at least one AE between months 12 and 24. In the Placebo Arm, 91.7% of patients experienced at least one AE while receiving a placebo. After switching to Risdiplam, 80.0% of patients experienced at least one AE.  • Serious Adverse Events (SAEs): In the Risdiplam Arm, 20.0% of patients experienced at least one serious adverse event (SAE) between months 0 and 12, and 20.8% experienced at least one SAE between months 12 and 24. In the Placebo Arm, 18.3% of patients experienced at least one SAE. After switching to Risdiplam, 5.0% of patients experienced at least one SAE.  • Overall, SAE Rate per 100 Patient-Years (PY): The overall SAE rate for Risdiplam-exposed patients showed consistent rates over time, ranging from 19.4 AEs/100 PY to 36.1 AEs/100 PY.  • Grade 3–5 AEs: In the Risdiplam Arm, 17.5% of patients experienced Grade 3–5 AEs between months 0 and 12, and 15.8% experienced Grade 3–5 AEs between months 12 and 24. In the Placebo Arm, 13.3% of patients experienced Grade 3–5 AEs. After switching to Risdiplam, 5.0% of patients experienced at least one Grade 3–5 AE.  • Withdrawals: No adverse events or serious adverse events led to withdrawal from treatment.  • CADTH for sunfish part 2: In the SUNFISH Part 2 study, at least 1 AE was reported in 111 (92.5%) and 55 (91.7%) of enrolled patients in the Risdiplam and placebo arms, respectively.  • AE Reporting and Examinations: Safety assessments included AE reporting, laboratory assessments, electrocardiography, anthropometric measurements, and physical examinations. Ophthalmological assessments were performed every 2 months due to observed effects on the retina in preclinical studies.  • Placebo-Controlled Period: There was no numerical difference in safety findings across assessed dose levels.  • All-Exposure-to-Risdiplam Treatment Period: The median exposure duration was 31.9 months, with a total of 737 AEs reported in 49 (96%) patients during this period. The AE rate per 100 patient-years (100PY) decreased over 24 months.  Mercuri2022: In this study, 120 patients received Risdiplam, and 60 received placebo. The incidence of serious adverse events was similar between treatment groups, except for pneumonia. No grade 5 (fatal) adverse events were reported, and no adverse events led to dose modification of Risdiplam. |
| Szabo 2020 | Medical conditions such as pneumonia, pneumothorax, bone fracture, leg pain, transient and mild thrombocytopenia, and UTI (Urinary Tract Infection) were occasionally observed during the treatment period. |
| Tachibana 2022 | occurred in 127 (31.7%) patients in the all-patient cohort, 47 of 126 (37.3%) patients with infantile-onset SMA, and 80 of 275 (29.1%) patients with later-onset SMA.  The most common AEs were headache (4.5%), pyrexia (4.2%), pneumonia (3.7%), bronchitis (2.5%), nausea (2.2%), and post-lumbar puncture syndrome (2.0%).  Serious AEs (SAEs) occurred in 46 (11.5%) patients, including 24 (19.0%) patients with infantile-onset SMA and 22 (8.0%) patients with later-onset SMA.  AEs leading to discontinuation occurred in 3 patients with infantile-onset SMA and 1 patient with later-onset SMA.  Priority surveillance items occurred in 1 (0.2%) patient for hydrocephalus, 5 (1.2%) patients for coagulation-related AEs, 3 (0.7%) patients for renal impairment-related AEs, 11 (2.7%) patients for hypersensitivity reactions, and 3 (0.7%) patients for QT interval prolongation.  Four patients started Nusinersen treatment before turning 2 months old; AEs occurred in 2 of these patients.  One patient with renal impairment as a comorbidity started Nusinersen treatment and reported AEs.  Twenty of 401 (5.0%) patients received ≥9 administrations of Nusinersen. The incidence proportion of AEs in patients who received <9 administrations (117/381 patients, 30.7%) was lower than in those who received ≥9 administrations (10/20 patients, 50.0%).  AEs occurred in 20 of 62 (32.3%) patients with ≥4 copies of the SMN2 gene.  AEs occurred in 2 of 14 (14.3%) patients aged ≥65 years at treatment initiation, 1 of 7 (14.3%) patients with hepatic impairment, 120 of 387 (31.0%) patients with advanced disease, and 2 of 6 (33.3%) patients with permanent assisted ventilation introduced.  Lumbar puncture-related AEs occurred in 43 of 401 (10.7%) patients overall, 12 of 126 (9.5%) patients with infantile-onset SMA, and 31 of 275 (11.3%) patients with later-onset SMA. |
| Tachibana 2023 | AEs occurred in 188/524 (35.9%) patients in the all-patient cohort:  - 75/153 (49.0%) patients with infantile-onset SMA  - 113/369 (30.6%) patients with later-onset SMA.  The most common AEs were:  - headache (5.0%), pyrexia (4.8%), and pneumonia (3.2%).  Serious AEs (SAEs): 74 (14.1%) patients, including 39 infantile-onset SMA patients and 35 later-onset SMA patients; pneumonia was the most common SAE (2.3% in the all-patient cohort).  - AEs leading to discontinuation: 7 (1.3%), 5 infantile-onset SMA patients and 2 later-onset SMA patients.  - AEs leading to death: 3 (0.6%) patients, including one (0.7%) infantile-onset SMA and 2 (0.5%) with later-onset SMA.  Priority surveillance items: 1 (0.2%) patient for hydrocephalus (causal relationship cannot be ruled out), 11 (2.1%) patients for coagulation-related AEs (causal relationship cannot be ruled out for 5/11), six (1.1%) patients for renal impairment-related AEs (causal relationship cannot be ruled out for 4/6), 17 (3.2%) patients for hepatic impairment-related AEs (causal relationship cannot be ruled out for 11/17), 25 (4.8%) patients for hypersensitivity reactions (causal relationship cannot be ruled out for 6/25), and six (1.1%) patients for QT interval prolongation (causal relationship cannot be ruled out for 1/6). Central nervous system-related AEs and effects on memory and learning were not observed during the period of the interim analysis.  Nusinersen Treatment:  - Seven patients with infantile-onset SMA started Nusinersen treatment before turning 2 months old, and AEs occurred in 6 of these patients (causal relationship cannot be ruled out for 3/6).  - AEs were reported by two patients with renal disease as a comorbidity when Nusinersen treatment was started (one patient with infantile-onset SMA who had renal disease of unknown severity and one patient with later-onset SMA who had severe renal disease); however, these were not related to the treatment.  AEs and Administrations:  - AEs occurred in 183/524 (34.9%) patients when they received <9 administrations.  - 162/524 (30.9%) patients received ≥9 administrations, and AEs occurred in 17 of these 162 (10.5%) patients.  SMN2 Gene Copies:  - 106/523 (20.3%) patients had ≥4 copies of the SMN2 gene.  AEs occurred in:  - 27/106 (25.5%)  - 158/408 (38.7%) patients with an SMN2 gene copy number ≥4 and <4  - 0/4 (0%) patients with other types of SMN2 copies  - 3/5 (60%) patients with unknown copy number.  Lumbar Puncture-Related AEs:  Lumbar puncture-related AEs occurred in 70/524 (13.4%) patients (24/153, 15.7% infantile-onset SMA and 46/369, 12.5% with later-onset SMA).  Malignancy AEs, breast cancer, lymphoma, and dermato-fibro-sarcoma protuberance were reported in one patient each.  Effects of Nusinersen: The effects of Nusinersen on growth, and the effect of differences in impurity profiles between drug substance batches on safety, were not reported. |
| Trancho 2024 | n/a |
| Trucco 2023 | n/a |
| Trucco 2024 | n/a |
| Tscherter 2022 | 15 patients (34%) had at least one adverse event other than complications due to lumbar puncture. These side effects include proteinuria, thrombocytosis, thrombocytopenia, coagulation disorder (low prothrombin) and ECG changes (mild repolarisation abnormalities and transient prolonged QT measures). Proteinuria, low prothrombin, thrombocytosis and thrombocytopenia were asymptomatic, mild and transient in all. The findings were not explained by concomitant medication or infection. Two patients had transient elevated opening pressure without clinical signs or ophthalmological findings of elevated pressure. Six patients had side effects caused by lumbar puncture like headache and lumbar pain.  Proteinuria – 6 (14%)  Thrombocytosis = 7(16%)  Thrombocytopenia = 1(2%)  Coagulation disorder = 2 (5%)  ECG changes = 2(%)  Lumbar Puncture = 6 (14%)  Other adverse events (elevated opening pressure) = 2 (5%) |
| van der Heul 2020 | n/a |
| van der Woude 2024 | n/a |
| Vazquez-Costa 2022 | 30 treated patients (77%) presented at least one AE during the follow-up.  Overall, 55 AEs were reported, mostly related to the administration procedure: 45 were mild (post lumbar puncture syndrome and back pain) and 10 were moderate (seven post lumbar puncture syndrome, two urinary retentions due to neurogenic bladder, one radial neurapraxia).  Two patients (5%) discontinued treatment due to AEs (repeated post-lumbar puncture syndromes) and another due to technically challenging lumbar punctures.  One treated patient started NIV during follow-up, after a respiratory infection that required hospitalization.  No clinically relevant laboratory changes were found.  Most treated patients (77%) presented at  At least one adverse event, mostly mild. |
| Vidovic 2023 | n/a |
| Vill 2021 | n/a |
| Wang 2024 | 30 patients treated with 152 injections over 1 year  46.77% (14/30) experienced at least one adverse reaction  Most common adverse reactions:  Headache: 13.82% (21/152)  Lumbar pain: 9.86% (15/152)  Median ALT, AST, and CK in serum remained stable  No serious adverse events except for two hospitalizations due to fractures from falls |
| Weaver 2021 | n/a |
| Weber 2024 | n/a |
| Weststrate 2022 | n/a |
| Wohnrade 2023 | n/a |
| Yao 2024 | Patients: 132 initiating nusinersen upon or after enrollment.  AEs: 62.9% (83 patients) experienced at least one AE, with 203 AEs recorded.  Incidence Rate: 2.23 AEs per patient-year.  Mild: 1.45 per patient-year.  Moderate: 0.52 per patient-year.  Severe: 0.26 per patient-year.  Most Frequent AEs:  Infections and infestations: 37.1% (49 patients).  Upper respiratory tract infection: 15.2% (20 patients).  Pneumonia: 9.9% (13 patients).  COVID-19: 6.1% (8 patients).  Procedural pain: 13.6% (18 patients).  Vitamin D deficiency: 6.1% (8 patients).  Post-lumbar puncture syndrome: 21.2% (28 patients).  Related AEs: Two cases (aseptic meningitis and myalgia), both mild with no sequelae.  Death: One patient with SMA type I died from asphyxia, unrelated to nusinersen. |
| Yasar 2024 | n/a |
| Yu 2024 | Number of events =1588  Types of report  Non-serious = 885  Serious = 703  Life-threatening = 29  Hospitalization–Initial or Prolonged = 304  Disability = 40  Death = 127  Congenital anomaly = 3  Required Intervention to Prevent Permanent Impairment/Damage= 0  Other serious and important medical events= 309  Distribution of reported adverse events by sex, age, etc  General disorders and administration site conditions, n=747  Gastrointestinal disorders, n=571  Infections and infestations, n=405  Injury, poisoning, and procedural complications, n=279  Nervous system disorders, n=204  Investigations, n=203  Musculoskeletal and connective tissue disorders, n=186  Respiratory, thoracic, and mediastinal disorders, n=172  Skin and subcutaneous tissue disorders, n=128  Product issues, n=88  Metabolism and nutrition disorders, n=85  Renal and urinary disorders, n=82  Cardiac disorders, n=67  Psychiatric disorders, n=63  Vascular disorders, n=39  Reproductive system and breast disorders, n=37  Surgical and medical procedures, n=22  Blood and lymphatic system disorders, n=19  Hepatobiliary disorders, n=17  Congenital, familial, and genetic disorders, n=12  Eye disorders, n=12  Neoplasms benign, malignant, and unspecified (incl. cysts and polyps), n=10  Endocrine disorders, n=7  Immune system disorders, n=6  Ear and labyrinth disorders, n=4  Social circumstances, n=4  Pregnancy, puerperium, and perinatal conditions, n=1 |
| Zhang 2024 | The primary adverse event outcome with the highest severity was mortality, with a rate of 18.1%. The other three outcomes included hospitalization (54.57%), life threatening diseases (2.29%), and disability (0.71%). Specifically, during the observation period, 377 cases (12.06%) of ADEs occurred within 0–7 days after medication, 225 cases (7.2%) within 7–28 days, 210 cases (6.72%) within 28–60 days, and 1122 cases (35.89%) occurred after more than 60 days of medication. However, it should also be noted that 1192 cases (38.13%) had incomplete records.  Mortality: 18.1%.  Hospitalization: 54.57%.  Life-Threatening Diseases: 2.29%.  Disability: 0.71%.  Timing of ADEs:  0-7 days: 12.06% (377 cases).  7-28 days: 7.2% (225 cases).  28-60 days: 6.72% (210 cases).  >60 days: 35.89% (1122 cases).  Incomplete records: 38.13% (1192 cases).  Top 5 preferred terms by Number of Cases:  Post-lumbar puncture syndrome: 441 cases.  Headache: 441 cases.  Pyrexia: 304 cases.  Dural pain: 298 cases.  Pneumonia: 294 cases.  Overview  Positive ADE Signals: Related to Nusinersen involving several systems, such as general disorders, administration site conditions, infections, and nervous system disorders.  Consistency with Previous Studies: ADEs observed in previous clinical studies (e.g., post-lumbar puncture syndrome, headache, pyrexia, pneumonia, back pain) are consistent with current findings.  Specific Findings  Post-Lumbar Puncture Syndrome: Less frequent in children compared to adults, likely due to the lumbar puncture procedure itself rather than Nusinersen treatment.  Proteinuria: Fewer adverse events in children than adults, possibly due to the inherent character of SMA rather than a response to Nusinersen.  Increased CSF Pressure: More likely in pediatric patients due to natural variability and compliance of the ventricular system. Repeated intrathecal administration may elevate the probability of symptomatic intracranial hypertension.  Respiratory Infections: Lower prevalence in adults than in children, with early-onset SMA patients having a higher incidence of adverse respiratory events |
